# Ruthenium(ii)-catalyzed olefination *via* carbonyl reductive cross-coupling†
†Electronic supplementary information (ESI) available: Experimental details. See DOI: 10.1039/c7sc04207h


**DOI:** 10.1039/c7sc04207h

**Published:** 2017-10-09

**Authors:** Wei Wei, Xi-Jie Dai, Haining Wang, Chenchen Li, Xiaobo Yang, Chao-Jun Li

**Affiliations:** a Department of Chemistry , FQRNT Center for Green Chemistry and Catalysis , McGill University , 801 Sherbrooke St. W. , Montreal , Quebec H3A 0B8 , Canada . Email: cj.li@mcgill.ca; b School of Chemistry and Chemical Engineering , Qufu Normal University , Qufu 273165 , Shandong , China

## Abstract

Natural availability of carbonyl groups offers reductive carbonyl coupling tremendous synthetic potential for efficient olefin synthesis, yet the catalytic carbonyl cross-coupling remains largely elusive. We report herein such a reaction, mediated by hydrazine under ruthenium(ii) catalysis. This method enables facile and selective cross-couplings of two unsymmetrical carbonyl compounds in either an intermolecular or intramolecular fashion. Moreover, this chemistry accommodates a variety of substrates, proceeds under mild reaction conditions with good functional group tolerance, and generates stoichiometric benign byproducts. Importantly, the coexistence of KO^*t*^Bu and bidentate phosphine dmpe is vital to this transformation.

## Abstract 2

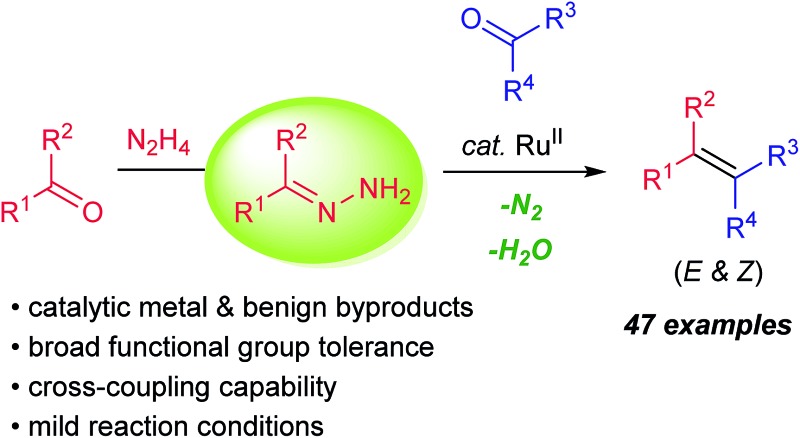
A catalytic olefination method *via* carbonyl reductive cross-coupling was achieved by ruthenium(ii) catalysis.

## 

Efficient construction of carbon–carbon double bonds has long been a central pursuit in the synthetic community. Recent decades have witnessed impressive accomplishments in the field of carbonyl olefination.^1^–^6^ Well-known milestones include, among others, the Wittig reaction,^4^ the Peterson reaction,^5^ the Julia olefination^6^ and the Tebbe–Petasis olefination.^7^ Parallel to these classical olefination methods, the McMurry reaction mediated by low-valent titanium (LVT) reagents enables direct reductive homo-couplings of carbonyl compounds for facile synthesis of olefins (Scheme 1, eqn (a)).^8^ Mechanistically, the synergy between oxophilic titanium(iii)/(iv) and strong metal reductants (*e.g.* LiAIH_4_ and alkali metals) is crucial to form metal pinacolates as key intermediates. One problematic scenario pertaining to the original protocol, however, is that the cross-coupling of two unsymmetrical carbonyl compounds generally affords a statistic mixture of coupling products.^8^ Despite McMurry-type variants (*e.g.* external ligands and auxiliaries) having been developed to bypass this issue,^9^ two challenges endure: (1) stoichiometric quantities of metal wastes accompanied by the excessive usage of metal-based reagents, and (2) poor chemoselectivity and functional group tolerance stemmed from the presence of strong metal reductants. As the synthetic community calls for more sustainable and efficient chemical syntheses, carbonyl cross-coupling represents an ideal strategy to access olefins because naturally prevailing carbonyl functionalities are generally regarded as renewable feedstocks.

**Scheme 1:**
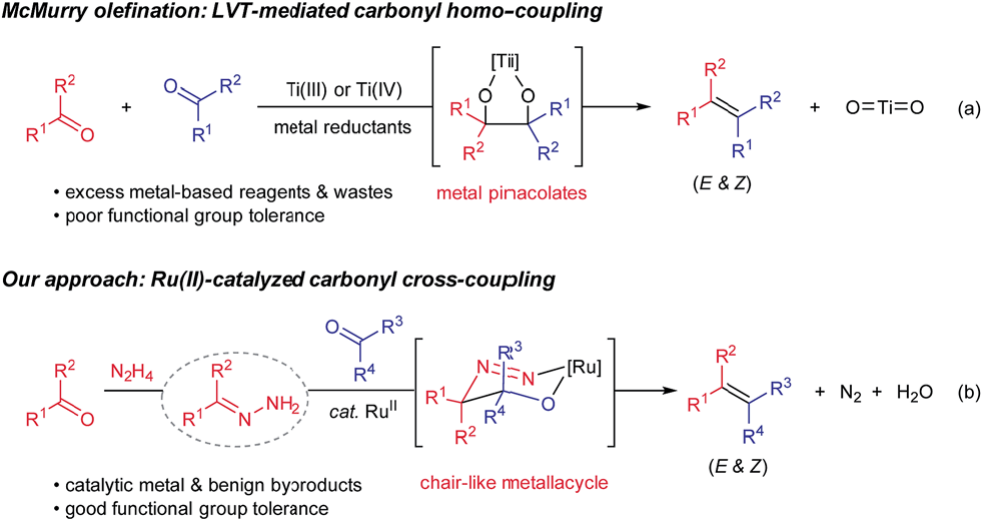
Olefination methods *via* reductive carbonyl coupling.

Very recently, we have disclosed a ruthenium-catalyzed deoxygenation reaction for the highly selective cleavage of aliphatic primary C–O bonds.^10^ Building on the similar ruthenium(ii) catalysis, we further demonstrated its robustness in catalyzing a series of new carbon–carbon bond forming processes through addition reactions to various carbonyl compounds, imines and activated alkenes.^11^–^13^ Variations of these precedents notwithstanding, one of their commonalities is to use aldehydes/ketones as alkyl carbanion equivalents *via* hydrazone formation. As a continuation of our interests in utilizing such carbanion equivalents for useful synthetic transformations, we describe herein the development of a ruthenium(ii)-catalyzed, hydrazine-mediated olefination reaction *via* carbonyl reductive cross-coupling (Scheme 1, eqn (b)). This catalytic method features good functional group tolerance and generates nitrogen and water as the only environmentally benign byproducts in stoichiometric quantities.^14^,^15^

Initially, propionaldehyde **1a** and benzophenone **2a** were used as carbonyl partners in our investigation. By varying the reaction conditions employed in carbonyl addition,^11^ we detected the corresponding olefin **3a** in 51% yield from the cross-coupling between preformed hydrazone of **1a** and **2a** after 12 h (Table 1, entry 1). In line with our basicity rationale, a variety of bases were prioritized in the optimization process. In fact, improved yields were consistently observed using stronger ionic bases such as hydroxides and alkoxides, among which KO^*t*^Bu gave the best result (Table 1, entries 1–5; Table S3, ESI†). In contrast, organic base (*e.g.* Et_3_N) and weaker inorganic base (*e.g.* K_2_CO_3_) were significantly inferior (Table 1, entries 6 and 7). Moreover, the amount of base matters significantly for this reaction. Specifically, lower loadings than substoichiometric quantity (*i.e.* 50 mol%) were associated with yield attenuation (Table S4, ESI†). As expected, the olefination reaction did not proceed in the absence of base (Table 1, entry 8). In addition to the basicity, both ruthenium(ii) pre-catalyst and bidentate phosphine ligand 1,2-bis(dimethylphosphino)ethane (dmpe) are essential for the elimination to occur. Likewise, no desired product was obtained without either one of them (Table 1, entries 9 and 10). Subsequent screenings on various ruthenium catalysts (Table S1, ESI†) and phosphine ligands (Table 1, entries 11–14; Table S2, ESI†)^16^ revealed that the combination of [Ru(*p*-cymene)Cl_2_]_2_ and dmpe provided the biggest catalytic turnover number, and thus the highest yield. Although other reaction parameters such as solvent, additive and temperature play minor roles in the current reaction, the following observations are worthy noting. For example, ether solvents (*e.g.* THF, 1,4-dioxane, 1,2-dimethoxyethane) favor the olefination over other types of solvents (Table S5, ESI†). Addition of cesium fluoride as an additive slightly increases the reaction yield (Table S6, ESI†).

**Table 1:** Optimization of reaction conditions^*a*^

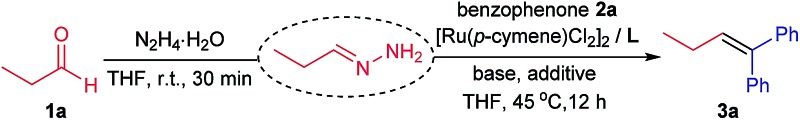			
Entry	**L**	Base	Yield^*b*^ (%)
1	dmpe	K_3_PO_4_	51
2	dmpe	KO^*t*^Bu	84
3	dmpe	NaO^*t*^Bu	80
4	dmpe	CsOH	82
5	dmpe	KOH	78
6	dmpe	Et_3_N	0
7	dmpe	K_2_CO_3_	4
8	dmpe	—	0
9	—	KO^*t*^Bu	0
10^*c*^	dmpe	KO^*t*^Bu	0
11	dppb	KO^*t*^Bu	11
12	dppp	KO^*t*^Bu	15
13	dppm	KO^*t*^Bu	4
14	P(*p*-Tolyl)_3_	KO^*t*^Bu	4

^*a*^ **1a** (0.28 mmol, 1.4 equiv.), N_2_H_4_·H_2_O (0.3 mmol, 1.5 equiv.), THF (0.14 mL), rt, 30 min; **2a** (0.20 mmol, 1.0 equiv.), [Ru(*p*-cymene)Cl_2_]_2_ (0.003 mmol, 1.5 mol%), ligand (0.006 mmol, 3.0 mol%), base (0.1 mmol, 50 mol%), additive: CsF (0.15 mmol, 75 mol%), 45 °C, 12 h, under N_2_.

^*b*^Yields were determined by crude ^1^H NMR using mesitylene as an internal standard.

^*c*^Without [Ru(*p*-cymene)Cl_2_]_2_.

With the standard reaction conditions in hand, we moved on to study the scope of electrophilic carbonyl partners (Table 2). In general, this chemistry covers a broad spectrum of benzophenone and its derivatives, regardless of their electronic nature. Consequently, the corresponding olefins (**3a–h**) were obtained in moderate to good yields. For unsymmetrical benzophenones, a mixture of stereoisomers (*E*/*Z* isomers) was produced (**3e–h**). Similarly, acetophenones bearing a wide range of aryl substituents were surveyed, with the formation of *E*/*Z* isomers (*E*-isomer predominant) in moderate to good yields (**3i–ip**). Notably, functional groups that are commonly incompatible with traditional carbonyl olefination approach such as unprotected alcohol, ester and amide groups, were well tolerated in this chemistry and thus amenable to further functionalization (**3ij**, **3in** and **3ip**). Intriguingly, aromatic ketones substituted by linear alkyl chains (2–5 carbons) did not hamper the reaction leading to the desired products in good yields (**3j–l**). Aromatic aldehyde and aliphatic ketones can also serve as coupling partners, including the relatively complex oxo-steroid compound. Asymmetric azines were observed as major products with cyclic aliphatic ketones (**3o**). However, the complex reaction mixture was resulted in the case of acyclic aliphatic ketone (**3n**), whereby the corresponding asymmetric azine was obtained as one of the major side products. Low yields were therefore seen in all these substrates at this stage.

**Table 2:** Scope of electrophilic carbonyl coupling partners^*a*^ ^,^^*b*^

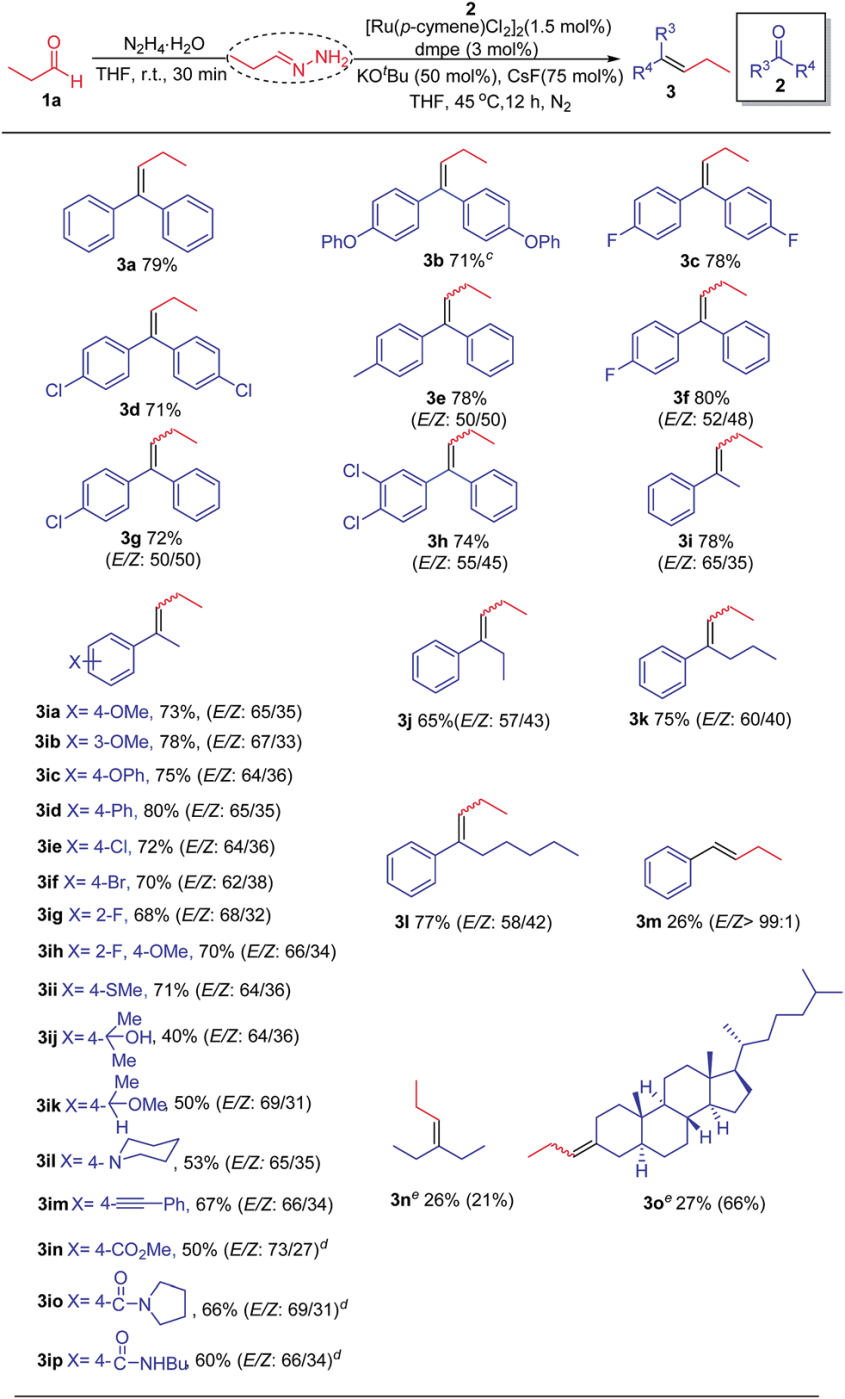

^*a*^ **1a** (0.28 mmol, 1.4 equiv.), N_2_H_4_·H_2_O (0.3 mmol, 1.5 equiv.), THF (0.14 mL), rt, 30 min; **2** (0.20 mmol, 1.0 equiv.), [Ru(*p*-cymene)Cl_2_]_2_ (0.003 mmol, 1.5 mol%), dmpe (0.006 mmol, 3.0 mol%), KO^*t*^Bu (0.1 mmol, 50 mol%), CsF (0.15 mmol, 75 mol%), 45 °C, 12 h, under N_2_.

^*b*^Isolated yields and the ratio of *E*/*Z* isomers were determined by crude ^1^H NMR analysis.

^*c*^80 °C.

^*d*^K_3_PO_4_ (0.1 mmol, 50 mol%), 12 h.

^*e*^Yields were determined by crude ^1^H NMR using mesitylene as an internal standard. Yields of asymmetric azines were given in the parentheses.

Subsequently, the scope of nucleophilic carbonyl coupling partners was tested (Table 3). Linear saturated aliphatic aldehydes, irrespective of carbon chain length, all delivered the corresponding olefins in moderate to good yields (**4a–f**). Although aromatic aldehydes were poorly coupled with aromatic ketones (*i.e.* The reductive coupling of benzaldehyde with benzophenone leading to product **4g**) due to competing alcohol formation, their couplings with other aromatic aldehydes typically proceeded well to yield various stilbenes with excellent stereoselectivity (**4h–k**). In the latter cases, using K_2_CO_3_ as the base at elevated temperature was necessary. Encouragingly, more sterically demanding aromatic ketones (*i.e.* acetophenone) worked as nucleophilic partner, providing **4l** in moderate yield with high stereoselectivity. Finally, the intramolecular olefination was evaluated with 3-(2-benzoylphenyl)propanal **1m** and 6-oxo-6-phenylhexanal **1n**. The corresponding cyclohexene derivatives **4m** and **4n** were obtained in 51% and 48% yields, respectively.

**Table 3:** Scope of nucleophilic carbonyl coupling partners^*a*^ ^,^^*b*^

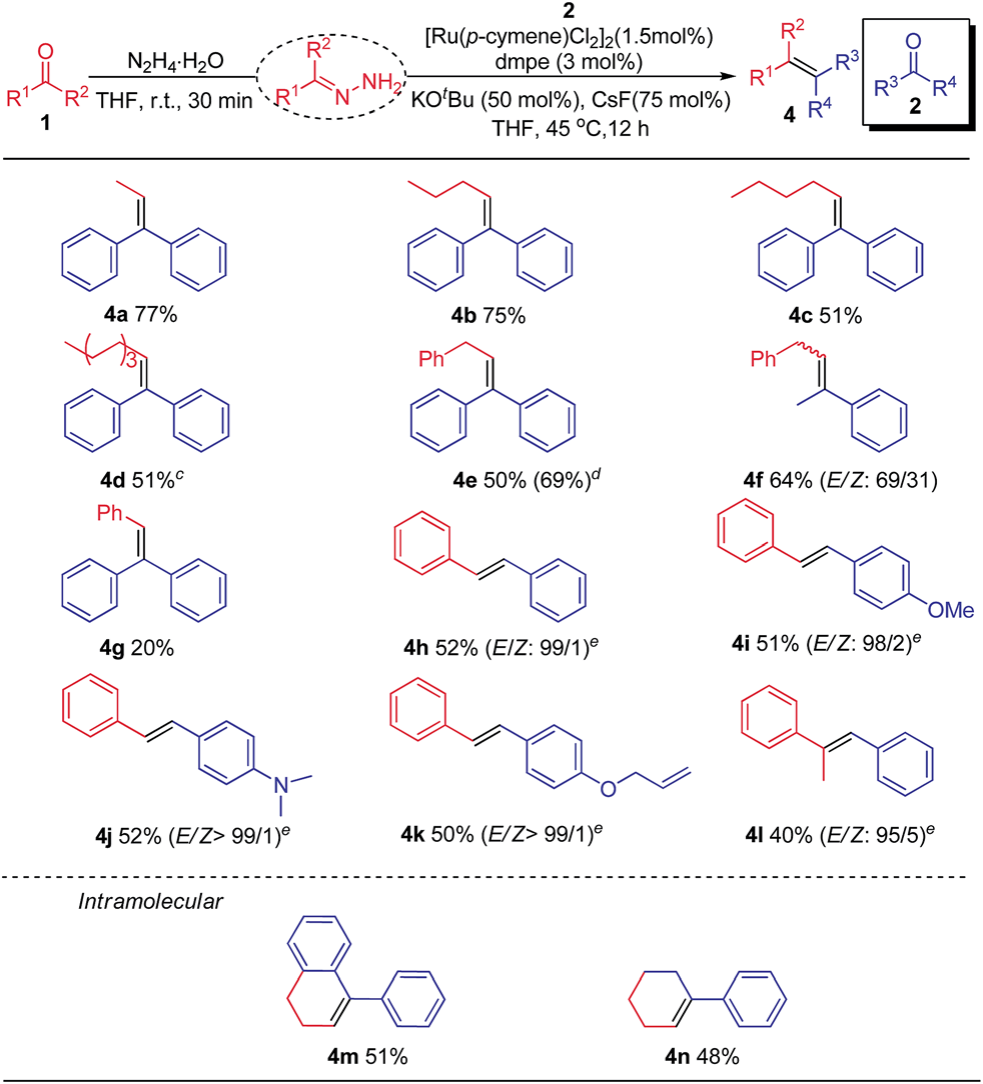

^*a*^ **1** (0.28 mmol, 1.4 equiv.), N_2_H_4_·H_2_O (0.3 mmol, 1.5 equiv.), THF (0.14 mL), rt, 30 min; **2** (0.20 mmol, 1.0 equiv.), [Ru(*p*-cymene)Cl_2_]_2_ (0.003 mmol, 1.5 mol%), dmpe (0.006 mmol, 3.0 mol%), KO^*t*^Bu (0.1 mmol, 50 mol%), CsF (0.15 mmol, 75 mol%), 45 °C, 12 h, under N_2_.

^*b*^Isolated yields and the ratio of *E*/*Z* isomers were determined by crude ^1^H NMR analysis.

^*c*^60 °C, 24 h.

^*d*^[(C_6_Me_6_)RuCl_2_]_2_ (0.003 mmol, 1.5 mol%) was used as catalyst.

^*e*^K_2_CO_3_ (0.1 mmol, 50 mol%), 120 °C, 24 h.

Mechanistically, two scenarios are possible to form olefins. One is metal-assisted decomposition of the corresponding asymmetric azine. The other is base-mediated elimination of the corresponding alcohol. In other words, both asymmetric azines and alcohols might have been generated prior to olefins. To exclude these possibility, two parallel control experiments were conducted with the presynthesized azine **6i** and 1,1-diphenylbutan-1-ol **5a** under standard conditions, respectively (Scheme 2). However, olefin products (**3i** and **3a**) were not detected in both cases. The above results strongly suggested that olefins were eliminated from a transient intermediate, rather than azines or alcohols, *via* a E1cB-type mechanism.

**Scheme 2:**
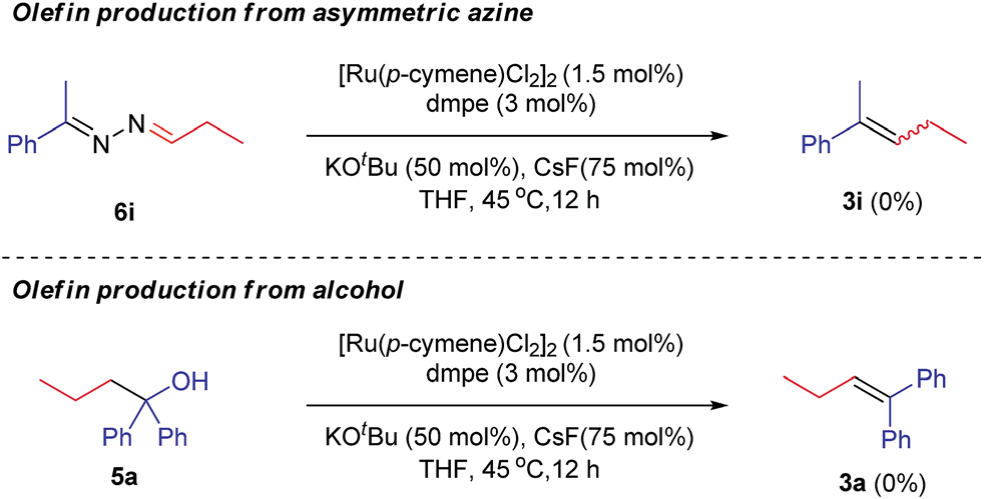
Control experiments for the olefin formation.

Based on all experimental data, the preliminary computational calculations done on the previous carbonyl addition chemistry^11^ and studies from others,^14^ a postulated reaction pathway was proposed as shown in Scheme 3. The bidentate phosphine coordinated complex **I** is initially generated by a ligand dissociation/association between [Ru(*p*-cymene)Cl_2_]_2_ and dmpe, followed by a ligand association with carbonyl-derived hydrazone **B** and carbonyl **C** in the presence of KO^*t*^Bu, giving rise to complex **II** and **III,** respectively. Formation of the coordinately intermediate **III** sets the stage for the intramolecular isomerization. This concerted process yields the key six-membered ring intermediate **IV** by forming a new carbon–carbon bond between **A** and **C**.^14^ Base-catalyzed decomposition of diimide intermediate **IV***via* a E1cB-type mechanism produces the desired olefins along with the formation of ruthenium hydroxide species **V**. To turnover the cycle, **V** then reacts with hydrazone **B** to release water and active species **II** (X = Cl). Alternatively, the chloride anionic ligand on **V** could have been replaced by **B**, leaving hydroxide on the active species **VI** (X = OH) and other ruthenium complexes (**III–V**).

**Scheme 3:**
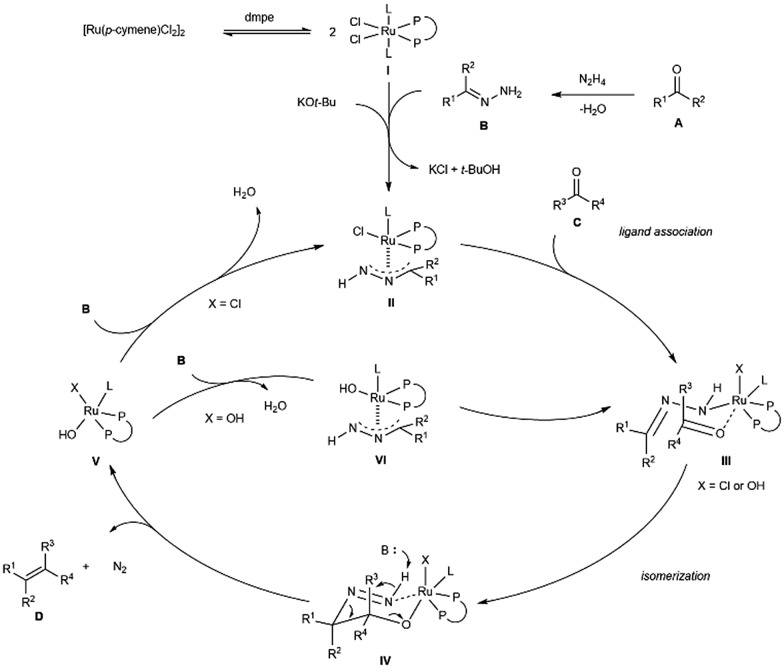
Possible reaction mechanism.

## Conclusions

In summary, we have developed a ruthenium(ii)-catalyzed, hydrazine-mediated olefination method *via* reductive carbonyl coupling reactions. This chemistry possesses a distinct mechanistic profile and highlights the use of naturally abundant carbonyl functionalities for efficient olefin synthesis. Other striking features include cross-coupling capability, mild reaction conditions, good functional group tolerance and stoichiometric benign byproducts. Taken together, our findings are expected to spur more interest in developing catalytic methods in this field. Further investigations on increasing the reaction scope, synthetic applications and mechanistic details are undergoing in our laboratory.

## Conflicts of interest

There are no conflicts to declare.

## Supplementary Material

Supplementary information
