# Supplementary material for: Ruthenium(ii)-catalyzed olefination via carbonyl reductive cross-coupling
Source: Chem Sci. 2017 Oct 9;8(12):8193–7. doi: 10.1039/c7sc04207h (PMC5855968; doi:10.1039/c7sc04207h)
Supplement: Supplementary file 1 [file SC-008-C7SC04207H-s001.pdf]

Electronic Supplementary Material (ESI) for Chemical Science.
This journal is © The Royal Society of Chemistry 2017

Supplementary Information

Ruthenium(II)-catalyzed olefination *via* carbonyl reductive cross-coupling

Wei Wei,[a,b†] Xi-Jie Dai,[a†] Haining Wang,[a] Chenchen Li,[a] Xiaobo Yang,[a] and Chao-Jun Li[a*]

[a] *Department of Chemistry, FQRNT Center for Green Chemistry and Catalysis, McGill University, 801 Sherbooke Street West, Montreal, Quebec H3A 0B8, Canada*

[b] *School of Chemistry and Chemical Engineering, Qufu Normal University, Qufu 273165, Shandong, China.*

[†] *These authors contributed equally to this work.*

Email: cj.li@mcgill.ca

TABLE OF CONTENTS

1. General information S2

2. Optimization of the reaction conditions S3

3. General reaction procedure S6

4. Mechanism investigation S7

5. Characterization data of products **3a−4s** S8

6. Copies of NMR spectra for **3a−4s** S25

7. Copies of crude ^{1}H NMR and HRMS for cross azines S93

1. General information

Chemicals: All catalysts, ligands, bases and additives that are commercially available (Aldrich), were used without further purification: hydrazine hydrate (reagent grade, 64-65% wt), mesitylene, anhydrous sodium sulfate. All liquid carbonyls were distilled and solid ones were recrystallized prior to use.

Solvents: Tetrahydrofuran (THF), dimethyl sulfoxide (DMSO) and toluene were taken directly from the *Pure Solvent MD-7* purification system (Innovative Technology). Reaction solvent *tert*-butanol (*t*-BuOH) (ACS grade) was distilled over CaH_2 prior to use. Solvents for filtration, transfers, chromatography and recrystallization were hexane (Fisher, ACS grade), pentane (ACS grade) and dichloromethane (CH_2Cl_2)(ACS grade, amylene stabilized).

NMR Spectroscopy: Nuclear magnetic resonance (^{1}H and ^{13}C NMR) spectra were recorded on a Bruker AV500 equipped with a 60-position SampleXpress sample changer (^{1}H, 500 MHz; ^{13}C, 125 MHz), or Bruker AV400 spectrometer (^{1}H, 400 MHz; ^{13}C, 100 MHz). Chemical shifts for both ^{1}H NMR and ^{13}C NMR spectra are expressed in parts per million (ppm) units downfield from TMS, with the solvent residue peak as the reference ($CDCl_3$: δ 7.26 ppm in ^{1}H NMR; δ 77.0 ppm in ^{13}C NMR). Data are reported as following: chemical shift, multiplicity (s = singlet, d = doublet, dd = doublet of doublets, t = triplet, td = triplet of doublets, dt = doublet of triplets, q = quartet, quin = quintet, sep = septet, m = multiplet, br = broad singlet), coupling constants *J* (Hz), and integration.

Mass Spectrometry: Mass spectrometry (MS) was performed by the McGill Chemistry Department Mass Spectrometry Facility. High Resolution Mass spectra were recorded using electrospray ionization (ESI+) and/or atmospheric pressure chemical ionization APCI (+/-), performed either on an "Exactive Plus Orbitrap" Thermo Scientific high resolution accurate mass (HR/AM) FT mass spectrometer, or a Bruker Daltonics Maxis Impact quadrupole-time of flight (QTOF) mass spectrometer.

Reaction Setup: All reactions were carried out in flamed-dried V-shaped microwave reaction vials, covered by aluminum seals with PTFE-faced silicone septa, under an atmosphere of nitrogen unless otherwise stated. All reported reaction temperatures correspond to oil bath temperatures. All air and moisture sensitive catalysts, ligands, and reagents were stored and charged in MBRAUN UNIlab Pro Glove Box Workstation.

Purifications: All work-up and purification procedures were carried out with reagent grade solvents. Analytical thin-layer chromatography (TLC) was performed using E. Merck silica gel 60 F254 pre-coated plates (0.25 mm). Flash column chromatography was performed with E. Merck silica gel P60 (40-63 μm particle size, 230–00 mesh) (SiO_2). Unless otherwise specified, “SiO_2” refers to P60 grade silica gel. Automated flash column chromatography was performed on Biotage Isolera™Spektra Systems with ACI™.

2. Optimization of the reaction conditions

Table S1. The effect of catalysts[a]

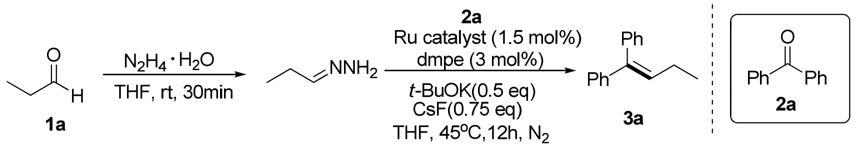

Entry	Catalyst	Yield(%)[b]	Entry	Catalyst	Yield(%)[b]
1	---	0	8	Chlorocyclopentadienyl-bis(triphenylphosphine)-ruthenium(II)	22
2	$[(C_5H_5)Ru(CH_3CN)_3]PF_6$	2	9	dichloro(pentamethylcyclopentadienyl)ruthenium(III) polymer	2
3	$[(C_6H_5)_3P]_3Ru(CO)(Cl)H$	3	10	$Ru_3(CO)_{12}$	0
4	[Ru(*p*-cymene)Cl_2]$_2$	84	11	bis(cyclopentadienyl)-ruthenium(II)	0
5	$RuCl_2(PPh_3)_3$	74	12	$Ru_2(C_6H_6)_2Cl_4$	68
6	$[Ru(COD)Cl_2]_n$	78	13	$[Ru(CO)_3Cl_2]_2$	16
7	$RuCl_3$	2			

[a] **1a** (0.28 mmol, 1.4 equiv.), $N_2H_4 \cdot H_2O$ (0.3 mmol, 1.5 equiv.), THF (0.14 mL), r.t., 30 min; **2a** (0.20 mmol, 1.0 equiv.), Ru catalyst (0.003 mmol, 1.5 mol%), dmpe (0.006 mmol, 3.0 mol%), *t*-BuOK (0.1 mmol, 50 mol%), CsF (0.15 mmol, 75 mol%), 45ºC, 12 h, under N_2. [b] Yields were determined by crude ^{1}H NMR using mesitylene as an internal standard.

Table S2. The effect of ligands[a]

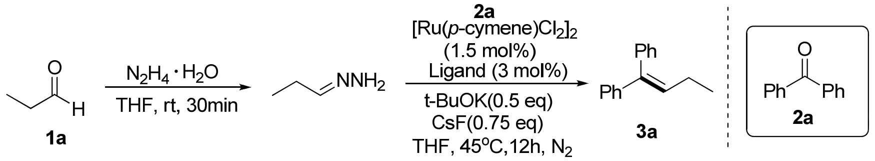

Entry	Ligand	Yield(%)[b]	Entry	Ligand	Yield(%)[b]
1	---	0	8	P(*p*-tolyl)$_3$	4
2	dppe	10	9	$Ph_4P^+BF_4$	2
3	dppb	11	10	trimesitylphosphine	0
4	dppp	15	11	1,4-bis(dicyclohexyl-phosphino)butane	10
5	dppm	4	12	dicyclohexylphenyl-phosphine	8
6	dmpe	84	13	DavePhos	2
7	*trans*-PPh_2CH=$CHPPh_2$	3	14	BrettPhos	0

[a] **1a** (0.28 mmol, 1.4 equiv.), $N_2H_4 \cdot H_2O$ (0.3 mmol, 1.5 equiv.), THF (0.14 mL), r.t., 30 min; **2a** (0.20 mmol, 1.0 equiv.), [Ru(*p*-cymene)Cl_2]$_2$ (0.003 mmol, 1.5 mol%), ligand (0.006 mmol, 3.0 mol%), *t*-BuOK (0.1 mmol, 50 mol%), CsF (0.15 mmol, 75 mol%), 45ºC, 12 h, under N_2. [b] Yields were determined by crude ^{1}H NMR using mesitylene as an internal standard.

Table S3. The effect of bases[a]

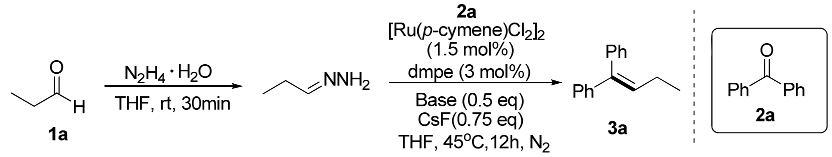

Entry	Base	Yield(%)[b]	Entry	Base	Yield(%)[b]
1	---	0	7	NaOH	78
2	K_3PO_4	51	8	*t*-BuOK	84
3	K_2CO_3	4	9	*t*-BuONa	80
4	Na_2CO_3	0	10	CsOH	82
5	Cs_2CO_3	21	11	DBU	0
6	KOH	78	12	Et_3N	0

[a] **1a** (0.28 mmol, 1.4 equiv.), $N_2H_4·H_2O$ (0.3 mmol, 1.5 equiv.), THF (0.14 mL), r.t., 30 min; **2a** (0.20 mmol, 1.0 equiv.), $[Ru(p\text{-cymene})Cl_2]_2$ (0.003 mmol, 1.5 mol%), dmpe (0.006 mmol, 3.0 mol%), base (0.1 mmol, 50 mol%), additive: CsF (0.15 mmol, 75 mol%), 45°C, 12 h, under N_2. [b] Yields were determined by crude ^{1}H NMR using mesitylene as an internal standard.

Table S4. The effect of loading of base[a]

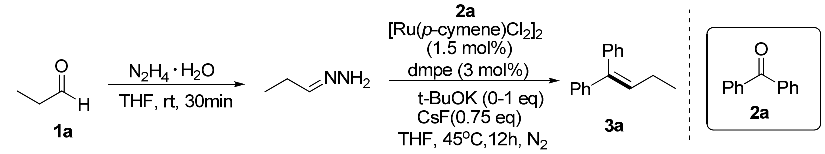

Entry	*t*-BuOK(equiv)	Yield(%)[b]	Entry	*t*-BuOK(equiv)	Yield(%)[b]
1	0	0	4	0.5	84
2	0.125	41	5	0.75	84
3	0.25	74	6	1	58

[a] **1a** (0.28 mmol, 1.4 equiv.), $N_2H_4·H_2O$ (0.3 mmol, 1.5 equiv.), THF (0.14 mL), r.t., 30 min; **2a** (0.20 mmol, 1.0 equiv.), $[Ru(p\text{-cymene})Cl_2]_2$ (0.003 mmol, 1.5 mol%), dmpe (0.006 mmol, 3.0 mol%), *t*-BuOK (0-1eq), additive: CsF (0.15 mmol, 75 mol%), 45°C, 12 h, under N_2. [b] Yields were determined by crude ^{1}H NMR using mesitylene as an internal standard.

Table S5. The effect of solvents[a]

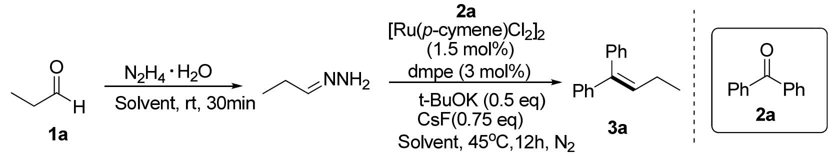

Entry	Solvent	Yield(%)[b]	Entry	Solvent	Yield(%)[b]
1	THF	84	6	DMSO	0
2	1,4-dioxane	67	7	CH_3CN	0
3	DME	76	8	DCE	0
4	toluene	7	9	DMF	0
5	*t*-BuOH	12	10	EtOH	0

[a] **1a** (0.28 mmol, 1.4 equiv.), $N_2H_4{\cdot}H_2O$ (0.3 mmol, 1.5 equiv.), solvent (0.14 mL), r.t., 30 min; **2a** (0.20 mmol, 1.0 equiv.), $[Ru(p\text{-cymene})Cl_2]_2$ (0.003 mmol, 1.5 mol%), dmpe (0.006 mmol, 3.0 mol%), *t*-BuOK (0.1 mmol, 50 mol%), additive: CsF (0.15 mmol, 75 mol%), 45°C, 12 h, under N_2. [b] Yields were determined by crude ^1H NMR using mesitylene as an internal standard.

Table S6. The effect of loading of CsF[a]

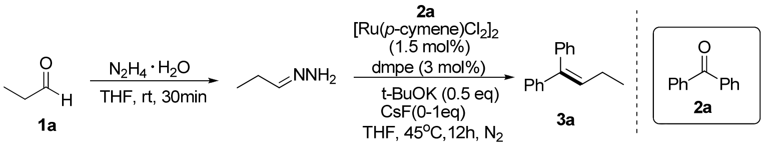

Entry	CsF(equiv)	Yield(%)[b]	Entry	CsF(equiv)	Yield(%)[b]
1	0	52	4	0.75	84
2	0.25	60	5	1	76
3	0.5	69			

[a] **1a** (0.28 mmol, 1.4 equiv.), $N_2H_4{\cdot}H_2O$ (0.3 mmol, 1.5 equiv.), THF (0.14 mL), r.t., 30 min; **2a** (0.20 mmol, 1.0 equiv.), $[Ru(p\text{-cymene})Cl_2]_2$ (0.003 mmol, 1.5 mol%), dmpe (0.006 mmol, 3.0 mol%), KO^{t}Bu (0.1 mmol, 50 mol%), additive: CsF (0-1 equiv.), 45°C, 12 h, under N_2. [b] Yields were determined by crude ^1H NMR using mesitylene as an internal standard.

Table S7. The effect of temperature[a]

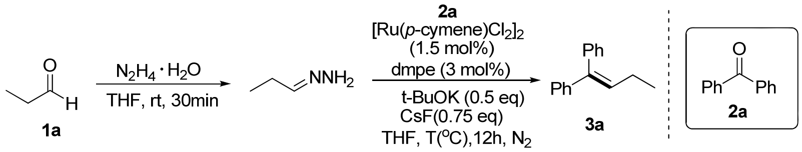

Entry	T(°C)	Yield(%)[b]	Entry	T(°C)	Yield(%)[b]
1	25	48	4	55	70
2	35	80	5	65	77
3	45	84			

[a] **1a** (0.28 mmol, 1.4 equiv.), $N_2H_4{\cdot}H_2O$ (0.3 mmol, 1.5 equiv.), THF (0.14 mL), r.t., 30 min; **2a** (0.20 mmol, 1.0 equiv.), $[Ru(p\text{-cymene})Cl_2]_2$ (0.003 mmol, 1.5 mol%), dmpe (0.006 mmol, 3.0 mol%), *t*-BuOK (0.1 mmol, 50 mol%), additive: CsF (0.15 mmol, 75 mol%), 25-65°C, 12 h, under N_2. [b] Yields were determined by crude ^1H NMR using mesitylene as an internal standard.

Table S8. The scope of other substrates [a,b]

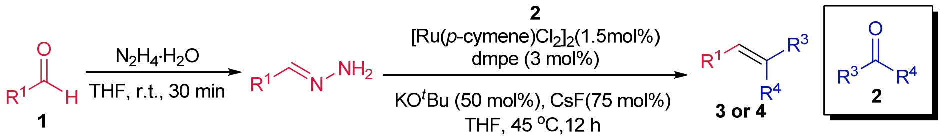

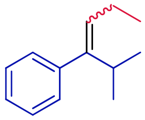

3p 30% (*E*/*Z*: 57/43)

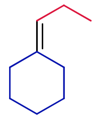

3q 25% (41%)[c]

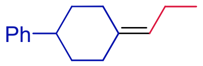

3r 25% (56%)[c]

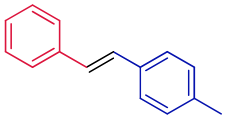

4o 50% (*E*/*Z*: 98/2)[d]

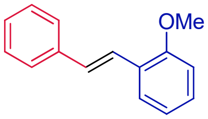

4p 49% (*E*/*Z*: 98/2)[d]

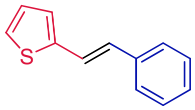

4q38% (*E*/*Z*> 99/1)[d]

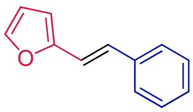

4r 36% (*E*/*Z*> 99/1)[d]

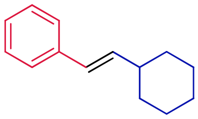

4s 23%(*E*/*Z*> 99/1)[e]

[a] **1** (0.28 mmol, 1.4 equiv.), $N_2H_4{\cdot}H_2O$ (0.3 mmol, 1.5 equiv.), THF (0.14 mL), r.t., 30 min; **2** (0.20 mmol, 1.0 equiv.), [Ru(*p*-cymene)Cl_2]$_2$ (0.003 mmol, 1.5 mol%), dmpe (0.006 mmol, 3.0 mol%), *t*-BuOK (0.1 mmol, 50 mol%); additive: CsF (0.15 mmol, 75 mol%), 45°C, 12 h, under N_2. [b] Isolated yields and the ratio of *E/Z* isomers were determined by crude ^{1}H NMR analysis. [c] Yields were determined by crude ^{1}H NMR using mesitylene as an internal standard. [d] K_2CO_3 (0.1 mmol, 50 mol%), 120 °C, 24 h. [e] K_3PO_4 (0.1 mmol, 50 mol%), 12h.

3. General reaction procedure

3.1 The general procedure for intermolecular olefination via carbonyl cross-coupling

A flamed-dried V-shape microwave reaction vial (10 cm^3) equipped with a magnetic stir bar was charged with [Ru(*p*-cymene)Cl_2]$_2$ (1.8 mg, 0.003 mmol, 1.5 mol%) and KO^{t}Bu (11.2 mg, 0.1 mmol, 50 mol%). The reaction vial was then transferred into the glovebox and charged with dmpe (1.0 µL, 0.006 mmol, 3 mol%) and CsF (22.8 mg, 0.15 mmol, 75 mol%), before being sealed with a rubber septum. The reaction vial was then moved out of the glovebox and sequentially charged with ketones or aldehydes (0.2 mmol, 1.0 equiv) and the **hydrazone solution** (0.28 mmol). The reaction mixture was then heated to 45 °C in an oil bath. Upon stirring for 12 h,

the reaction mixture was filtered through a plug of silica gel with CH_2Cl_2 (10 mL) as eluent, concentrated and purified by flash chromatography (hexane as eluent) to give the corresponding alkenes.

Hydrazone solution: A mixture of carbonyls (0.28 mmol, 1.4 equiv) and hydrazine monohydrate (17.5 μL, 0.3 mmol, 64-65 wt%, 1.5 equiv) in THF (0.14 mL) solution was stirred at room temperature for 30 min. Prior to the injection of this hydrazone solution into reaction mixture, a small amount of anhydrous Na_2SO_4 was added.

3.2 The general procedure for intramolecular olefination via carbonyl cross-coupling

A flamed-dried V-shape microwave reaction vial (10 cm^3) equipped with a magnetic stir bar was charged with $[Ru(p\text{-cymene})Cl_2]_2$ (1.8 mg, 0.003 mmol, 1.5 mol%) and KO^tBu (11.2 mg, 0.1 mmol, 50 mol%). The reaction vial was then transferred into the glovebox and charged with dmpe (1.0 μL, 0.006 mmol, 3 mol%) and CsF (22.8 mg, 0.15 mmol, 75 mol%), before being sealed with a rubber septum. The reaction vial was then moved out of the glovebox and charged with the **hydrazone solution** (0.2 mmol). The reaction mixture was then heated to 45 ^oC in an oil bath. Upon stirring for 12 h, the reaction mixture was filtered through a plug of silica gel with CH_2Cl_2 (10 mL) as eluent, concentrated and purified by flash chromatography (hexane as eluent) to give the corresponding cyclic alkenes.

Hydrazone solution: A mixture of carbonyls (0.2 mmol, 1 equiv) and hydrazine monohydrate (15 μL, 0.26 mmol, 64-65 wt%, 1.3 equiv) in THF (0.15 mL) solution was stirred at room temperature for 30 min. Prior to the injection of this hydrazone solution into reaction mixture, a small amount of anhydrous Na_2SO_4 was added.

4. Mechanism investigation

4.1 Olefin production from asymmetric azine

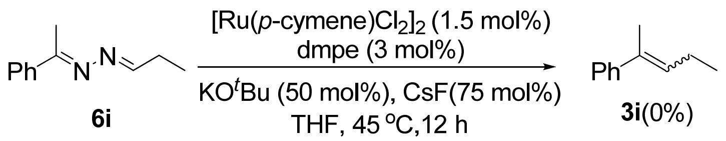

A flamed-dried V-shape microwave reaction vial (10 cm^3) equipped with a magnetic stir bar was charged with [Ru(*p*-cymene)Cl_2]$_2$ (1.8 mg, 0.003 mmol, 1.5 mol%) and KO^{t}Bu (11.2 mg, 0.1 mmol, 50 mol%). The reaction vial was then transferred into the glovebox and charged with dmpe (1.0 μL, 0.006 mmol, 3 mol%) and CsF (22.8 mg, 0.15 mmol, 75 mol%), before being sealed with a rubber septum. The reaction vial was then moved out of the glovebox and sequentially charged with presynthesized azine **6i** (0.2 mmol, 1.0 equiv) and THF (140 μL). The reaction mixture was then heated to 45 °C in an oil bath. Upon stirring for 12 h, the reaction mixture was analyzed by ^{1}H NMR using mesitylene as an internal standard. No desired alkene **3i** was detected.

4.2 Olefin production from alcohol

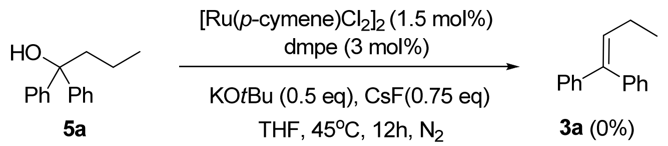

A flamed-dried V-shape microwave reaction vial (10 cm^3) equipped with a magnetic stir bar was charged with [Ru(*p*-cymene)Cl_2]$_2$ (1.8 mg, 0.003 mmol, 1.5 mol%) and KO^{t}Bu (11.2 mg, 0.1 mmol, 50 mol%). The reaction vial was then transferred into the glovebox and charged with dmpe (1.0 μL, 0.006 mmol, 3 mol%) and CsF (22.8 mg, 0.15 mmol, 75 mol%), before being sealed with a rubber septum. The reaction vial was then moved out of the glovebox and sequentially charged with 1,1-diphenylbutan-1-ol **5a** (0.2 mmol, 1.0 equiv) and THF (140 μL). The reaction mixture was then heated to 45 °C in an oil bath. Upon stirring for 12 h, the reaction mixture was analyzed by ^{1}H NMR using mesitylene as an internal standard. No desired alkene **3a** was detected.

5. Characterization data of products 3a-4s

but-1-ene-1,1-diyldibenzene 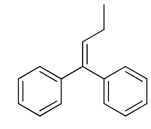 **(3a)**

Colorless oil; The spectroscopic data correspond to those previously reported in the literature.[1] ^{1}H NMR ($CDCl_3$, 500 MHz, ppm): δ 7.43-7.40 (m, 2H), 7.34 (tt, J_1 = 1.4

Hz, J_2 = 7.5 Hz, 1H), 7.32-7.22 (m, 7H), 6.12 (t, J = 7.5 Hz, 1H), 2.17 (quint, J = 7.5 Hz, 2H), 1.09 (t, J = 7.5 Hz, 3H); ^{13}C NMR ($CDCl_3$, 125 MHz, ppm): δ 142.9, 141.0, 140.3, 131.8, 129.9, 128.1, 128.0, 127.2, 126.9, 126.8, 23.2, 14.6; MS (EI) m/z: 208.1, 193.1, 178.1, 165.1, 129.1.

4,4'-(but-1-ene-1,1-diyl)bis(phenoxybenzene) 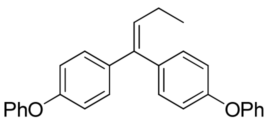

(3b)

Colorless oil; ^{1}H NMR ($CDCl_3$, 500 MHz, ppm): δ 7.41-7.34 (m, 4H), 7.23 (d, J = 8.7 Hz, 2H), 7.18-7.09 (m, 6H), 7.06-7.03 (m, 4H), 6.94 (d, J = 8.7 Hz, 2H), 6.04 (t, J = 7.5 Hz, 1H), 2.18 (quint, J = 7.5 Hz, 2H), 1.08 (t, J = 7.5 Hz, 3H); ^{13}C NMR ($CDCl_3$, 125 MHz, ppm): δ 157.3, 157.1, 156.3, 156.2, 139.6, 138.1, 135.1, 131.2, 131.2, 129.8, 129.7, 128.5, 123.4, 123.2, 119.1, 118.8, 118.5, 118.3, 23.3, 14.6; HRMS calc. for $C_{28}H_{25}O_2$ $(M+H)^+$, 393.18491; found, 393.18478.

4,4'-(but-1-ene-1,1-diyl)bis(fluorobenzene) 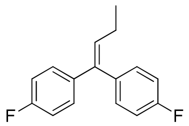

(3c)

Colorless oil; ^{1}H NMR ($CDCl_3$, 500 MHz, ppm): δ 7.20-7.14 (m, 4H), 7.09 (tt, J_1 = 2.7 Hz, J_2 = 8.8 Hz, 2H), 6.98 (tt, J_1 = 3.1 Hz, J_2 = 8.7 Hz, 2H), 6.03 (t, J = 7.5 Hz, 1H), 2.13 (quint, J = 7.5 Hz, 2H), 1.07 (t, J = 7.5 Hz, 3H); ^{13}C NMR ($CDCl_3$, 125 MHz, ppm): δ 162.9 (d, J = 15.2 Hz), 160.9 (d, J = 15.0 Hz), 139.0, 138.8 (d, J = 3.2 Hz), 135.9 (d, J = 3.3 Hz), 131.9, 131.4 (d, J = 7.9 Hz), 128.7 (d, J = 7.8 Hz), 115.1 (d, J = 21.2 Hz), 114.9 (d, J = 21.2 Hz), 23.2, 14.5; HRMS calc. for $C_{16}H_{15}F_2$ $(M+H)^+$, 245.11363; found, 245.11323.

4,4'-(but-1-ene-1,1-diyl)bis(chlorobenzene) 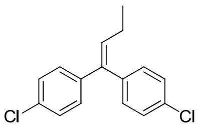

(3d)

Colorless oil; ^{1}H NMR ($CDCl_3$, 500 MHz, ppm): δ 7.37 (d, J = 8.4 Hz, 2H), 7.25 (d, J = 8.6 Hz, 2H), 7.14 (d, J = 8.6 Hz, 2H), 7.11 (d, J = 8.4 Hz, 2H), 6.08 (t, J = 7.5 Hz, 1H), 2.13 (quint, J = 7.5 Hz, 2H), 1.06 (t, J = 7.5 Hz, 3H); ^{13}C NMR ($CDCl_3$, 125 MHz, ppm): δ 140.9, 138.9, 138.2, 133.0, 132.8, 132.7, 131.2, 128.5, 128.4, 128.3, 23.2, 14.4; HRMS calc. for $C_{16}H_{15}Cl_2$ $(M+H)^+$, 277.05453; found, 277.05431.

1-methyl-4-(1-phenylbut-1-enyl)benzene 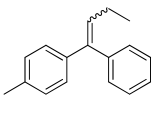 **(*E/Z*) (3e)**

Colorless oil; ^{1}H NMR ($CDCl_3$, 500 MHz, ppm): δ 7.41-7.38 (m, 2H), 7.34-7.20 (m, 10H), 7.16-7.14 (m, 2H), 7.11-7.09 (m, 4H), 6.09-6.06 (m, 2H), 2.42 (s, 3H), 2.36 (s, 3H), 2.20-2.11 (m, 4H), 1.08-1.05 (m, 6H); ^{13}C NMR ($CDCl_3$, 125 MHz, ppm): δ 143.1, 140.9, 140.8, 140.5, 140.1, 137.3, 136.5, 136.4, 131.6, 130.9, 129.9, 129.8, 128.8, 128.8, 128.1, 128.0, 127.3, 127.1, 126.8, 126.7, 23.3, 23.2, 21.3, 21.1, 14.6, 14.6; HRMS calc. for $C_{17}H_{19}$ $(M+H)^+$, 223.14813; found, 223.14783.

1-fluoro-4-(1-phenylbut-1-enyl)benzene 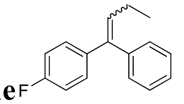 **(*E/Z*)(3f)**

Colorless oil; ^{1}H NMR ($CDCl_3$, 500 MHz, ppm): δ 7.41-7.38 (m, 1.83H), 7.35-7.27 (m, 3.49H), 7.26-7.16 (m, 8.65H), 7.08 (tt, J_1 = 2.2 Hz, J_2 = 6.6 Hz, 2H), 6.97 (tt, J_1 = 2.2 Hz, J_2 = 6.6 Hz, 1.84H), 6.10 (t, J = 7.5 Hz, 1H), 6.03 (t, J = 7.5 Hz, 0.92H), 2.17-2.11 (m, 3.88H), 1.08-1.05 (m, 5.76H); ^{13}C NMR ($CDCl_3$, 125 MHz, ppm): δ 161.9 (d, J = 244.2 Hz), 161.8 (d, J = 244.2 Hz), 142.7, 140.1, 140.0, 140.0, 139.0 (d, J = 3.2 Hz), 136.1 (d, J = 3.4 Hz), 132.1, 131.6, 131.6, 131.5 (d, J = 7.8 Hz), 129.8, 128.7 (d, J = 7.8 Hz), 128.2 (d, J = 10.3 Hz), 127.2, 126.9 (d, J = 10.6 Hz), 115.0 (d, J = 21.1 Hz), 114.8 (d, J = 21.1 Hz), 23.2, 14.5, 14.5; HRMS calc. for $C_{16}H_{16}F$ $(M+H)^+$, 227.12306; found, 227.12267.

1-chloro-4-(1-phenylbut-1-enyl)benzene 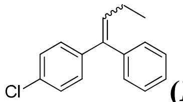 **(*E/Z*)(3g)**

Colorless oil; ^{1}H NMR ($CDCl_3$, 500 MHz, ppm): δ 7.41-7.33 (m, 5H), 7.29-7.21 (m, 7H), 7.19-7.13 (m, 6H), 6.10 (t, J = 7.5 Hz, 1H), 6.08 (t, J = 7.5 Hz, 1H), 2.17-2.10 (m, 4H), 1.08-1.04 (m, 6H); ^{13}C NMR ($CDCl_3$, 125 MHz, ppm): δ 142.4, 141.3, 140.0, 139.9, 139.8, 138.7, 132.8, 132.6, 132.3, 132.2, 131.3, 129.8, 128.5, 128.4, 128.3, 128.2, 128.2, 127.2, 127.1, 127.0, 23.2, 23.2, 14.5; HRMS calc. for $C_{16}H_{16}Cl$ $(M+H)^+$, 243.09350; found, 243.09303.

1,2-dichloro-4-(1-phenylbut-1-enyl)benzene 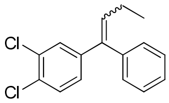 **(*E/Z*)(3h)**

Colorless oil; ^{1}H NMR ($CDCl_3$, 500 MHz, ppm): δ 7.46 (d, J = 8.2 Hz, 0.56H), 7.41 (d, J = 7.0 Hz, 0.9H), 7.37-7.26 (m, 3.68H), 7.22-7.20 (m, 1.12H), 7.17-7.16 (m, 0.9H), 7.05(dd, J_1 = 2.0 Hz, J_2 = 8.2 Hz, 1H), 6.13-6.08 (m, 1H), 2.14 (quint, J = 7.5 Hz, 2H), 1.09-1.05 (m, 3H); ^{13}C NMR ($CDCl_3$, 125 MHz, ppm): δ 143.0, 141.8, 140.3, 139.1, 138.9, 133.4, 133.0, 132.3, 132.2, 131.7, 131.0, 130.6, 130.2, 129.9, 129.8, 129.4, 129.0, 128.4, 128.3, 127.3, 127.2, 127.2, 126.6, 23.3, 23.2, 14.4, 14.4; HRMS calc. for $C_{16}H_{15}Cl_2$ $(M+H)^+$, 277.05453; found, 277.05406.

(*E*)-pent-2-en-2-ylbenzene 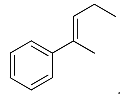 ***E*-(3i)**

Colorless oil; The spectroscopical data correspond to those previously reported in the literature.[1a] ^{1}H NMR ($CDCl_3$, 500 MHz, ppm): δ 7.44-7.42 (m, 2H), 7.35 (t, J = 7.4 Hz, 2H), 7.26 (tt, J_1 = 1.3 Hz, J_2 = 7.3 Hz, 1H), 5.83 (tq, J_1 = 1.3 Hz, J_2 = 7.5 Hz, 1H), 2.26 (quint, J = 7.5 Hz, 2H), 2.08 (d, J = 1.2 Hz, 3H), 1.11 (t, J = 7.5 Hz, 3H); ^{13}C NMR ($CDCl_3$, 125 MHz, ppm): δ 144.0, 134.1, 130.3, 128.2, 126.4, 125.6, 22.1, 15.6, 14.1; MS (EI) m/z: 146.1, 131.1, 115.0, 91.1.

(*Z*)-pent-2-en-2-ylbenzene 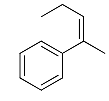 ***Z*-(3i)**

Colorless oil; ^{1}H NMR ($CDCl_3$, 500 MHz, ppm): δ 7.35 (t, J = 7.4 Hz, 2H), 7.25 (tt, J_1

= 1.3 Hz, J_2 = 7.5 Hz, 1H), 7.22-7.20 (m, 2H), 5.48 (td, J_1 = 1.3 Hz, J_2 = 7.4 Hz, 1H), 2.05 (d, J = 1.7 Hz, 3H), 2.01 (quintd, J_1 = 1.1 Hz, J_2 = 7.4 Hz, 2H), 0.96 (t, J = 7.4 Hz, 3H); ^{13}C NMR ($CDCl_3$, 125 MHz, ppm): δ 142.2, 135.4, 129.5, 128.0, 127.9, 126.4, 25.5, 22.4, 14.7; HRMS calc. for $C_{11}H_{15}$ $(M+H)^+$, 147.11683; found, 147.11686.

(*E*)-1-methoxy-4-(pent-2-en-2-yl)benzene 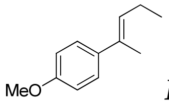 ***E*-(3ia)**

Colorless oil; ^{1}H NMR ($CDCl_3$, 500 MHz, ppm): δ 7.34 (dt, J_1 = 3.1 Hz, J_2 = 8.9 Hz, 2H), 6.87 (dt, J_1 = 3.1 Hz, J_2 = 8.9 Hz, 2H), 5.72 (td, J_1 = 1.3 Hz, J_2 = 7.5 Hz, 1H), 3.83 (s, 3H), 2.22 (quint, J = 7.5 Hz, 2H), 2.03 (d, J = 1.0 Hz, 3H), 1.08 (t, J = 7.5 Hz, 3H); ^{13}C NMR ($CDCl_3$, 125 MHz, ppm): δ 158.3, 136.6, 133.3, 128.7, 126.6, 113.5, 55.3, 22.0, 15.7, 14.2; HRMS calc. for $C_{12}H_{17}O$ $(M+H)^+$, 177.12739; found, 177.12710.

(*Z*)-1-methoxy-4-(pent-2-en-2-yl)benzene 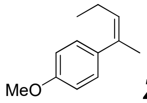 ***Z*-(3ia)**

Colorless oil; ^{1}H NMR ($CDCl_3$, 500 MHz, ppm): δ 7.15 (dt, J_1 = 2.9 Hz, J_2 = 8.8 Hz, 2H), 6.89 (dt, J_1 = 2.9 Hz, J_2 = 8.8 Hz, 2H), 5.44 (tq, J_1 = 1.4 Hz, J_2 = 7.5 Hz, 1H), 3.84 (s, 3H), 2.05-1.99 (m, 5H), 0.96 (t, J = 7.5 Hz, 3H); ^{13}C NMR ($CDCl_3$, 125 MHz, ppm): δ 158.1, 134.8, 134.5, 129.2, 129.0, 113.4, 55.2, 25.6, 22.5, 14.8; HRMS calc. for $C_{12}H_{17}O$ $(M+H)^+$, 177.12739; found, 177.12712.

(*E*)-1-methoxy-3-(pent-2-en-2-yl)benzene 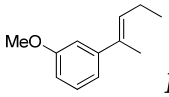 ***E*-(3ib)**

Colorless oil; ^{1}H NMR ($CDCl_3$, 500 MHz, ppm): δ 7.25 (t, J = 8.0 Hz, 1H), 7.03-7.00 (m, 1H), 6.95 (t, J = 2.4 Hz, 1H), 6.81-6.79 (m, 1H), 5.72 (td, J_1 = 1.3 Hz, J_2 = 7.1 Hz, 1H), 3.85 (s, 3H), 2.24 (quint, J = 7.5 Hz, 2H), 2.05 (d, J = 1.1 Hz, 3H), 1.09 (t, J = 7.5 Hz, 3H); ^{13}C NMR ($CDCl_3$, 125 MHz, ppm): δ 159.5, 145.6, 133.9, 130.5, 129.0, 118.2, 111.7, 111.6, 55.2, 22.0, 15.7, 14.1; HRMS calc. for $C_{12}H_{17}O$ $(M+H)^+$, 177.12739; found, 177.12717.

(*Z*)-1-methoxy-3-(pent-2-en-2-yl)benzene 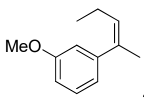 ***Z*-(3ib)**

Colorless oil; ^{1}H NMR ($CDCl_3$, 500 MHz, ppm): δ 7.27 (t, J = 7.9 Hz, 1H), 6.82-6.79 (m, 2H), 6.76 (t, J = 1.7 Hz, 1H), 5.44 (td, J_1 = 1.4 Hz, J_2 = 7.5 Hz, 1H), 3.84 (s, 3H), 2.04-1.98 (m, 5H), 0.96 (t, J = 7.5 Hz, 3H); ^{13}C NMR ($CDCl_3$, 125 MHz, ppm): δ 159.3, 143.7, 135.3, 129.6, 129.0, 120.5, 113.7, 111.7, 55.2, 25.5, 22.5, 14.7; HRMS calc. for $C_{12}H_{17}O$ $(M+H)^+$, 177.12739; found, 177.12706.

(*E*)-1-(pent-2-en-2-yl)-4-phenoxybenzene 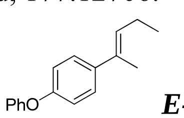 ***E*-(3ic)**

Colorless oil; ^{1}H NMR ($CDCl_3$, 500 MHz, ppm): δ 7.40-7.34 (m, 4H), 7.18 (tt, J_1 = 2.8 Hz, J_2 = 7.4 Hz, 1H), 7.05-7.03 (m, 2H), 6.99 (d, J = 8.8Hz, 2H), 5.78 (tq, J_1 = 1.2

Hz, J_2 = 7.2 Hz, 1H), 2.24 (quint, J = 7.5 Hz, 2H), 2.05 (d, J = 1.2 Hz, 3H), 1.09 (t, J = 7.5 Hz, 3H); ^{13}C NMR ($CDCl_3$, 125 MHz, ppm): δ 157.5, 155.7, 139.2, 133.3, 129.7, 129.7, 126.8, 123.0, 118.7, 118.6, 22.1, 15.7, 14.2; HRMS calc. $C_{17}H_{19}O$ $(M+H)^+$, 239.14304; found, 239.14258.

(*Z*)-1-(pent-2-en-2-yl)-4-phenoxybenzene 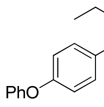 ***E*-(3ic)**

Colorless oil; ^{1}H NMR ($CDCl_3$, 500 MHz, ppm): δ 7.38-7.35 (m, 2H), 7.18 (dt, J_1 = 2.8 Hz, J_2 = 8.7 Hz, 2H), 7.14-7.11 (m, 1H), 7.07-7.05 (m, 2H), 6.99 (dt, J_1 = 2.8 Hz, J_2 = 8.7 Hz, 2H), 5.47 (td, J_1 = 1.5 Hz, J_2 = 7.4 Hz, 1H), 2.07-2.01 (m, 5H), 0.98 (t, J = 7.5 Hz, 3H); ^{13}C NMR ($CDCl_3$, 125 MHz, ppm): δ 157.3, 155.7, 137.1, 134.6, 129.7, 129.6, 129.3, 123.2, 118.9, 118.3, 25.5, 22.5, 14.7; HRMS calc. $C_{17}H_{19}O$ $(M+H)^+$, 239.14304; found, 239.14264.

(*E*)-4-(pent-2-en-2-yl)biphenyl 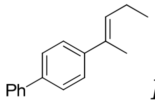 ***E*-(3id)**

White solid; ^{1}H NMR ($CDCl_3$, 500 MHz, ppm): δ 7.63 (d, J = 8.4 Hz, 2H), 7.58 (d, J = 8.4 Hz, 2H), 7.51-7.45 (m, 4H), 7.36 (t, J = 7.4 Hz, 1H), 5.89 (td, J_1 = 1.2 Hz, J_2 = 7.1 Hz, 1H), 2.28 (quint, J = 7.1 Hz, 2H), 2.10 (s, 3H), 1.12 (t, J = 7.5 Hz, 3H); ^{13}C NMR ($CDCl_3$, 125 MHz, ppm): δ 142.9, 140.9, 139.2, 133.6, 130.4, 128.7, 127.1, 127.0, 126.9, 125.9, 22.1, 15.6, 14.1; HRMS calc. $C_{17}H_{19}$ $(M+H)^+$, 223.14813; found, 223.14774.

(*Z*)-4-(pent-2-en-2-yl)biphenyl 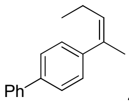 ***Z*-(3id)**

Colorless oil; ^{1}H NMR ($CDCl_3$, 500 MHz, ppm): δ 7.64 (d, J = 8.4 Hz, 2H), 7.58 (d, J = 8.4 Hz, 2H), 7.47 (t, J = 7.5 Hz, 2H), 7.37 (t, J = 7.4 Hz, 1H), 7.30 (d, J = 8.3 Hz, 2H), 5.52 (td, J_1 = 1.4 Hz, J_2 = 7.4 Hz, 1H), 2.11-2.06 (m, 5H), 1.00 (t, J = 7.5 Hz, 3H); ^{13}C NMR ($CDCl_3$, 125 MHz, ppm): δ 141.1, 141.0, 139.2, 134.9, 129.9, 128.7, 128.4, 127.1, 127.0, 126.7, 25.4, 22.6, 14.8; HRMS calc. $C_{17}H_{19}$ $(M+H)^+$, 223.14813; found, 223.14760.

(*E*)-1-chloro-4-(pent-2-en-2-yl)benzene 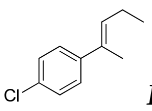 ***E*-(3ie)**

Colorless oil; ^{1}H NMR ($CDCl_3$, 500 MHz, ppm): δ 7.33 (dt, J_1 = 2.3 Hz, J_2 = 8.8 Hz, 2H), 7.29 (dt, J_1 = 2.3 Hz, J_2 = 8.8 Hz, 2H), 5.79 (tq, J_1 = 1.3 Hz, J_2 = 7.1 Hz, 1H), 2.23 (quint, J = 7.5 Hz, 2H), 2.03 (d, J = 1.3 Hz, 3H), 1.09 (t, J = 7.5 Hz, 3H); ^{13}C NMR ($CDCl_3$, 125 MHz, ppm): δ 142.4, 133.0, 132.1, 130.8, 128.2, 126.9, 22.1, 15.6, 14.0; HRMS calc. for $C_{11}H_{14}Cl$ $(M+H)^+$, 181.07785; found, 181.07753.

(*Z*)-1-chloro-4-(pent-2-en-2-yl)benzene 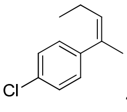 ***Z*-(3ie)**

Colorless oil; ^{1}H NMR ($CDCl_3$, 500 MHz, ppm): δ 7.31 (dt, J_1 = 2.5 Hz, J_2 = 8.5 Hz, 2H), 7.13 (dt, J_1 = 2.5 Hz, J_2 = 8.5 Hz, 2H), 5.49 (td, J_1 = 1.3 Hz, J_2 = 7.4 Hz, 1H),

2.02 (d, J = 1.3 Hz, 3H), 1.98 (quint, J = 7.5 Hz, 2H), 0.95 (t, J = 7.5 Hz, 3H); ^{13}C NMR ($CDCl_3$, 125 MHz, ppm): δ 140.5, 134.2, 132.1, 130.2, 129.3, 128.2, 25.3, 22.4, 14.6; HRMS calc. for $C_{11}H_{14}Cl$ $(M+H)^+$, 181.07785; found, 181.07769.

(*E*)-1-bromo-4-(pent-2-en-2-yl)benzene 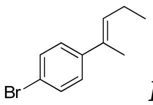 ***E*-(3if)**

Colorless oil; ^{1}H NMR ($CDCl_3$, 500 MHz, ppm): δ 7.44 (dt, J_1 = 2.6 Hz, J_2 = 8.7 Hz, 2H), 7.27 (dt, J_1 = 2.6 Hz, J_2 = 8.7 Hz, 2H), 5.79 (tq, J_1 = 1.3 Hz, J_2 = 7.1 Hz, 1H), 2.22 (quint, J = 7.6 Hz, 2H), 2.02 (d, J = 1.2 Hz, 3H), 1.08 (t, J = 7.6 Hz, 3H); ^{13}C NMR ($CDCl_3$, 125 MHz, ppm): δ 142.8, 133.1, 131.1, 130.9, 127.2, 120.2, 22.1, 15.5, 14.0; HRMS calc. for $C_{11}H_{14}Br$ $(M+H)^+$, 225.02734; found, 225.02725.

(*Z*)-1-bromo-4-(pent-2-en-2-yl)benzene 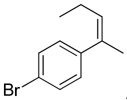 ***Z*-(3if)**

Colorless oil; ^{1}H NMR ($CDCl_3$, 500 MHz, ppm): δ 7.47 (dt, J_1 = 2.5 Hz, J_2 = 8.5 Hz, 2H), 7.08 (dt, J_1 = 2.5 Hz, J_2 = 8.4 Hz, 2H), 5.49 (td, J_1 = 1.4 Hz, J_2 = 7.4 Hz, 1H), 2.01 (d, J = 1.4 Hz, 3H), 2.00-1.94 (m, 2H), 0.95 (t, J = 7.5 Hz, 3H); ^{13}C NMR ($CDCl_3$, 125 MHz, ppm): δ 141.0, 134.3, 131.1, 130.2, 129.7, 120.2, 25.3, 22.4, 14.6; HRMS calc. for $C_{11}H_{14}Br$ $(M+H)^+$, 225.02734; found, 225.02727.

1-fluoro-2-(pent-2-en-2-yl)benzene 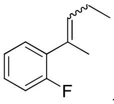 ***E/Z*-(3ig)**

Colorless oil; ^{1}H NMR ($CDCl_3$, 500 MHz, ppm): δ 7.28-7.19 (m, 2.41H), 7.17-7.01 (m, 3.37H), 5.63-5.58 (m, 1.45H), 2.23 (quint, J = 7.4 Hz, 2.04H), 2.03 (s, 4.39H), 1.89 (quint, J = 7.4 Hz, 0.9H), 1.09 (t, J = 7.5 Hz, 3H), 0.95 (t, J = 7.5 Hz, 1.37H); ^{13}C NMR ($CDCl_3$, 125 MHz, ppm): δ 159.9 (d, J = 244.8 Hz), 159.5 (d, J = 243.4 Hz), 133.5 (d, J = 2.0 Hz), 132.8 (d, J = 14.1 Hz), 131.7, 130.7, 130.4 (d, J = 4.6 Hz), 129.9, 129.7 (d, J = 4.7 Hz), 129.5 (d, J = 16.9 Hz), 128.2 (d, J = 8.0 Hz), 127.9 (d, J = 8.2 Hz), 123.8 (d, J = 3.5 Hz), 123.7 (d, J = 3.5 Hz), 115.6 (d, J = 22.7 Hz), 115.5 (d, J = 22.6 Hz), 24.7 (d, J = 1.6 Hz), 22.7, 21.7, 16.7(d, J = 3.7 Hz), 14.2, 13.9; HRMS calc. $C_{11}H_{14}F$ $(M+H)^+$, 165.10741; found, 165.10719.

2-fluoro-4-methoxy-1-(pent-2-en-2-yl)benzene 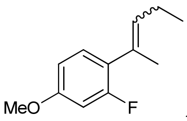 ***E/Z*-(3ih)**

Colorless oil; ^{1}H NMR ($CDCl_3$, 500 MHz, ppm): δ 7.15 (t, J= 8.7 Hz, 0.66H), 7.04 (t, J = 8.4 Hz, 0.34H), 6.71-6.59 (m, 2H), 5.56 (t, J= 7.4 Hz, 1H), 3.82 (s, 1.02H), 3.81 (s, 1.98H), 2.21 (quint, J= 7.5 Hz, 1.32H), 2.00 (s, 3H), 1.88(quint, J= 7.5 Hz, 0.68H), 1.07 (t, J = 7.5 Hz, 1.98H), 0.94 (t, J = 7.5 Hz, 1.02H); ^{13}C NMR ($CDCl_3$, 125 MHz, ppm): δ 160.4 (d, J= 244.9 Hz), 159.9 (d, J= 243.9 Hz), 159.5 (d, J= 10.6 Hz), 159.4 (d, J= 10.8 Hz), 132.6 (d, J= 2.2 Hz), 131.6, 130.6 (d, J= 6.6 Hz), 130.2 (d, J= 1.1 Hz), 130.0 (d, J= 6.6 Hz), 129.6, 125.1 (d, J= 14.5 Hz), 121.5 (d, J= 17.5 Hz), 109.6 (d, J= 3.1 Hz), 109.5 (d, J= 2.8 Hz), 101.7 (d, J= 26.7 Hz), 101.6 (d, J= 26.4 Hz), 55.53, 55.50, 24.8 (d, J= 1.7 Hz), 22.7, 21.7, 16.8 (d, J= 3.6 Hz), 14.2, 14.0;

HRMS calc. $C_{12}H_{16}OF$ $(M+H)^+$, 195.11797, found, 195.11792.

(*E*)-methyl(4-(pent-2-en-2-yl)phenyl)sulfane 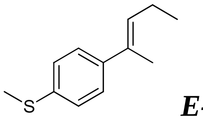 ***E*-(3ii)**

Colorless oil; ^{1}H NMR ($CDCl_3$, 500 MHz, ppm): δ 7.34 (d, J = 8.5 Hz, 2H), 7.14 (d, J = 8.5 Hz, 2H), 5.79 (dt, J_1 = 1.4 Hz, J_2 = 7.2 Hz, 1H), 2.51 (s, 3H), 2.23 (quint, J = 7.5 Hz, 2H), 2.03 (d, J = 1.2 Hz, 3H), 1.08 (t, J = 7.5 Hz, 3H); ^{13}C NMR ($CDCl_3$, 125 MHz, ppm): δ 141.0, 136.1, 133.4, 130.0, 126.7, 126.0, 22.1, 16.2, 15.5, 14.1; HRMS calc. $C_{12}H_{16}S$ $(M+H)^+$, 193.10455, found, 193.10444.

(*Z*)-methyl(4-(pent-2-en-2-yl)phenyl)sulfane 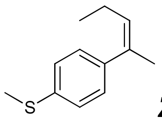 ***Z*-(3ii)**

Colorless oil; ^{1}H NMR ($CDCl_3$, 500 MHz, ppm): δ 7.25 (d, J = 8.4 Hz, 2H), 7.14 (d, J = 8.4 Hz, 2H), 5.47 (dt, J_1 = 1.8 Hz, J_2 = 7.5 Hz, 1H), 2.52 (s, 3H), 2.04-1.98 (m, 5H), 0.96 (t, J = 7.5 Hz, 3H); ^{13}C NMR ($CDCl_3$, 125 MHz, ppm): δ 139.1, 136.1, 134.7, 129.8, 128.5, 126.4, 25.3, 22.5, 16.0, 14.7; HRMS calc. $C_{12}H_{16}S$ $(M+H)^+$, 193.10455, found, 193.10449.

2-(4-(pent-2-en-2-yl)phenyl)propan-2-ol 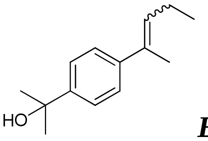 ***E*/*Z*-(3ij)**

Colorless oil; ^{1}H NMR ($CDCl_3$, 500 MHz, ppm): δ 7.48-7.44 (m, 3.14H), 7.39 (d, J = 8.5 Hz, 2H), 7.19 (d, J = 8.3 Hz, 1.14H), 5.82 (t, J_1 = 1.4 Hz, J_2 = 7.2 Hz, 1H), 5.48 (t, J_1 = 1.4 Hz, J_2 = 7.4 Hz, 0.57H), 2.24 (quint, J = 7.5 Hz, 2H), 2.06-2.00 (m, 5.75H), 1.77 (brs, 1.58H), 1.63 (s, 3.42H), 1.61 (s, 6H), 1.08 (t, J = 7.5 Hz, 3H), 0.97 (t, J = 7.5 Hz, 1.71H); ^{13}C NMR ($CDCl_3$, 125 MHz, ppm): δ 147.3, 147.1, 142.4, 140.5, 135.0, 133.6, 130.2, 129.7, 127.8, 125.4, 124.2, 124.1, 72.4, 72.4, 31.7, 25.4, 22.5, 22.1, 15.6, 14.7, 14.1; HRMS calc. $C_{14}H_{20}ONa$$(M+Na)^+$, 227.14064, found, 227.14064.

1-(1-methoxyethyl)-4-(pent-2-en-2-yl)benzene 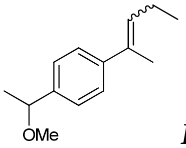 ***E*/*Z*-(3ik)**

Colorless oil; ^{1}H NMR ($CDCl_3$, 500 MHz, ppm): δ 7.39 (d, J = 8.3 Hz, 2H), 7.29-7.26 (m, 2.88H), 7.19 (d, J = 8.1 Hz, 0.88H), 5.82 (t, J_1 = 1.4 Hz, J_1 = 7.2 Hz, 1H), 5.47 (t, J_1 = 1.4 Hz, J_1 = 7.4 Hz, 0.44H), 4.34-4.29 (m, 1.44H), 3.27 (s, 1.32H), 3.25 (s, 3H), 2.24 (quint, J = 7.5 Hz, 2H), 2.06-2.01 (m, 5.07H), 1.48-1.45(m, 4.34H), 1.08 (t, J = 7.5 Hz, 3H), 0.97 (t, J = 7.5 Hz, 1.32H); ^{13}C NMR ($CDCl_3$, 125 MHz, ppm): δ 143.2, 141.6, 141.5, 141.3, 135.1, 133.7, 130.2, 129.6, 128.0, 126.0, 125.8, 125.6, 79.5, 79.4, 56.5, 56.4, 25.5, 23.8, 23.7, 22.5, 22.1, 15.6, 14.7, 14.1; HRMS calc. $C_{14}H_{20}ONa$$(M+Na)^+$, 227.14064, found, 227.14060.

(*E*)1-(4-(pent-2-en-2-yl)phenyl)piperidine 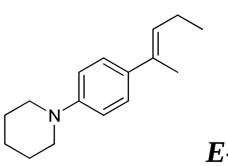 ***E*-(3il)**

Colorless oil; ^{1}H NMR ($CDCl_3$, 500 MHz, ppm): δ 7.32 (d, J = 8.8 Hz, 2H), 7.50 (d, J = 8.8 Hz, 2H), 5.73 (dt, J_1 = 1.4 Hz, J_2 = 7.2 Hz, 1H), 3.17 (t, J = 5.5 Hz, 4H), 2.22 (quint, J = 7.5 Hz, 2H), 2.06 (d, J= 1.2 Hz, 3H), 1.76-1.72 (m, 4H), 1.62-1.58 (m, 2H), 1.08 (t, J = 7.5 Hz, 3H); ^{13}C NMR ($CDCl_3$, 125 MHz, ppm): δ 150.9, 134.7, 133.4, 127.9, 126.1, 116.2, 50.7, 25.9, 24.3, 22.0, 15.5, 14.3; HRMS calc. $C_{16}H_{24}N$ $(M+H)^+$, 230.19033, found, 230.19014.

(*Z*)1-(4-(pent-2-en-2-yl)phenyl)piperidine 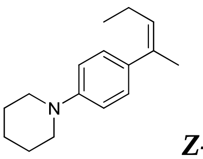 ***Z*-(3il)**

Colorless oil; ^{1}H NMR ($CDCl_3$, 500 MHz, ppm): δ 7.12 (d, J = 8.7 Hz, 2H), 6.92 (d, J = 8.8 Hz, 2H), 5.41 (dt, J_1 = 1.4 Hz, J_2 = 7.2 Hz, 1H), 3.18 (t, J = 5.5 Hz, 4H), 2.08-2.04 (m, 2H), 2.03 (d, J= 1.4 Hz, 3H), 1.76-1.72 (m, 4H), 1.62-1.59 (m, 2H), 0.96 (t, J = 7.5 Hz, 3H); ^{13}C NMR ($CDCl_3$, 125 MHz, ppm): δ 150.6, 134.9, 132.7, 128.8, 128.6, 115.8, 50.6, 25.9, 25.4, 24.3, 22.5, 14.8; HRMS calc. $C_{16}H_{24}N$ $(M+H)^+$, 230.19033, found, 230.19012.

(*E*)-1-(pent-2-en-2-yl)-4-(phenylethynyl)benzene 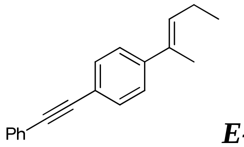 ***E*-(3im)**

Colorless oil; ^{1}H NMR ($CDCl_3$, 500 MHz, ppm): δ 7.57-7.55 (m, 2H), 7.50 (d, J = 8.4 Hz, 2H), 7.42-7.35 (m, 5H), 5.88 (dt, J_1 = 1.4 Hz, J_2 = 7.2 Hz, 1H), 2.26 (quint, J = 7.5 Hz, 2H), 2.06 (d, J= 1.2 Hz, 3H), 1.10 (t, J = 7.5 Hz, 3H); ^{13}C NMR ($CDCl_3$, 125 MHz, ppm): δ 143.7, 133.5, 131.6, 131.4, 131.2, 128.3, 128.1, 125.4, 123.5, 121.1, 89.6, 89.5, 22.2, 15.4, 14.0; HRMS calc. $C_{19}H_{19}$ $(M+H)^+$, 247.14813, found, 247.14718.

(*Z*)-1-(pent-2-en-2-yl)-4-(phenylethynyl)benzene 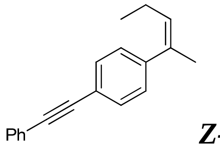 ***Z*-(3im)**

Colorless oil; ^{1}H NMR ($CDCl_3$, 500 MHz, ppm): δ 7.57-7.55 (m, 2H), 7.52 (d, J = 8.3 Hz, 2H), 7.39-7.35 (m, 3H), 7.20 (d, J = 8.3 Hz, 2H), 5.51 (dt, J_1 = 1.5 Hz, J_2 = 7.4 Hz, 1H), 2.05 (d, J = 1.4 Hz, 3H), 2.04-1.99 (m, 2H), 0.97 (t, J = 7.5 Hz, 3H); ^{13}C NMR ($CDCl_3$, 125 MHz, ppm): δ 142.3, 134.8, 131.6, 131.3, 130.3, 128.3, 128.2, 128.0, 123.4, 121.2, 89.5, 89.2, 25.2, 22.5, 14.7; HRMS calc. $C_{19}H_{19}$ $(M+H)^+$, 247.14813, found, 247.14729.

(*E*)-methyl 4-(pent-2-en-2-yl)benzoate 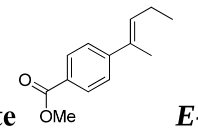 ***E*-(3in)**

Colorless oil; ^{1}H NMR ($CDCl_3$, 500 MHz, ppm): δ 7.99 (d, J = 8.7 Hz, 2H), 7.46 (d, J J = 8.7 Hz, 2H), 5.92 (td, J_1 = 1.4 Hz, J_2 = 7.2 Hz, 1H), 3.93 (s, 3H), 2.26 (quint, J = 7.5 Hz, 2H), 2.06 (d, J = 1.2 Hz, 3H), 1.10 (t, J = 7.5 Hz, 3H); ^{13}C NMR ($CDCl_3$, 125 MHz, ppm): δ 167.1, 148.4, 133.4, 132.5, 129.5, 128.0, 125.4, 52.0, 22.2, 15.4, 13.9; HRMS calc. for $C_{13}H_{16}O_2Na$ $(M+Na)^+$, 227.1043; found, 227.1047.

(*Z*)-methyl 4-(pent-2-en-2-yl)benzoate 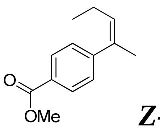 ***Z*-(3in)**

Colorless oil; ^{1}H NMR ($CDCl_3$, 500 MHz, ppm): δ 8.02 (d, J = 8.2 Hz, 2H), 7.27 (d, J = 8.2 Hz, 2H), 5.53 (td, J_1 = 1.3 Hz, J_2 = 7.4 Hz, 1H), 3.94 (s, 3H), 2.05 (d, J = 1.1 Hz, 3H), 1.99 (quint, J = 7.5 Hz, 2H), 0.96 (t, J = 7.5 Hz, 3H); ^{13}C NMR ($CDCl_3$, 125 MHz, ppm): δ 167.1, 147.2, 134.6, 130.8, 129.4, 128.2, 128.0, 52.0, 25.1, 22.5, 14.6; HRMS calc. for $C_{13}H_{16}O_2Na$ $(M+Na)^+$, 227.1043; found, 227.1045.

(4-(pent-2-en-2-yl)phenyl)(pyrrolidin-1-yl)methanone 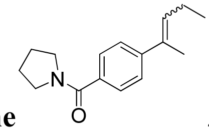 ***Z/E*-(3io)**

Colorless oil; ^{1}H NMR ($CDCl_3$, 500 MHz, ppm): δ 7.51-7.48 (m, 2.75H), 7.41 (d, J = 8.1 Hz, 2H), 7.21 (d, J = 7.4 Hz, 0.82H), 5.84 (t, J = 7.4 Hz, 1H), 5.50 (t, J = 7.4 Hz, 0.41H), 3.69-3.65 (m, 2.82H), 3.51-3.46 (m, 2.82H), 2.24 (quint, J = 7.5 Hz, 2H), 2.04 (s, 3H), 2.03 (s, 1.22H), 2.00-1.95 (m, 3.45H), 1.92-1.86 (m, 2.89H), 1.08 (t, J = 7.5 Hz, 1H), 0.95 (t, J = 7.4 Hz, 1.24H); ^{13}C NMR ($CDCl_3$, 125 MHz, ppm): δ 169.67, 169.66, 145.39, 143.85, 135.21, 135.08, 134.75, 133.47, 131.36, 130.28, 127.80, 127.16, 126.95, 125.27, 49.65, 46.21, 26.45, 25.26, 24.48, 22.42, 22.12, 15.50, 14.64, 14.02; HRMS calc. for $C_{16}H_{22}ON$ $(M+H)^+$, 244.16959; found, 244.17014.

(*E*)-N-butyl-4-(pent-2-en-2-yl)benzamide 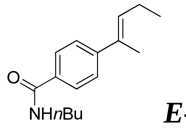 ***E*-(3ip)**

Colorless oil; ^{1}H NMR ($CDCl_3$, 500 MHz, ppm): δ 7.72 (d, J = 8.5 Hz, 2H), 7.44 (d, J = 8.5 Hz, 2H), 6.14 (s, 1H), 5.87 (td, J_1 = 1.4 Hz, J_2 = 7.5 Hz, 1H), 3.50-3.46 (m, 2H), 2.25 (quint, J = 7.5 Hz, 2H), 2.05 (d, J = 1.2 Hz, 2H), 1.65-1.59 (m, 2H), 1.47-1.40 (m, 2H), 1.09 (t, J = 7.5 Hz, 3H), 0.98 (t, J = 7.5 Hz, 3H); ^{13}C NMR ($CDCl_3$, 125 MHz, ppm): δ 167.3, 146.9, 133.3, 132.6, 131.9, 126.7, 125.6, 39.8, 31.8, 22.2, 20.2, 15.5, 14.0, 13.8; HRMS calc. for $C_{16}H_{24}ON$ $(M+H)^+$, 246.18524; found, 246.18564.

(*Z*)-N-butyl-4-(pent-2-en-2-yl)benzamide 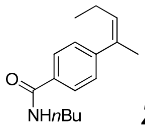 ***Z*-(3ip)**

Colorless oil; ^{1}H NMR ($CDCl_3$, 500 MHz, ppm): δ 7.74 (d, J = 8.3 Hz, 2H), 7.26 (d, J = 8.3 Hz, 2H), 6.12 (s, 1H), 5.52 (td, J_1 = 1.4 Hz, J_2 = 7.5 Hz, 1H), 3.51-3.47 (m, 2H), 2.04 (d, J = 1.4 Hz, 2H), 1.97 (quintd, J_1 = 1.3 Hz, J_2 = 7.5 Hz, 2H), 1.66-1.60 (m, 2H), 1.48-1.41 (m, 2H), 0.99 (t, J = 7.5 Hz, 3H), 0.95 (t, J = 7.5 Hz, 3H); ^{13}C NMR ($CDCl_3$, 125 MHz, ppm): δ 167.4, 145.5, 134.6, 132.9, 130.5, 128.2, 126.6, 39.8, 31.8, 25.2, 22.4, 20.2, 14.6, 13.8; HRMS calc. for $C_{16}H_{24}ON$ $(M+H)^+$, 246.18524; found, 246.18578.

(*E*)-hex-3-en-3-ylbenzene 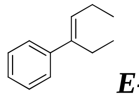 ***E*-(3j)**

Colorless oil; ^{1}H NMR ($CDCl_3$, 500 MHz, ppm): δ 7.39 (d, J = 7.7 Hz, 2H), 7.33 (t, J = 7.4 Hz, 2H), 7.24 (t, J = 7.4 Hz, 1H), 5.67 (t, J = 7.2 Hz, 1H), 2.55 (quint, J = 7.5 Hz, 2H), 2.25 (quint, J = 7.5 Hz, 2H), 1.10 (t, J = 7.5 Hz, 3H), 1.02 (t, J = 7.5 Hz, 3H); ^{13}C NMR ($CDCl_3$, 125 MHz, ppm): δ 143.1, 140.9, 130.0, 128.1, 126.4, 126.3, 22.9, 21.7, 14.5, 13.7; HRMS calc. for $C_{12}H_{17}$ $(M+H)^+$, 161.13248; found, 161.13263.

(*Z*)-hex-3-en-3-ylbenzene 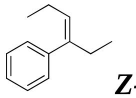 ***Z*-(3j)**

Colorless oil; ^{1}H NMR ($CDCl_3$, 500 MHz, ppm): δ 7.35 (t, J = 7.4 Hz, 2H), 7.26 (tt, J_1 = 1.3 Hz, J_2 = 7.5 Hz, 1H), 7.18-7.16 (m, 2H), 5.45 (t, J = 7.4 Hz, 1H), 2.36 (q, J = 7.5 Hz, 2H), 1.96 (quint, J = 7.5 Hz, 2H), 0.99 (t, J = 7.5 Hz, 3H), 0.95 (t, J = 7.5 Hz, 3H); ^{13}C NMR ($CDCl_3$, 125 MHz, ppm): δ 142.0, 141.6, 128.4, 127.9, 127.7, 126.3, 32.0, 22.2, 14.8, 13.1; HRMS calc. for $C_{12}H_{17}$ $(M+H)^+$, 161.13248; found, 161.13280.

(*E*)-hept-3-en-4-ylbenzene 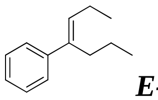 ***E*-(3k)**

Colorless oil; ^{1}H NMR ($CDCl_3$, 500 MHz, ppm): δ 7.39-7.37 (m, 2H), 7.34-7.31 (m, 2H), 7.24 (tt, J_1 = 1.3 Hz, J_2 = 7.2 Hz, 1H), 5.69 (t, J = 7.2 Hz, 1H), 2.51 (t, J = 7.5 Hz, 2H), 2.25 (quint, J = 7.5 Hz, 2H), 1.44-1.37 (m, 2H), 1.09 (t, J = 7.5 Hz, 3H), 0.92 (t, J = 7.4 Hz, 3H); ^{13}C NMR ($CDCl_3$, 125 MHz, ppm): δ 143.4, 139.4, 130.9, 128.1, 126.4, 126.3, 31.6, 21.9, 21.8, 14.5, 13.9; HRMS calc. for $C_{13}H_{19}$ $(M+H)^+$, 175.14813; found, 175.14864.

(*Z*)-hept-3-en-4-ylbenzene 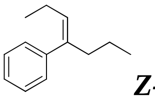 ***Z*-(3k)**

Colorless oil; ^{1}H NMR ($CDCl_3$, 500 MHz, ppm): δ 7.36-7.32 (m, 2H), 7.25 (tt, J_1 = 1.3 Hz, J_2 = 7.4 Hz, 1H), 7.17-7.15 (m, 2H), 5.45 (t, J = 7.4 Hz, 1H), 2.32 (t, J = 7.5 Hz, 2H), 1.96 (quint, J = 7.5 Hz, 2H), 1.37-1.31 (m, 2H), 0.95 (t, J = 7.5 Hz, 3H), 0.89 (t, J = 7.4 Hz, 3H); ^{13}C NMR ($CDCl_3$, 125 MHz, ppm): δ 141.5, 140.2, 129.1, 128.4, 127.9, 126.2, 41.3, 22.2, 21.2, 14.8, 13.6; HRMS calc. for $C_{13}H_{19}$ $(M+H)^+$, 175.14813; found, 175.14834.

(*E*)-non-3-en-4-ylbenzene 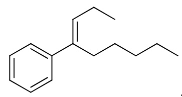 ***E*-(3l)**

Colorless oil; ^{1}H NMR ($CDCl_3$, 500 MHz, ppm): δ 7.38-7.36 (m, 2H), 7.34-7.30 (m, 2H), 7.24 (tt, J_1 = 1.3 Hz, J_2 = 7.2 Hz, 1H), 5.67 (t, J = 7.2 Hz, 1H), 2.51 (t, J = 7.2 Hz, 2H), 2.24 (quint, J = 7.5 Hz, 2H), 1.39-1.34 (m, 2H), 1.31-1.29 (m, 4H), 1.08 (t, J = 7.5 Hz, 3H), 0.88 (t, J = 7.0 Hz, 3H); ^{13}C NMR ($CDCl_3$, 125 MHz, ppm): δ 143.4, 139.6, 130.6, 128.1, 126.4, 126.3, 31.8, 29.7, 28.5, 22.5, 21.9, 14.5, 14.1; HRMS calc. for $C_{15}H_{23}$ $(M+H)^+$, 203.17943; found, 203.17949.

(*Z*)-non-3-en-4-ylbenzene 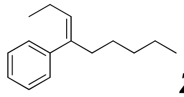 ***Z*-(3l)**

Colorless oil; ^{1}H NMR ($CDCl_3$, 500 MHz, ppm): δ 7.36-7.33 (m, 2H), 7.26 (tt, J_1 = 1.3 Hz, J_2 = 7.4 Hz, 1H), 7.17-7.16 (m, 2H), 5.45 (t, J = 7.4 Hz, 1H), 2.34 (t, J = 7.4 Hz, 2H), 1.96 (quint, J = 7.5 Hz, 2H), 1.34-1.31 (m, 2H), 1.29-1.27 (m, 4H), 0.95 (t, J = 7.5 Hz, 3H), 0.88 (t, J = 7.0 Hz, 3H); ^{13}C NMR ($CDCl_3$, 125 MHz, ppm): δ 141.6, 140.5, 128.8, 128.4, 127.9, 126.2, 39.2, 31.4, 27.9, 22.5, 22.2, 14.8, 14.1; HRMS calc. for $C_{15}H_{23}$ $(M+H)^+$, 203.17943; found, 203.17951.

(*E*)-but-1-enylbenzene 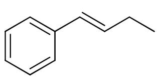 **(3m)**

Colorless oil; The spectroscopic data correspond to those previously reported in the literature.[4] ^{1}H NMR ($CDCl_3$, 500 MHz, ppm): δ 7.38-7.36 (m, 2H), 7.31 (t, J = 7.5 Hz, 2H), 7.21 (t, J = 7.3 Hz, 1H), 6.41(d, J = 15.9 Hz, 1H), 6.31 (dt, J_1 = 6.4 Hz, J_2 = 15.9 Hz, 1H), 2.26 (quint, J = 7.5 Hz, 2H), 1.12 (t, J = 7.5 Hz, 3H); ^{13}C NMR ($CDCl_3$, 125 MHz, ppm): δ 137.9, 132.7, 128.8, 128.5, 126.7, 125.9, 26.1, 13.7; MS (EI) m/z: 132.1, 117.1, 91.1.

(5S,8R,9S,10S,13R,14S,17R)-10,13-dimethyl-17-((R)-6-methylheptan-2-yl)-3-propylidenehexadecahydro-1H-cyclopenta[a]phenanthrene

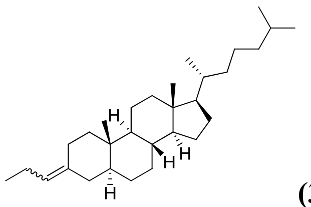

(3o)

Colorless oil; ^{1}H NMR ($CDCl_3$, 500 MHz, ppm): δ 5.08 (t, J = 7.0 Hz, 1H), 2.22-2.16 (m, 1H), 2.03-1.96 (m, 4H), 1.87-1.65 (m, 5H), 1.59-1.49 (m, 3H), 1.38-1.24 (m, 9H), 1.17-0.98 (m, 10H), 0.95 (t, J = 7.5 Hz, 3H), 0.92 (d, J = 6.5 Hz, 3H), 0.91-0.88 (m 10H), 0.68 (s, 3H); ^{13}C NMR ($CDCl_3$, 125 MHz, ppm): δ 138.8, 138.7, 122.9, 122.7, 56.6, 56.3, 54.53, 54.49, 48.4, 47.6, 42.62, 42.61, 40.13, 40.11, 39.54, 39.50, 39.46, 36.53, 36.49, 36.2, 35.8, 35.5, 32.5, 32.1, 32.0, 31.2, 29.2, 28.9, 28.3, 28.0, 24.2, 24.1, 23.8, 22.8, 22.6, 21.1, 21.0, 20.3, 18.7, 14.90, 14.87, 12.1, 11.82, 11.80; HRMS calc. for $C_{30}H_{53}$ $(M+H)^+$, 413.41418; found, 413.41460.

(2-methylhex-3-en-3-yl)benzene 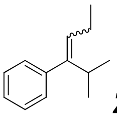 ***Z/E*-(3p)**

Colorless oil; ^{1}H NMR ($CDCl_3$, 500 MHz, ppm): *δ* 7.36-7.32 (m, 2H), 7.30-7.23 (m, 3.5H), 7.20-7.18 (m, 1.5H), 7.11-7.09 (m, 2H), 5.42 (t, J_1 =1.2 Hz, J_2 =7.3 Hz, 1H), 5.28 (t, *J* =7.3 Hz, 0.75H), 3.10-3.02 (m, 0.75H), 2.58-2.53 (m, 1H), 2.23 (quint, *J* = 7.4 Hz, 1.5H), 1.84 (quint, *J* = 7.2Hz, 2H), 1.08-1.05 (m, 6.75H), 1.02 (d, *J* = 6.8 Hz, 6H), 0.91 (d, *J* = 7.5 Hz, 3H); ^{13}C NMR ($CDCl_3$, 125 MHz, ppm): *δ* 146.7, 146.2, 143.5, 141.5, 130.7, 128.9, 128.6, 127.7, 127.4, 126.3, 126.1, 126.0, 35.7, 29.2, 22.1, 21.9, 21.8, 21.0, 14.7, 14.6; HRMS calc. for $C_{13}H_{19}$ $(M+H)^+$, 175.14813; found, 175.14786.

propylidenecyclohexane 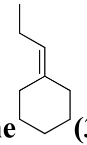 **(3q)**

Colorless oil; ^{1}H NMR ($CDCl_3$, 500 MHz, ppm): *δ* 5.09 (t, *J* = 7.2 Hz, 1H), 2.14 (t, *J* = 6.2 Hz, 2H), 2.08 (t, *J* = 6.0 Hz, 2H), 2.01 (quint, *J* = 7.5 Hz, 2H), 1.57-1.50 (m, 6H), 0.95 (t, *J* = 7.5 Hz, 3H); ^{13}C NMR ($CDCl_3$, 125 MHz, ppm): *δ* 139.0, 123.1, 37.1, 28.7, 28.6, 27.9, 27.0, 20.3, 14.9; HRMS calc. for C_9H_{17} $(M+H)^+$, 125.13248; found, 125.13276.

(4-propylidenecyclohexyl)benzene 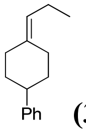 **(3r)**

Colorless oil; ^{1}H NMR ($CDCl_3$, 500 MHz, ppm): *δ* 7.32 (t, *J* = 8.0 Hz, 2H), 7.25-7.19 (m, 3H), 5.19 (t, *J* = 7.3 Hz, 1H), 2.78-2.68 (m, 2H), 2.34-2.29 (m, 1H), 2.25-2.19 (m, 1H), 2.10-2.03 (m, 2H), 2.02-1.96 (m, 2H), 1.93-1.86 (m, 1H), 1.59-1.43 (m, 2H), 1.00 (t, *J* = 7.5 Hz, 3H); ^{13}C NMR ($CDCl_3$, 125 MHz, ppm): *δ* 147.2, 137.6, 128.3, 126.9, 125.9, 124.0, 44.9, 36.8, 35.9, 35.2, 28.3, 20.5, 14.9; HRMS calc. for $C_{15}H_{21}$ $(M+H)^+$, 201.16378; found, 201.16400.

prop-1-ene-1,1-diyldibenzene 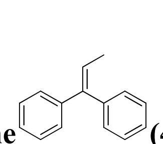 **(4a)**

White solid, mp. 50-50.8°C, The spectroscopic data correspond to those previously reported in the literature.[5] ^{1}H NMR ($CDCl_3$, 500 MHz, ppm): *δ* 7.42 (t, *J* = 7.2 Hz, 2H), 7.34 (t, *J* = 7.4 Hz, 1H), 7.31-7.22 (m, 7H), 6.22 (q, *J* = 7.0 Hz, 1H), 1.80 (d, *J* = 7.0 Hz, 3H); ^{13}C NMR ($CDCl_3$, 125 MHz, ppm): *δ* 143.0, 142.4, 140.0, 130.1, 128.2, 128.1, 127.2, 126.8, 126.7, 124.2, 15.7; MS (EI) m/z: 194.1, 178.1, 165.1, 115.0.

pent-1-ene-1,1-diyldibenzene 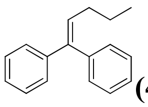 **(4b)**

Colorless oil; The spectroscopic data correspond to those previously reported in the literature.[6] ^{1}H NMR ($CDCl_3$, 500 MHz, ppm): δ 7.41 (t, J = 7.1 Hz, 2H), 7.35 (tt, J_1 = 1.4 Hz, J_2 = 7.4 Hz, 1H), 7.31-7.22 (m, 7H), 6.14 (t, J = 7.5 Hz, 1H), 2.14 (q, J = 7.5 Hz, 2H), 1.55-1.48 (m, 2H), 0.95 (t, J = 7.5 Hz, 3H); ^{13}C NMR ($CDCl_3$, 125 MHz, ppm): δ 142.9, 141.6, 140.4, 130.1, 130.0, 128.1, 128.0, 127.2, 126.8, 126.7, 31.9, 23.2, 13.9; MS (EI) m/z: 222.2, 193.2, 165.2, 115.2, 91.2.

hex-1-ene-1,1-diyldibenzene 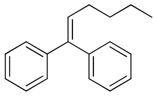 **(4c)**

Colorless oil; The spectroscopic data correspond to those previously reported in the literature.[7] ^{1}H NMR ($CDCl_3$, 500 MHz, ppm): δ 7.42 (tt, J_1 = 1.1 Hz, J_2 = 7.1 Hz, 2H), 7.34 (tt, J_1 = 2.7 Hz, J_2 = 7.4 Hz, 1H), 7.31-7.21 (m, 7H), 6.13 (t, J = 7.5 Hz, 1H), 2.15 (q, J = 7.5 Hz, 2H), 1.49-1.43 (m, 2H), 1.39-1.32 (m, 2H), 0.90 (t, J = 7.4 Hz, 3H); ^{13}C NMR ($CDCl_3$, 125 MHz, ppm): δ 142.9, 141.4, 140.4, 130.3, 130.0, 128.1, 128.0, 127.2, 126.8, 126.7, 32.2, 29.5, 22.4, 14.0; MS (EI) m/z: 236.2, 193.2, 178.2, 165.1, 115.1.

non-1-ene-1,1-diyldibenzene 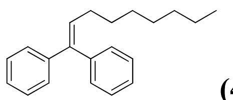 **(4d)**

Colorless oil; The spectroscopic data correspond to those previously reported in the literature.[8] ^{1}H NMR ($CDCl_3$, 500 MHz, ppm): δ 7.40 (t, J = 7.2 Hz, 2H), 7.34 (t, J = 7.5 Hz, 1H), 7.31-7.21 (m, 7H), 6.12 (t, J = 7.5 Hz, 1H), 2.15 (q, J = 7.5 Hz, 2H), 1.50-1.44 (m, 2H), 1.33-1.28 (m, 8H), 0.91 (t, J = 7.4 Hz, 3H); ^{13}C NMR ($CDCl_3$, 125 MHz, ppm): δ 142.9, 141.4, 140.4, 130.4, 130.0, 128.1, 128.0, 127.2, 126.8, 126.7, 31.8, 30.0, 29.8, 29.3, 29.2, 22.7, 14.1; MS (EI) m/z: 278.1, 193.1, 165.1, 115.1.

prop-1-ene-1,1,3-triyltribenzene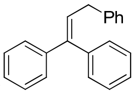**(4e)**

Colorless oil; The spectroscopic data correspond to those previously reported in the literature.[9] ^{1}H NMR ($CDCl_3$, 500 MHz, ppm): δ 7.43 (t, J = 7.1 Hz, 2H), 7.38-7.32 (m, 3H), 7.31-7.28 (m, 6H), 7.27-7.22 (m, 4H), 6.32 (t, J = 7.6 Hz, 1H), 3.52 (d, J = 7.6Hz, 1H); ^{13}C NMR ($CDCl_3$, 125 MHz, ppm): δ 142.5, 142.4, 141.0, 139.8, 129.9, 128.5, 128.4, 128.3, 128.1, 127.8, 127.4, 127.2, 127.1, 126.0, 35.9; MS (EI) m/z: 270.1, 255.2, 192.1, 178.1, 115.1.

(*E*)-but-2-ene-1,3-diyldibenzene 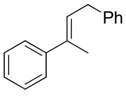 ***E*-(4f)**

Colorless oil; ^{1}H NMR ($CDCl_3$, 500 MHz, ppm): δ 7.46-7.44 (m, 2H), 7.36-7.32 (m, 4H), 7.29-7.23 (m, 4H), 6.01 (tq, J_1 = 1.3 Hz, J_2 = 7.4 Hz, 1H), 3.61 (d, J = 7.4 Hz, 2H), 2.19 (d, J = 0.8 Hz, 3H); ^{13}C NMR ($CDCl_3$, 125 MHz, ppm): δ 143.6, 141.0, 135.7, 128.5, 128.4, 128.2, 126.8, 126.7, 125.9, 125.7, 35.0, 16.0; HRMS calc. for

$C_{16}H_{16}$ (M)$^+$, 208.12465; found, 208.12451.

(*Z*)-but-2-ene-1,3-diyldibenzene 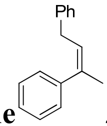 ***Z*-(4f)**

Colorless oil; ^{1}H NMR ($CDCl_3$, 500 MHz, ppm): δ 7.38 (t, J = 7.4 Hz, 2H), 7.32-7.27 (m, 5H), 7.22-7.18 (m, 3H), 5.69 (td, J_1 = 1.4 Hz, J_2 = 7.5 Hz, 1H), 3.36 (d, J = 7.5 Hz, 2H), 2.11(d, J = 1.3 Hz, 3H); ^{13}C NMR ($CDCl_3$, 125 MHz, ppm): δ 141.8, 141.5, 137.4, 128.4, 128.3, 128.2, 127.9, 126.7, 125.8, 125.7, 35.3, 25.7; HRMS calc. for $C_{16}H_{16}$ (M)$^+$, 208.12465; found, 208.12390.

ethene-1,1,2-triyltribenzene 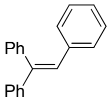 **(4g)**

Colorless oil; The spectroscopic data correspond to those previously reported in the literature.[10] ^{1}H NMR ($CDCl_3$, 500 MHz, ppm): δ 7.38-7.31 (m, 8H), 7.26-7.23 (m, 2H), 7.18-7.13 (m, 3H), 7.07-7.06 (m, 2H), 7.00 (s, 1H); ^{13}C NMR ($CDCl_3$, 125 MHz, ppm): δ 143.4, 142.6, 140.4, 137.4, 130.4, 129.6, 128.6, 128.2, 128.1, 127.9, 127.6, 127.5, 127.4, 126.8; MS (EI) m/z: 256.1, 239.1, 178.1, 165.1.

(*E*)-1,2-diphenylethene 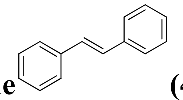 **(4h)**

White solid; mp. 124.6-126.1°C. The spectroscopic data correspond to those previously reported in the literature.[11] ^{1}H NMR ($CDCl_3$, 500 MHz, ppm): δ 7.56 (d, J = 7.7 Hz, 4H), 7.40 (t, J = 7.6 Hz, 4H), 7.30 (t, J = 7.4 Hz, 2H), 7.16 (s, 2H); ^{13}C NMR ($CDCl_3$, 125 MHz, ppm): δ 137.3, 128.7, 127.6, 126.5; MS (EI) m/z: 236.2, 193.2, 178.2, 165.1, 115.1.

(*E*)-1-methoxy-4-styrylbenzene 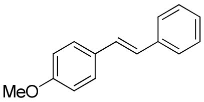 **(4i)**

White solid, mp 108.4-108.6°C; The spectroscopic data correspond to those previously reported in the literature.[11a] ^{1}H NMR ($CDCl_3$, 500 MHz, ppm): δ 7.52 (d, J = 7.6 Hz, 2H), 7.49 (d, J = 8.7 Hz, 2H), 7.38 (t, J = 7.6 Hz, 2H), 7.27 (t, J = 7.4 Hz, 1H), 7.10 (d, J = 16.3 Hz, 1H), 7.01 (d, J = 16.3 Hz, 1H), 6.93 (d, J = 8.7 Hz, 2H), 3.86 (s, 3H); ^{13}C NMR ($CDCl_3$, 125 MHz, ppm): δ 159.3, 137.7, 130.2, 128.7, 128.2, 127.7, 127.2, 126.6, 126.3, 114.1, 55.3; MS (EI) m/z: 210.1, 165.1, 152.1, 104.1.

(*E*)-N,N-dimethyl-4-styrylaniline 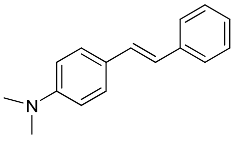 **(4j)**

White solid, mp 144.2-145.6°C. The spectroscopic data correspond to those previously reported in the literature.[13] ^{1}H NMR ($CDCl_3$, 500 MHz, ppm): δ 7.51 (d, J = 6.9 Hz, 2H), 7.45 (d, J = 8.8 Hz, 2H), 7.36 (t, J = 7.6 Hz, 2H), 7.23 (t, J = 7.3 Hz, 1H), 7.09 (d, J = 16.4 Hz, 1H), 6.95 (d, J = 16.4 Hz, 1H), 6.76 (d, J = 8.8 Hz, 2H), 3.02 (s, 6H); ^{13}C NMR ($CDCl_3$, 125 MHz, ppm): δ 150.1, 138.2, 128.8, 128.6, 127.6,

126.7, 126.0, 125.8, 124.4, 112.5, 40.5; MS (EI) m/z: 223.1, 165.1, 114.1.

(*E*)-1-(allyloxy)-4-styrylbenzene 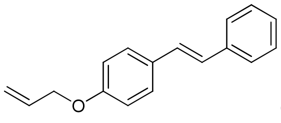 **(4k)**

White solid, mp 124.4-126.2°C. The spectroscopic data correspond to those previously reported in the literature[14]. ^{1}H NMR (CDCl$_3$, 500 MHz, ppm): δ 7.52 (d, J = 6.9 Hz, 2H), 7.48 (d, J = 8.7 Hz, 2H), 7.37 (t, J = 7.7 Hz, 2H), 7.26 (t, J = 7.3 Hz, 1H), 7.08 (d, J = 16.3 Hz, 1H), 7.01 (d, J = 16.3 Hz, 1H), 6.94 (d, J = 8.8 Hz, 2H), 6.13-6.06 (m, 1H), 5.47(dq, J_1 = 1.6 Hz, J_2 = 17.3 Hz, 1H), 5.33(dq, J_1 = 1.5 Hz, J_2 = 10.4 Hz, 1H), 4.59(dt, J_1 = 1.6 Hz, J_2 = 5.3 Hz, 1H); ^{13}C NMR (CDCl$_3$, 125 MHz, ppm): δ 158.3, 137.6, 133.2, 130.3, 128.6, 128.2, 127.7, 127.2, 126.7, 126.3, 117.8, 115.0, 68.9; MS (EI) m/z: 236.1, 195.1, 165.1, 115.1

(*E*)-prop-1-ene-1,2-diyldibenzene 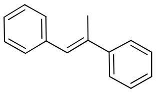 **(4l)**

White solid, mp. 78.2-80.5; The spectroscopic data correspond to those previously reported in the literature.[16] ^{1}H NMR (CDCl$_3$, 500 MHz, ppm): δ 7.58-7.56 (m, 2H), 7.43-7.40 (m, 6H), 7.35-7.32 (m, 1H), 7.29-7.27(m, 1H), 6.88 (d, J = 1.1 Hz, 1H), 2.33 (d, J = 1.4 Hz, 3H); ^{13}C NMR (CDCl$_3$, 125 MHz, ppm): δ 144.0, 138.4, 137.4, 129.2, 128.4, 128.2, 127.7, 127.2, 126.5, 126.0, 17.5; MS (EI) m/z: 194.1, 179.1, 165.1, 115.0.

4-phenyl-1,2-dihydronaphthalene 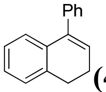 **(4m)**

Colorless oil; The spectroscopic data correspond to those previously reported in the literature[17] . ^{1}H NMR (CDCl$_3$, 500 MHz, ppm): δ 7.44-7.35 (m, 5H), 7.24 (d, J = 7.3 Hz, 1H), 7.20 (t, J = 7.3 Hz, 1H), 7.15 (t, J = 7.6 Hz, 1H), 7.05 (d, J = 7.6 Hz, 1H), 6.13 (t, J = 4.7 Hz, 1H), 2.90 (t, J = 7.9 Hz, 2H), 2.47-2.43 (m, 2H); ^{13}C NMR (CDCl$_3$, 125 MHz, ppm): δ 140.8, 139.9, 136.8, 135.1, 128.8, 128.2, 127.7, 127.6, 127.1, 127.0, 126.2, 125.4, 28.3, 23.6; MS (EI) m/z: 206.1, 191.1, 178.1, 152.0, 128.1.

cyclohexenylbenzene 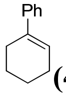 **(4n)**

Colorless oil; The spectroscopic data correspond to those previously reported in the literature[6]. ^{1}H NMR (CDCl$_3$, 500 MHz, ppm): δ 7.42-7.40 (m, 2H), 7.33 (t, J = 7.4 Hz, 2H), 7.24 (tt, J_1 = 1.2 Hz, J_2 = 7.4 Hz, 1H), 6.16-6.14 (m, 1H), 2.46-2.43 (m, 2H), 2.26-2.22 (m, 2H), 1.84-1.79 (m, 2H), 1.72-1.67 (m, 2H); ^{13}C NMR (CDCl$_3$, 125 MHz, ppm): δ 142.7, 136.6, 128.2, 126.5, 124.9, 124.8, 27.4, 25.9, 23.1, 22.2; MS (EI) m/z: 158.1, 143.1, 129.1, 115.1.

(*E*)-1-methyl-4-styrylbenzene 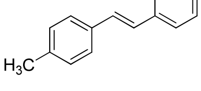 **(4o)**

White solid; mp 119.4-121.3°C. The spectroscopic data correspond to those previously reported in the literature.[11] ^{1}H NMR ($CDCl_3$, 500 MHz, ppm): δ 7.54 (d, J = 8.0 Hz, 2H), 7.45 (d, J = 8.0 Hz, 2H), 7.38 (d, J = 7.5 Hz, 2H), 7.30-7.26 (m, 1H), 7.20 (d, J = 7.9 Hz, 2H), 7.11(d, J = 3.8 Hz, 2H), 2.40 (s, 3H); ^{13}C NMR ($CDCl_3$, 125 MHz, ppm): δ 137.5, 134.6, 129.4, 128.7, 128.6, 127.7, 127.4, 126.4, 126.4, 21.3; MS (EI) m/z: 194.1, 179.1, 165.1, 152.1, 115.1.

(*E*)-1-methoxy-2-styrylbenzene 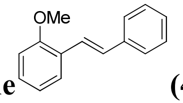

(4p)

White solid, mp 59.1-59.7°C. The spectroscopic data correspond to those previously reported in the literature.[12] ^{1}H NMR ($CDCl_3$, 500 MHz, ppm): δ 7.63 (dd, J_1 = 1.6 Hz, J_2 = 7.7 Hz, 1H), 7.57 (d, J = 7.4 Hz, 2H), 7.52 (d, J = 16.5 Hz, 1H), 7.38 (t, J = 7.5 Hz, 2H), 7.30-7.26 (m, 2H), 7.15 (d, J = 16.5 Hz, 1H), 7.00 (t, J = 7.4 Hz, 1H), 6.94 (d, J = 8.3 Hz, 1H), 3.92 (s, 3H); ^{13}C NMR ($CDCl_3$, 125 MHz, ppm): δ 156.9, 138.0, 129.1, 128.7, 128.6, 127.4, 126.6, 126.5, 126.4, 123.5, 120.8, 111.0, 55.5; MS (EI) m/z: 210.1, 165.1, 152.1, 104.1.

(*E*)-2-styrylfuran 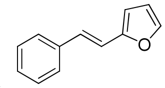

(4q)

White solid, mp. 51.4-53.2°C; The spectroscopic data correspond to those previously reported in the literature[15]. ^{1}H NMR ($CDCl_3$, 500 MHz, ppm): δ 7.50 (d, J = 7.4 Hz, 2H), 7.43 (d, J = 1.8 Hz, 1H), 7.39-7.36 (m, 2H), 7.29-7.26 (m, 1H), 7.07 (d, J = 16.3 Hz, 1H), 6.93 (d, J = 16.3 Hz, 1H); 6.46 (dd, J_1 = 1.8, J_2 =3.3 Hz, 1H), 6.38 (d, J = 3.3 Hz, 1H); ^{13}C NMR ($CDCl_3$, 125 MHz, ppm): δ 153.3, 142.1, 137.0, 128.7, 127.6, 127.1, 126.3, 116.5, 111.6, 108.6; MS (EI) m/z: 170.1, 141.1, 115.1.

(*E*)-2-styrylthiophene 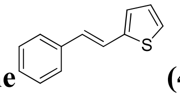

(4r)

White solid, mp. 110.2-111.6°C; The spectroscopic data correspond to those previously reported in the literature.[11a] ^{1}H NMR ($CDCl_3$, 500 MHz, ppm): δ 7.49 (d, J = 7.4 Hz, 2H), 7.37 (t, J = 7.5 Hz, 2H), 7.29-7.24 (m, 2H), 7.22 (d, J = 5.0 Hz, 1H), 7.10 (d, J = 3.1 Hz, 1H), 7.04-7.03 (m, 1H), 6.96 (d, J = 16.1 Hz, 1H); ^{13}C NMR ($CDCl_3$, 125 MHz, ppm): δ 142.9, 137.0, 128.7, 128.3, 127.6, 126.3, 126.1, 124.3, 121.8; MS (EI) m/z: 186.1, 171.1, 152.1, 115.1.

***(E)*-(2-cyclohexylvinyl)benzene** 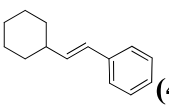 **(4s)**

Colorless oil; The spectroscopic data correspond to those previously reported in the literature.[6] ^{1}H NMR ($CDCl_3$, 500 MHz, ppm): δ 7.37 (d, J = 7.3 Hz, 2H), 7.31 (d, J = 7.5 Hz, 2H), 7.21 (tt, J_1 = 1.4 Hz, J_2 = 7.4 Hz, 1H), 6.36 (d, J = 16.0 Hz, 1H), 6.20 (dd, J_1 = 6.9 Hz, J_2 = 16.0 Hz, 1H), 2.19-2.12 (m, 1H), 1.85-1.77 (m, 4H), 1.73-1.69 (m, 1H), 1.39-1.30 (m, 2H), 1.27-1.17 (m, 3H); ^{13}C NMR ($CDCl_3$, 125 MHz, ppm): δ 138.1, 136.9, 128.5, 127.2, 126.7, 125.9, 41.2, 33.0, 26.2, 26.1; MS (EI) m/z: 186.2, 171.2, 129.2, 104.2, 91.2.

References

[1] (a) C-F. Yao , C-M. Chu , J-T. Liu, *J. Org. Chem.*, **1998**, *63*, 719–722; (b) X. Zhao, J. Jing, K. Lu, Y. Zhang, J. Wang, *Chem. Commun.*, **2010**, *46*, 1724–1726.

[2] J. Barluenga, P. Moriel, C. Valdés, F. Aznar, *Angew. Chem. Int. Ed.* **2007**, *46*, 5587-5590.

[3] (a) A. B. Smith, III, A. T. Hoye, D. Martinez-Solorio, W.-S. Kim, R. Tong, *J. Am. Chem. Soc.* **2012**, *134*, 4533-4536; (b) N. Tsukada, T. Mitsuboshi, H. Setoguchi, Y. Inoue, *J. Am. Chem. Soc.* **2003**, *125*, 12102.

[4] N. Sakai, T. Moriya, T. Konakahara, *J. Org. Chem.* **2007**, *72*, 5920-5922.

[5] J. Li, Q. Liu, H. Shen, R. Huang, X. Zhang, Y. Xiong, C. Chen, *RSC Adv.*, **2015**, *5*, 85291.

[6] S-W. Wu, J-L. Liu, F. Liu, *Org. Lett.* **2016**, *18*, 1−3.

[7] (a) C. Ha, J. H. Horner, M. Newcomb, T. R. Varick, B. R. Arnold, J. Lusztyk, *J. Org. Chem.* **1993**, *58*, 1194-1198; (b) H. Zeng, R. Hua, *J. Org. Chem.* **2008**, *73*, 558-562.

[8] J. Terao, A. Ikumi, H. Kuniyasu, N. Kambe, *J. Am. Chem. Soc.,* **2003**, *125*, 5646–5647.

[9] T. Miao, D. Xia, Y. Li, P. Lia, L. Wang, *Chem. Commun*., **2016**, *52*, 3175–3178.

[10] (a) F. Xue, J. Zhao, T. S. A. Hor, *Chem. Commun.*, **2013**, *49*, 10121; (b) J-Z. Zhang, Y. Tang, *Adv. Synth. Catal*. **2016**, *358*, 752-764.

[11] (a) S. Fu, N-Y. Chen, X. Liu, Z. Shao, S-P. Luo, Q. Liu, *J. Am. Chem. Soc.,* **2016**, *138*, 8588-8594; (b) B. Shrestha, S. Thapa, S. K. Gurung, R. A. S. Pike, R. Giri, *J. Org. Chem.* **2016**, *81*, 787-802.

[12] J. S. Foot, G. M. P. Giblin, A. C. Whitwooda, R. J. K. Taylor, *Org. Biomol. Chem.* **2005**, *3*, 756-763.

[13] S. Keesara, S. Parvathaneni, M. R. Mandapati, *Tetrahedron Lett* **201**4, *55*, 6769–6772.

[14] R. Martí-Centelles, R. Cejudo-Marín, E. Falomir, J. Murga, M. Carda, J. A. Marco, *Bioorg Med Chem*, **2013**, 21, 3010–3015.

[15] M. V. Sorokina, A. S. Pankova, M. A. Kuznetsov, *Asian J. Org. Chem.* **2016**, *5*, 389 -398.

[16] S. Kindt, K. Wicht, M. R. Heinrich, *Angew. Chem. Int. Ed.* **2016**, *55*, 8744 –8747.

[17] D. Eom, S. Park, Y. Park, K. Lee, G. Hong, P. H. Lee, *Eur. J. Org. Chem.* **2013**, 2672-2682.

7. Copies of NMR Spectra for 3a-4s

3a

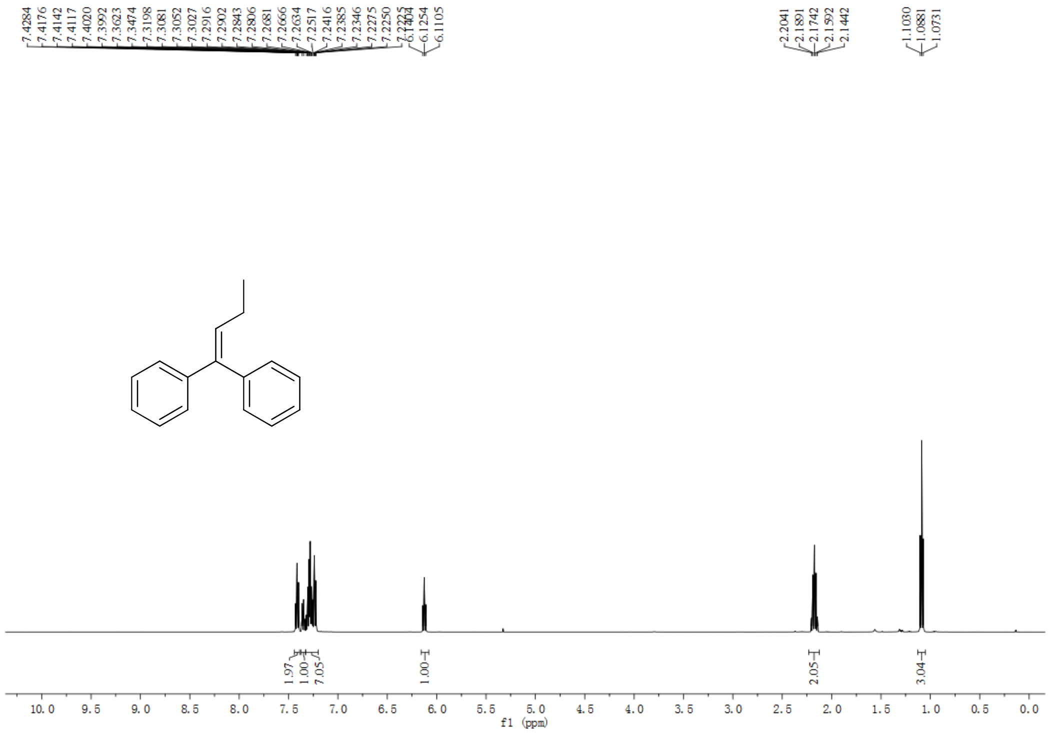

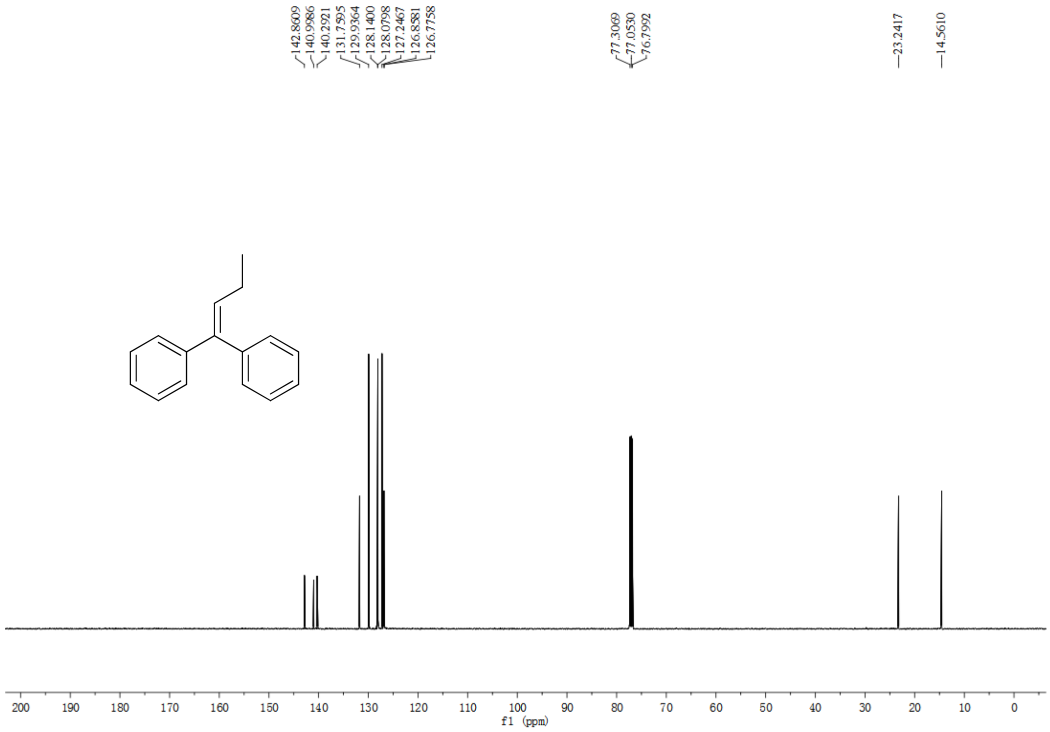

3b

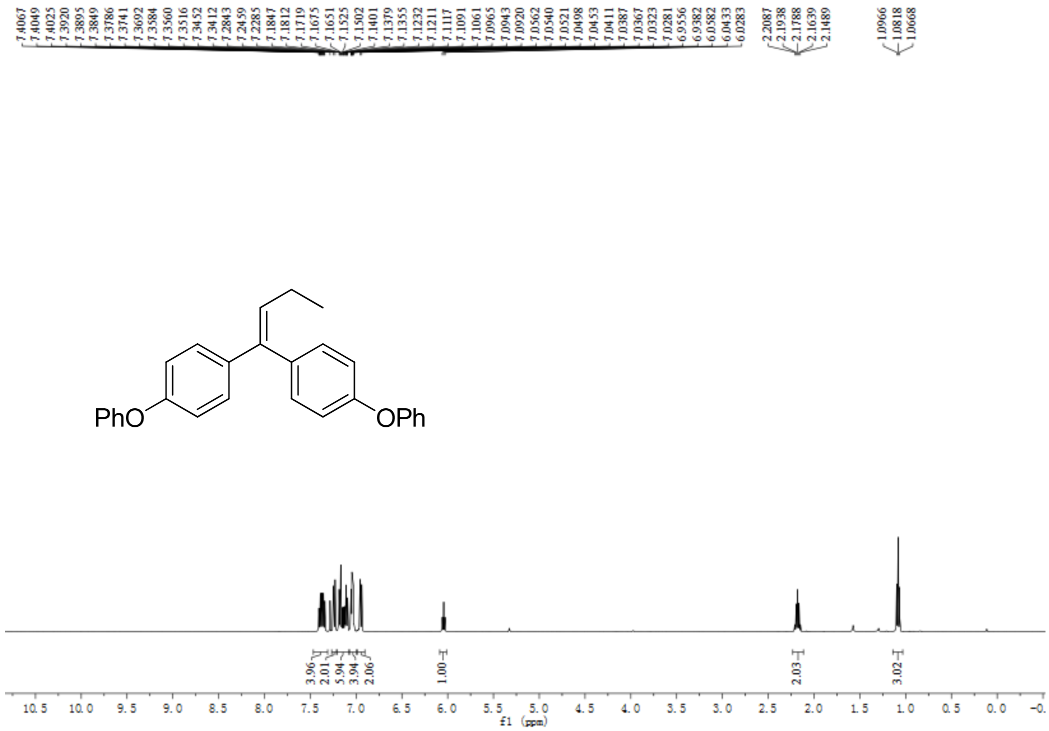
7.4067
7.4049
7.4025
7.3920
7.3895
7.3849
7.3786
7.3741
7.3692
7.3584
7.3560
7.3516
7.3452
7.3412
7.2843
7.2459
7.2285
7.1847
7.1812
7.1719
7.1675
7.1651
7.1525
7.1502
7.1401
7.1379
7.1355
7.1232
7.1211
7.1117
7.1091
7.1061
7.0965
7.0943
7.0920
7.0562
7.0540
7.0521
7.0498
7.0453
7.0411
7.0387
7.0367
7.0323
7.0281
6.9556
6.9382
6.0582
6.0433
6.0283
2.2087
2.1938
2.1788
2.1639
2.1489
1.0966
1.0818
1.0668
PhO
OPh
3.96
2.01
5.94
3.94
2.06
1.00
2.03
3.02
f1 (ppm)

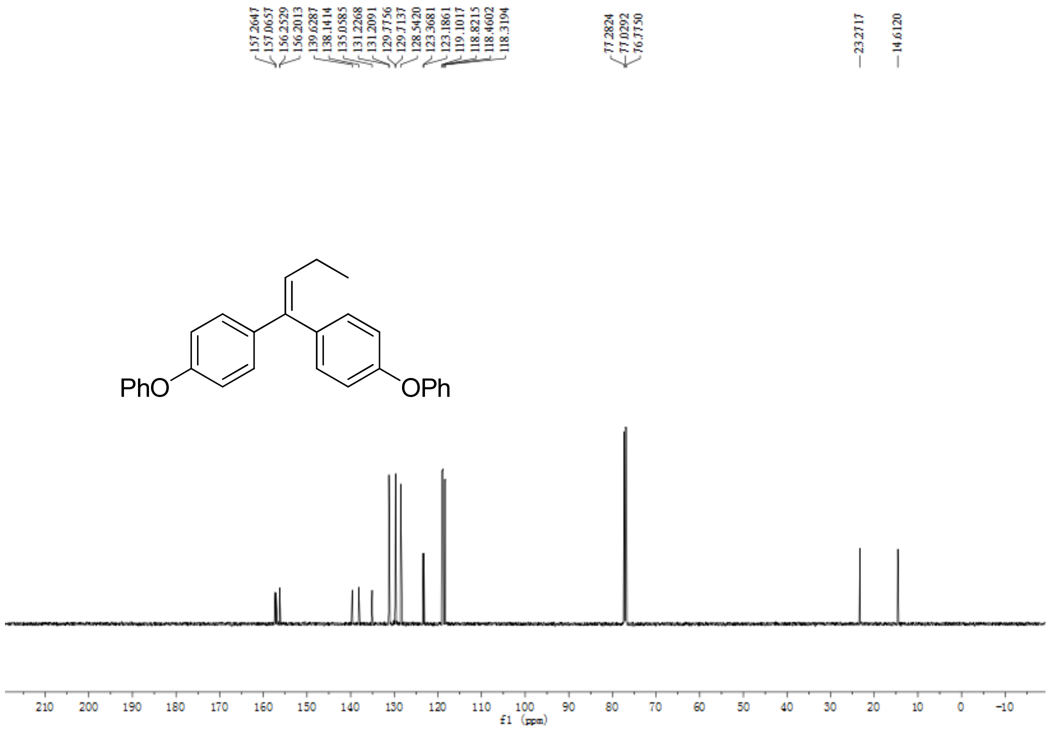
157.2647
157.0657
156.2529
156.2013
139.6287
138.1414
135.0585
131.2268
131.2091
129.7756
129.7137
128.5420
123.3681
123.1861
119.1017
118.8215
118.4602
118.3194
77.2824
77.0292
76.7750
23.2717
14.6120
PhO
OPh
f1 (ppm)

3c

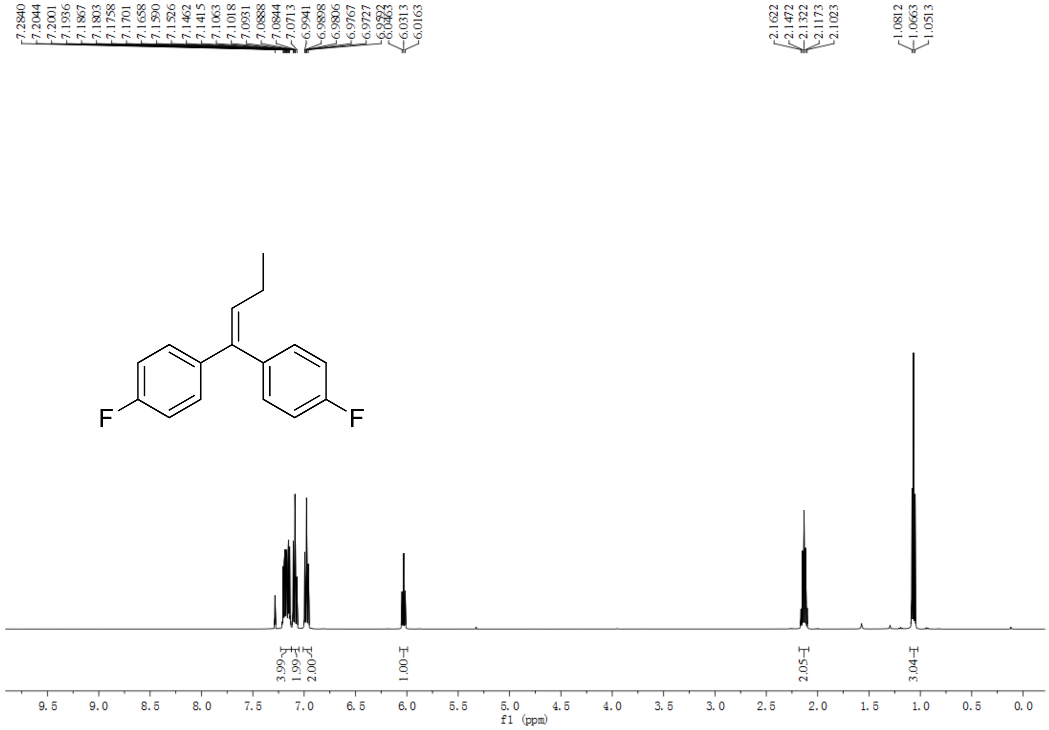
7.2840
7.2044
7.2001
7.1936
7.1867
7.1803
7.1758
7.1701
7.1658
7.1590
7.1526
7.1462
7.1415
7.1063
7.1018
7.0931
7.0888
7.0844
7.0713
6.9941
6.9898
6.9806
6.9767
6.9727
6.0313
6.0163
2.1622
2.1472
2.1322
2.1173
2.1023
1.0812
1.0663
1.0513
F
F
3.99
1.99
2.00
1.00
2.05
3.04
f1 (ppm)

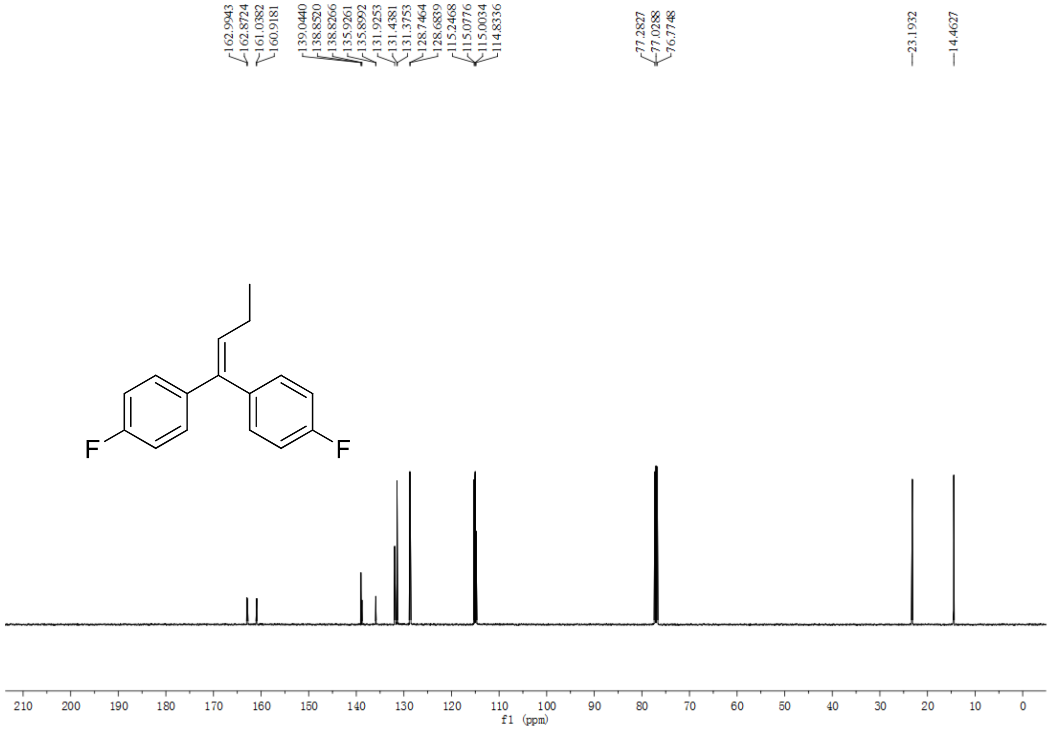
162.9943
162.8724
161.0382
160.9181
139.0440
138.8520
138.8266
135.9261
135.8992
131.9253
131.4381
131.3753
128.7464
128.6839
115.2468
115.0776
115.0034
114.8336
77.2827
77.0288
76.7748
23.1932
14.4627
F
F
f1 (ppm)

3d

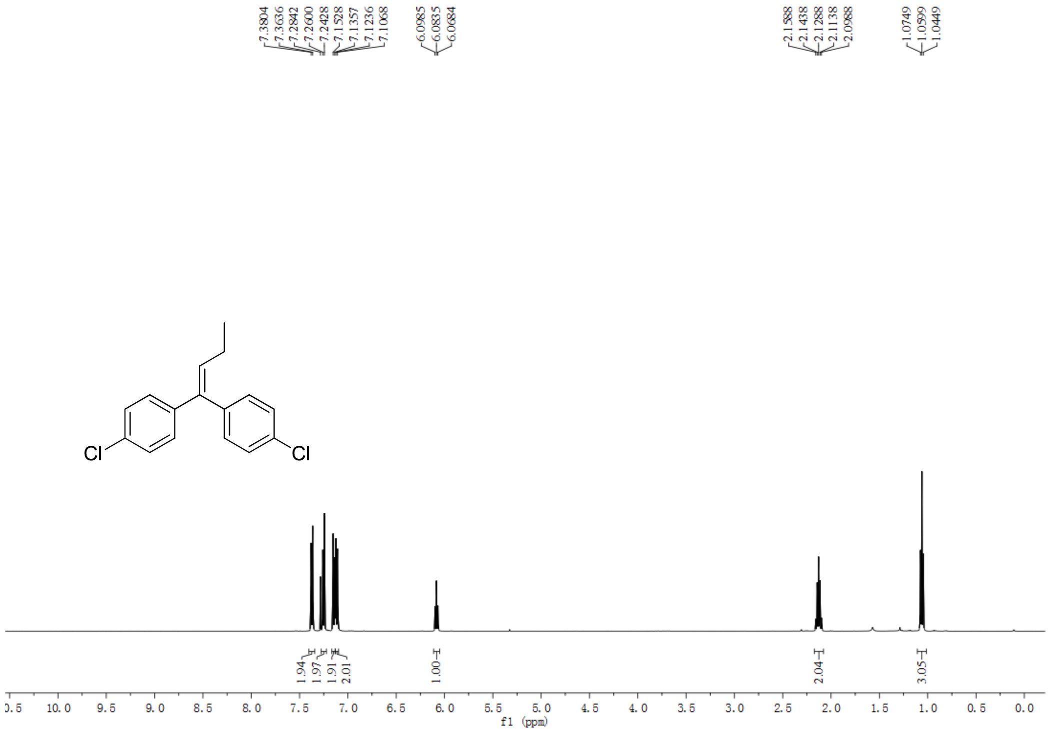
7.3804
7.3636
7.2842
7.2600
7.2428
7.1528
7.1357
7.1236
7.1068
6.0985
6.0835
6.0684
2.1588
2.1438
2.1288
2.1138
2.0988
1.0749
1.0599
1.0449
Cl
Cl
1.94
1.97
1.91
2.01
1.00
2.04
3.05
f1 (ppm)

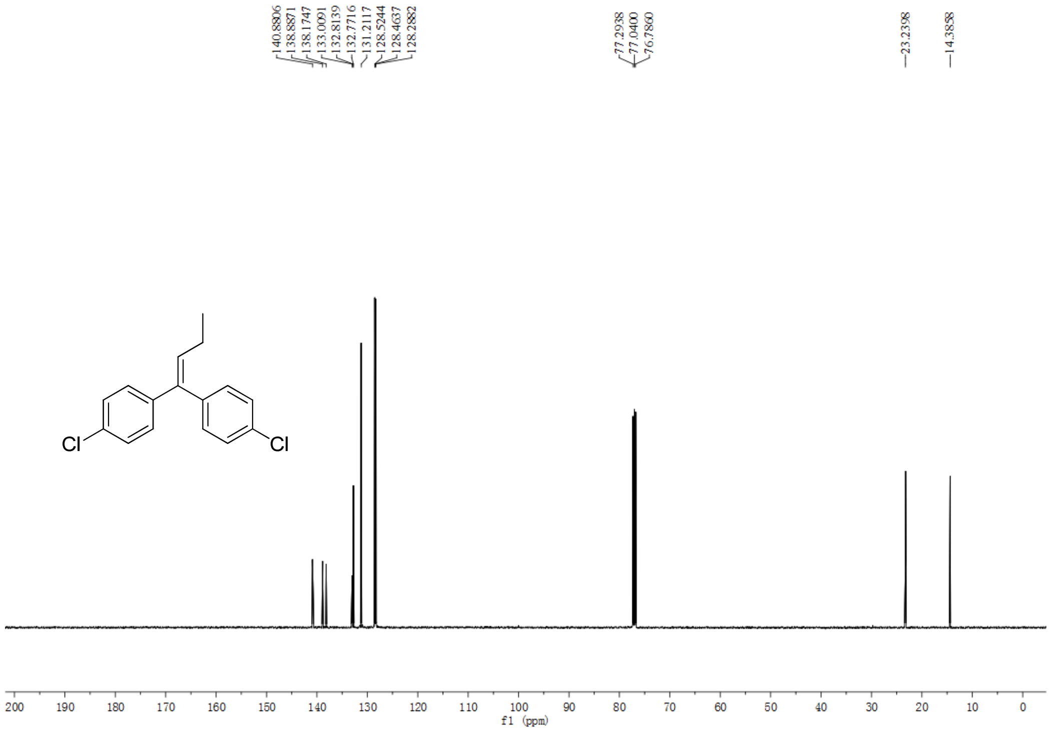
140.8806
138.8871
138.1747
133.0091
132.8139
132.7716
131.2117
128.5244
128.4637
128.2882
77.2938
77.0400
76.7860
23.2398
14.3858
Cl
Cl
f1 (ppm)

3e (*Z*/*E*)

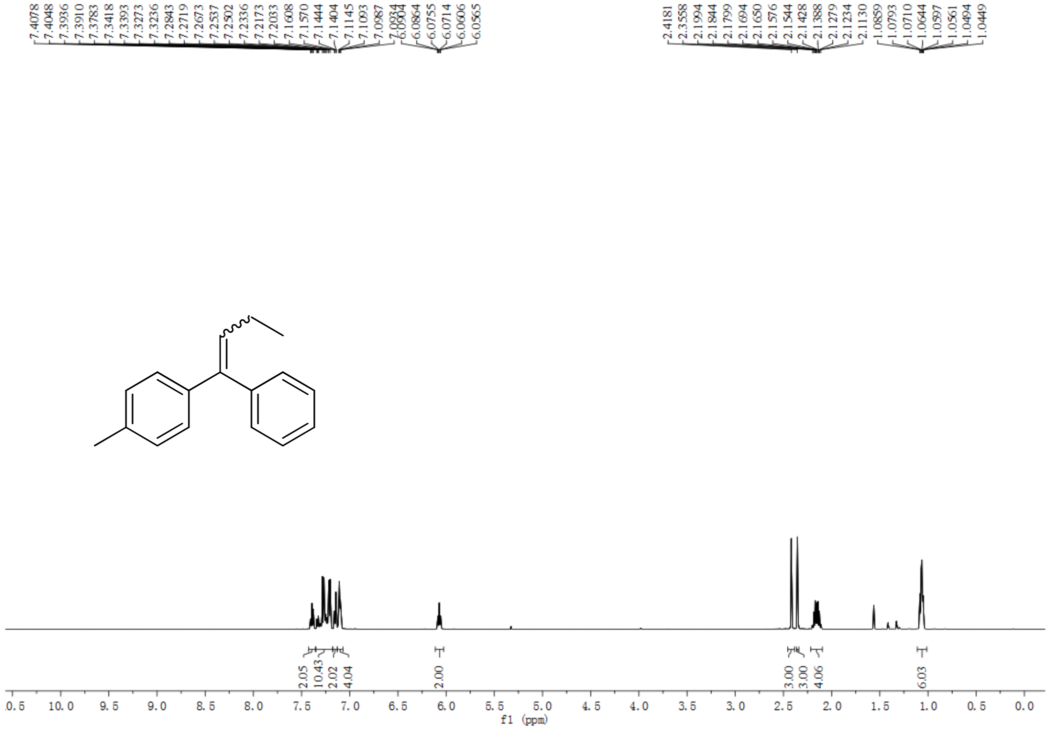
7.4078
7.4048
7.3936
7.3910
7.3783
7.3418
7.3393
7.3273
7.3236
7.2843
7.2719
7.2673
7.2537
7.2502
7.2336
7.2173
7.2033
7.1608
7.1570
7.1444
7.1404
7.1145
7.1093
7.0987
7.0934
6.0904
6.0864
6.0755
6.0714
6.0606
6.0565
2.4181
2.3558
2.1994
2.1844
2.1799
2.1694
2.1650
2.1576
2.1544
2.1428
2.1388
2.1279
2.1234
2.1130
1.0859
1.0793
1.0710
1.0644
1.0597
1.0561
1.0494
1.0449
2.05
10.43
2.02
4.04
2.00
3.00
3.00
4.06
6.03
f1 (ppm)

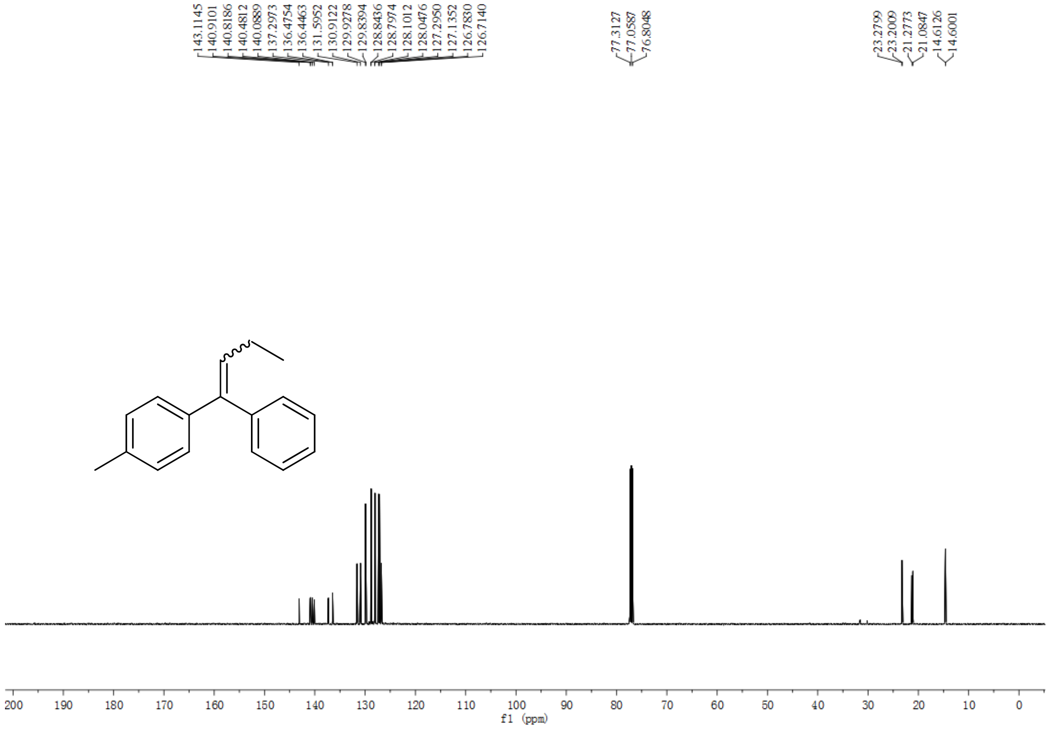
143.1145
140.9101
140.8186
140.4812
140.0889
137.2973
136.4754
136.4463
131.5952
130.9122
129.9278
129.8394
128.8436
128.7974
128.1012
128.0476
127.2950
127.1352
126.7830
126.7140
77.3127
77.0587
76.8048
23.2799
23.2009
21.2773
21.0847
14.6126
14.6001
f1 (ppm)

3f (*Z*/*E*)

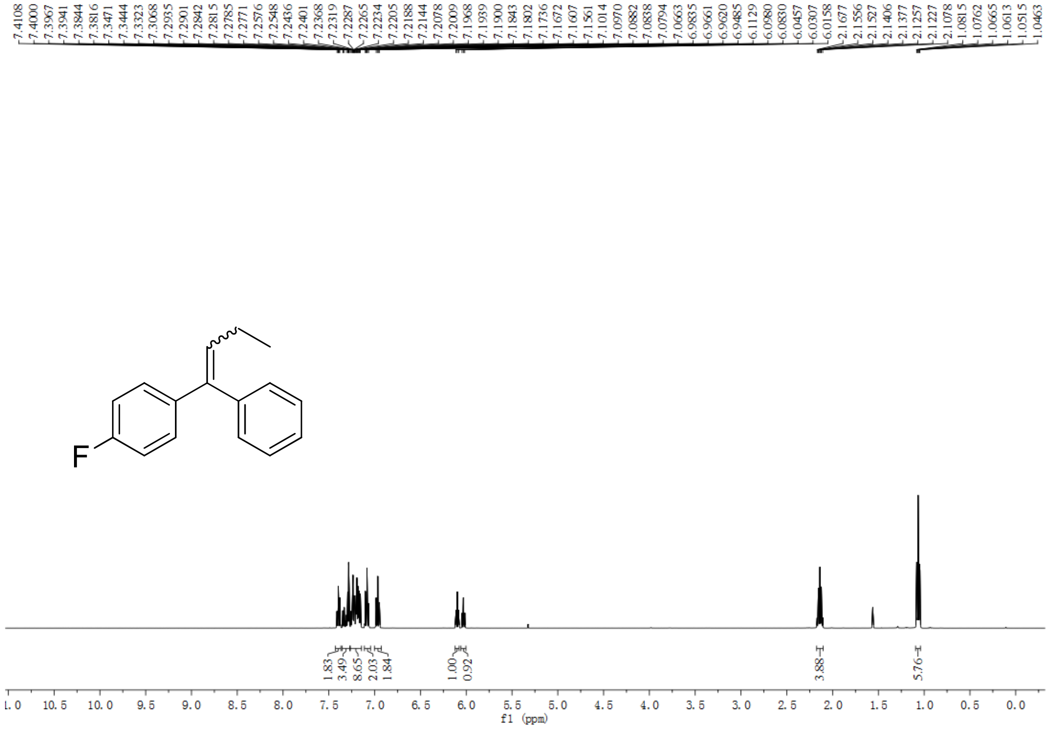
7.4108
7.4000
7.3967
7.3941
7.3844
7.3816
7.3471
7.3444
7.3323
7.3068
7.2935
7.2901
7.2842
7.2815
7.2785
7.2771
7.2576
7.2548
7.2436
7.2401
7.2368
7.2319
7.2287
7.2265
7.2234
7.2205
7.2188
7.2144
7.2078
7.2009
7.1968
7.1939
7.1900
7.1843
7.1802
7.1736
7.1672
7.1607
7.1561
7.1014
7.0970
7.0882
7.0838
7.0794
7.0663
6.9835
6.9661
6.9620
6.9485
6.1129
6.0980
6.0830
6.0457
6.0307
6.0158
2.1677
2.1556
2.1527
2.1406
2.1377
2.1257
2.1227
2.1078
1.0815
1.0762
1.0665
1.0613
1.0515
1.0463
F
1.83
3.49
8.65
2.03
1.84
1.00
0.92
3.88
5.76
f1 (ppm)

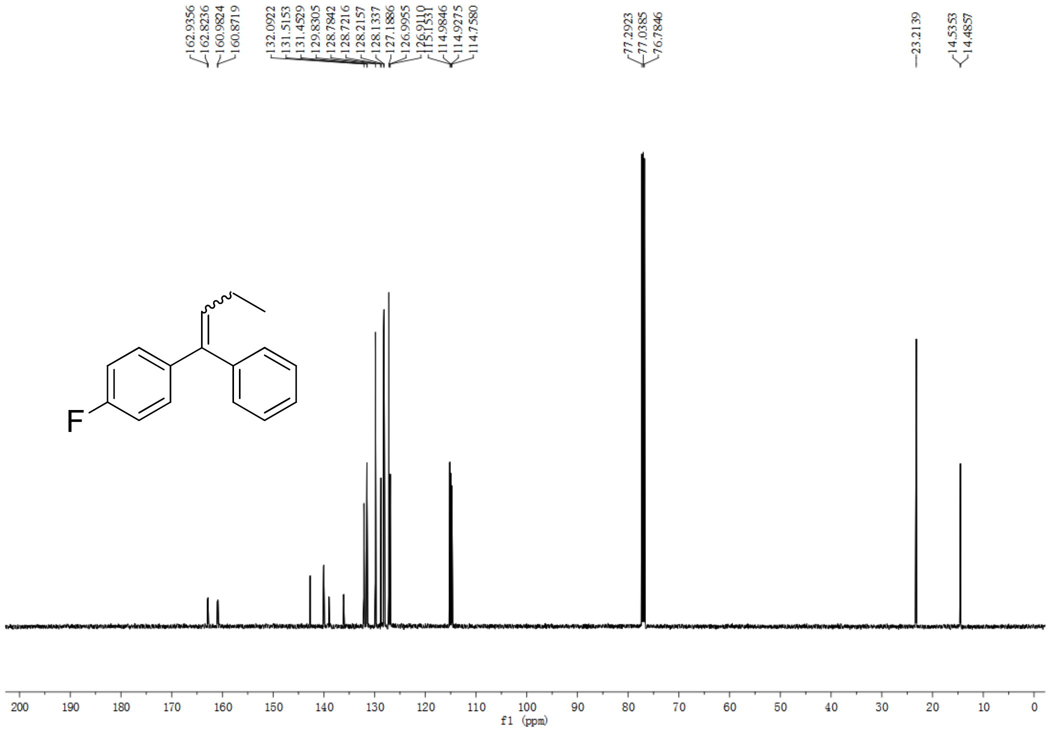
162.9356
162.8236
160.9824
160.8719
132.0922
131.5153
131.4529
129.8305
128.7842
128.7216
128.2157
128.1337
127.1886
126.9955
126.9110
115.1531
114.9846
114.9275
114.7580
77.2923
77.0385
76.7846
23.2139
14.5353
14.4857
F
f1 (ppm)

3g (*Z/E*)

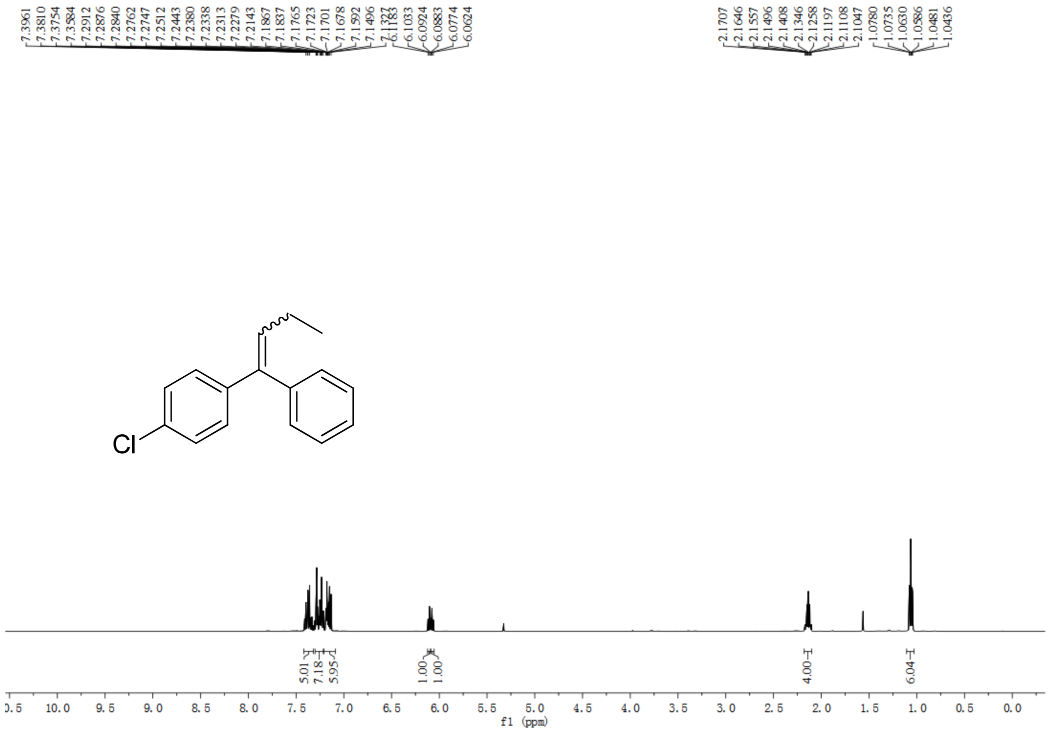

7.3961
7.3810
7.3754
7.3584
7.2912
7.2876
7.2840
7.2762
7.2747
7.2512
7.2443
7.2380
7.2338
7.2313
7.2279
7.2143
7.1867
7.1837
7.1765
7.1723
7.1701
7.1678
7.1592
7.1496
7.1327
6.1183
6.1033
6.0924
6.0883
6.0774
6.0624
2.1707
2.1646
2.1557
2.1496
2.1408
2.1346
2.1258
2.1197
2.1108
2.1047
1.0780
1.0735
1.0630
1.0586
1.0481
1.0436
Cl
5.01
7.18
5.95
1.00
1.00
4.00
6.04
f1 (ppm)

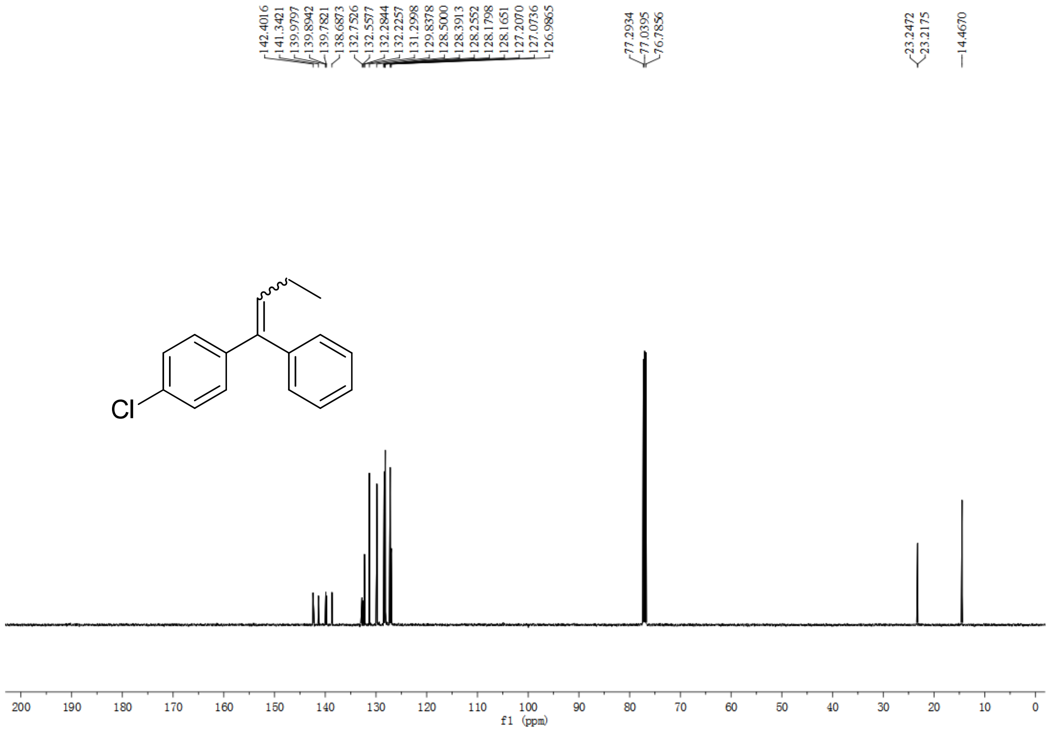

142.4016
141.3421
139.9797
139.8942
139.7821
138.6873
132.7526
132.5577
132.2844
132.2257
131.2998
129.8378
128.5000
128.3913
128.2552
128.1798
128.1651
127.2070
127.0736
126.9865
77.2934
77.0395
76.7856
23.2472
23.2175
14.4670
Cl
f1 (ppm)

3h (*Z/E*)

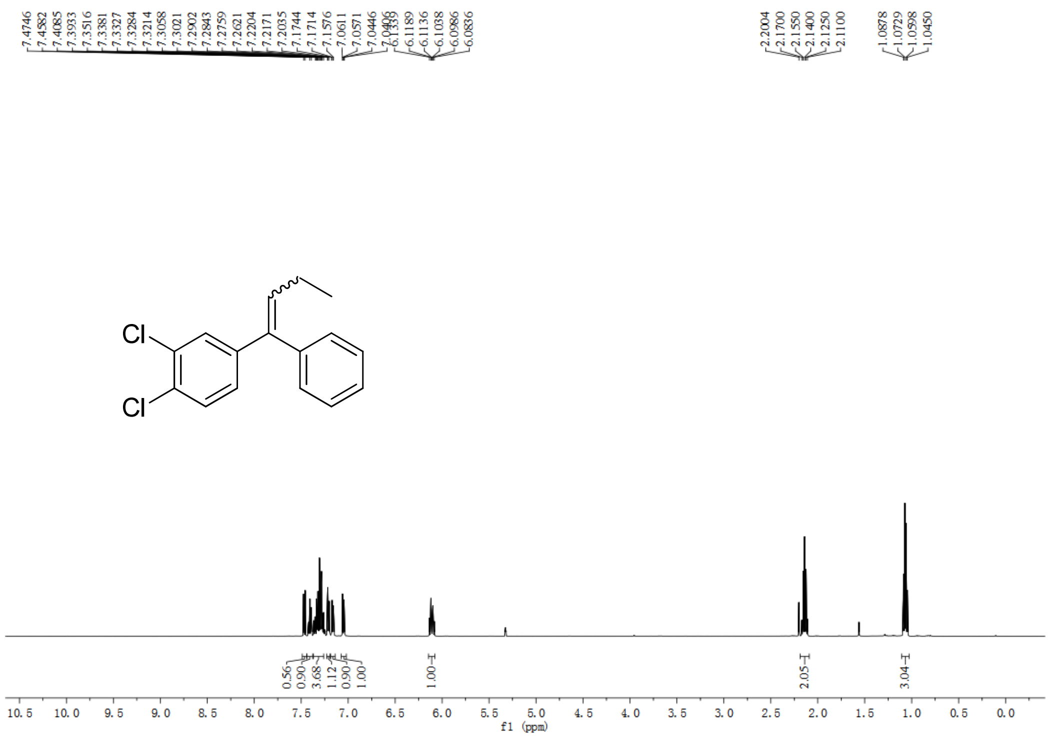
7.4746
7.4582
7.4085
7.3933
7.3516
7.3381
7.3327
7.3284
7.3214
7.3058
7.3021
7.2902
7.2843
7.2759
7.2621
7.2204
7.2171
7.2035
7.1744
7.1714
7.1576
7.0611
7.0571
7.0446
6.1189
6.1136
6.1038
6.0986
6.0836
2.2004
2.1700
2.1550
2.1400
2.1250
2.1100
1.0878
1.0729
1.0598
1.0450
Cl
Cl
0.56
0.90
3.68
1.12
0.90
1.00
1.00
2.05
3.04
f1 (ppm)

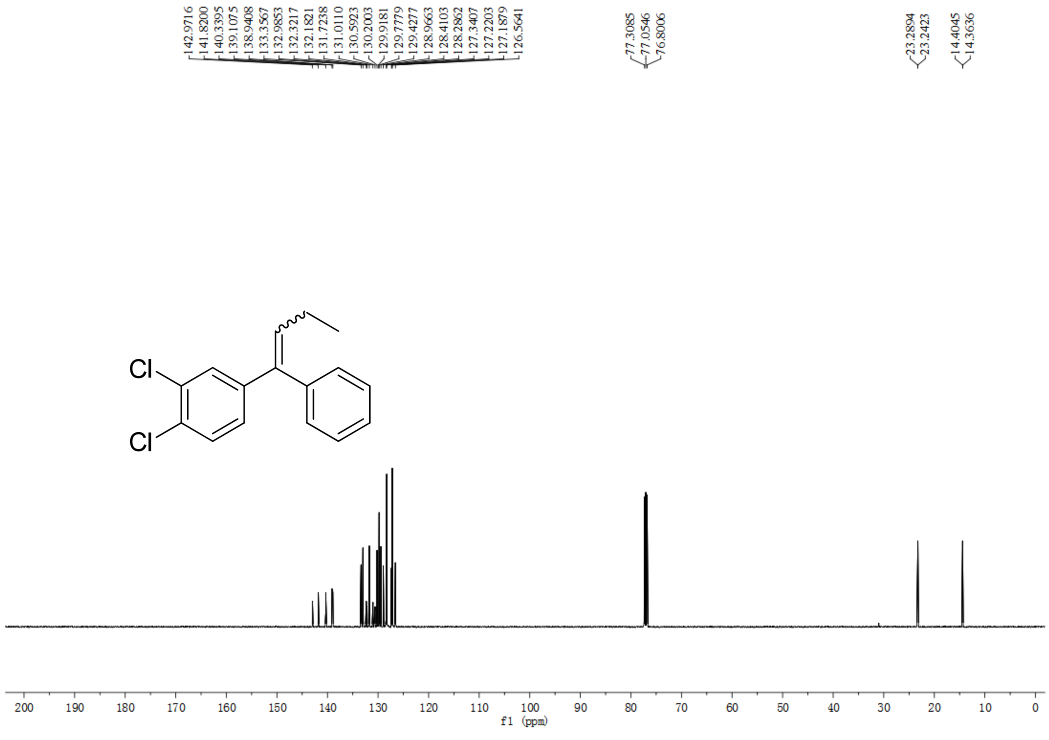
142.9716
141.8200
140.3395
139.1075
138.9408
133.3567
132.9853
132.3217
132.1821
131.7238
131.0110
130.5923
130.2003
129.9181
129.7779
129.4277
128.9663
128.4103
128.2862
127.3407
127.2203
127.1879
126.5641
77.3085
77.0546
76.8006
23.2894
23.2423
14.4045
14.3636
Cl
Cl
f1 (ppm)

3i(*E*)

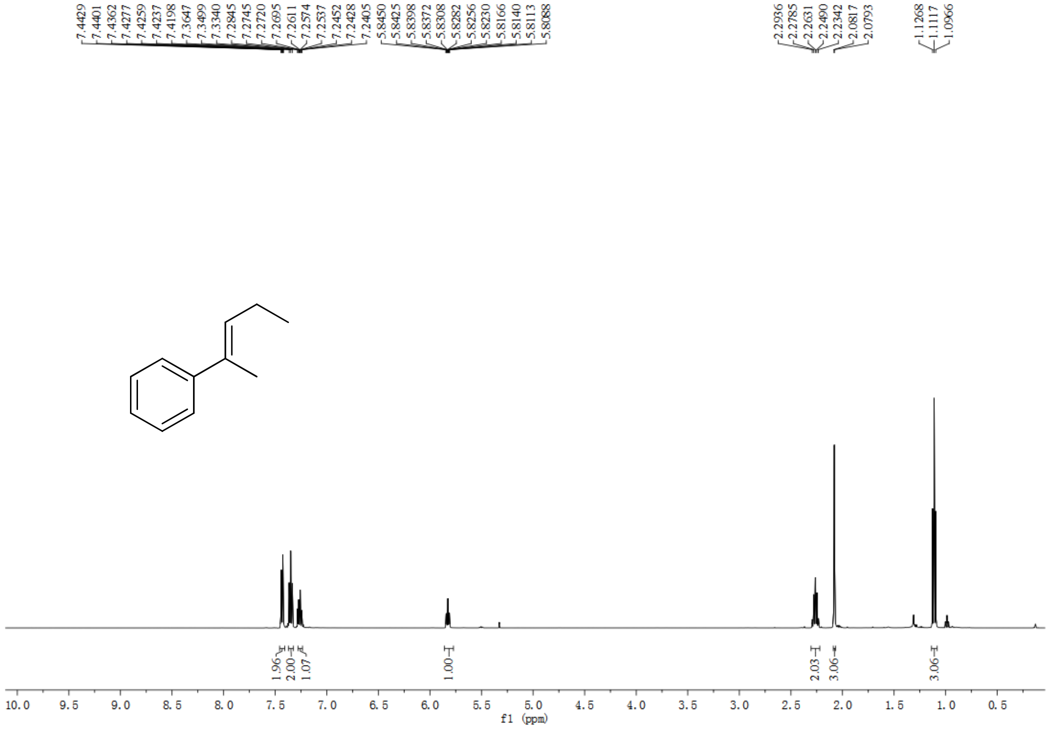
7.4429
7.4401
7.4362
7.4277
7.4259
7.4237
7.4198
7.3647
7.3499
7.3340
7.2845
7.2745
7.2720
7.2695
7.2611
7.2574
7.2537
7.2452
7.2428
7.2405
5.8450
5.8425
5.8398
5.8372
5.8308
5.8282
5.8256
5.8230
5.8166
5.8140
5.8113
5.8088
2.2936
2.2785
2.2631
2.2490
2.2342
2.0817
2.0793
1.1268
1.1117
1.0966
1.96
2.00
1.07
1.00
2.03
3.06
3.06
f1 (ppm)

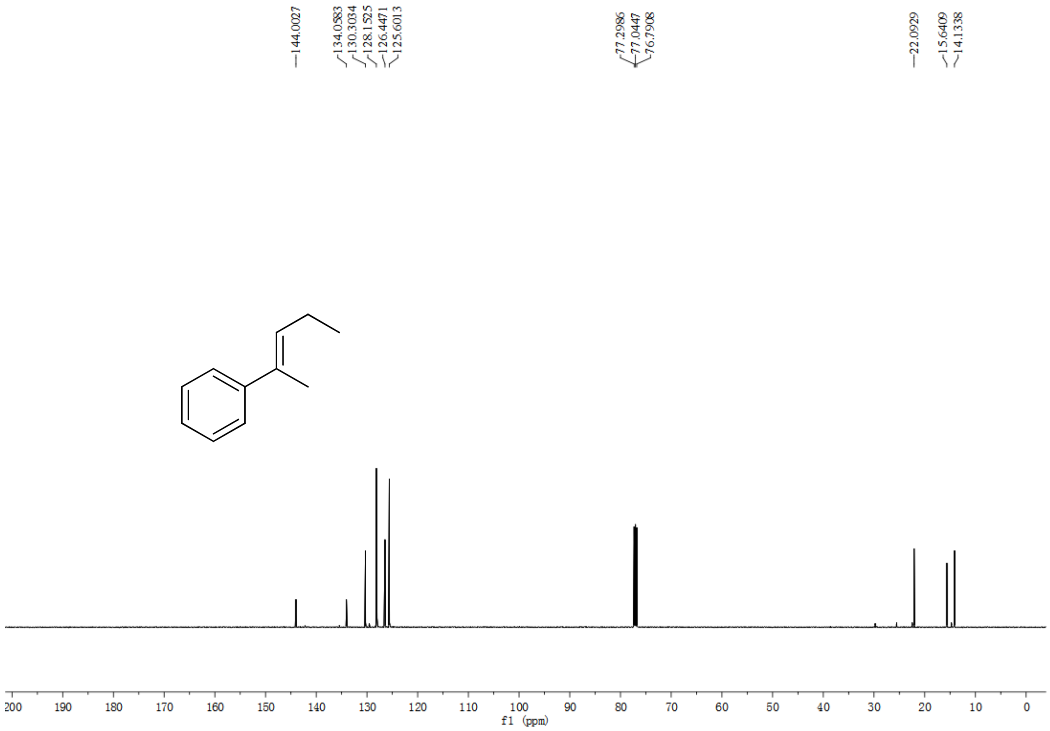
144.0027
134.0583
130.3034
128.1525
126.4471
125.6013
77.2986
77.0447
76.7908
22.0929
15.6409
14.1338
f1 (ppm)

3i(*Z*)

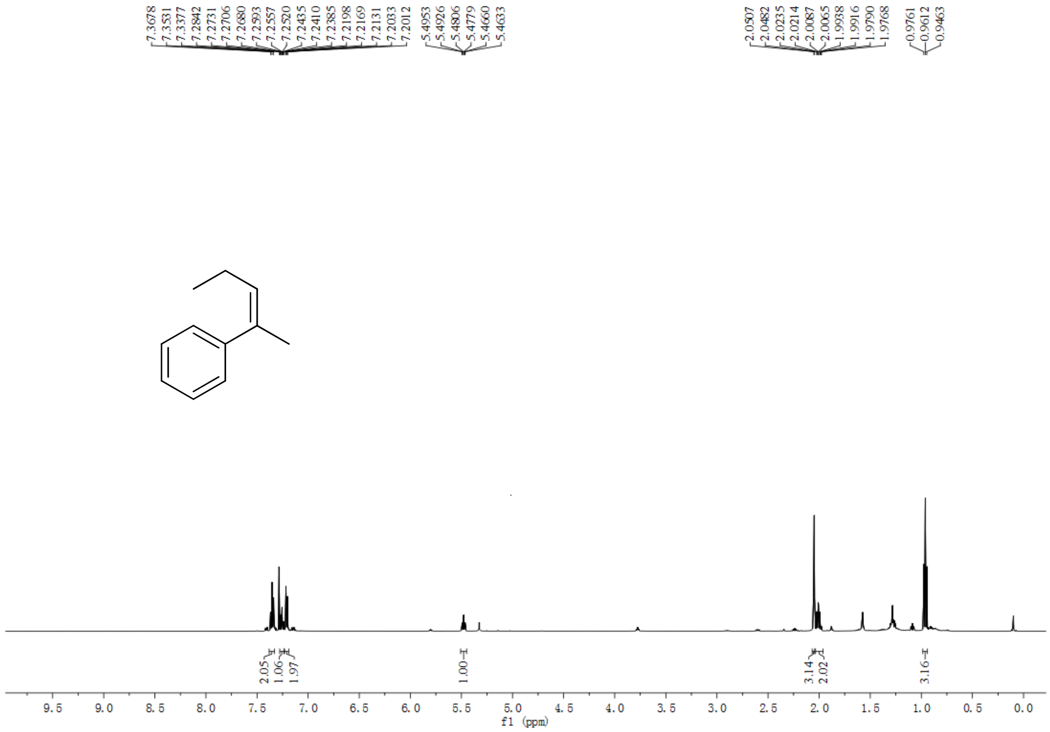
7.3678
7.3531
7.3377
7.2842
7.2731
7.2706
7.2680
7.2593
7.2557
7.2520
7.2435
7.2410
7.2385
7.2198
7.2169
7.2131
7.2033
7.2012
5.4953
5.4926
5.4806
5.4779
5.4660
5.4633
2.0507
2.0482
2.0235
2.0214
2.0087
2.0065
1.9938
1.9916
1.9790
1.9768
0.9761
0.9612
0.9463
2.05
1.06
1.97
1.00
3.14
2.02
3.16
f1 (ppm)

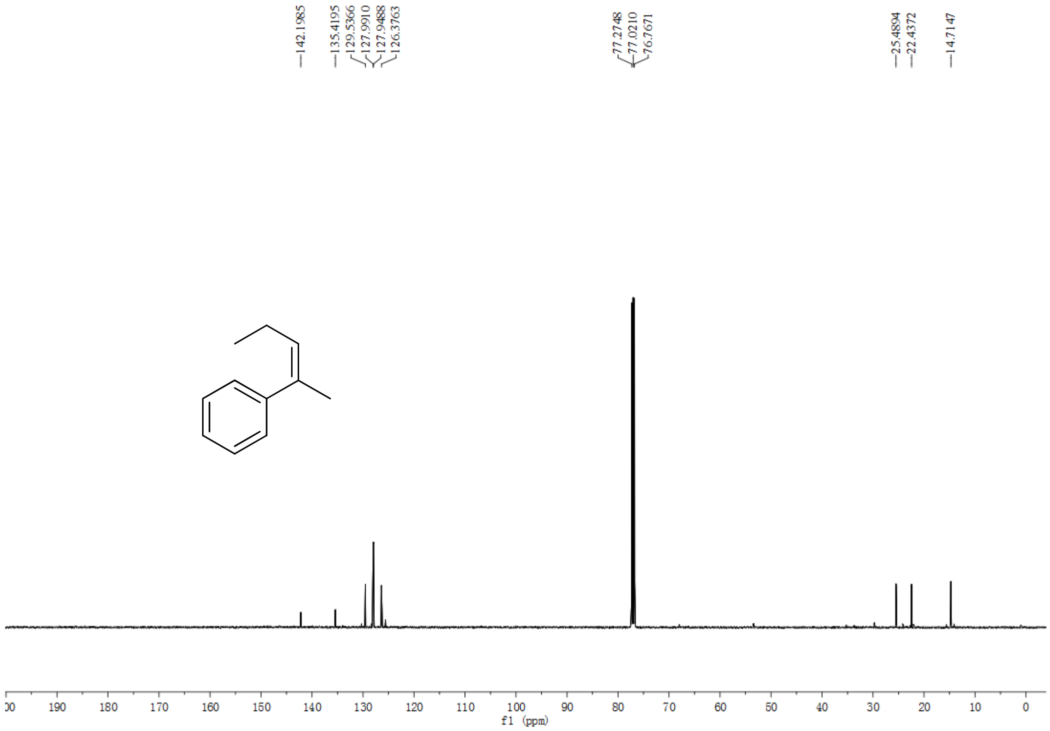
142.1985
135.4195
129.5366
127.9910
127.9488
126.3763
77.2748
77.0210
76.7671
25.4894
22.4372
14.7147
f1 (ppm)

3ia(*E*)

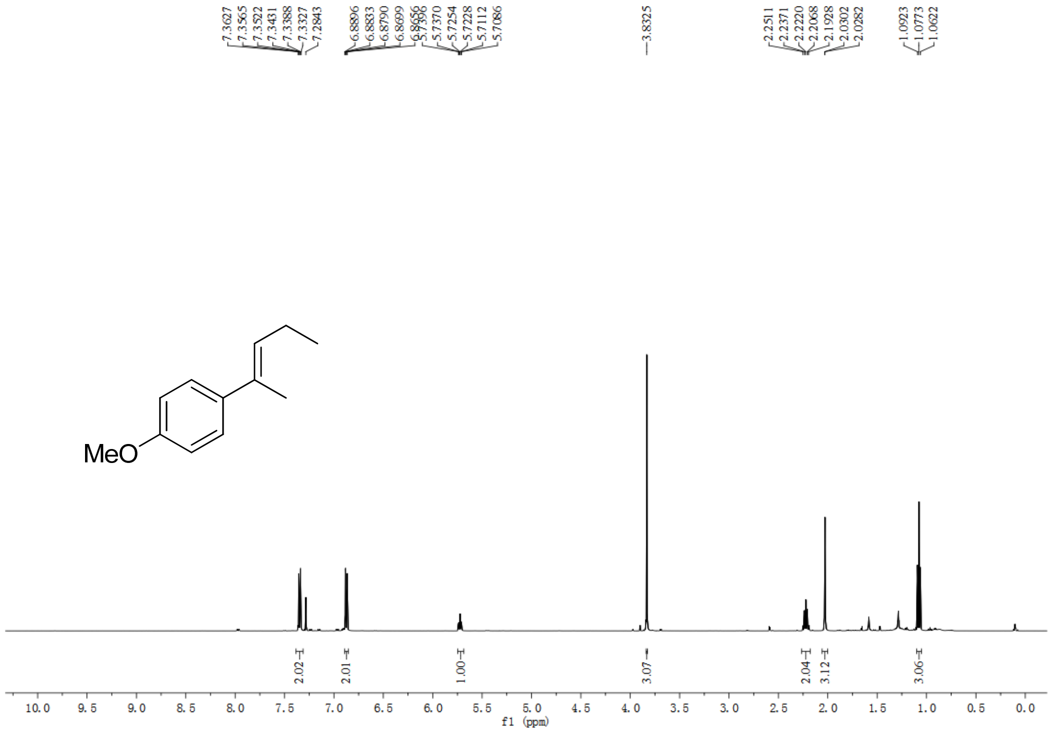

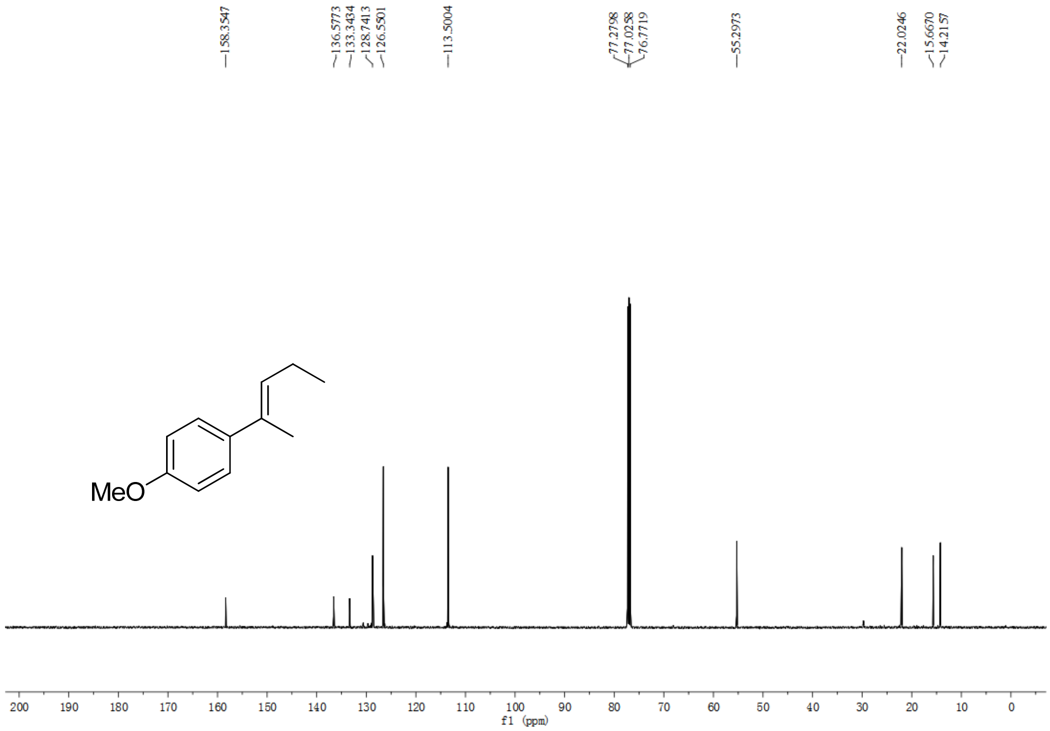

3ia(*Z*)

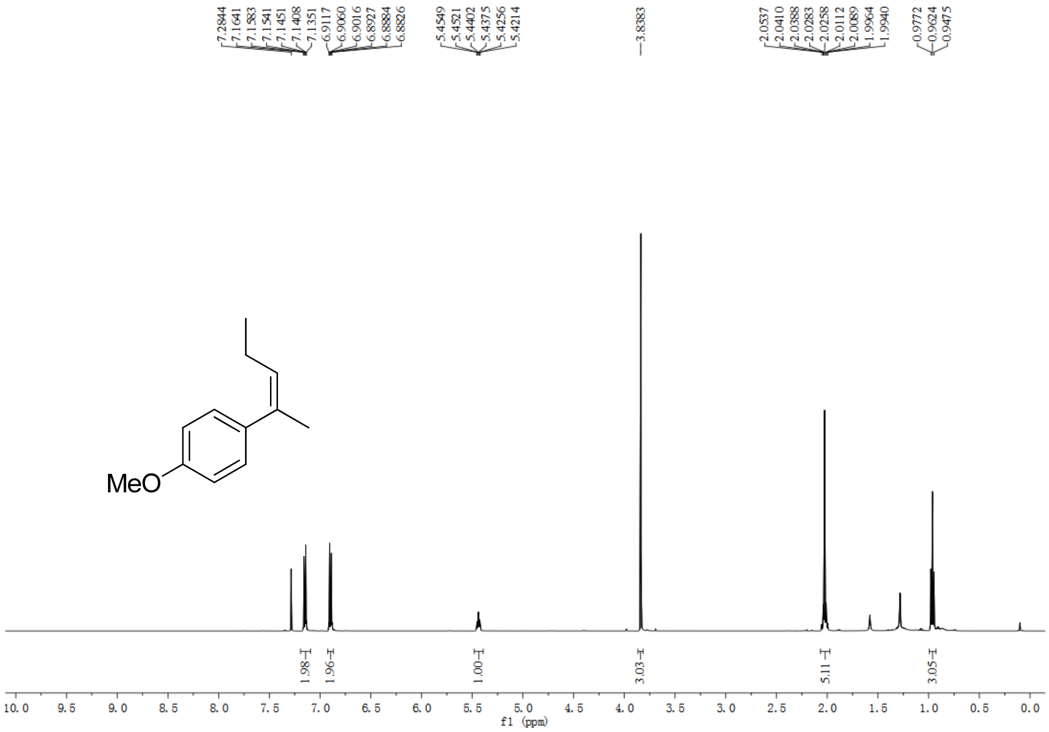

7.2844
7.1641
7.1583
7.1541
7.1451
7.1408
7.1351
6.9117
6.9060
6.9016
6.8927
6.8884
6.8826
5.4549
5.4521
5.4402
5.4375
5.4256
5.4214
3.8383
2.0537
2.0410
2.0388
2.0283
2.0258
2.0112
2.0089
1.9964
1.9940
0.9772
0.9624
0.9475
MeO
1.98
1.96
1.00
3.03
5.11
3.05
f1 (ppm)

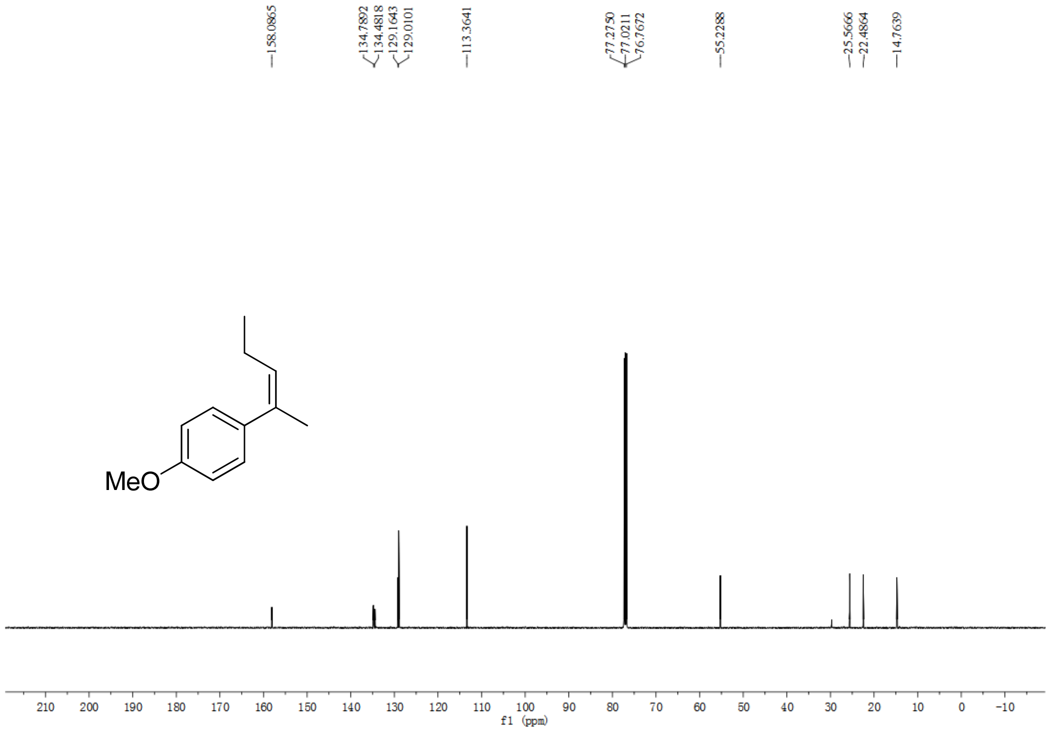

158.0865
134.7892
134.4818
129.1643
129.0101
113.3641
77.2750
77.0211
76.7672
55.2288
25.5666
22.4864
14.7639
MeO
f1 (ppm)

3ib(*E*)

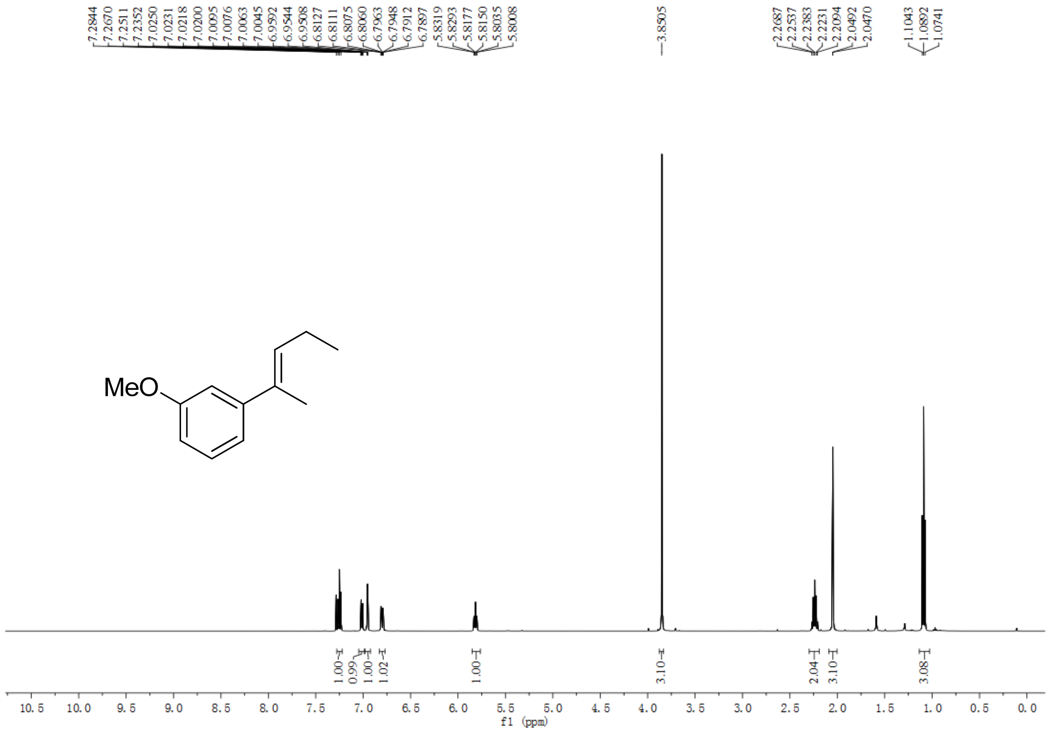
7.2844
7.2670
7.2511
7.2352
7.0250
7.0231
7.0218
7.0200
7.0095
7.0076
7.0063
7.0045
6.9592
6.9544
6.9508
6.8127
6.8111
6.8075
6.8060
6.7963
6.7948
6.7912
6.7897
5.8319
5.8293
5.8177
5.8150
5.8035
5.8008
3.8505
2.2687
2.2537
2.2383
2.2231
2.2094
2.0492
2.0470
1.1043
1.0892
1.0741
MeO
1.00
0.99
1.00
1.02
1.00
3.10
2.04
3.10
3.08
f1 (ppm)

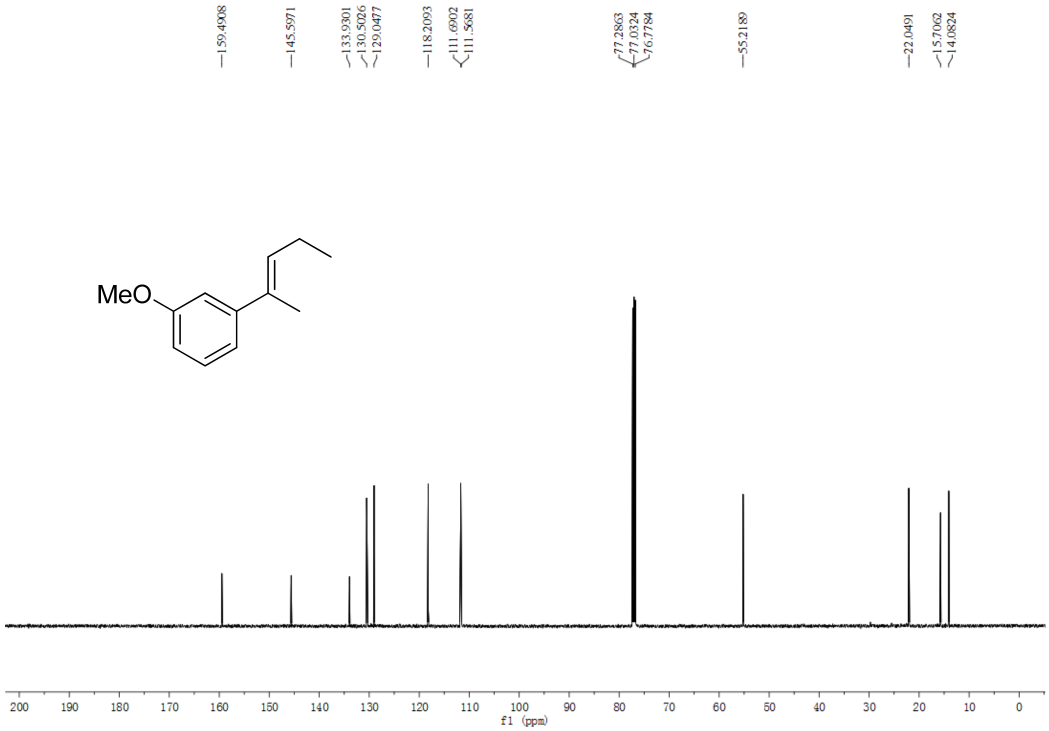
159.4908
145.5971
133.9301
130.5026
129.0477
118.2093
111.6902
111.5681
77.2863
77.0324
76.7784
55.2189
22.0491
15.7062
14.0824
MeO
f1 (ppm)

3ib(*Z*)

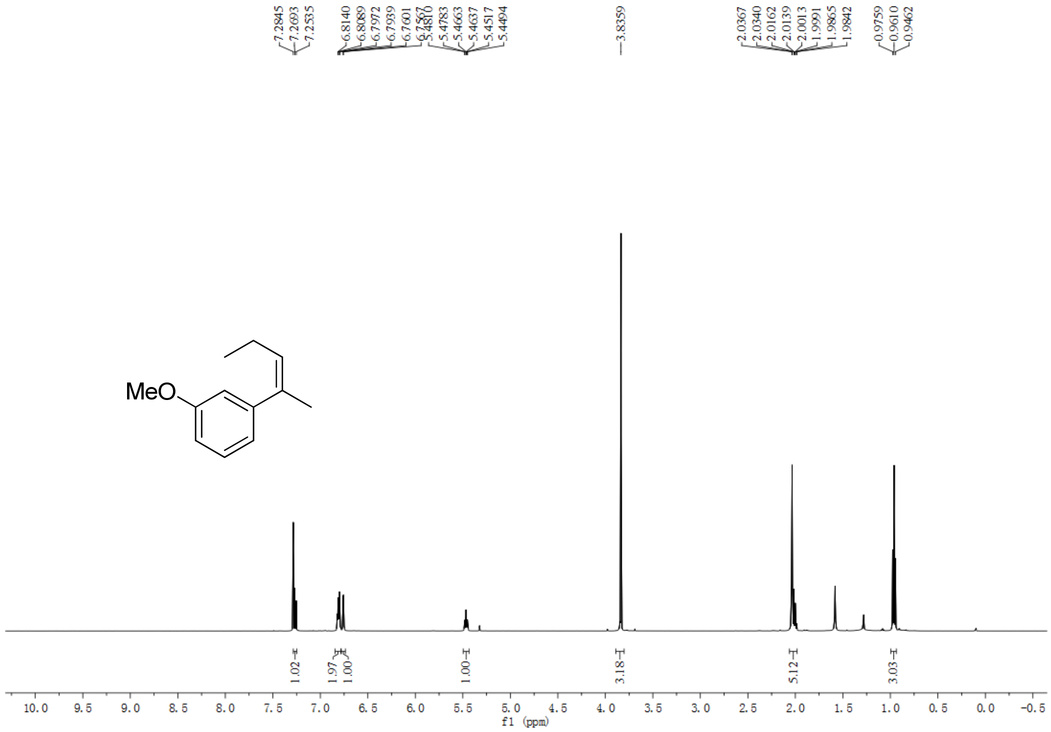

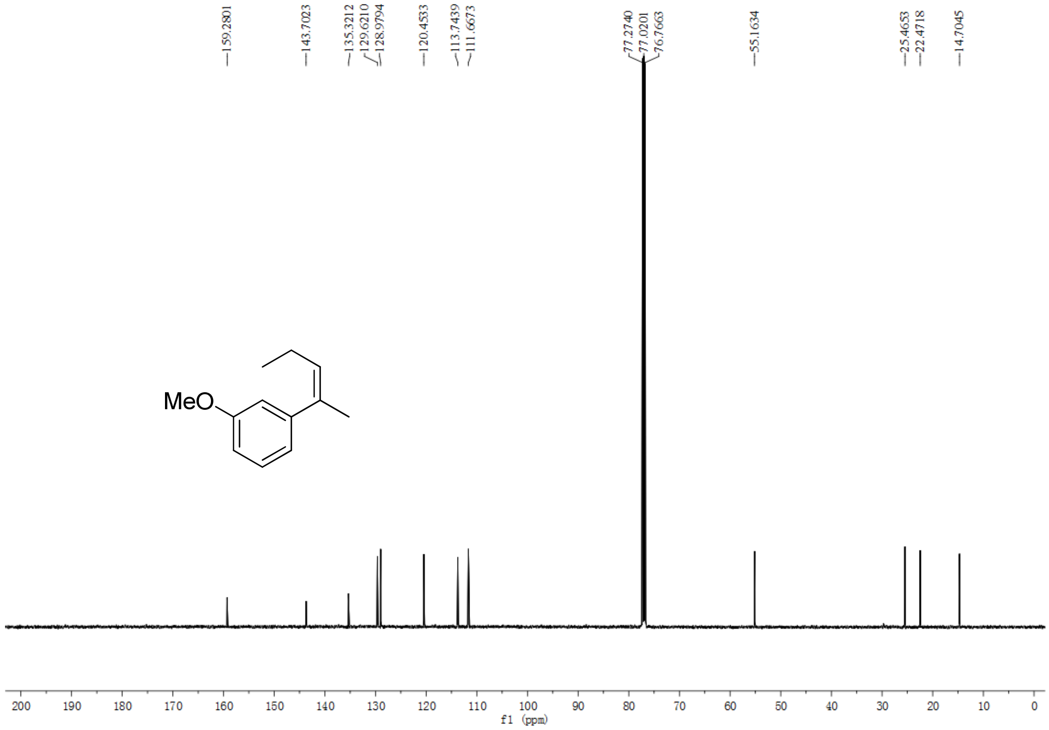

3ic(*E*)

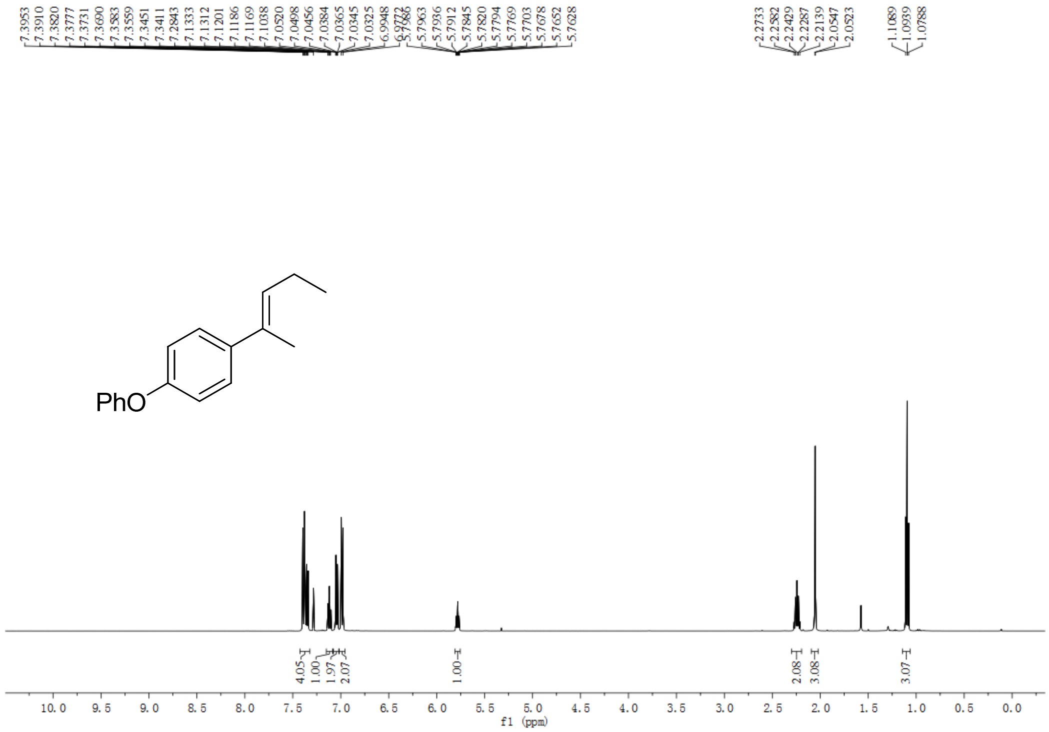

7.3953
7.3910
7.3820
7.3777
7.3731
7.3690
7.3583
7.3559
7.3451
7.3411
7.2843
7.1333
7.1312
7.1201
7.1186
7.1169
7.1038
7.0520
7.0498
7.0456
7.0384
7.0365
7.0345
7.0325
6.9948
5.7963
5.7936
5.7912
5.7845
5.7820
5.7794
5.7769
5.7703
5.7678
5.7652
5.7628
2.2733
2.2582
2.2429
2.2287
2.2139
2.0547
2.0523
1.1089
1.0939
1.0788
PhO
4.05
1.00
1.97
2.07
1.00
2.08
3.08
3.07
f1 (ppm)

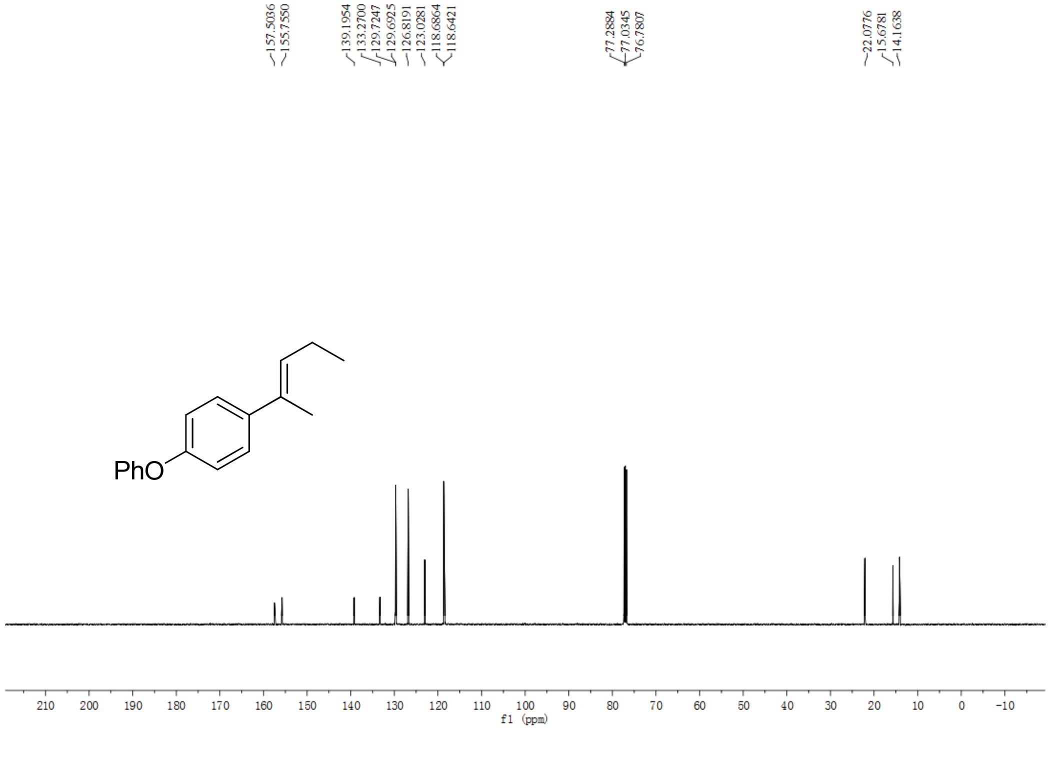

157.5036
155.7550
139.1954
133.2700
129.7247
129.6925
126.8191
123.0281
118.6864
118.6421
77.2884
77.0345
76.7807
22.0776
15.6781
14.1638
PhO
f1 (ppm)

3ic(*Z*)

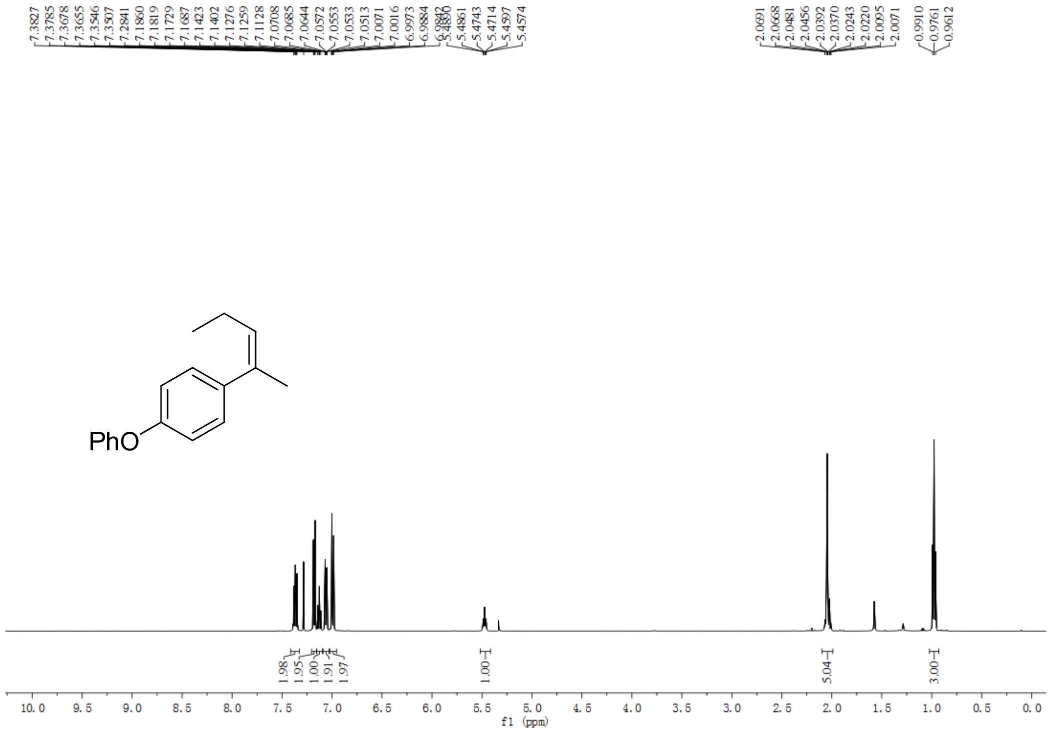
7.3827
7.3785
7.3678
7.3655
7.3546
7.3507
7.2841
7.1860
7.1819
7.1729
7.1687
7.1423
7.1402
7.1276
7.1259
7.1128
7.0708
7.0685
7.0644
7.0572
7.0553
7.0533
7.0513
7.0071
7.0016
6.9973
6.9884
5.4861
5.4743
5.4714
5.4597
5.4574
2.0691
2.0668
2.0481
2.0456
2.0392
2.0370
2.0243
2.0220
2.0095
2.0071
0.9910
0.9761
0.9612
PhO
1.98
1.95
1.00
1.91
1.97
1.00
5.04
3.00
f1 (ppm)

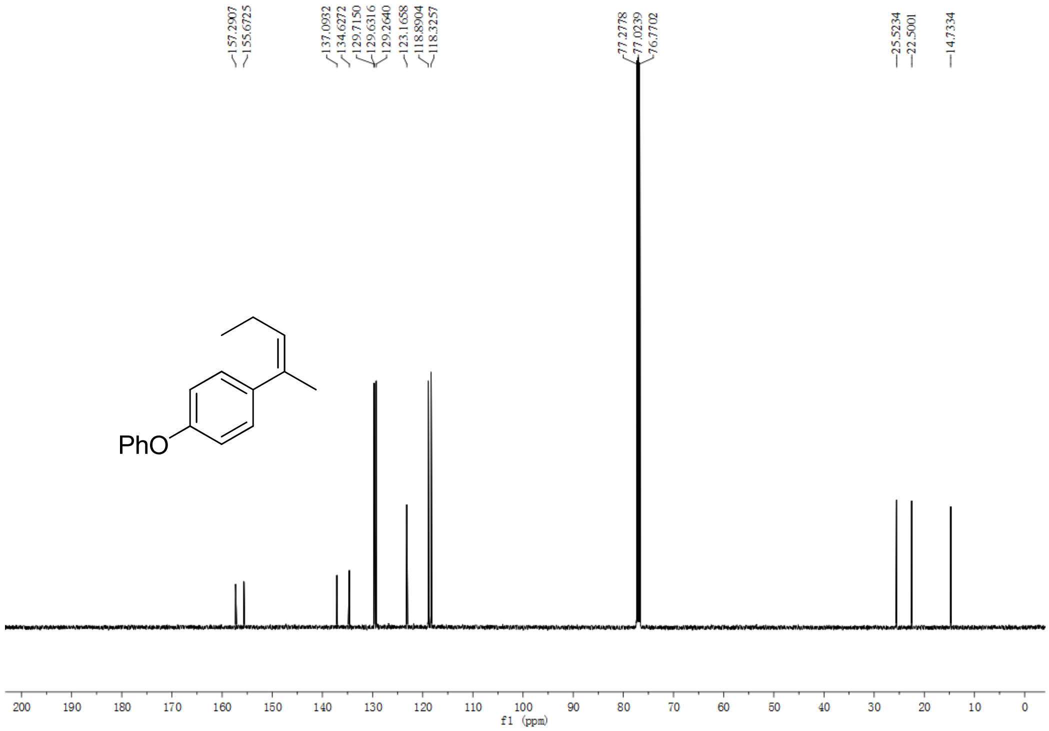
157.2907
155.6725
137.0932
134.6272
129.7150
129.6316
129.2640
123.1658
118.8904
118.3257
77.2778
77.0239
76.7702
25.5234
22.5001
14.7334
PhO
f1 (ppm)

3id(*E*)

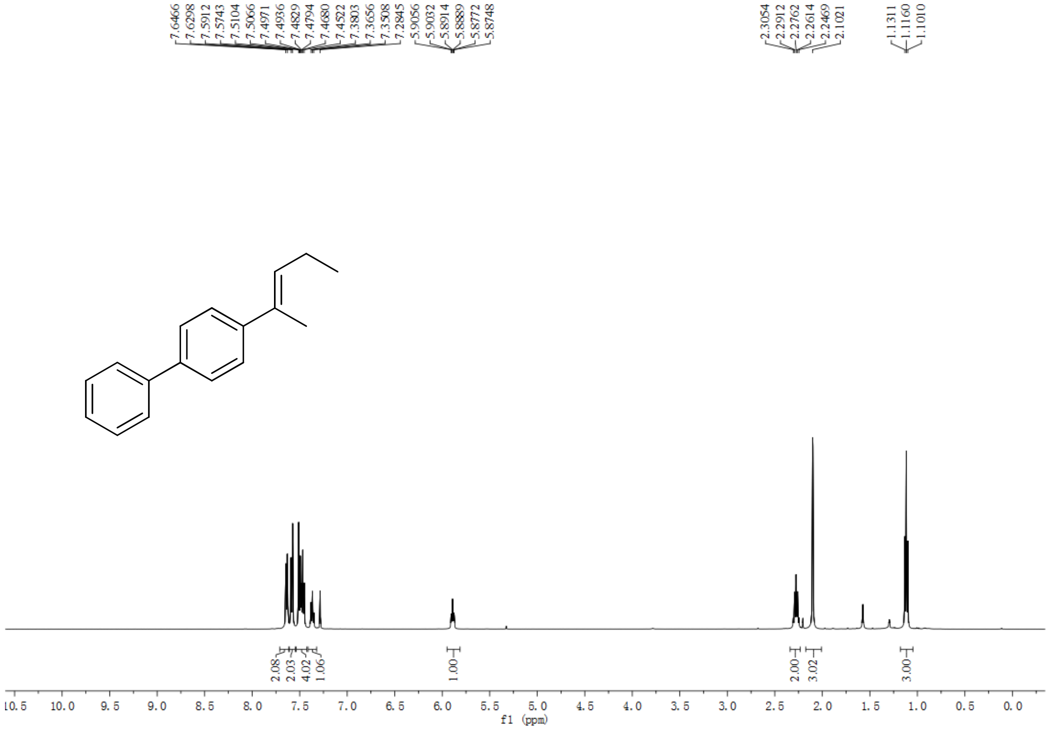

7.6466
7.6298
7.5912
7.5743
7.5104
7.5066
7.4971
7.4936
7.4829
7.4794
7.4680
7.4522
7.3803
7.3656
7.3508
7.2845
5.9056
5.9032
5.8914
5.8889
5.8772
5.8748
2.3054
2.2912
2.2762
2.2614
2.2469
2.1021
1.1311
1.1160
1.1010
2.08
2.03
4.02
1.06
1.00
2.00
3.02
3.00
f1 (ppm)

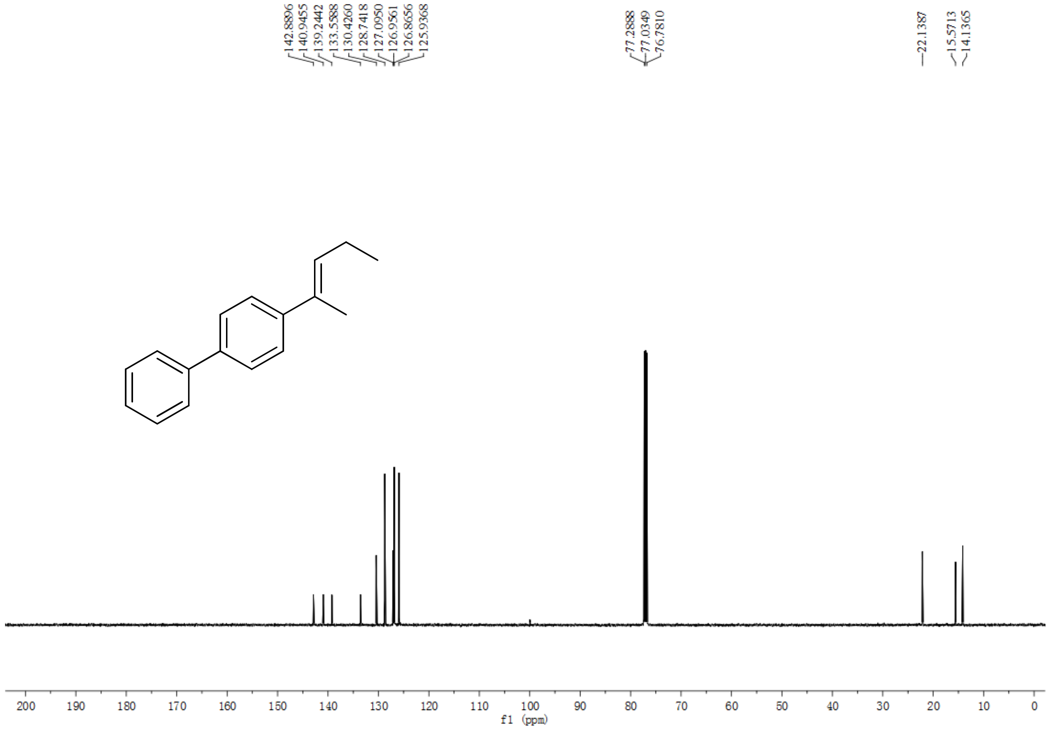

142.8896
140.9455
139.2442
133.5588
130.4260
128.7418
127.0950
126.9561
126.8656
125.9368
77.2888
77.0349
76.7810
22.1387
15.5713
14.1365
f1 (ppm)

3id(*Z*)

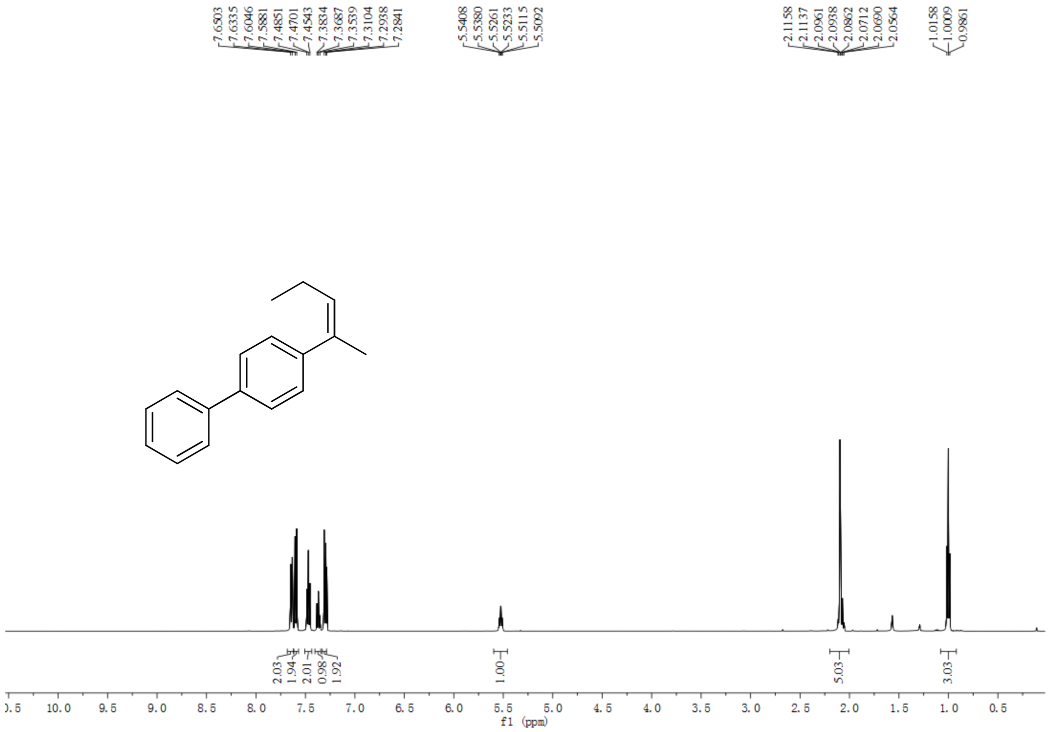

7.6503
7.6335
7.6046
7.5881
7.4851
7.4701
7.4543
7.3834
7.3687
7.3539
7.3104
7.2938
7.2841
5.5408
5.5380
5.5261
5.5233
5.5115
5.5092
2.1158
2.1137
2.0961
2.0938
2.0862
2.0712
2.0690
2.0564
1.0158
1.0009
0.9861
2.03
1.94
2.01
0.98
1.92
1.00
5.03
3.03
f1 (ppm)

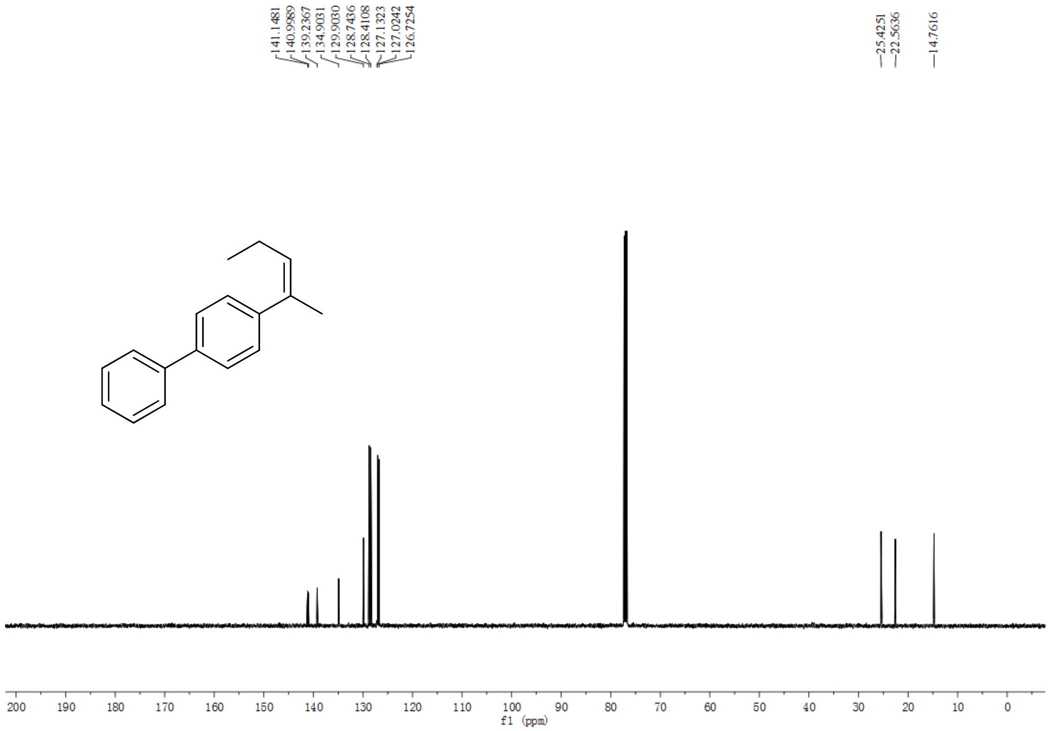

141.1481
140.9989
139.2367
134.9031
129.9030
128.7436
128.4108
127.1323
127.0242
126.7254
25.4251
22.5636
14.7616
f1 (ppm)

3ie(*E*)

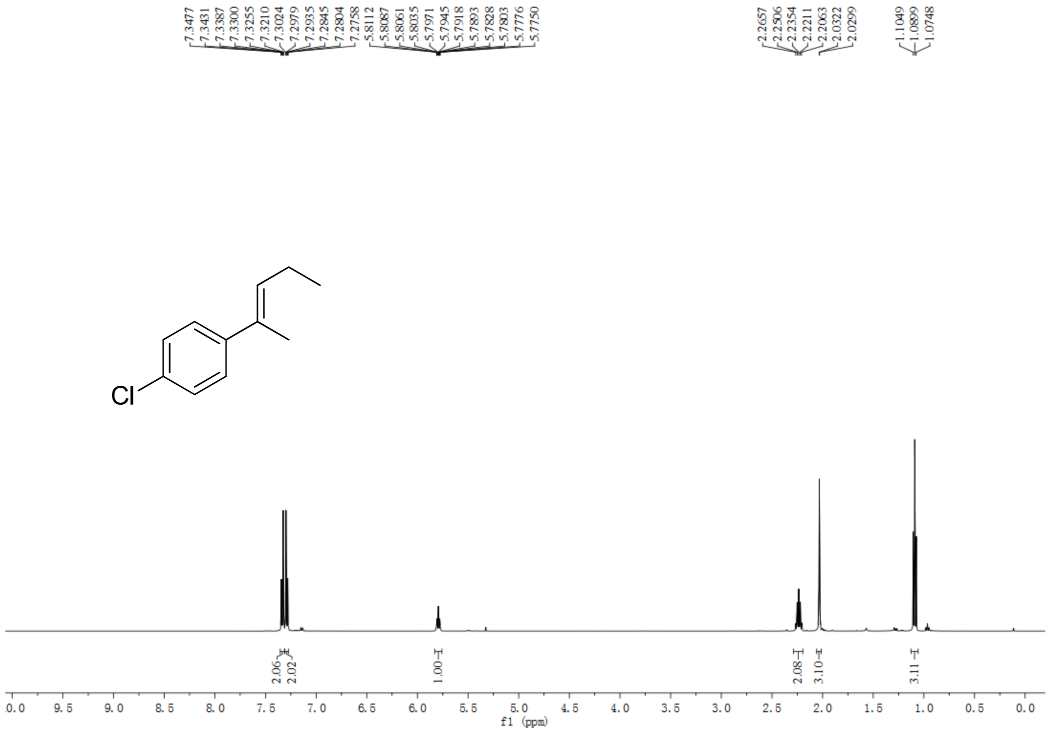
7.3477
7.3431
7.3387
7.3300
7.3255
7.3210
7.3024
7.2979
7.2935
7.2845
7.2804
7.2758
5.8112
5.8087
5.8061
5.8035
5.7971
5.7945
5.7918
5.7893
5.7828
5.7803
5.7776
5.7750
2.2657
2.2506
2.2354
2.2211
2.2063
2.0322
2.0299
1.1049
1.0899
1.0748
Cl
2.06
2.02
1.00
2.08
3.10
3.11
f1 (ppm)

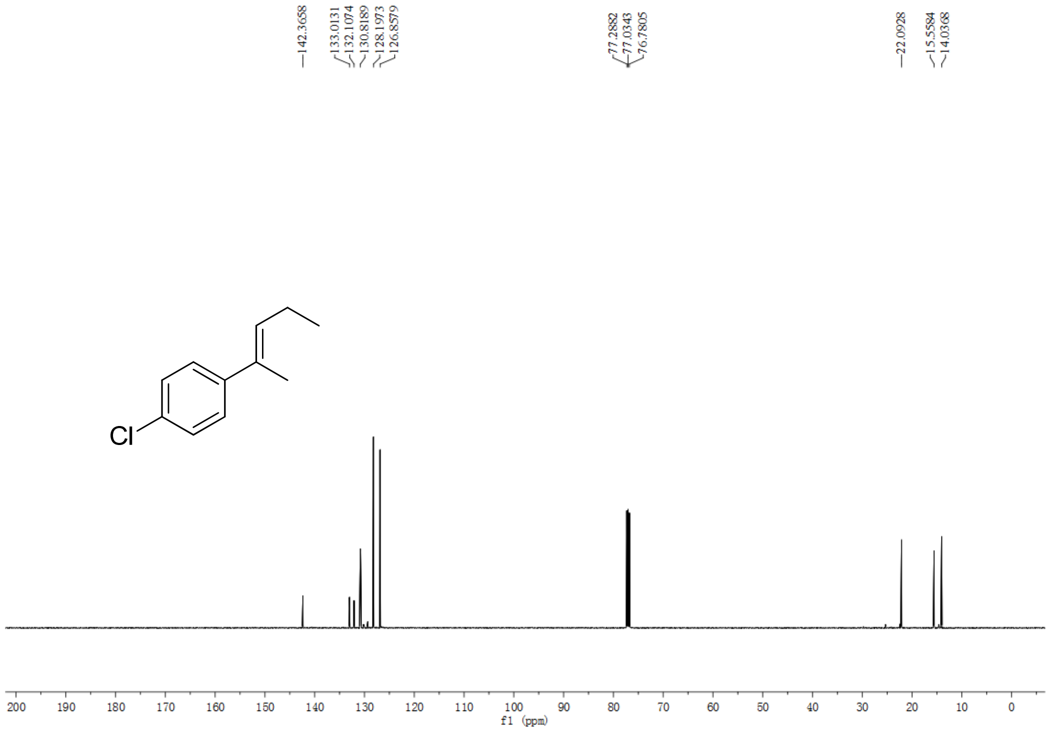
142.3658
133.0131
132.1074
130.8189
128.1973
126.8579
77.2882
77.0343
76.7805
22.0928
15.5584
14.0368
Cl
f1 (ppm)

3ie(*E*)

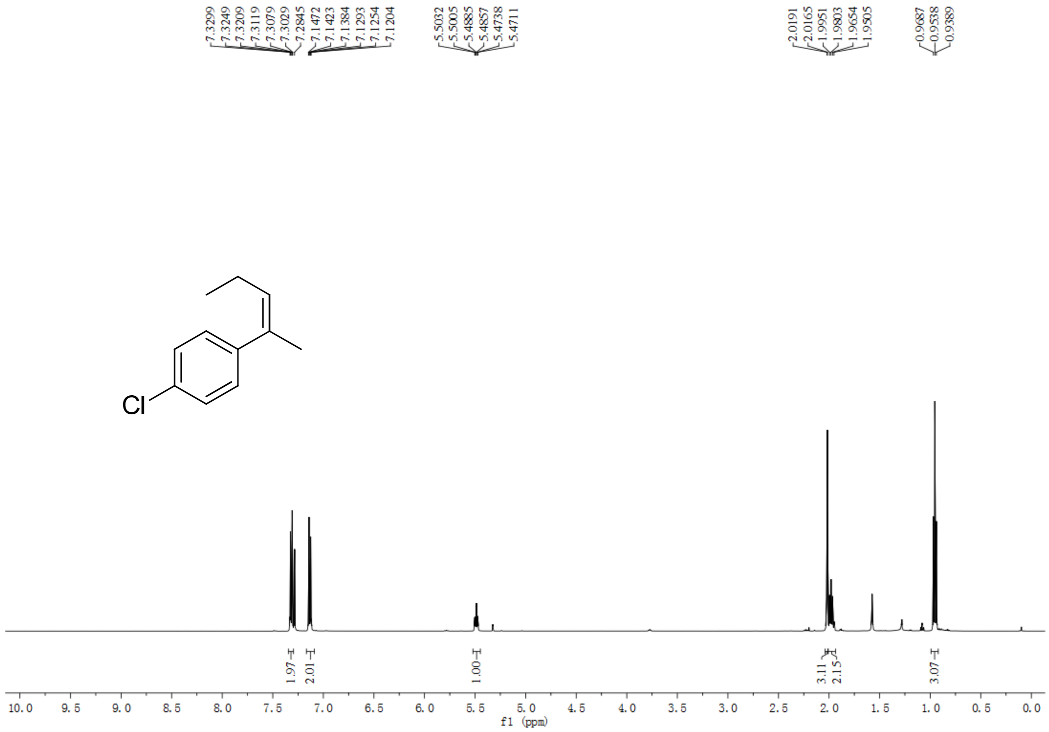
7.3299
7.3249
7.3209
7.3119
7.3079
7.3029
7.2845
7.1472
7.1423
7.1384
7.1293
7.1254
7.1204
5.5032
5.5005
5.4885
5.4857
5.4738
5.4711
2.0191
2.0165
1.9951
1.9803
1.9654
1.9505
0.9687
0.9538
0.9389
Cl
1.97
2.01
1.00
3.11
2.15
3.07
10.0 9.5 9.0 8.5 8.0 7.5 7.0 6.5 6.0 5.5 5.0 4.5 4.0 3.5 3.0 2.5 2.0 1.5 1.0 0.5 0.0
f1 (ppm)

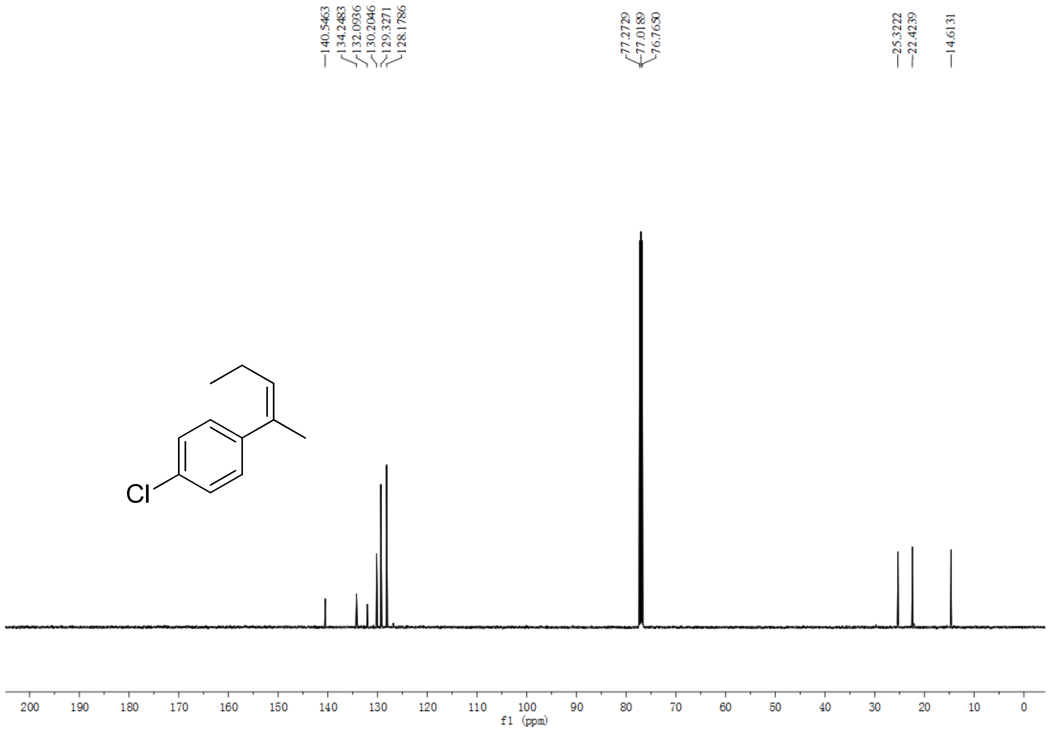
140.5463
134.2483
132.0936
130.2046
129.3271
128.1786
77.2729
77.0189
76.7650
25.3222
22.4239
14.6131
Cl
200 190 180 170 160 150 140 130 120 110 100 90 80 70 60 50 40 30 20 10 0
f1 (ppm)

3if(*E*)

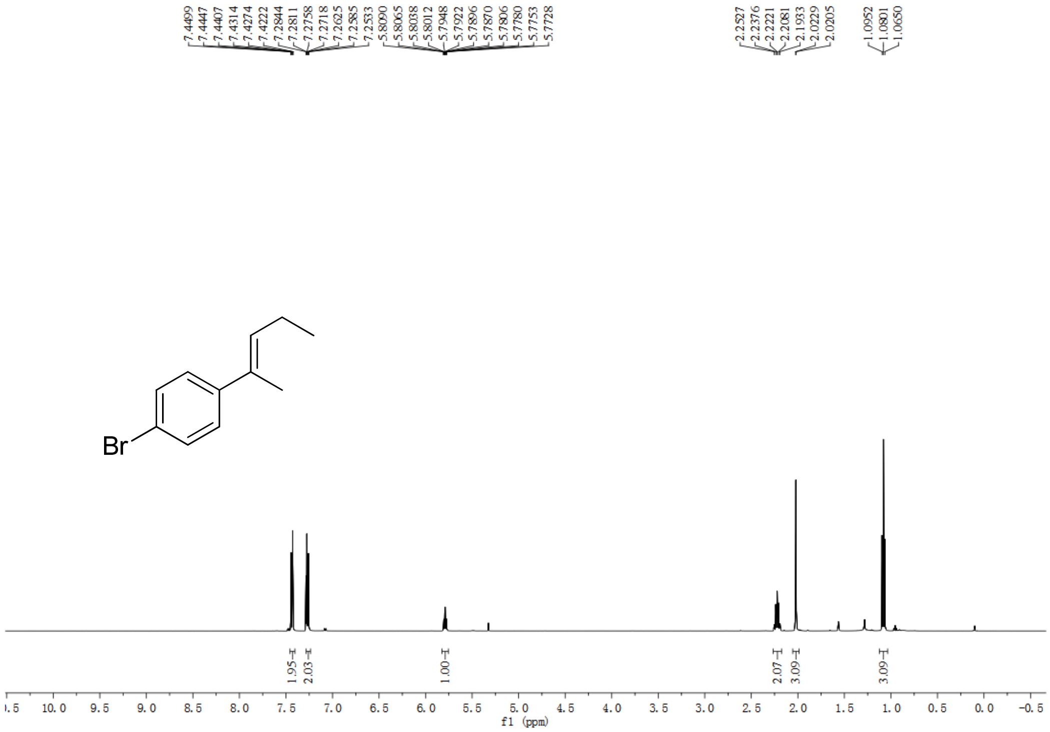
7.4499
7.4447
7.4407
7.4314
7.4274
7.4222
7.2844
7.2811
7.2758
7.2718
7.2625
7.2585
7.2533
5.8090
5.8065
5.8038
5.8012
5.7948
5.7922
5.7896
5.7870
5.7806
5.7780
5.7753
5.7728
2.2527
2.2376
2.2221
2.2081
2.1933
2.0229
2.0205
1.0952
1.0801
1.0650
Br
1.95
2.03
1.00
2.07
3.09
3.09
f1 (ppm)

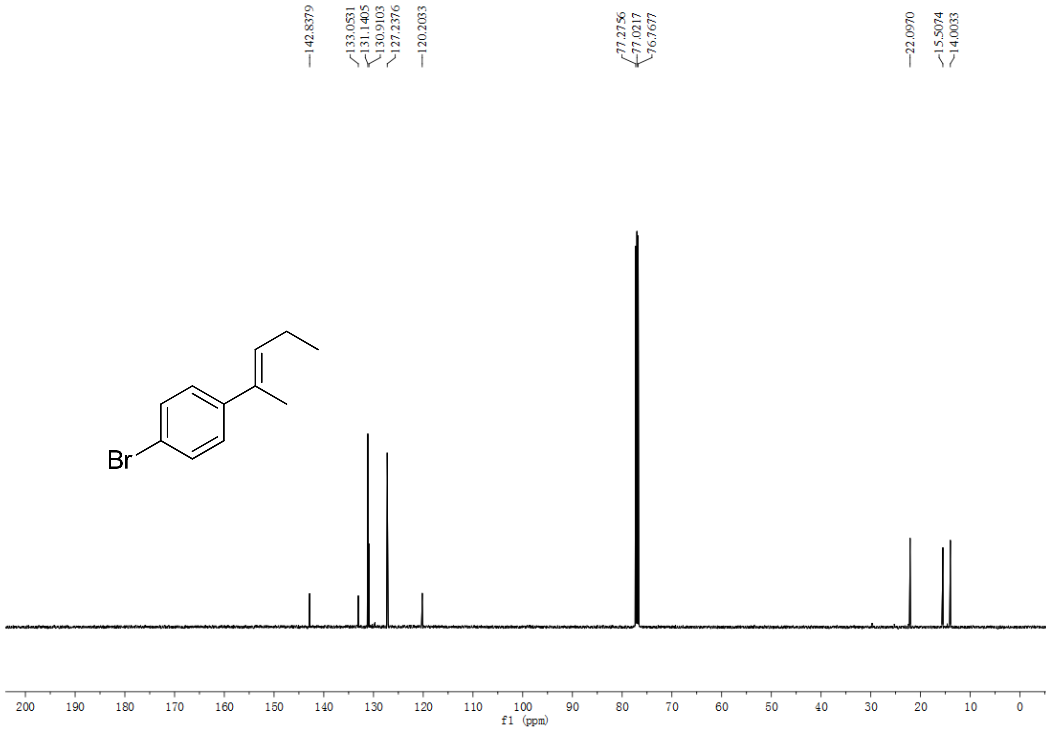
142.8379
133.0531
131.1405
130.9103
127.2376
120.2033
77.2756
77.0217
76.7677
22.0970
15.5074
14.0033
Br
f1 (ppm)

3if(*Z*)

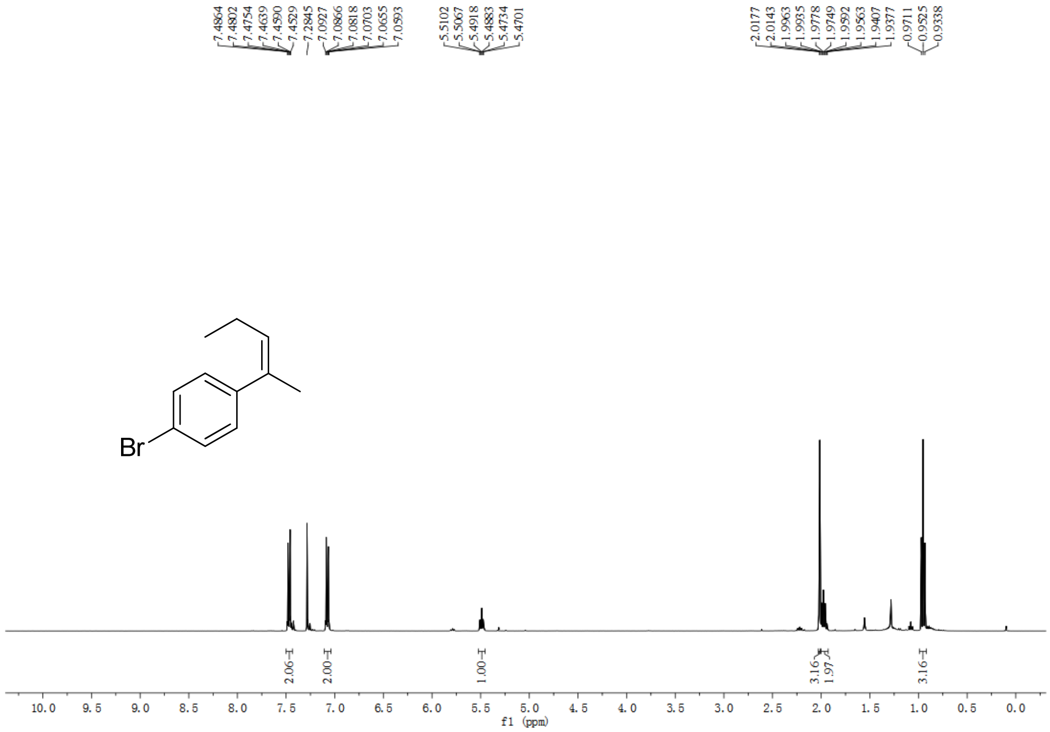

7.4864
7.4802
7.4754
7.4639
7.4590
7.4529
7.2845
7.0927
7.0866
7.0818
7.0703
7.0655
7.0593
5.5102
5.5067
5.4918
5.4883
5.4734
5.4701
2.0177
2.0143
1.9963
1.9935
1.9778
1.9749
1.9592
1.9563
1.9407
1.9377
0.9711
0.9525
0.9338
Br
2.06
2.00
1.00
3.16
1.97
3.16
10.0 9.5 9.0 8.5 8.0 7.5 7.0 6.5 6.0 5.5 5.0 4.5 4.0 3.5 3.0 2.5 2.0 1.5 1.0 0.5 0.0
f1 (ppm)

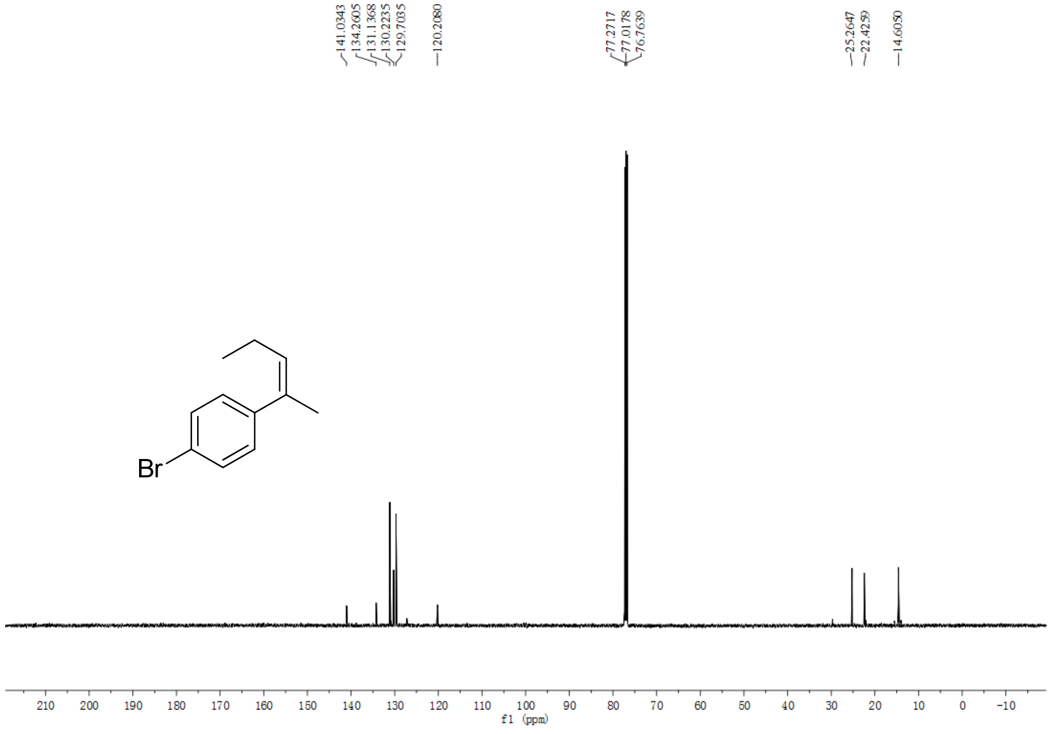

141.0343
134.2605
131.1368
130.2235
129.7035
120.2080
77.2717
77.0178
76.7639
25.2647
22.4259
14.6050
Br
210 200 190 180 170 160 150 140 130 120 110 100 90 80 70 60 50 40 30 20 10 0 -10
f1 (ppm)

3ig

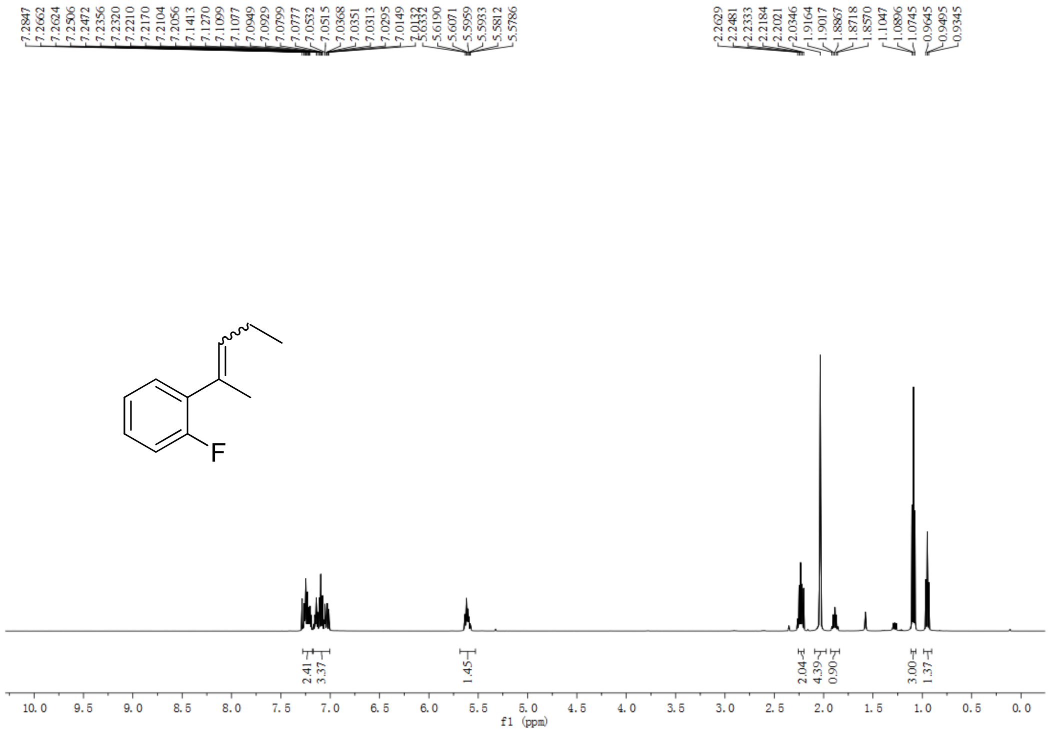

7.2847 7.2662 7.2624 7.2506 7.2472 7.2356 7.2320 7.2210 7.2170 7.2104 7.2056 7.1413 7.1270 7.1099 7.1077 7.0949 7.0929 7.0799 7.0777 7.0532 7.0515 7.0368 7.0351 7.0313 7.0295 7.0149 7.0132 5.6332 5.6190 5.6071 5.5959 5.5933 5.5812 5.5786
2.2629 2.2481 2.2333 2.2184 2.2021 2.0346 1.9164 1.9017 1.8867 1.8718 1.8570 1.1047 1.0896 1.0745 0.9645 0.9495 0.9345
F
2.41 3.37 1.45 2.04 4.39 0.90 3.00 1.37
f1 (ppm)

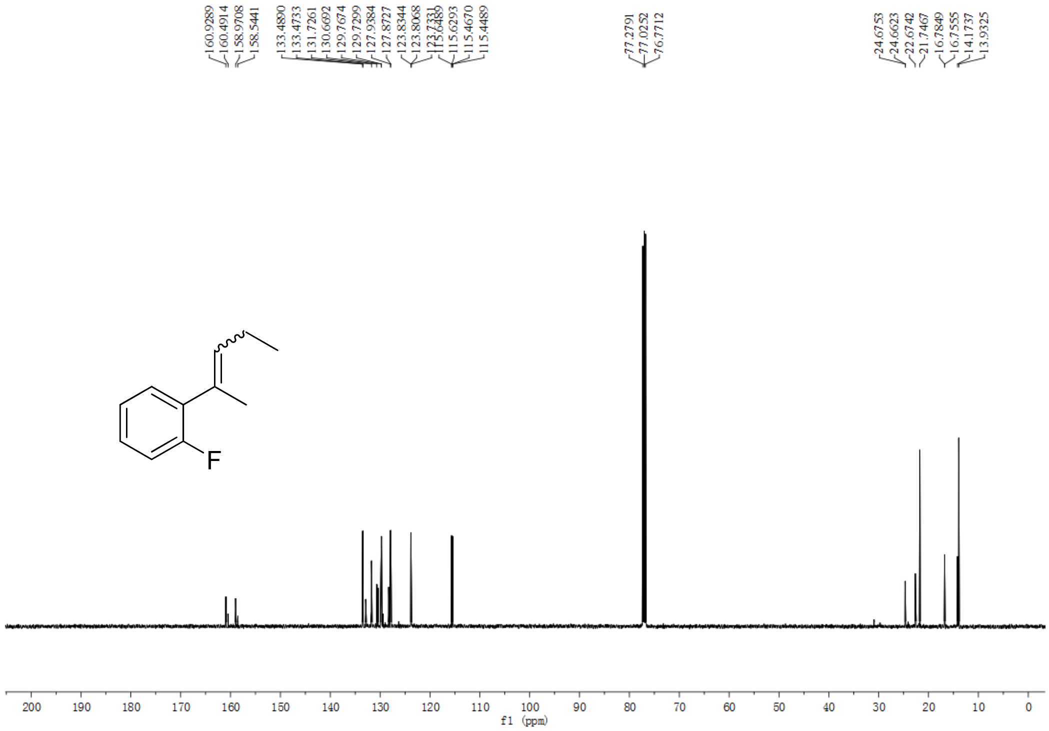

160.9289 160.4914 158.9708 158.5441 133.4890 133.4733 131.7261 130.6692 129.7674 129.7299 127.9384 127.8727 123.8344 123.8068 115.6293 115.4670 115.4489
77.2791 77.0252 76.7712
24.6753 24.6623 22.6742 21.7467 16.7849 16.7555 14.1737 13.9325
F
f1 (ppm)

3ih

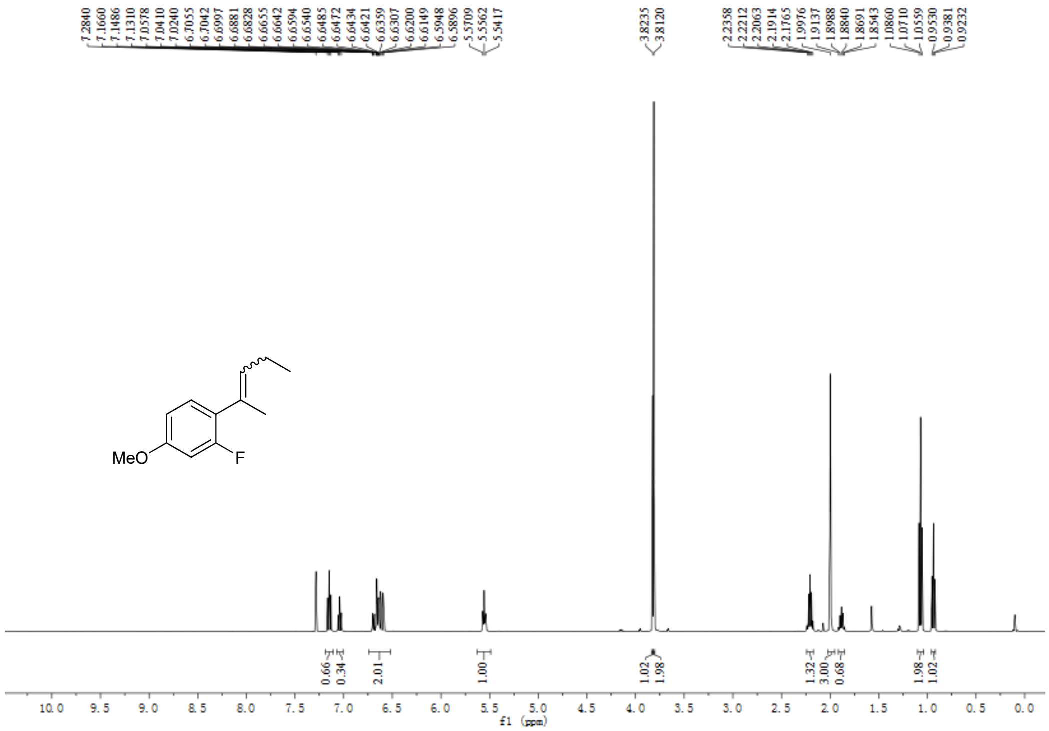

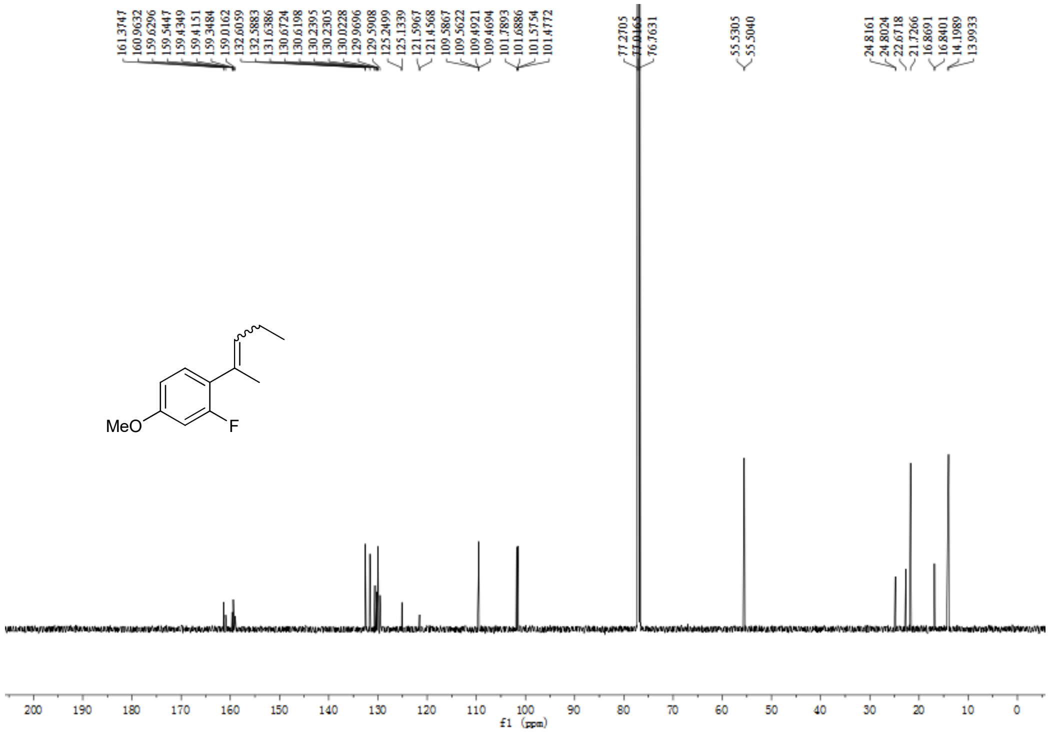

3ii(*E*)

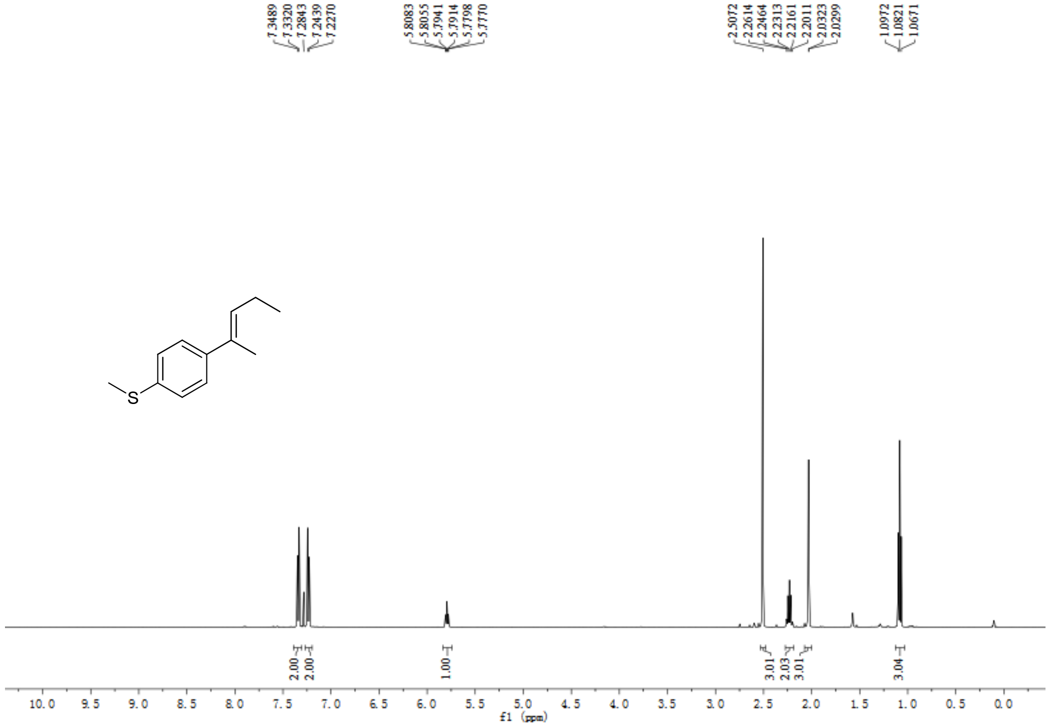

7.3489
7.3320
7.2843
7.2439
7.2270
5.8083
5.8055
5.7941
5.7914
5.7798
5.7770
2.5072
2.2614
2.2464
2.2313
2.2161
2.2011
2.0323
2.0299
1.0972
1.0821
1.0671
S
2.00
2.00
1.00
3.01
2.03
3.01
3.04
f1 (ppm)

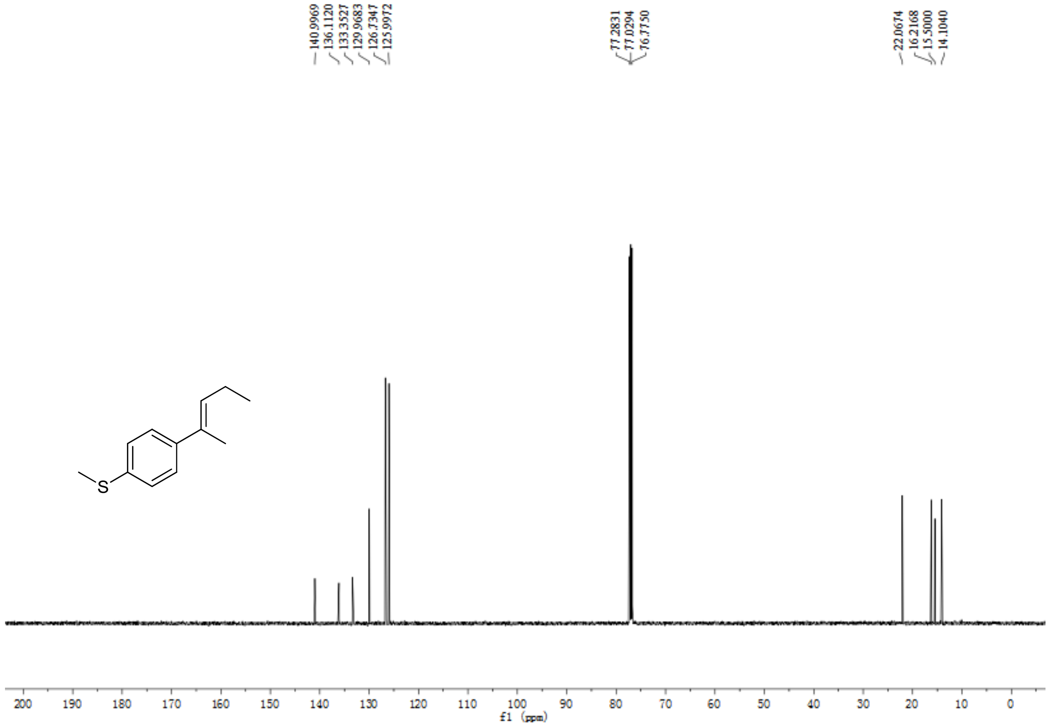

140.9969
136.1120
133.3527
129.9683
126.7347
125.9972
77.2831
77.0294
76.7750
22.0674
16.2168
15.5000
14.1040
S
f1 (ppm)

3ii(Z)

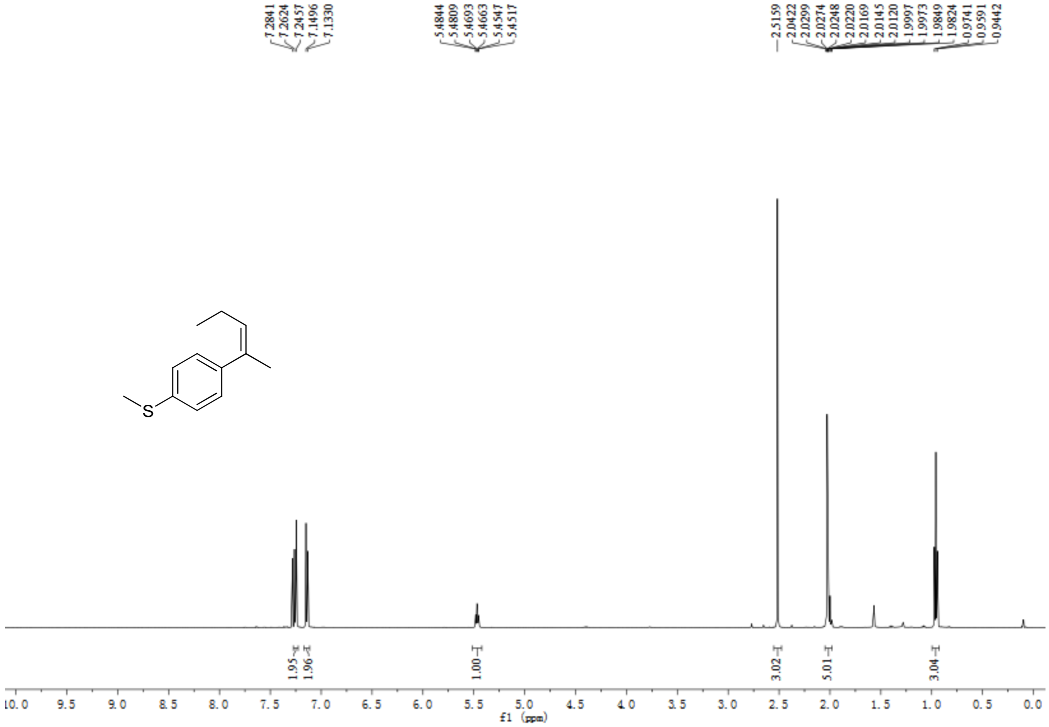

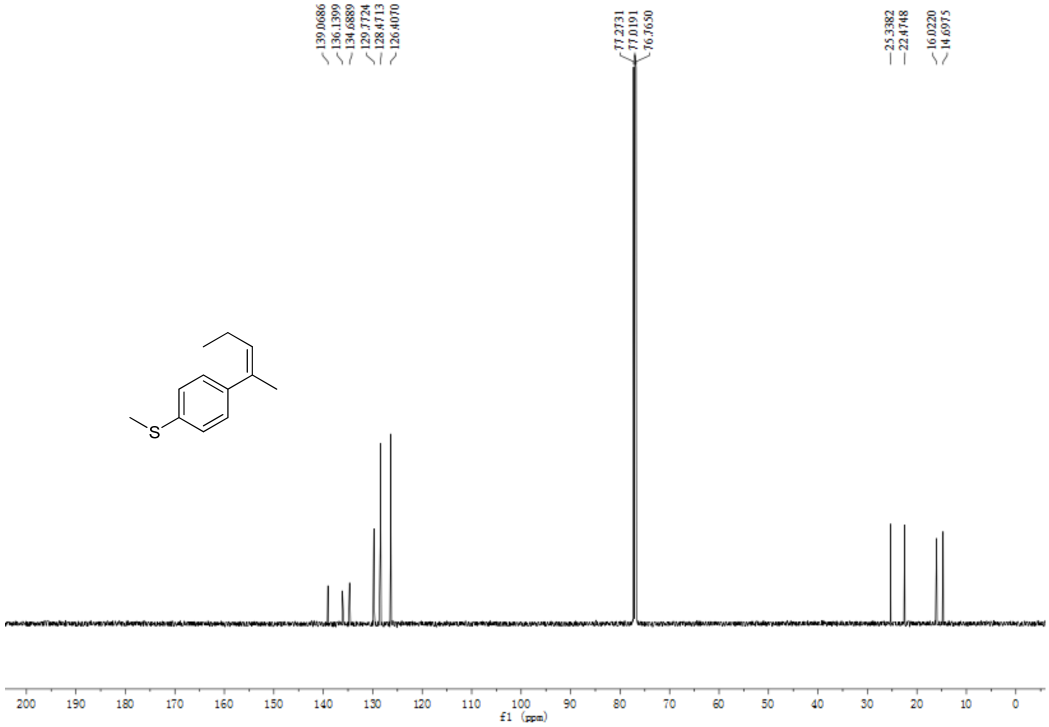

3ij

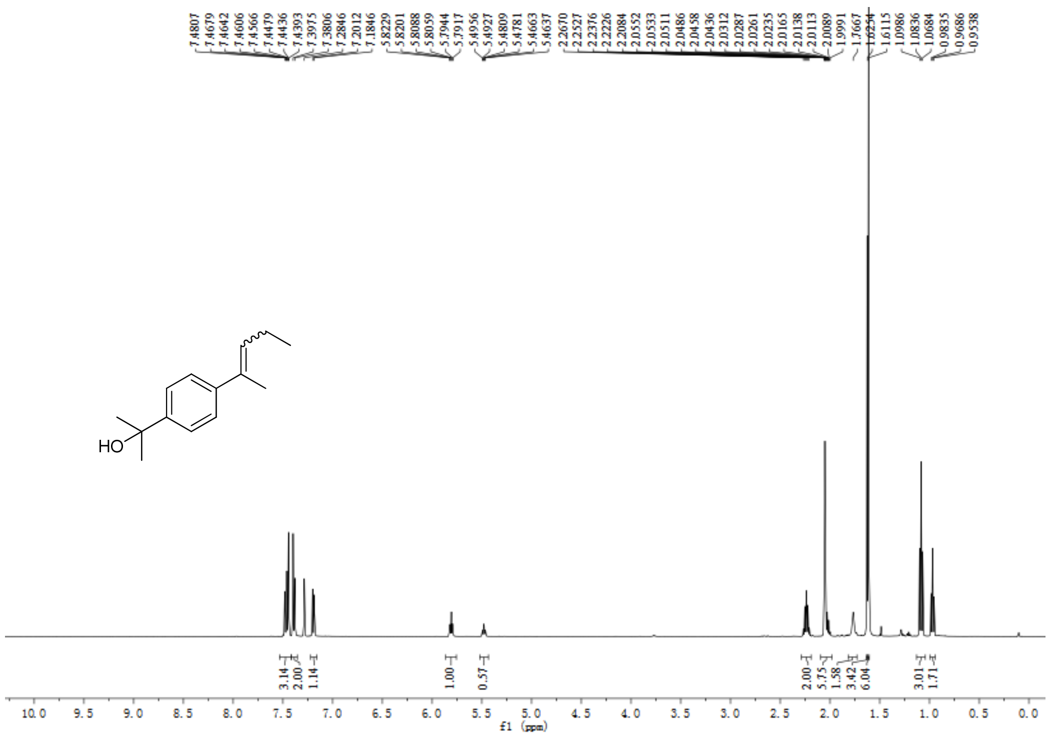

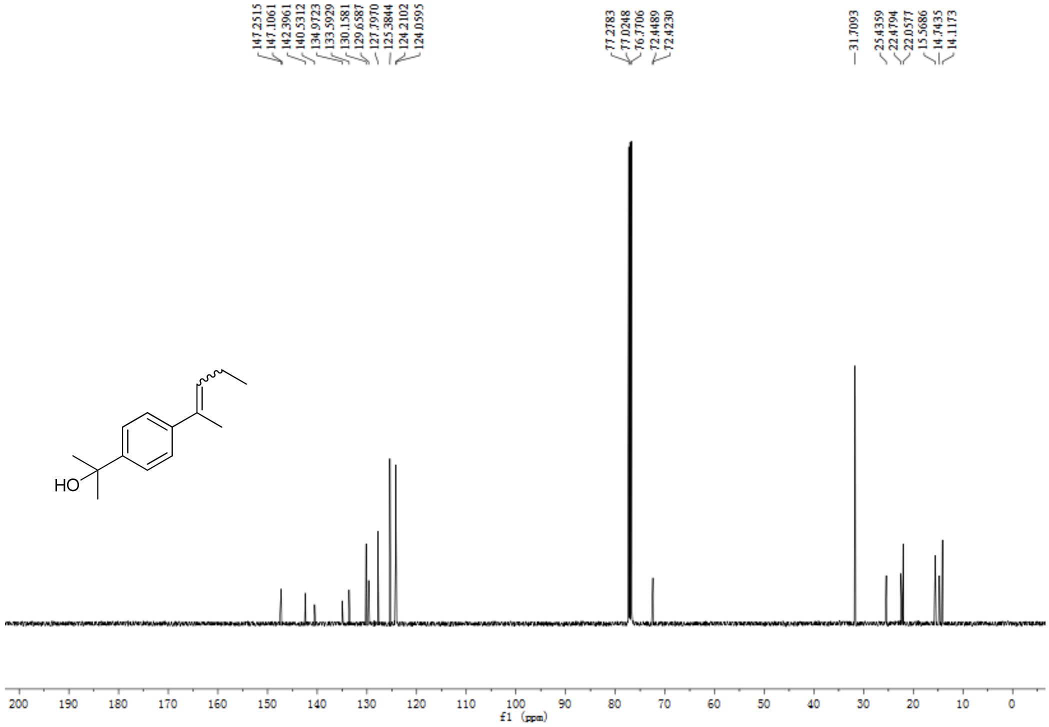

3ik

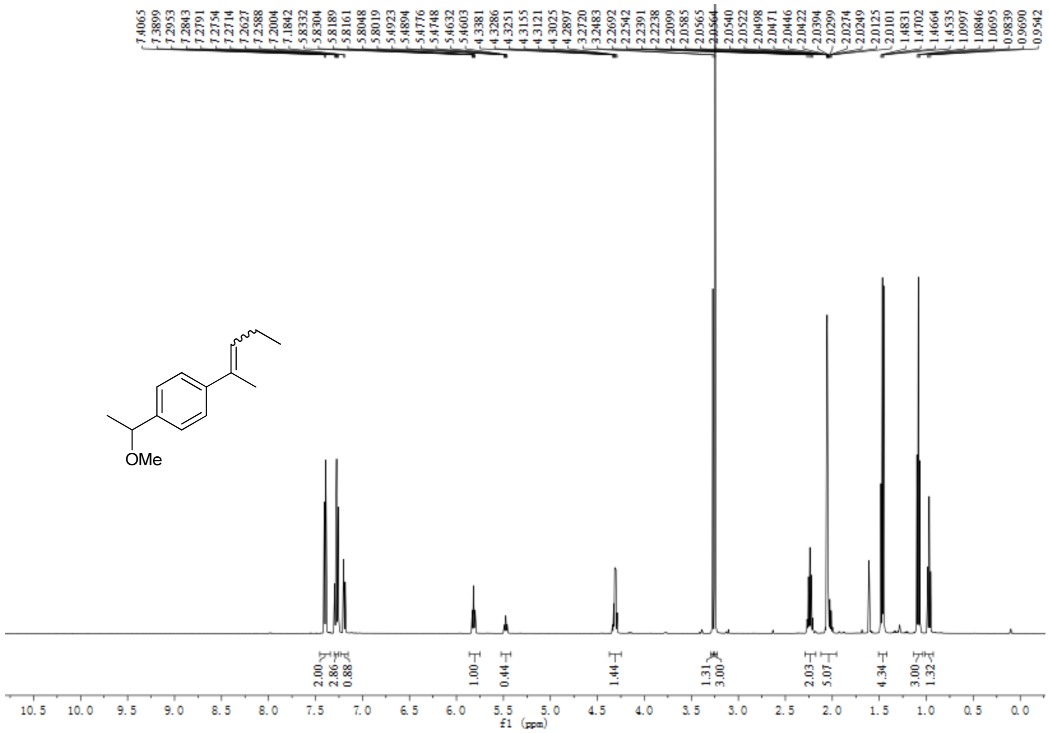
7.4065
7.3899
7.2953
7.2843
7.2791
7.2754
7.2714
7.2627
7.2588
7.2004
7.1842
5.8332
5.8304
5.8189
5.8161
5.8048
5.8019
5.4923
5.4894
5.4776
5.4748
5.4632
5.4603
4.3381
4.3286
4.3251
4.3155
4.3121
4.3025
4.2897
3.2720
3.2483
2.2692
2.2542
2.2391
2.2238
2.2099
2.0585
2.0565
2.0564
2.0540
2.0522
2.0498
2.0471
2.0446
2.0422
2.0394
2.0299
2.0274
2.0249
2.0125
2.0101
1.4831
1.4702
1.4664
1.4535
1.0997
1.0846
1.0695
0.9839
0.9690
0.9542
OMe
2.00
2.86
0.88
1.00
0.44
1.44
1.31
3.00
2.03
5.07
4.34
3.00
1.32
f1 (ppm)

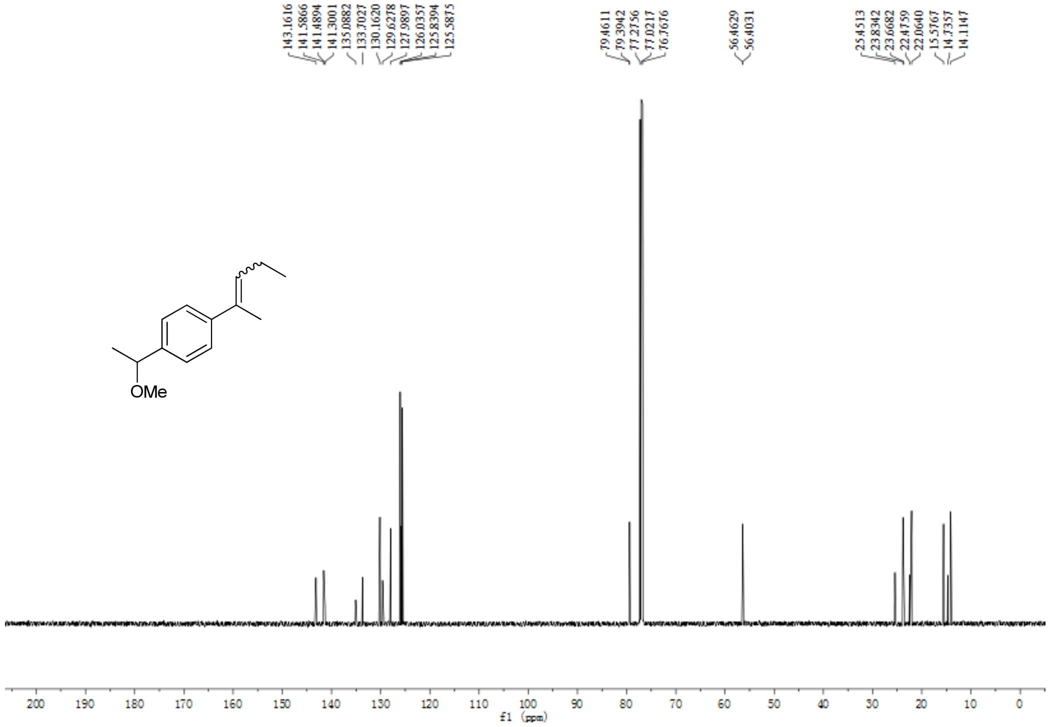
143.1616
141.5866
141.4894
141.3001
135.0882
133.7027
130.1620
129.6278
127.9897
126.0357
125.8394
125.5875
79.4611
79.3942
77.2756
77.0217
76.7676
56.4629
56.4031
25.4513
23.8342
23.6682
22.4759
22.0640
15.5767
14.7357
14.1147
OMe
f1 (ppm)

3il(*E*)

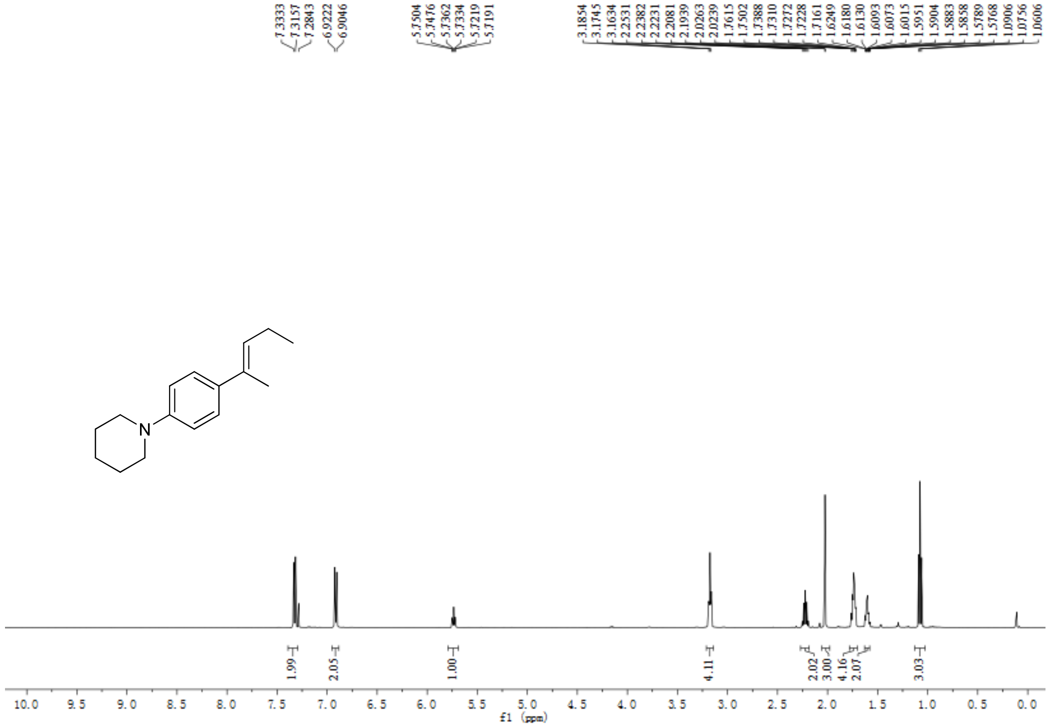

7.3333
7.3157
7.2843
6.9222
6.9046
5.7504
5.7476
5.7362
5.7334
5.7219
5.7191
3.1854
3.1745
3.1634
2.2531
2.2382
2.2231
2.2081
2.1939
2.0263
2.0239
1.7615
1.7502
1.7388
1.7310
1.7272
1.7228
1.7161
1.6249
1.6180
1.6130
1.6093
1.6073
1.6015
1.5951
1.5904
1.5883
1.5858
1.5789
1.5768
1.0906
1.0756
1.0606
N
1.99
2.05
1.00
4.11
2.02
3.00
4.16
2.07
3.03
10.0 9.5 9.0 8.5 8.0 7.5 7.0 6.5 6.0 5.5 5.0 4.5 4.0 3.5 3.0 2.5 2.0 1.5 1.0 0.5 0.0
f1 (ppm)

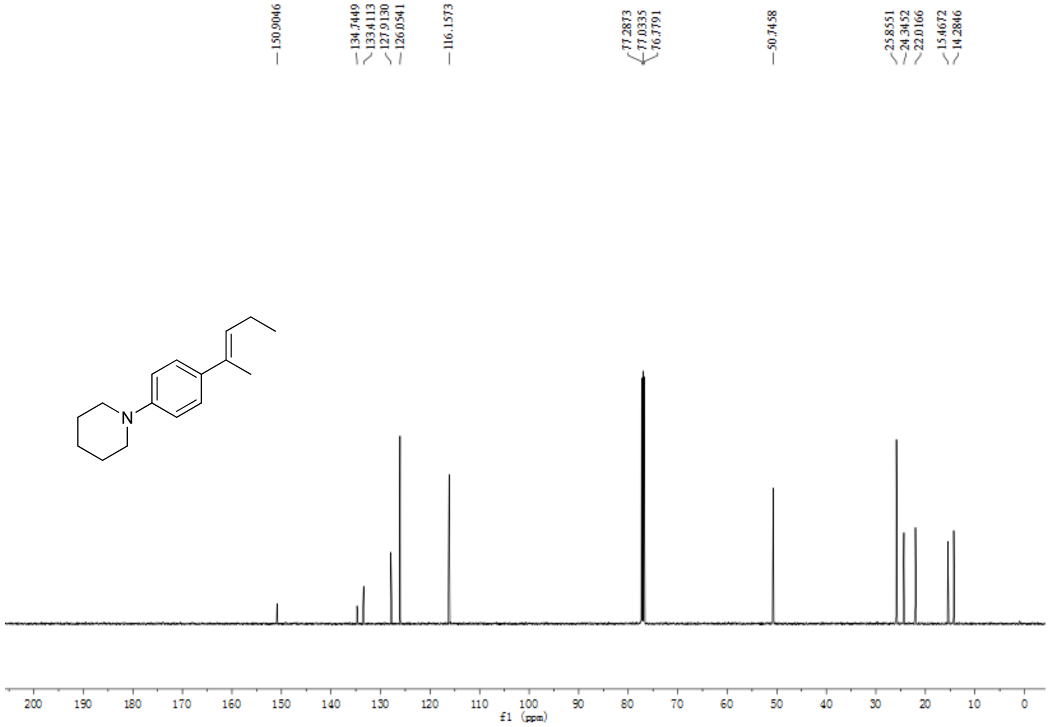

150.9046
134.7449
133.4113
127.9130
126.0541
116.1573
77.2873
77.0335
76.7791
50.7458
25.8551
24.3452
22.0166
15.4672
14.2846
N
200 190 180 170 160 150 140 130 120 110 100 90 80 70 60 50 40 30 20 10 0
f1 (ppm)

3il(*Z*)

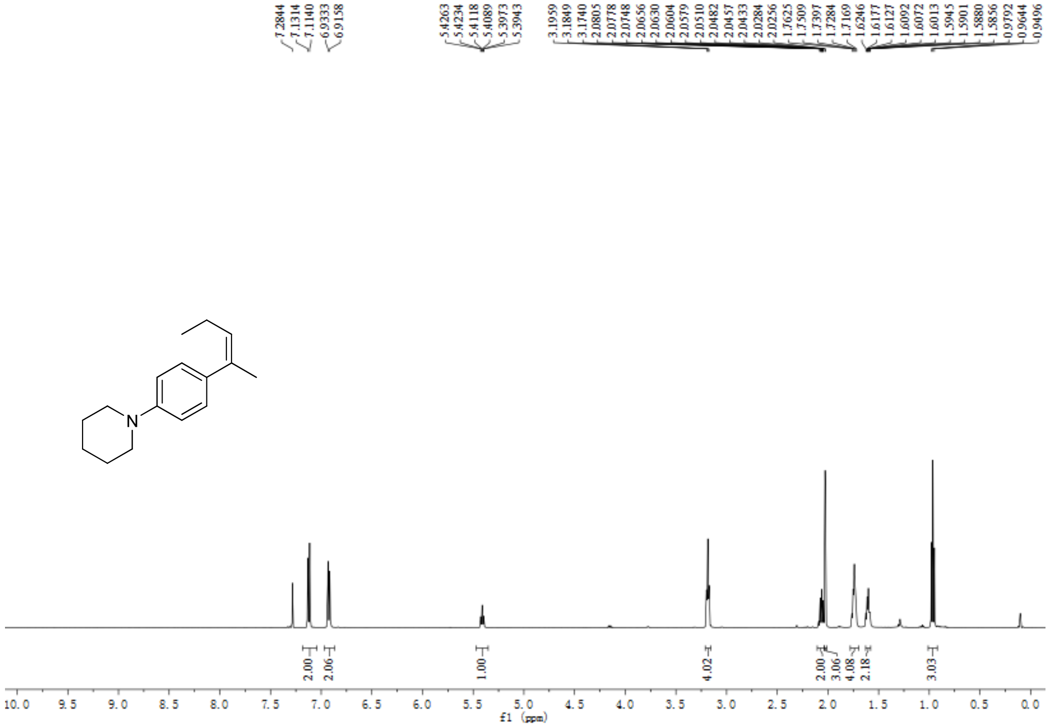
7.2844
7.1314
7.1140
6.9333
6.9158
5.4263
5.4234
5.4118
5.4089
5.3973
5.3943
3.1959
3.1849
3.1740
2.0805
2.0778
2.0748
2.0656
2.0630
2.0604
2.0579
2.0510
2.0482
2.0457
2.0433
2.0284
2.0256
1.7625
1.7509
1.7397
1.7284
1.7169
1.6246
1.6177
1.6127
1.6092
1.6072
1.6013
1.5945
1.5901
1.5880
1.5856
0.9792
0.9644
0.9496
2.00
2.06
1.00
4.02
2.00
3.06
4.08
2.18
3.03
f1 (ppm)

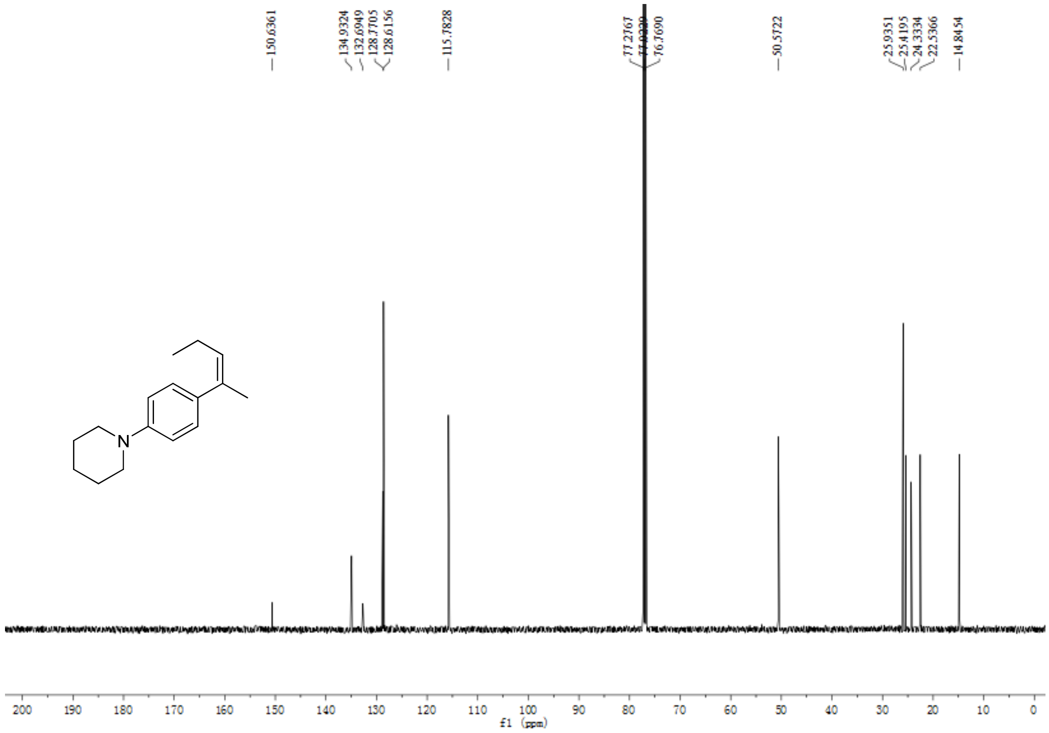
150.6361
134.9324
132.6949
128.7705
128.6156
115.7828
77.2767
76.7690
50.5722
25.9351
25.4195
24.3334
22.5366
14.8454
f1 (ppm)

3im(*E*)

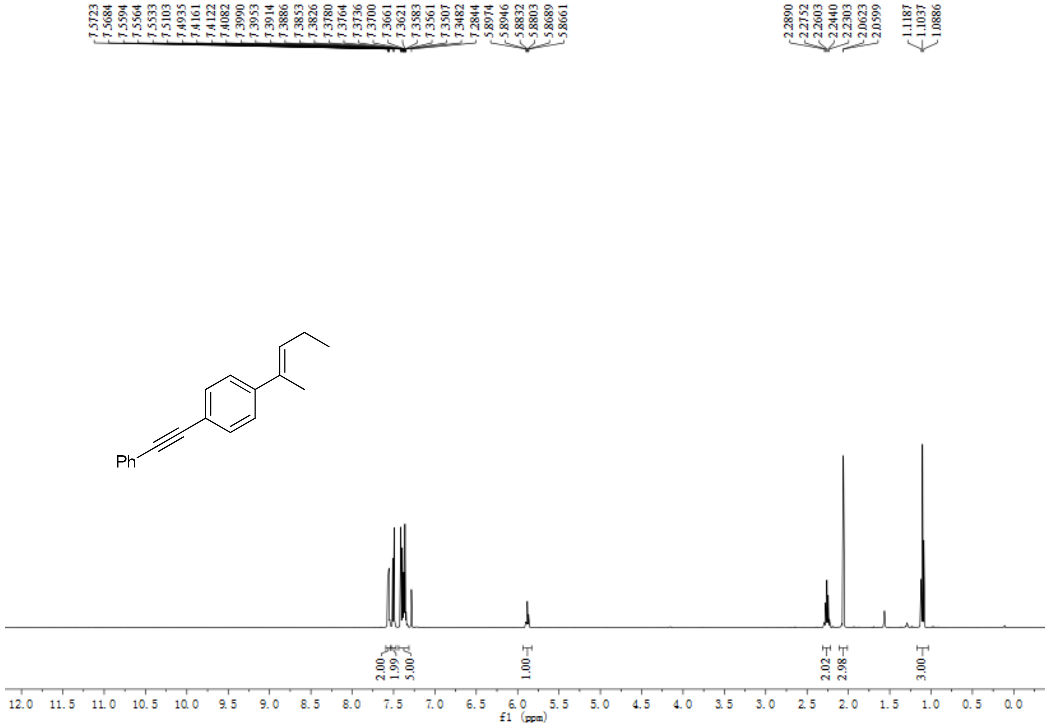

7.5723
7.5684
7.5594
7.5564
7.5533
7.5103
7.4935
7.4161
7.4122
7.4082
7.3990
7.3953
7.3914
7.3886
7.3853
7.3826
7.3780
7.3764
7.3736
7.3700
7.3661
7.3621
7.3583
7.3561
7.3507
7.3482
7.2844
5.8974
5.8946
5.8832
5.8803
5.8689
5.8661
2.2890
2.2752
2.2603
2.2440
2.2303
2.0623
2.0599
1.1187
1.1037
1.0886
Ph
2.00
1.99
5.00
1.00
2.02
2.98
3.00
12.0
11.5
11.0
10.5
10.0
9.5
9.0
8.5
8.0
7.5
7.0
6.5
6.0
5.5
5.0
4.5
4.0
3.5
3.0
2.5
2.0
1.5
1.0
0.5
0.0
f1 (ppm)

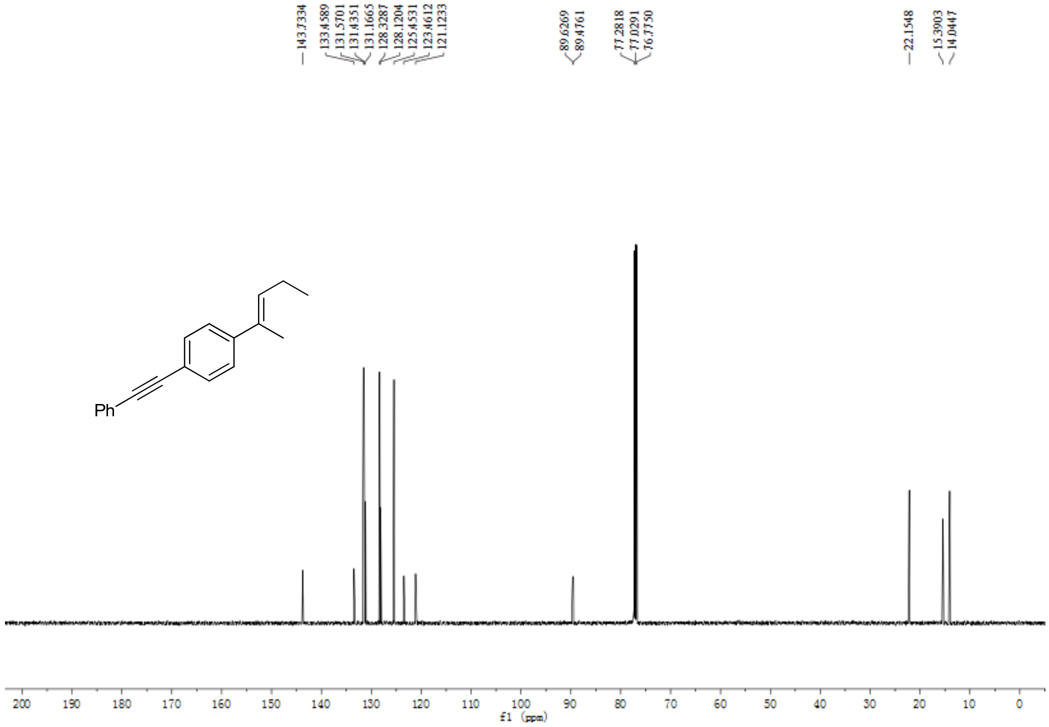

143.7334
133.4589
131.5701
131.4351
131.1665
128.3287
128.1204
125.4531
123.4612
121.1233
89.6269
89.4761
77.2818
77.0291
76.7750
22.1548
15.3903
14.0447
Ph
200
190
180
170
160
150
140
130
120
110
100
90
80
70
60
50
40
30
20
10
0
f1 (ppm)

3im(Z)

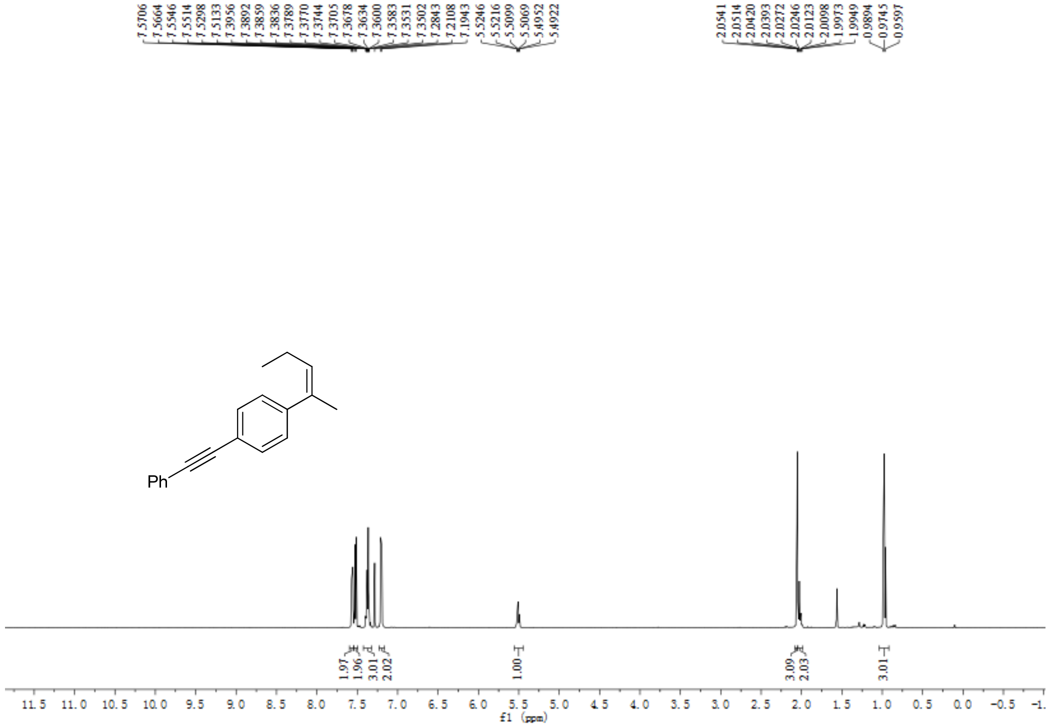

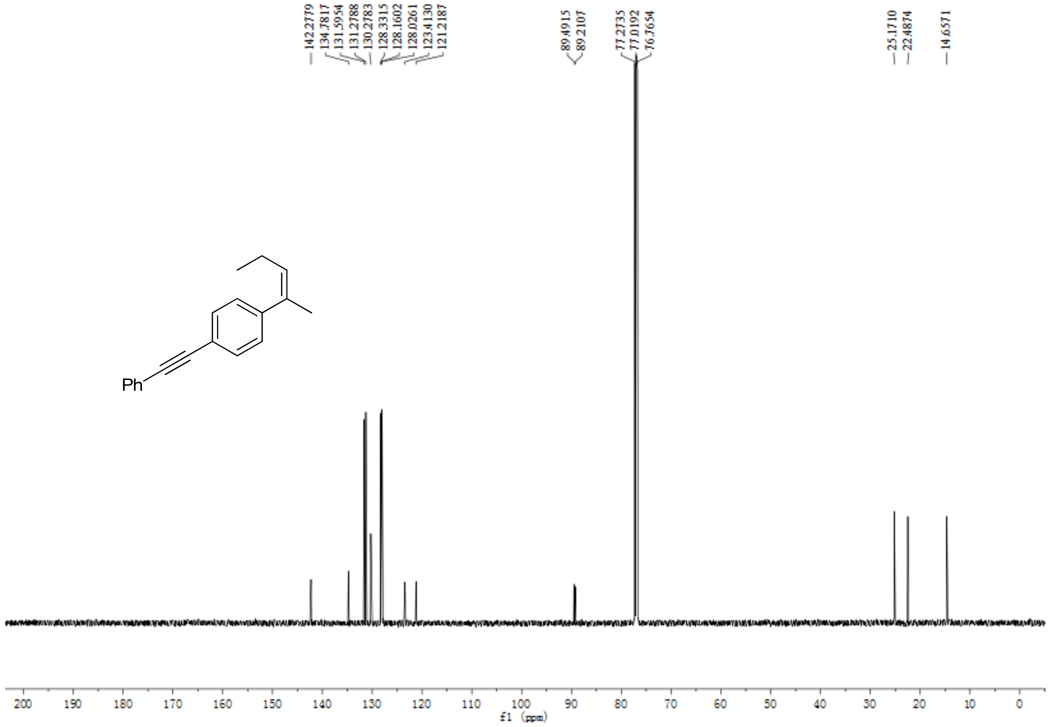

3in(*E*)

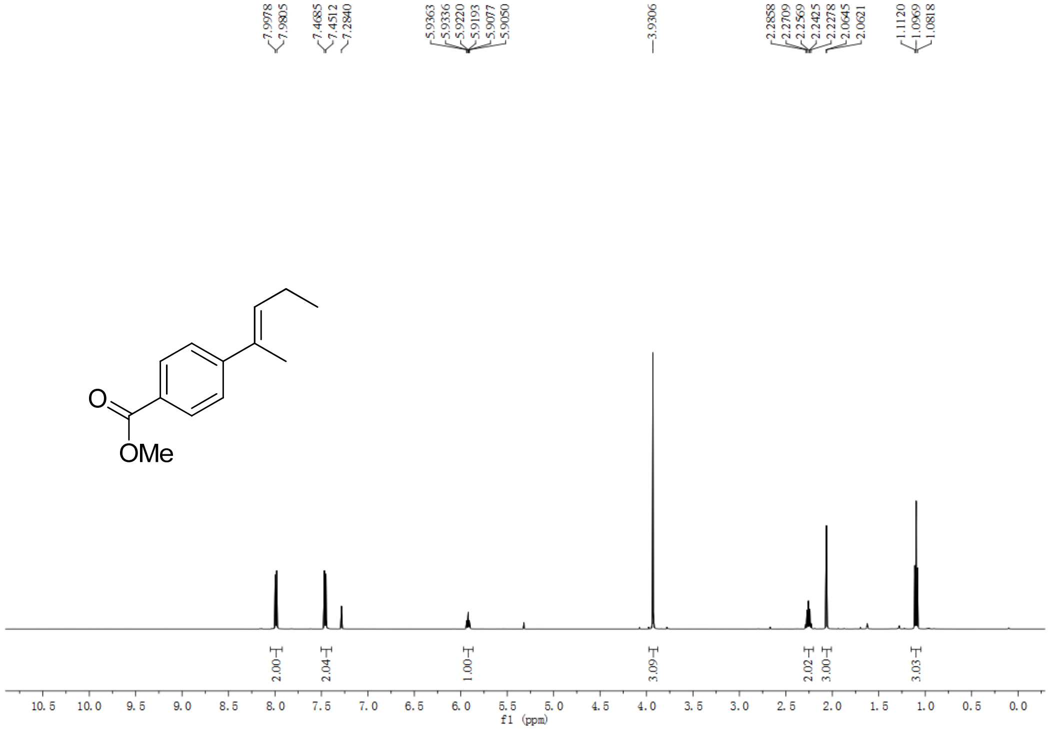
7.9978
7.9805
7.4685
7.4512
7.2840
5.9363
5.9336
5.9220
5.9193
5.9077
5.9050
3.9306
2.2858
2.2709
2.2569
2.2425
2.2278
2.0645
2.0621
1.1120
1.0969
1.0818
O
OMe
2.00
2.04
1.00
3.09
2.02
3.00
3.03
f1 (ppm)

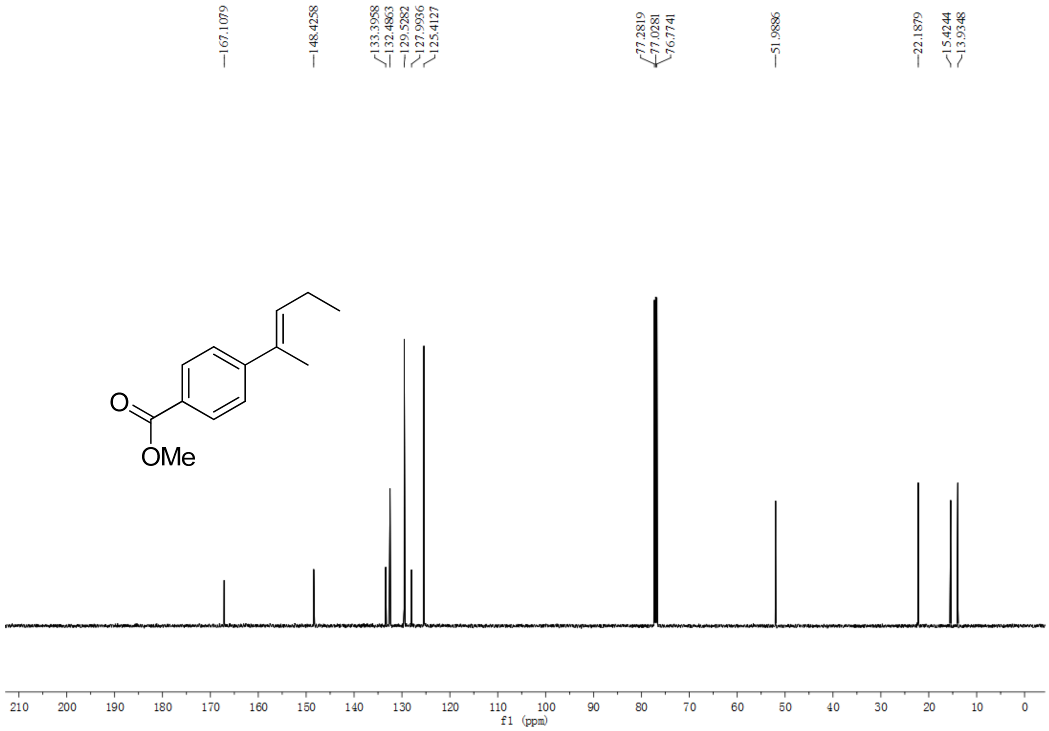
167.1079
148.4258
133.3958
132.4863
129.5282
127.9936
125.4127
77.2819
77.0281
76.7741
51.9886
22.1879
15.4244
13.9348
O
OMe
f1 (ppm)

3in(*Z*)

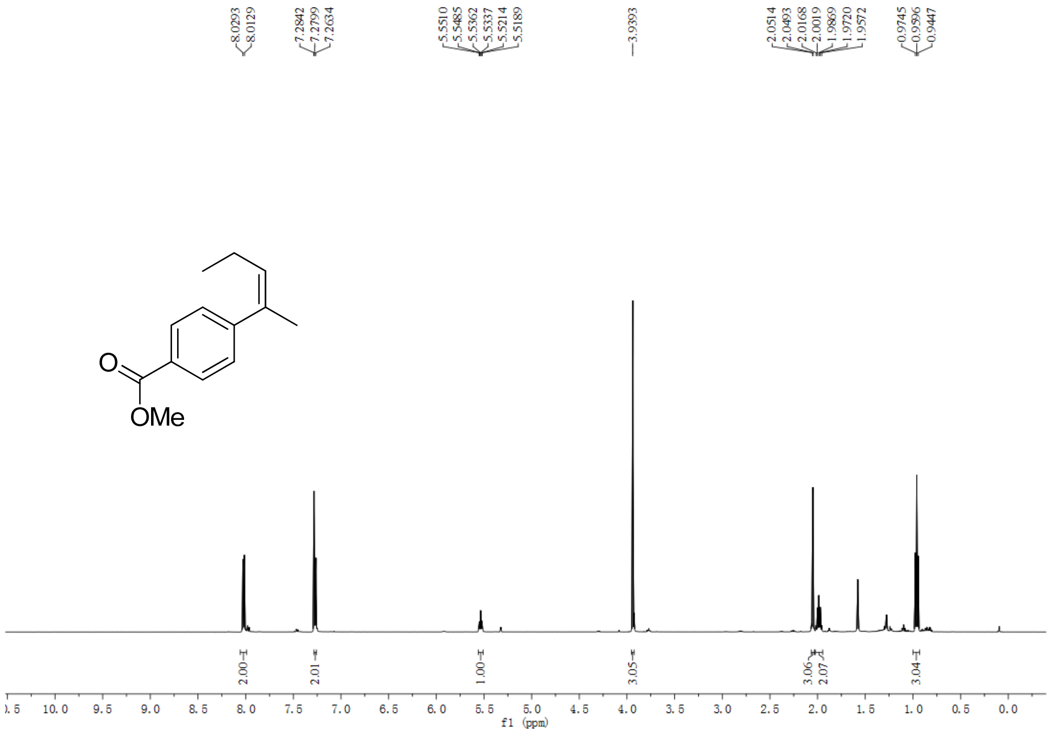
8.0293
8.0129
7.2842
7.2799
7.2634
5.5510
5.5485
5.5362
5.5337
5.5214
5.5189
3.9393
2.0514
2.0493
2.0168
2.0019
1.9869
1.9720
1.9572
0.9745
0.9596
0.9447
OMe
O
2.00
2.01
1.00
3.05
3.06
2.07
3.04
f1 (ppm)

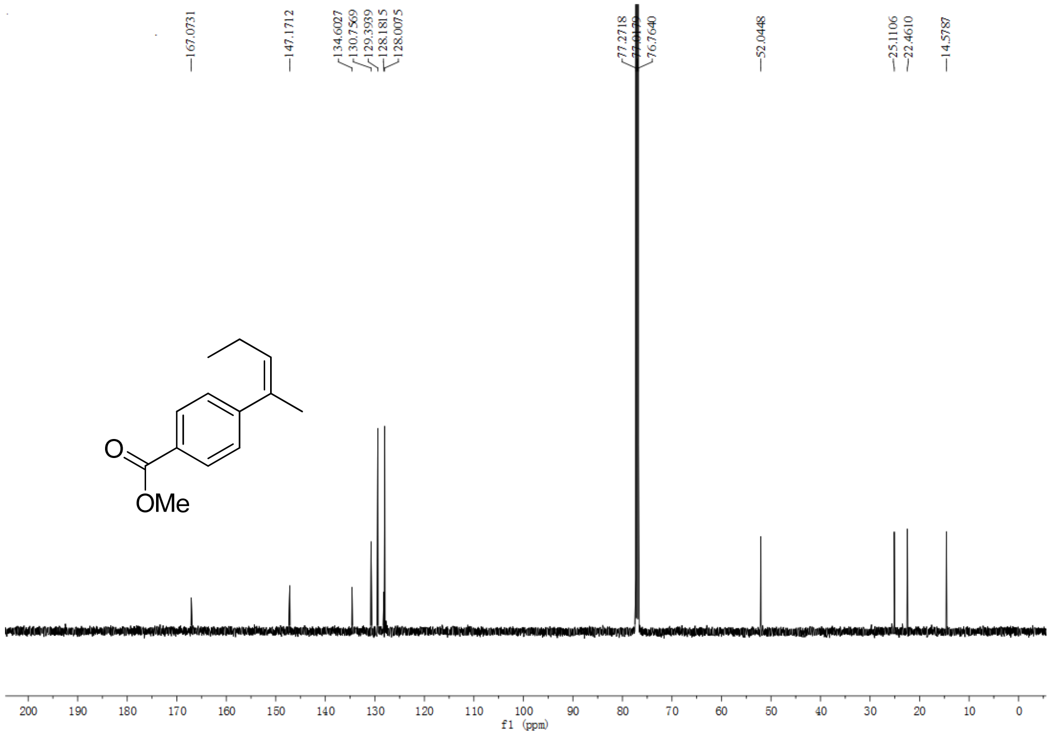
167.0731
147.1712
134.6027
130.7569
129.3939
128.1815
128.0075
77.2718
76.7640
52.0448
25.1106
22.4610
14.5787
O
OMe
f1 (ppm)

3io

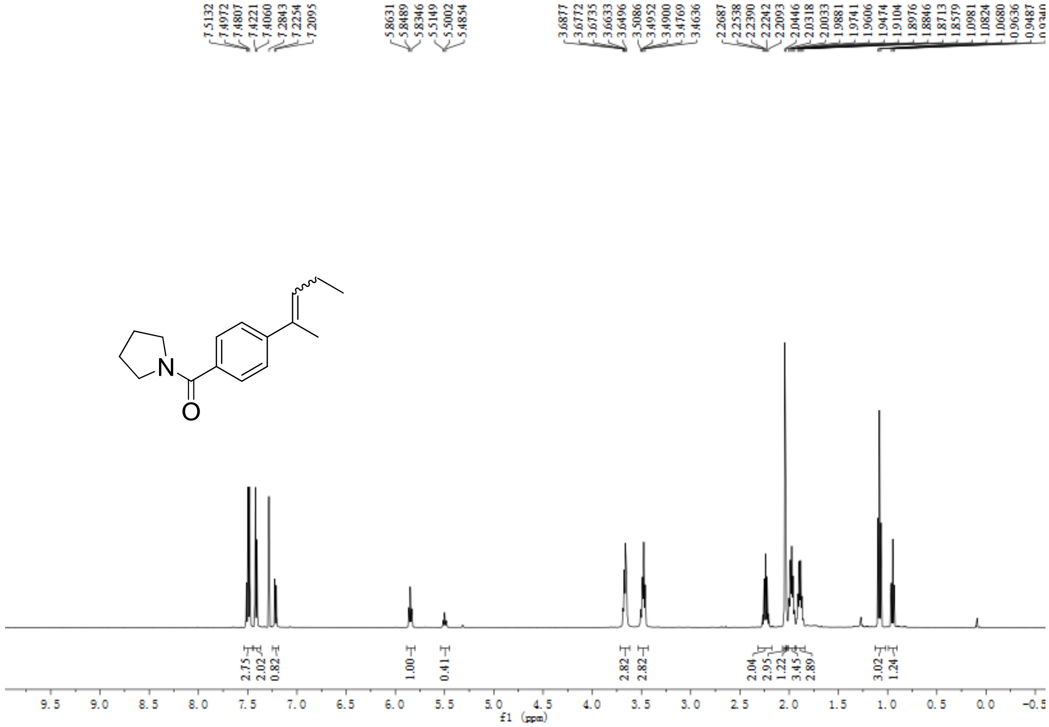

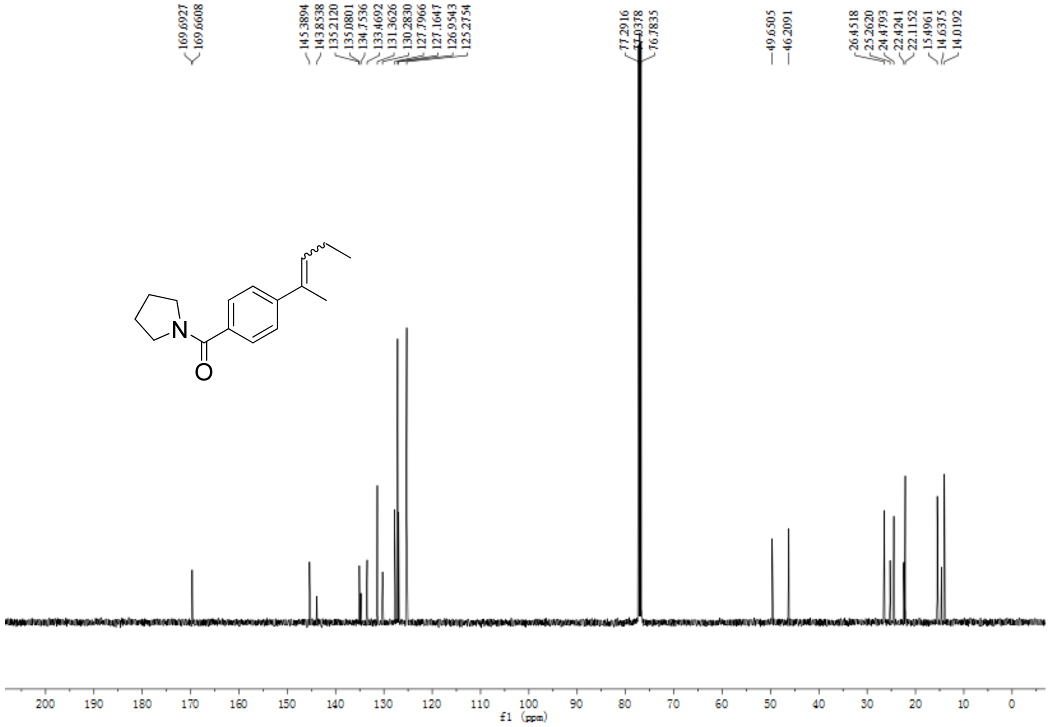

3ip(*E*)

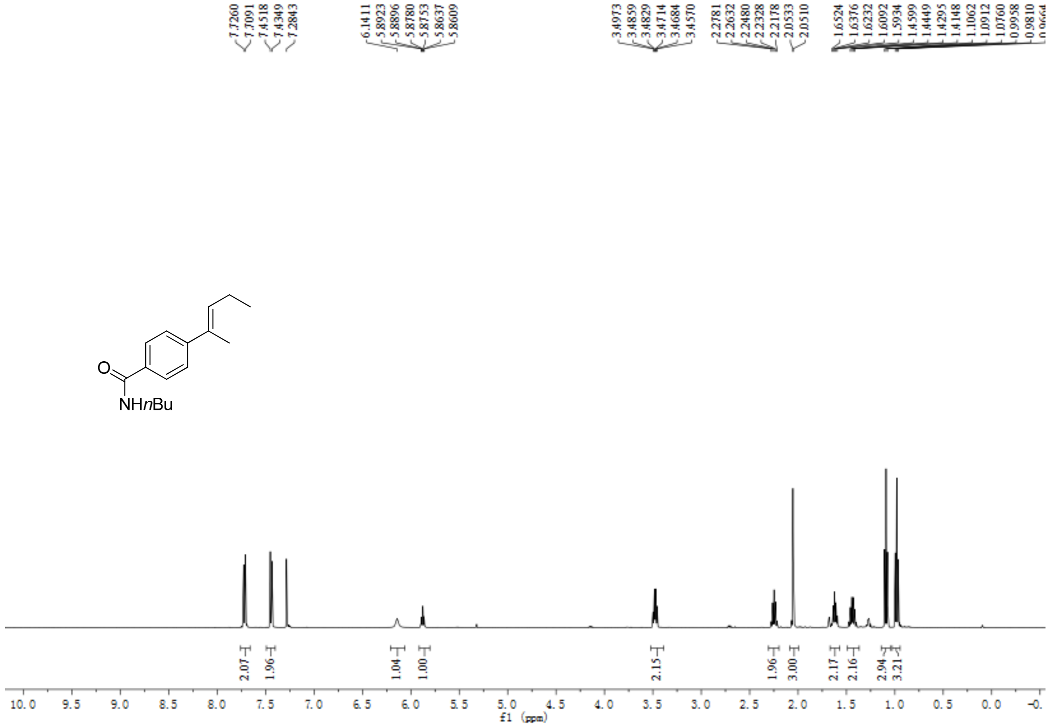

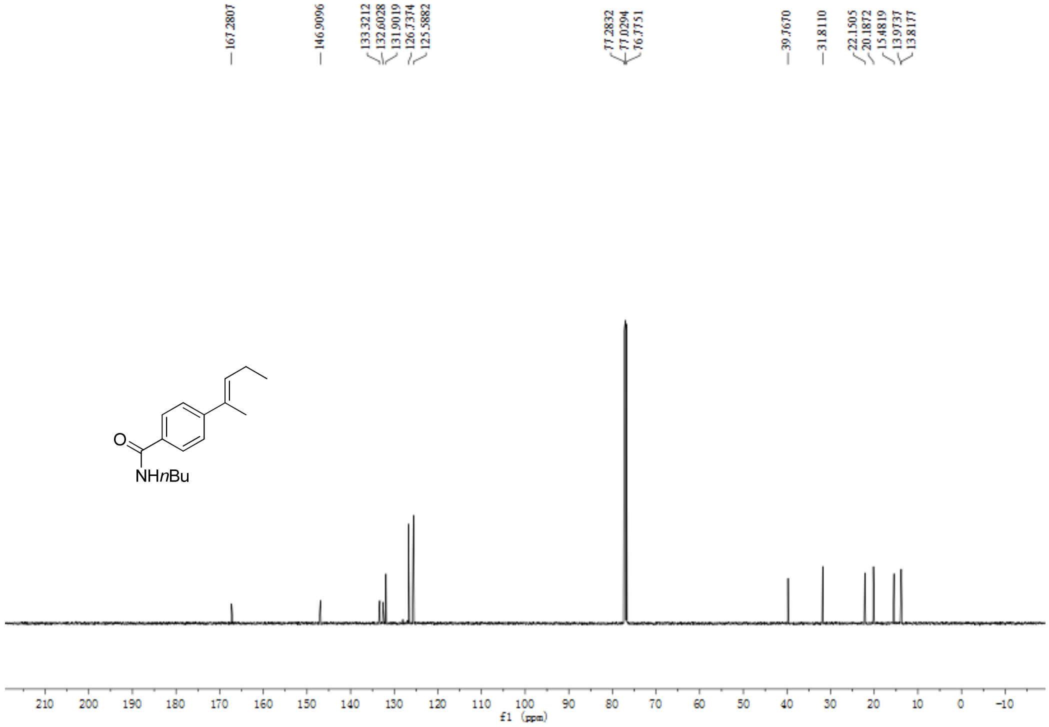

3ip(*Z*)

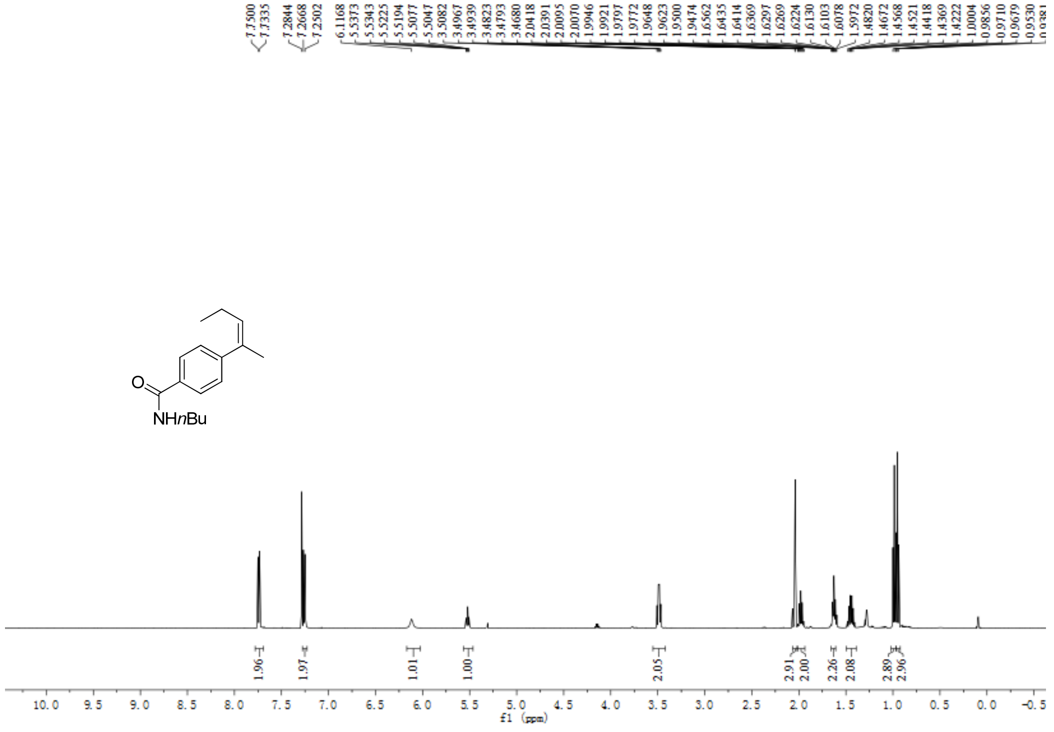

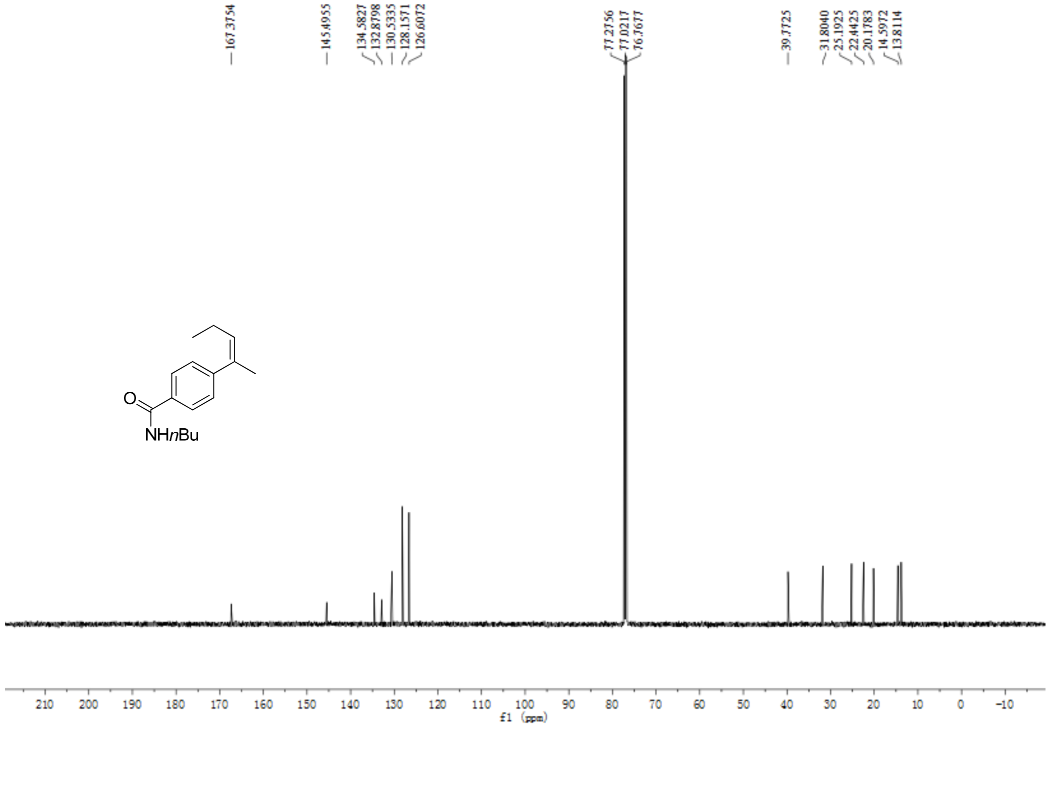

3j(*E*)

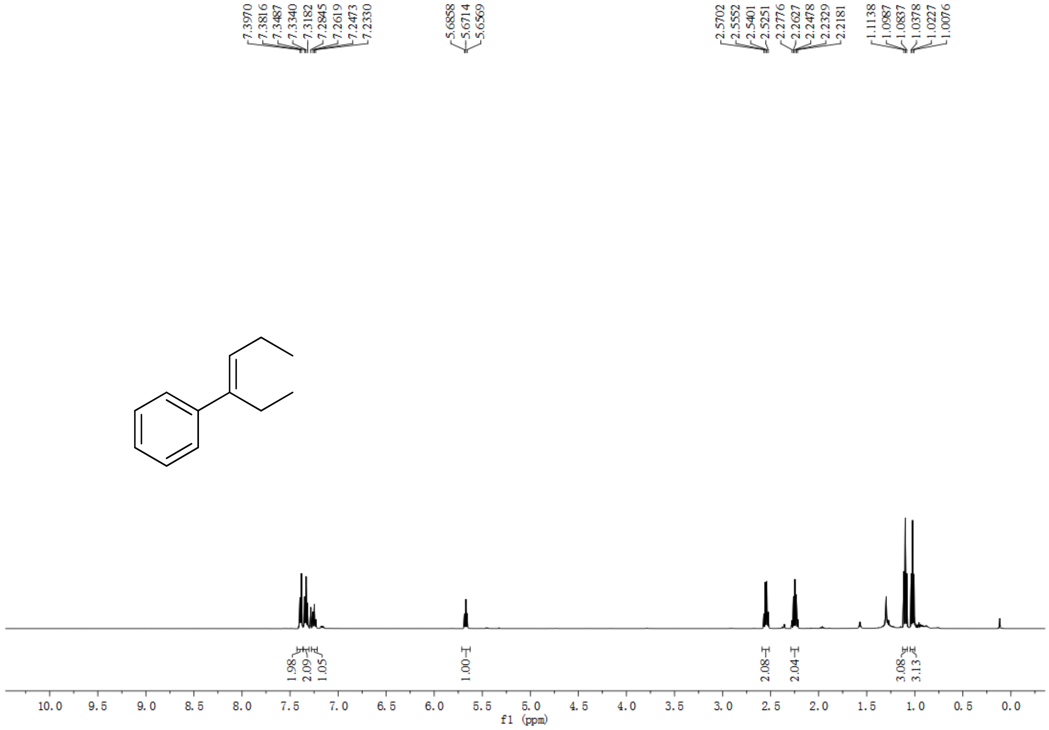

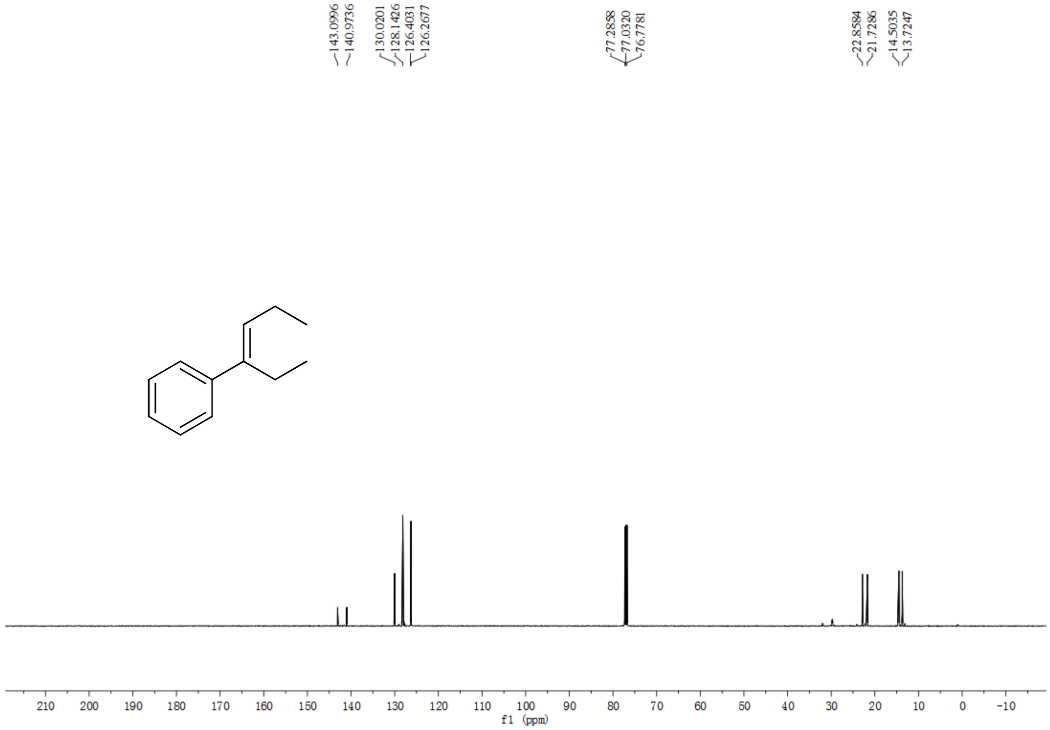

3j(*Z*)

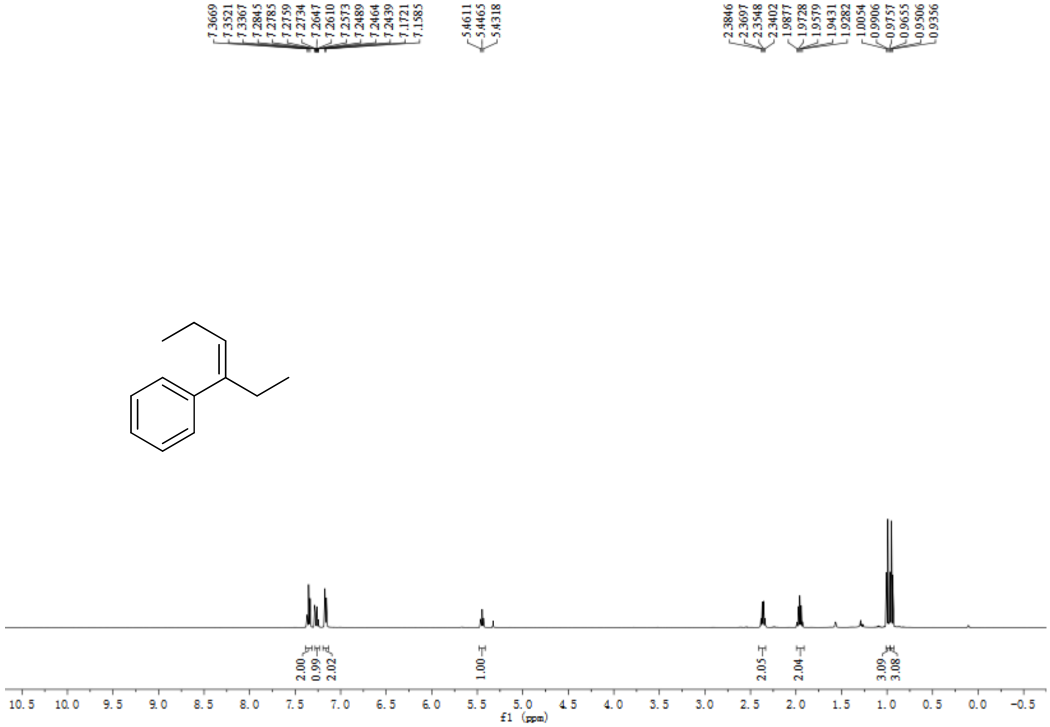
7.3669
7.3521
7.3367
7.2845
7.2785
7.2759
7.2734
7.2647
7.2610
7.2573
7.2489
7.2464
7.2439
7.1721
7.1585
5.4611
5.4465
5.4318
2.3846
2.3697
2.3548
2.3402
1.9877
1.9728
1.9579
1.9431
1.9282
1.0054
0.9906
0.9757
0.9655
0.9506
0.9356
2.00
0.99
2.02
1.00
2.05
2.04
3.09
3.08
10.5
10.0
9.5
9.0
8.5
8.0
7.5
7.0
6.5
6.0
5.5
5.0
4.5
4.0
3.5
3.0
2.5
2.0
1.5
1.0
0.5
0.0
-0.5
f1 (ppm)

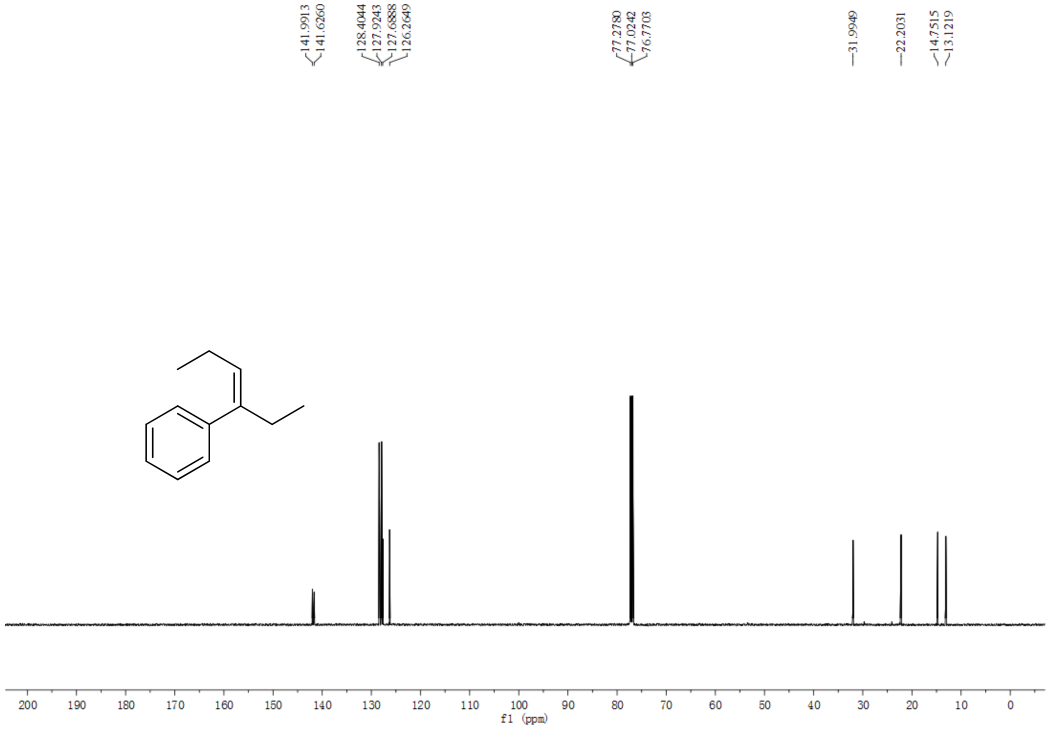
141.9913
141.6260
128.4044
127.9243
127.6888
126.2649
77.2780
77.0242
76.7703
31.9949
22.2031
14.7515
13.1219
200
190
180
170
160
150
140
130
120
110
100
90
80
70
60
50
40
30
20
10
0
f1 (ppm)

3k(*E*)

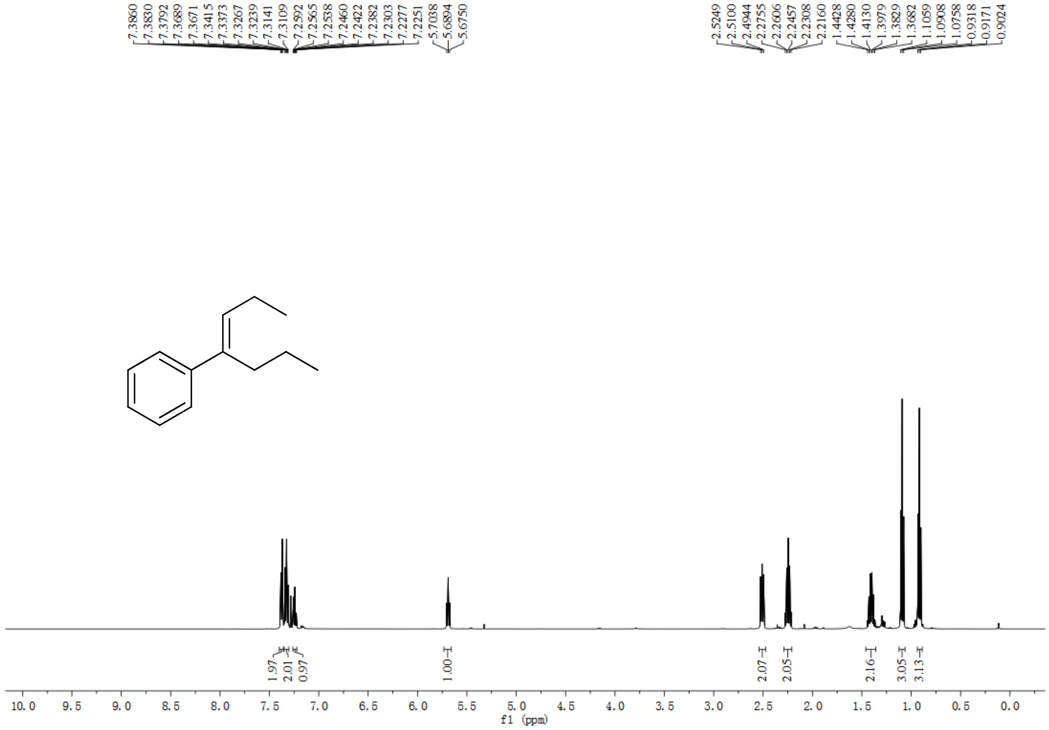

7.3860
7.3830
7.3792
7.3689
7.3671
7.3415
7.3373
7.3267
7.3239
7.3141
7.3109
7.2592
7.2565
7.2538
7.2460
7.2422
7.2382
7.2303
7.2277
7.2251
5.7038
5.6894
5.6750
2.5249
2.5100
2.4944
2.2755
2.2606
2.2457
2.2308
2.2160
1.4428
1.4280
1.4130
1.3979
1.3829
1.3682
1.1059
1.0908
1.0758
0.9318
0.9171
0.9024
1.97
2.01
0.97
1.00
2.07
2.05
2.16
3.05
3.13
10.0 9.5 9.0 8.5 8.0 7.5 7.0 6.5 6.0 5.5 5.0 4.5 4.0 3.5 3.0 2.5 2.0 1.5 1.0 0.5 0.0
f1 (ppm)

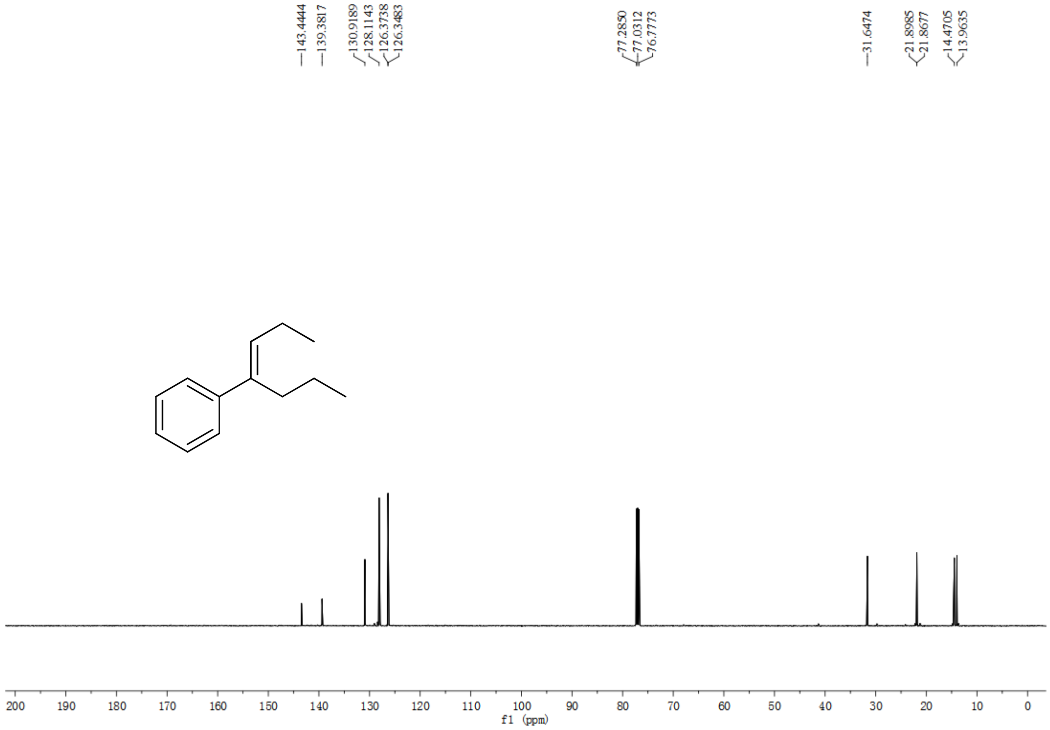

143.4444
139.3817
130.9189
128.1143
126.3738
126.3483
77.2850
77.0312
76.7773
31.6474
21.8985
21.8677
14.4705
13.9635
200 190 180 170 160 150 140 130 120 110 100 90 80 70 60 50 40 30 20 10 0
f1 (ppm)

3k(*Z*)

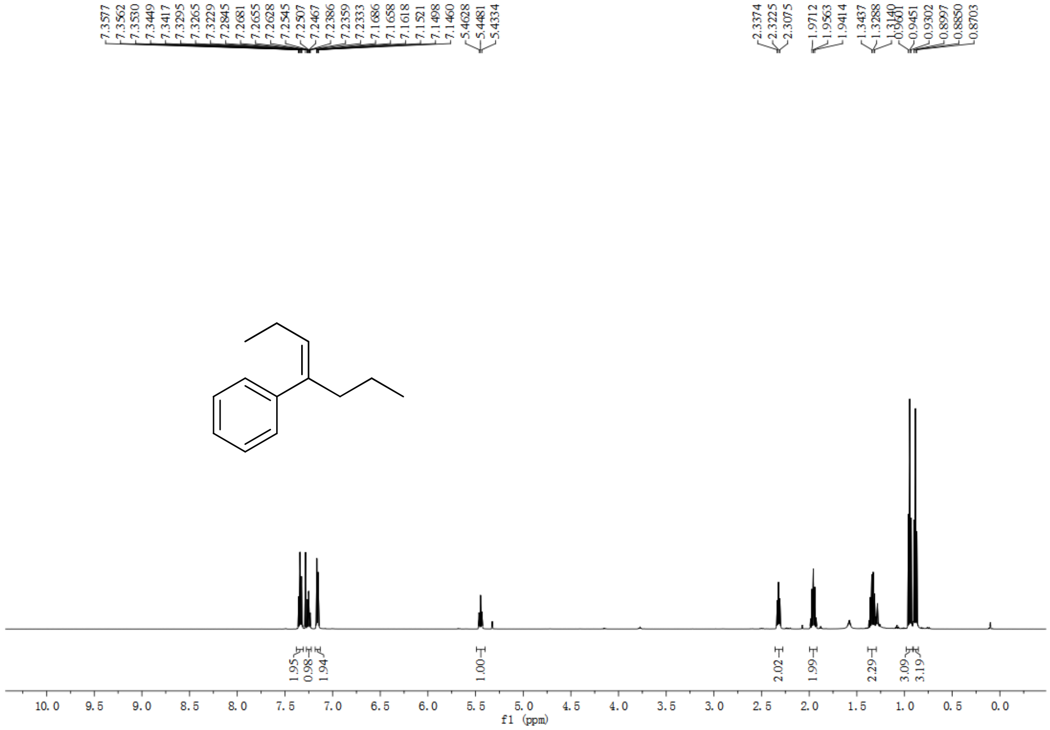
7.3577
7.3562
7.3530
7.3449
7.3417
7.3295
7.3265
7.3229
7.2845
7.2681
7.2655
7.2628
7.2545
7.2507
7.2467
7.2386
7.2359
7.2333
7.1686
7.1658
7.1618
7.1521
7.1498
7.1460
5.4628
5.4481
5.4334
2.3374
2.3225
2.3075
1.9712
1.9563
1.9414
1.3437
1.3288
0.9451
0.9302
0.8997
0.8850
0.8703
1.95
0.98
1.94
1.00
2.02
1.99
2.29
3.09
3.19
f1 (ppm)

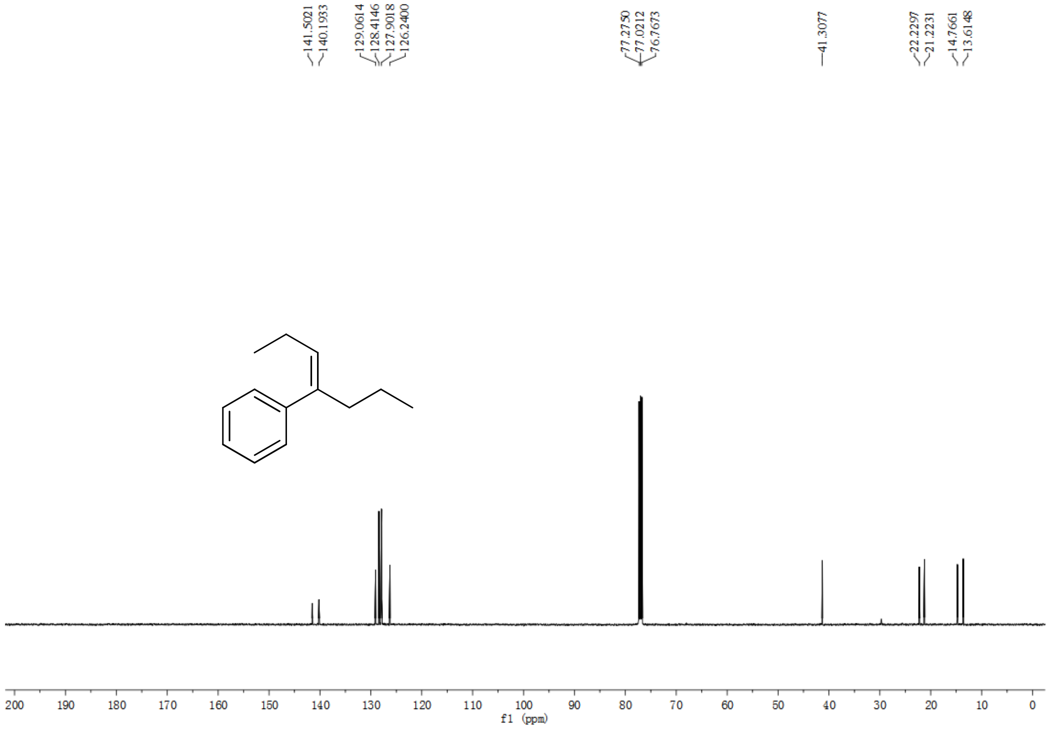
141.5021
140.1933
129.0614
128.4146
127.9018
126.2400
77.2750
77.0212
76.7673
41.3077
22.2297
21.2231
14.7661
13.6148
f1 (ppm)

3l(*E*)

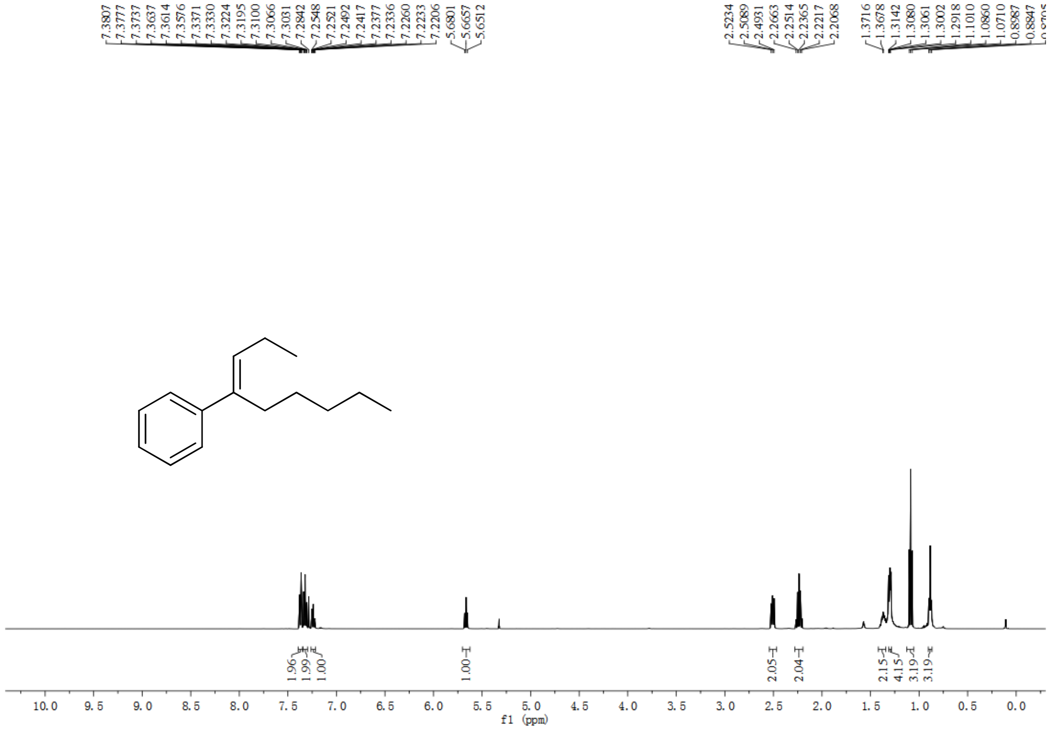
7.3807 7.3777 7.3737 7.3637 7.3614 7.3576 7.3371 7.3330 7.3224 7.3195 7.3100 7.3066 7.3031 7.2842 7.2548 7.2521 7.2492 7.2417 7.2377 7.2336 7.2260 7.2233 7.2206 5.6801 5.6657 5.6512
2.5234 2.5089 2.4931 2.2663 2.2514 2.2365 2.2217 2.2068
1.3716 1.3678 1.3142 1.3080 1.3061 1.3002 1.2918 1.1010 1.0860 1.0710 0.8987 0.8847
1.96 1.99 1.00 1.00 2.05 2.04 2.15 4.15 3.19 3.19
10.0 9.5 9.0 8.5 8.0 7.5 7.0 6.5 6.0 5.5 5.0 4.5 4.0 3.5 3.0 2.5 2.0 1.5 1.0 0.5 0.0
f1 (ppm)

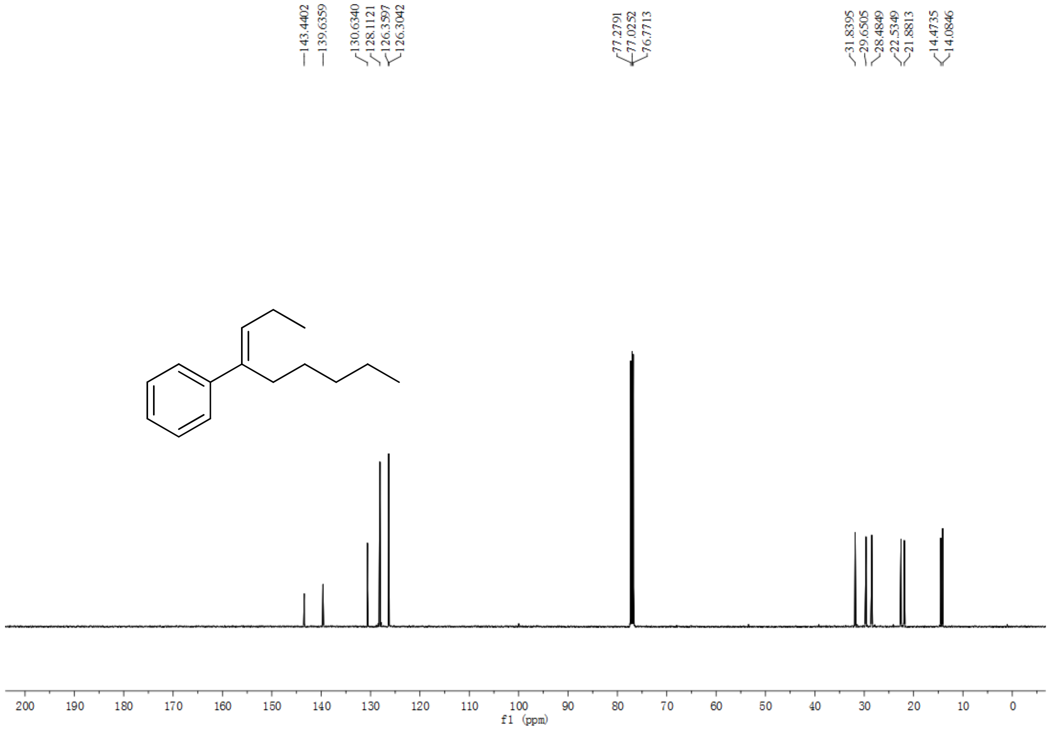
143.4402 139.6359 130.6340 128.1121 126.3597 126.3042
77.2791 77.0252 76.7713
31.8395 29.6505 28.4849 22.5349 21.8813 14.4735 14.0846
200 190 180 170 160 150 140 130 120 110 100 90 80 70 60 50 40 30 20 10 0
f1 (ppm)

3l(Z)

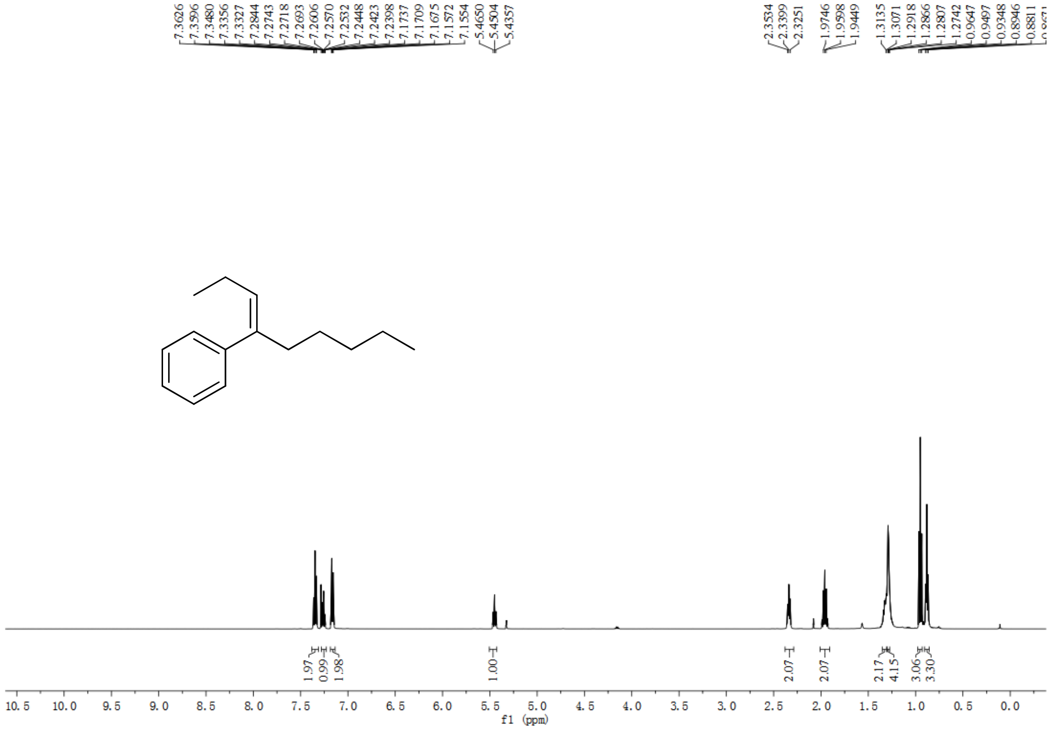
7.3626 7.3596 7.3480 7.3356 7.3327 7.2844 7.2743 7.2718 7.2693 7.2606 7.2570 7.2532 7.2448 7.2423 7.2398 7.1737 7.1709 7.1675 7.1572 7.1554 5.4650 5.4504 5.4357
2.3534 2.3399 2.3251 1.9746 1.9598 1.9449 1.3135 1.3071 1.2918 1.2866 1.2807 1.2742 0.9647 0.9497 0.9348 0.8946 0.8811
1.97 0.99 1.98 1.00 2.07 2.07 2.17 4.15 3.06 3.30
10.5 10.0 9.5 9.0 8.5 8.0 7.5 7.0 6.5 6.0 5.5 5.0 4.5 4.0 3.5 3.0 2.5 2.0 1.5 1.0 0.5 0.0
f1 (ppm)

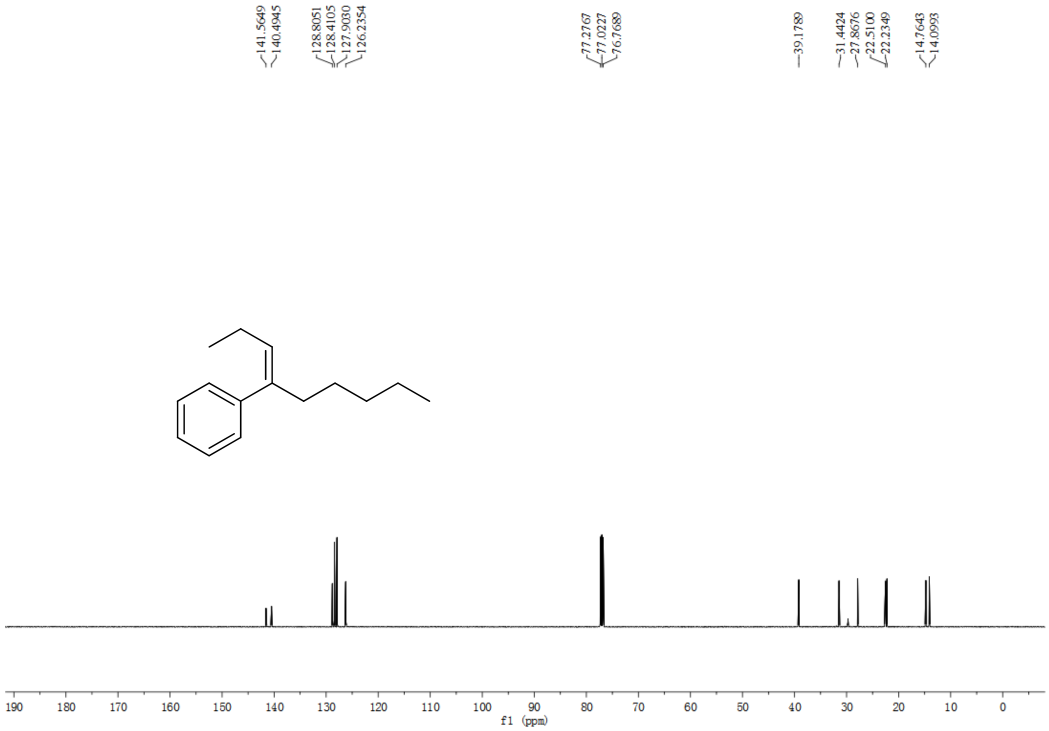
141.5649 140.4945 128.8051 128.4105 127.9030 126.2354 77.2767 77.0227 76.7689 39.1789 31.4424 27.8676 22.5100 22.2349 14.7643 14.0993
190 180 170 160 150 140 130 120 110 100 90 80 70 60 50 40 30 20 10 0
f1 (ppm)

3m

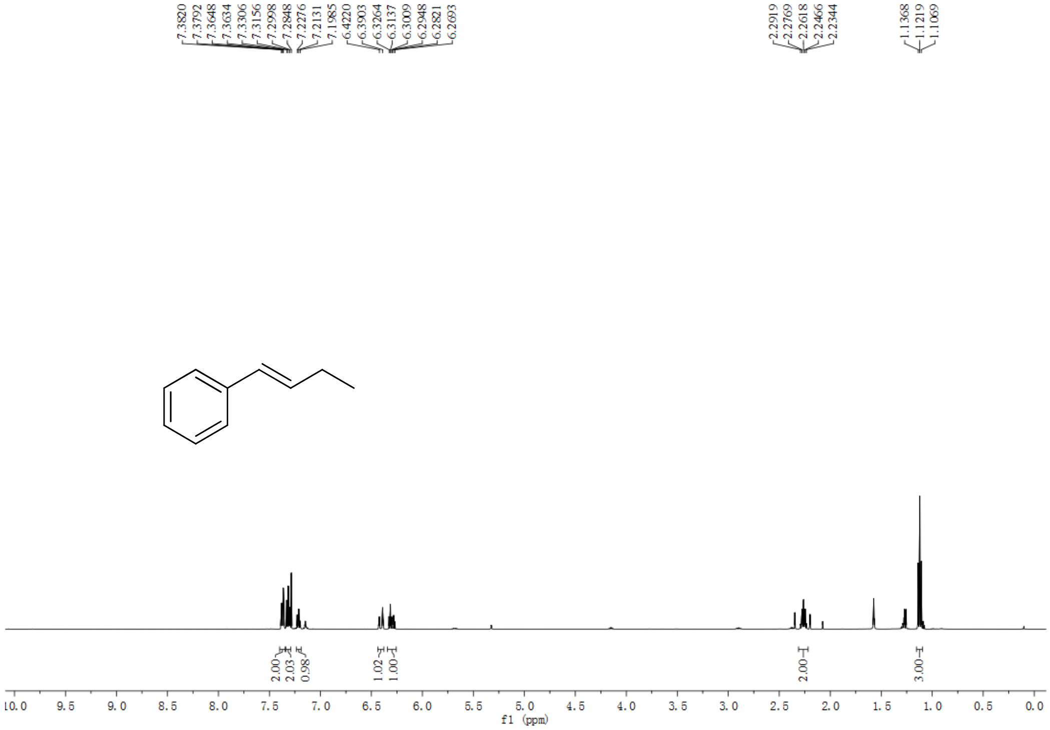
7.3820
7.3792
7.3648
7.3634
7.3306
7.3156
7.2998
7.2848
7.2276
7.2131
7.1985
6.4220
6.3903
6.3264
6.3137
6.3009
6.2948
6.2821
6.2693
2.2919
2.2769
2.2618
2.2466
2.2344
1.1368
1.1219
1.1069
2.00
2.03
0.98
1.02
1.00
2.00
3.00
f1 (ppm)

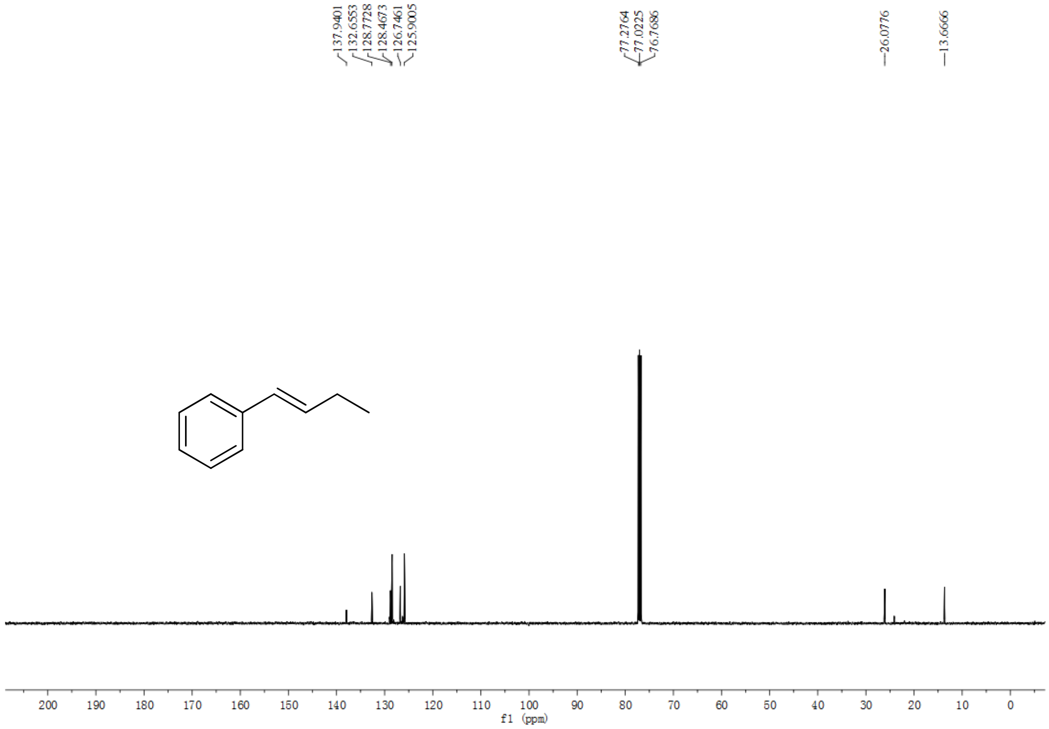
137.9401
132.6553
128.7728
128.4673
126.7461
125.9005
77.2764
77.0225
76.7686
26.0776
13.6666
f1 (ppm)

3o

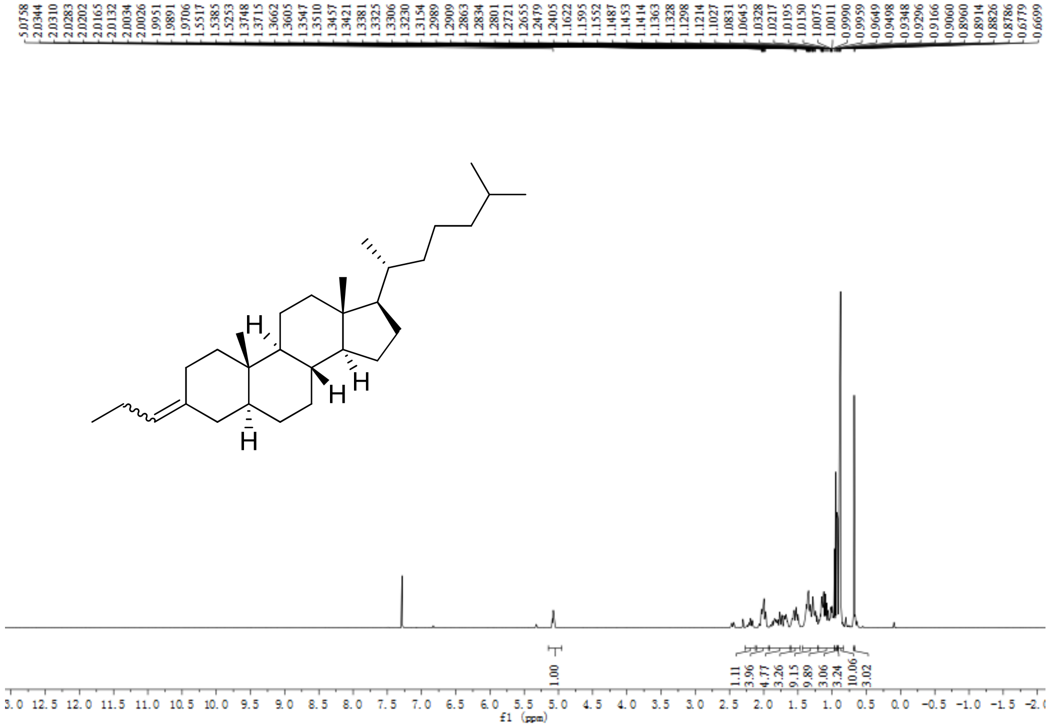

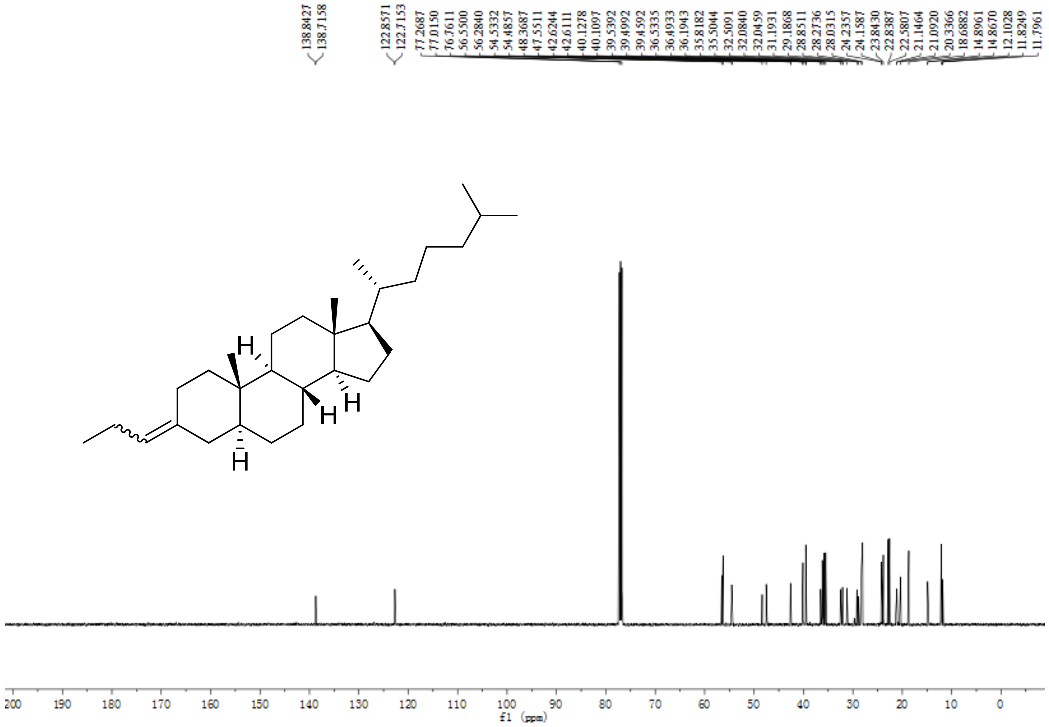

3p

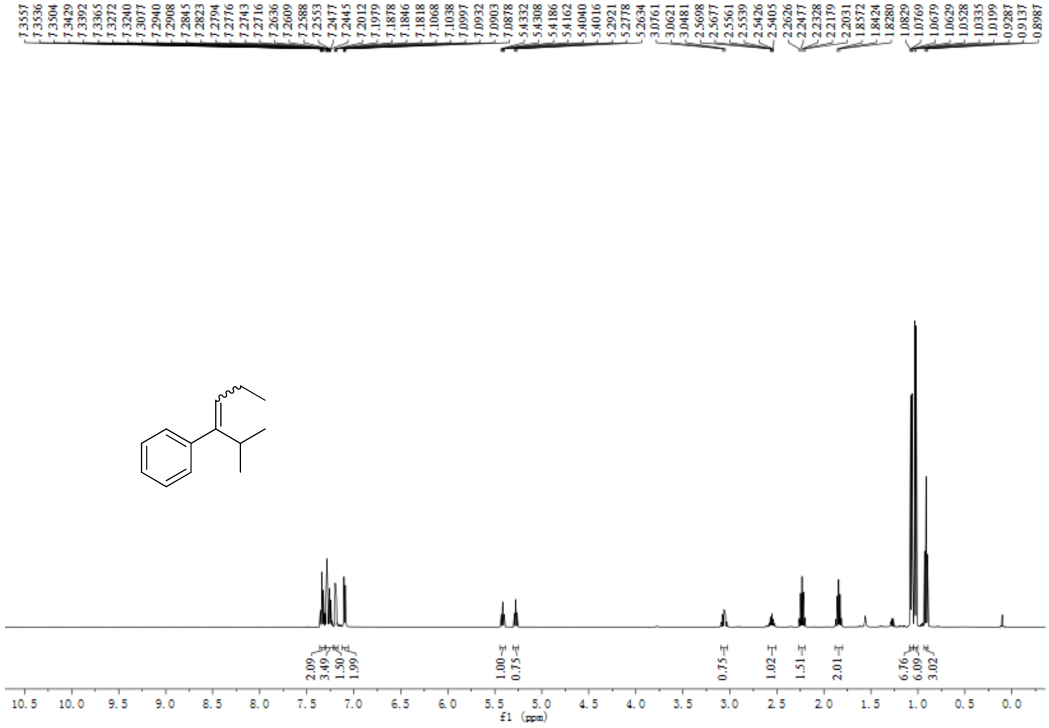
7.3557
7.3536
7.3504
7.3429
7.3392
7.3365
7.3272
7.3240
7.3077
7.2940
7.2908
7.2845
7.2823
7.2794
7.2776
7.2743
7.2716
7.2636
7.2609
7.2588
7.2553
7.2477
7.2445
7.2012
7.1979
7.1878
7.1846
7.1818
7.1068
7.1038
7.0997
7.0932
7.0903
7.0878
5.4332
5.4308
5.4186
5.4162
5.4040
5.4016
5.2921
5.2778
5.2634
3.0761
3.0621
3.0481
2.5698
2.5677
2.5561
2.5539
2.5426
2.5405
2.2626
2.2477
2.2328
2.2179
2.2031
1.8572
1.8424
1.8280
1.0829
1.0769
1.0679
1.0629
1.0528
1.0335
1.0199
0.9287
0.9137
0.8987
2.09
3.49
1.50
1.99
1.00
0.75
0.75
1.02
1.51
2.01
6.76
6.09
3.02
10.5 10.0 9.5 9.0 8.5 8.0 7.5 7.0 6.5 6.0 5.5 5.0 4.5 4.0 3.5 3.0 2.5 2.0 1.5 1.0 0.5 0.0
f1 (ppm)

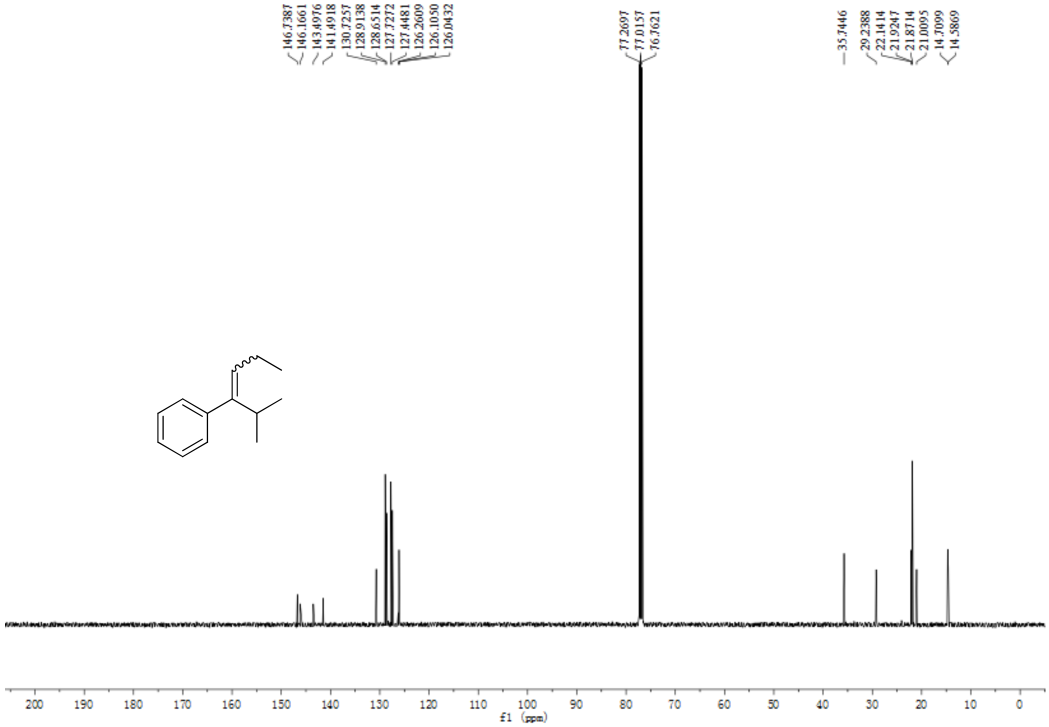
146.7387
146.1661
143.4976
141.4918
130.7257
128.9138
128.6514
127.7272
127.4481
126.2609
126.1050
126.0432
77.2697
77.0157
76.7621
35.7446
29.2388
22.1414
21.9247
21.8714
21.0095
14.7099
14.5869
200 190 180 170 160 150 140 130 120 110 100 90 80 70 60 50 40 30 20 10 0
f1 (ppm)

3q

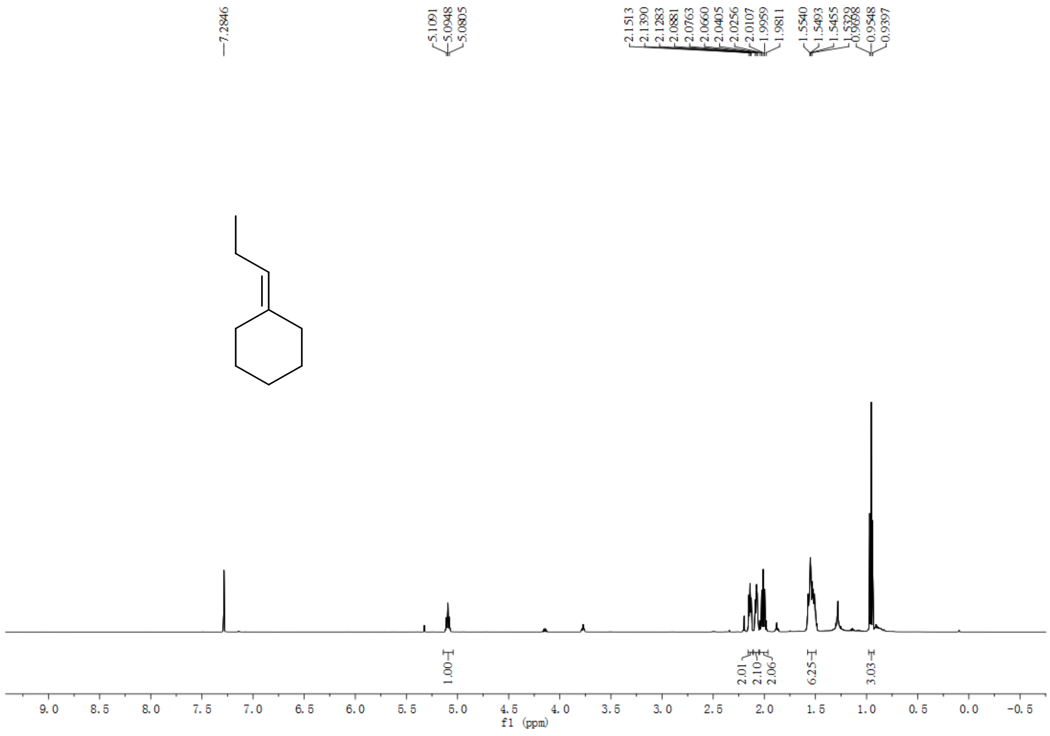

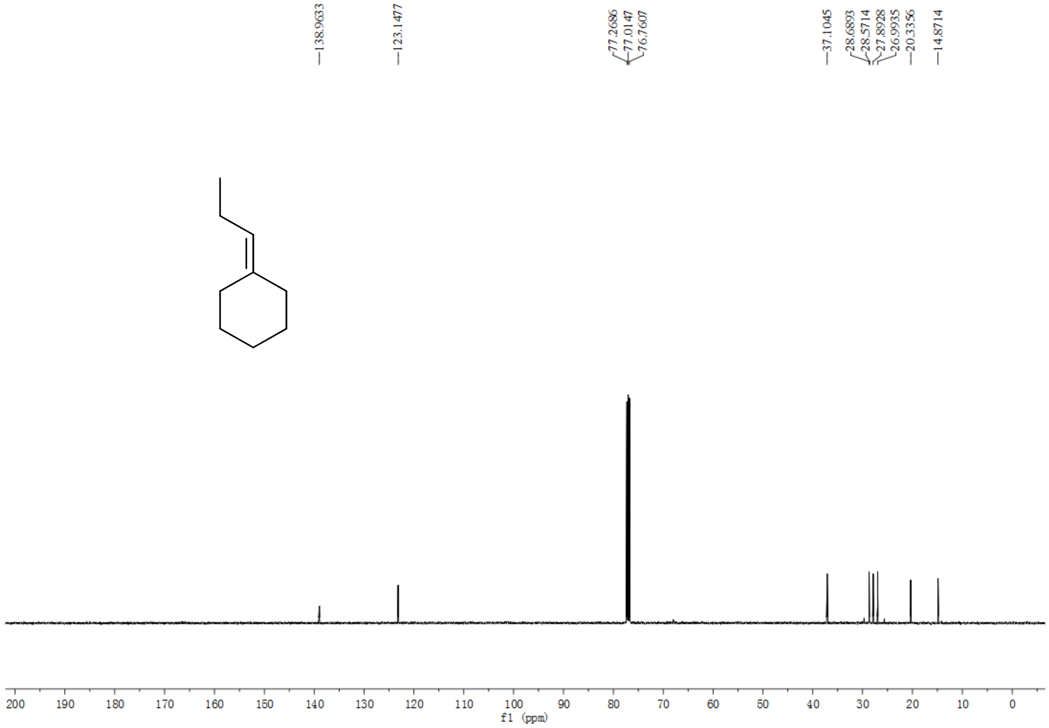

3r

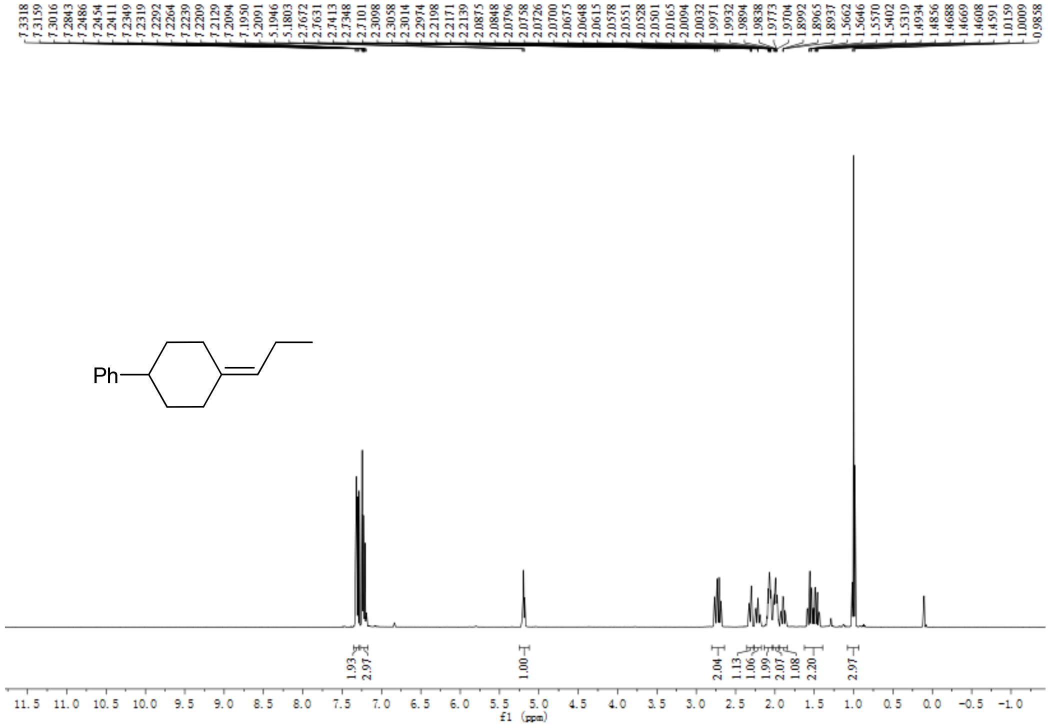
7.3318
7.3159
7.3016
7.2843
7.2486
7.2454
7.2411
7.2349
7.2319
7.2292
7.2264
7.2239
7.2209
7.2129
7.2094
7.1950
5.2091
5.1946
5.1803
2.7672
2.7631
2.7413
2.7348
2.7101
2.3098
2.3058
2.3014
2.2974
2.2198
2.2171
2.2139
2.0875
2.0848
2.0796
2.0758
2.0726
2.0700
2.0675
2.0648
2.0615
2.0578
2.0551
2.0528
2.0501
2.0165
2.0094
2.0032
1.9971
1.9932
1.9894
1.9838
1.9773
1.9704
1.8992
1.8965
1.8937
1.5662
1.5646
1.5570
1.5402
1.5319
1.4934
1.4856
1.4688
1.4669
1.4608
1.4591
1.0159
1.0009
0.9858
Ph
1.93
2.97
1.00
2.04
1.13
1.06
1.99
2.07
1.08
2.20
2.97
f1 (ppm)

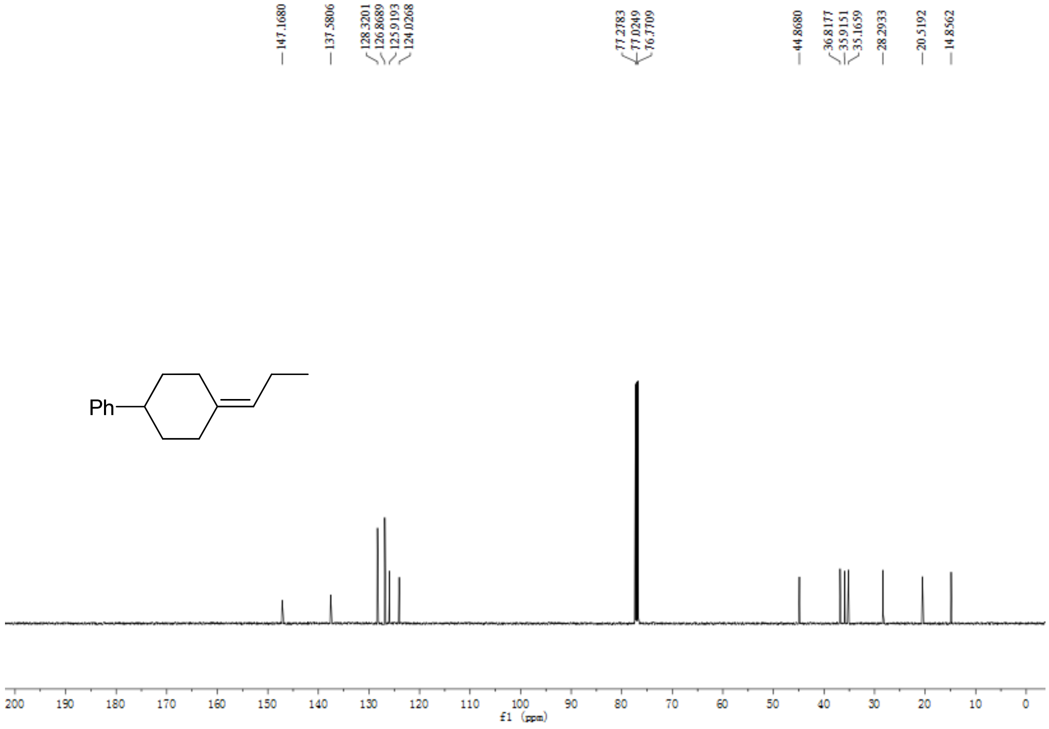
147.1680
137.5806
128.3201
126.8689
125.9193
124.0268
77.2783
77.0249
76.7709
44.8680
36.8177
35.9151
35.1659
28.2933
20.5192
14.8562
Ph
f1 (ppm)

4a

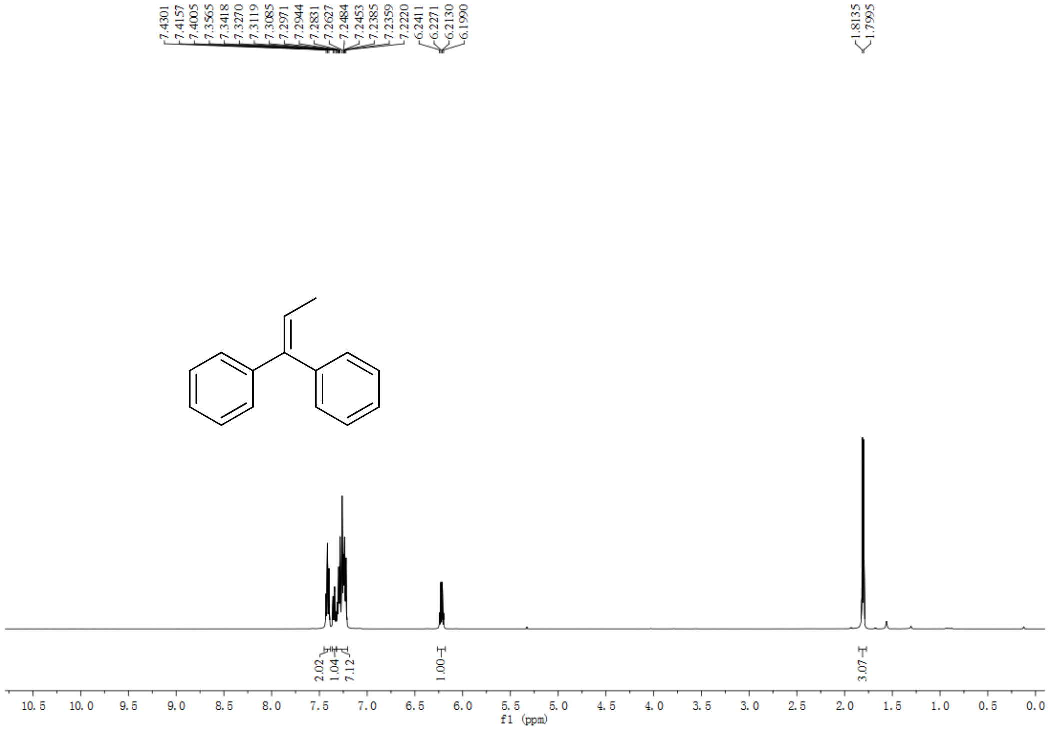
7.4301
7.4157
7.4005
7.3565
7.3418
7.3270
7.3119
7.3085
7.2971
7.2944
7.2831
7.2627
7.2484
7.2453
7.2385
7.2359
7.2220
6.2411
6.2271
6.2130
6.1990
1.8135
1.7995
2.02
1.04
7.12
1.00
3.07
10.5 10.0 9.5 9.0 8.5 8.0 7.5 7.0 6.5 6.0 5.5 5.0 4.5 4.0 3.5 3.0 2.5 2.0 1.5 1.0 0.5 0.0
f1 (ppm)

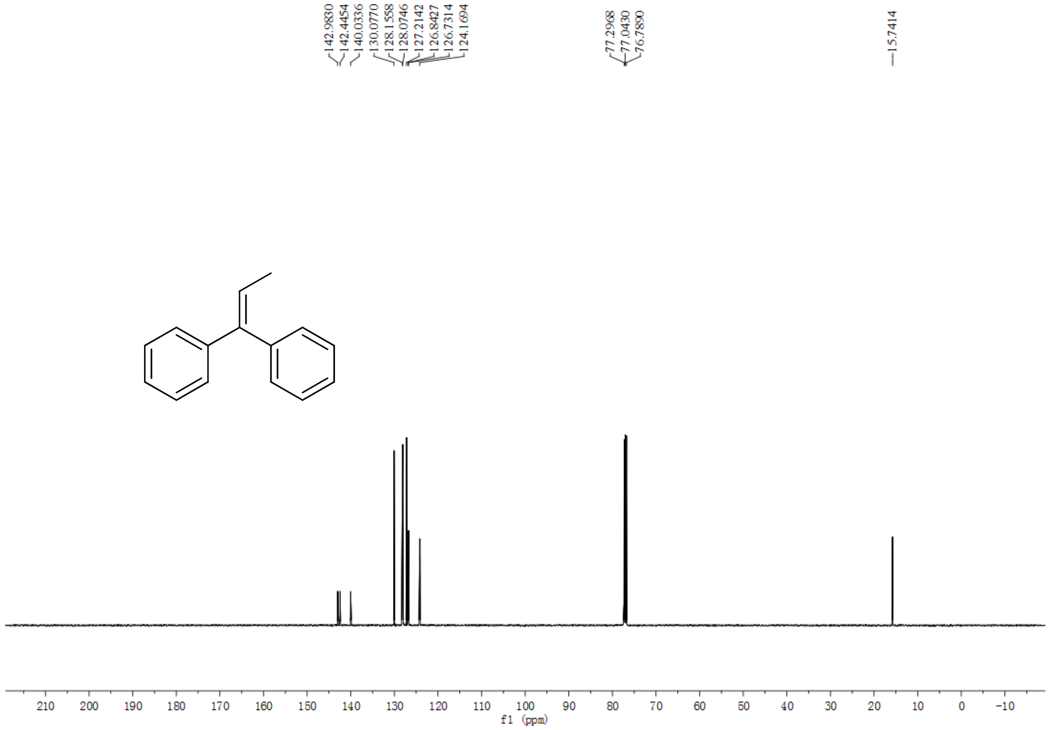
142.9830
142.4454
140.0336
130.0770
128.1558
128.0746
127.2142
126.8427
126.7314
124.1694
77.2968
77.0430
76.7890
15.7414
210 200 190 180 170 160 150 140 130 120 110 100 90 80 70 60 50 40 30 20 10 0 -10
f1 (ppm)

4b

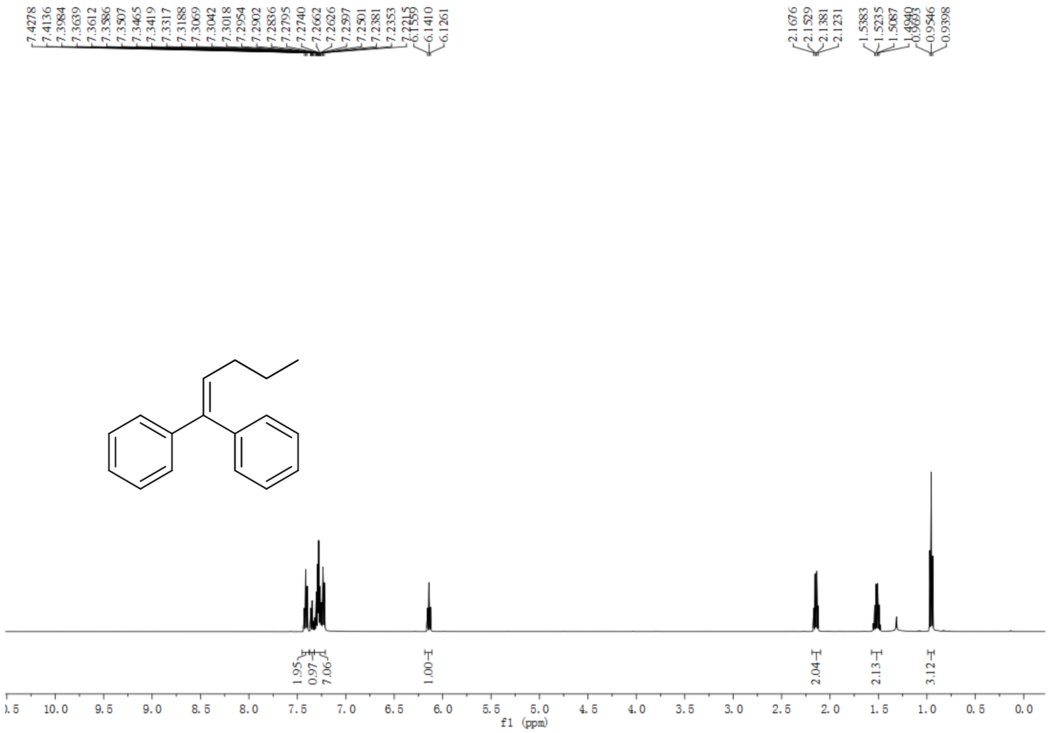
7.4278
7.4136
7.3984
7.3639
7.3612
7.3586
7.3507
7.3465
7.3419
7.3317
7.3188
7.3069
7.3042
7.3018
7.2954
7.2902
7.2836
7.2795
7.2740
7.2662
7.2626
7.2297
7.2501
7.2381
7.2353
7.2215
6.1559
6.1410
6.1261
2.1676
2.1529
2.1381
2.1231
1.5383
1.5235
1.5087
1.4940
0.9693
0.9546
0.9398
1.95
0.97
7.06
1.00
2.04
2.13
3.12
f1 (ppm)

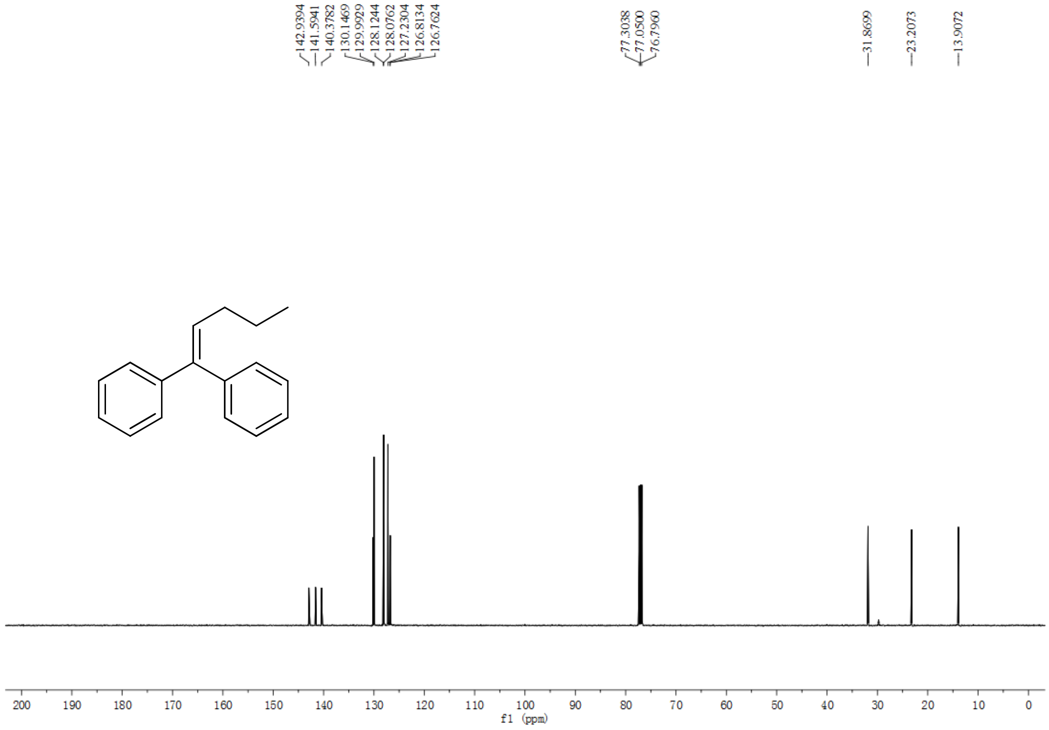
142.9394
141.5941
140.3782
130.1469
129.9929
128.1244
128.0762
127.2304
126.8134
126.7624
77.3038
77.0500
76.7960
31.8699
23.2073
13.9072
f1 (ppm)

4c

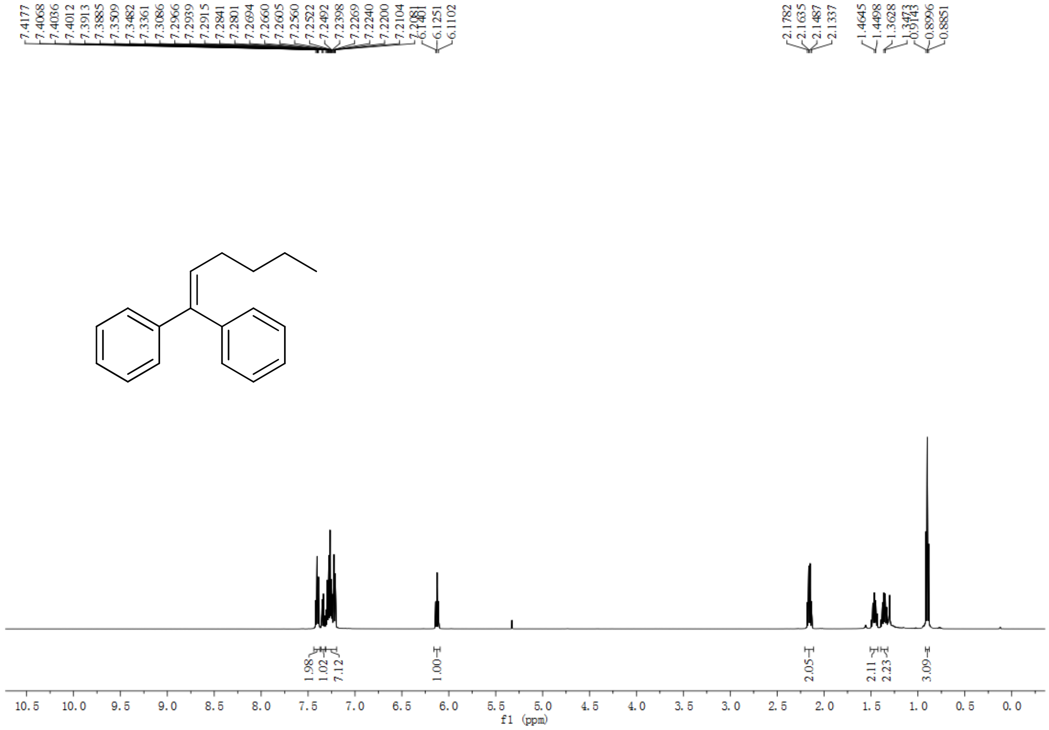
7.4177
7.4068
7.4036
7.4012
7.3913
7.3885
7.3509
7.3482
7.3361
7.3086
7.2966
7.2939
7.2915
7.2841
7.2801
7.2694
7.2660
7.2605
7.2560
7.2522
7.2492
7.2398
7.2269
7.2240
7.2200
7.2104
6.1251
6.1102
2.1782
2.1635
2.1487
2.1337
1.4645
1.4498
1.3628
0.8996
0.8851
1.98
1.02
7.12
1.00
2.05
2.11
2.23
3.09
10.5 10.0 9.5 9.0 8.5 8.0 7.5 7.0 6.5 6.0 5.5 5.0 4.5 4.0 3.5 3.0 2.5 2.0 1.5 1.0 0.5 0.0
f1 (ppm)

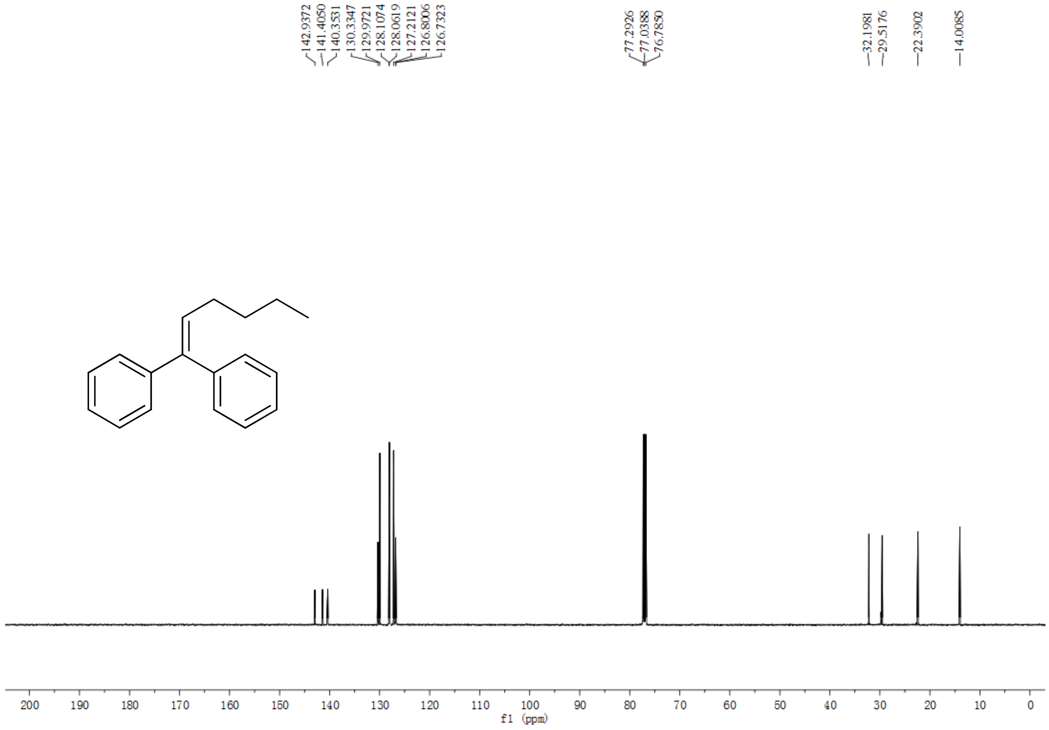
142.9372
141.4050
140.3531
130.3347
129.9721
128.1074
128.0619
127.2121
126.8006
126.7323
77.2926
77.0388
76.7850
32.1981
29.5176
22.3902
14.0085
200 190 180 170 160 150 140 130 120 110 100 90 80 70 60 50 40 30 20 10 0
f1 (ppm)

4d

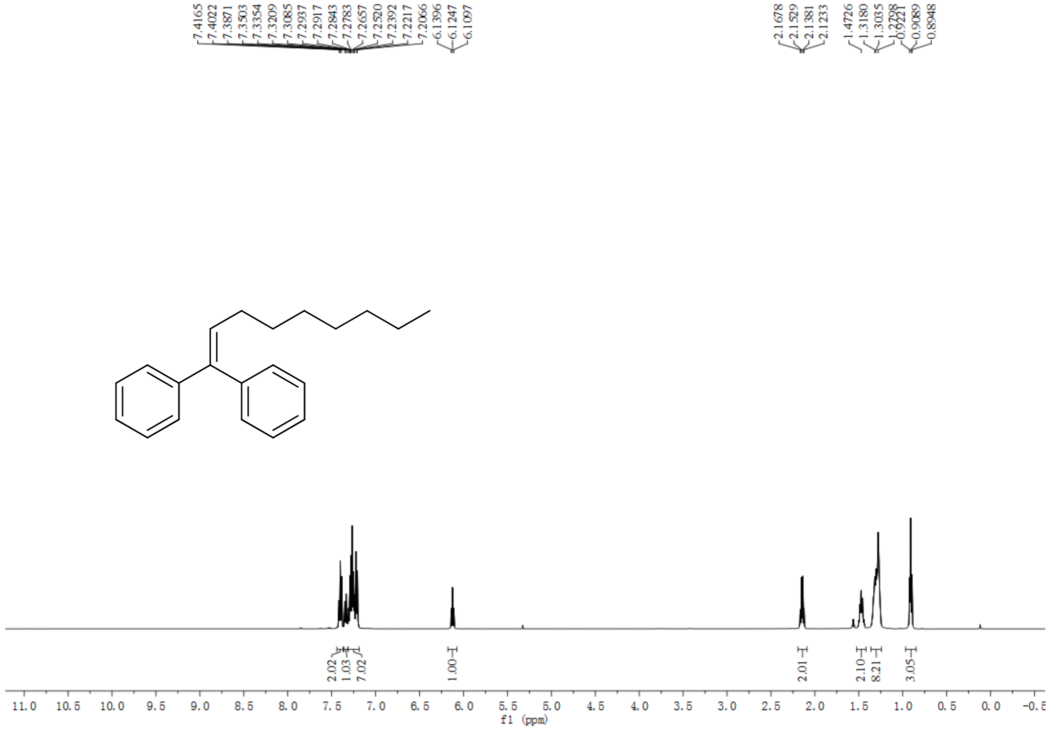
7.4165
7.4022
7.3871
7.3503
7.3354
7.3209
7.3085
7.2937
7.2917
7.2843
7.2783
7.2657
7.2520
7.2392
7.2217
7.2066
6.1396
6.1247
6.1097
2.1678
2.1529
2.1381
2.1233
1.4726
1.3180
1.3035
1.2798
0.9221
0.9089
0.8948
2.02
1.03
7.02
1.00
2.01
2.10
8.21
3.05
11.0 10.5 10.0 9.5 9.0 8.5 8.0 7.5 7.0 6.5 6.0 5.5 5.0 4.5 4.0 3.5 3.0 2.5 2.0 1.5 1.0 0.5 0.0 -0.5
f1 (ppm)

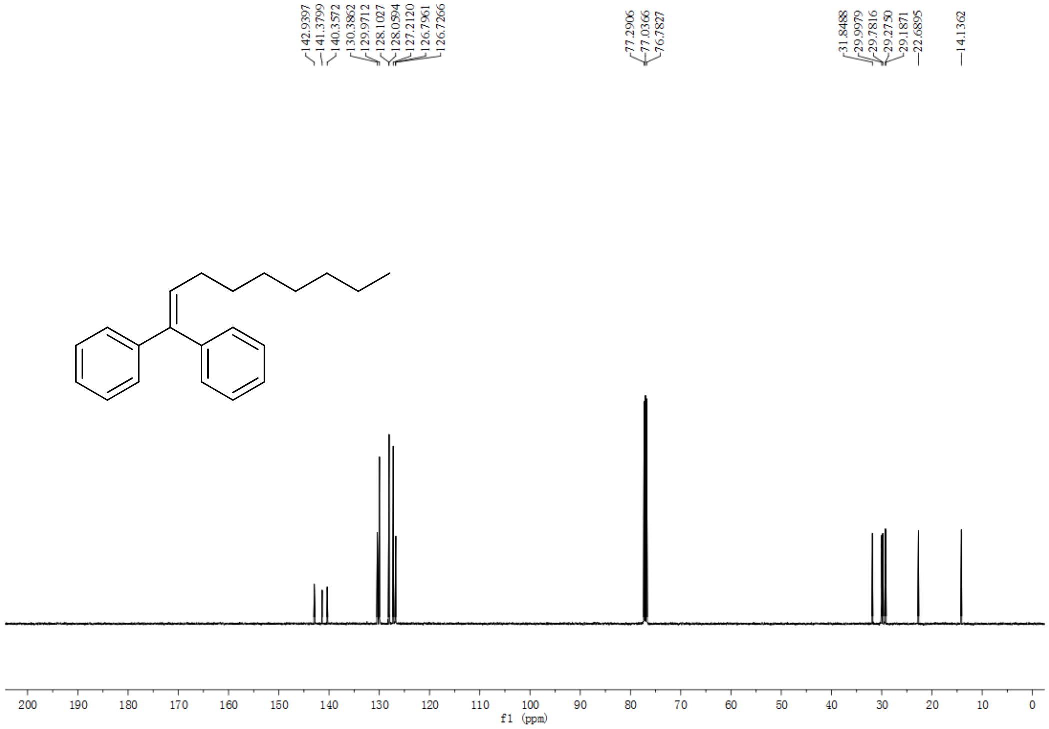
142.9397
141.3799
140.3572
130.3862
129.9712
128.1027
128.0594
127.2120
126.7961
126.7266
77.2906
77.0366
76.7827
31.8488
29.9979
29.7816
29.2750
29.1871
22.6895
14.1362
200 190 180 170 160 150 140 130 120 110 100 90 80 70 60 50 40 30 20 10 0
f1 (ppm)

4e

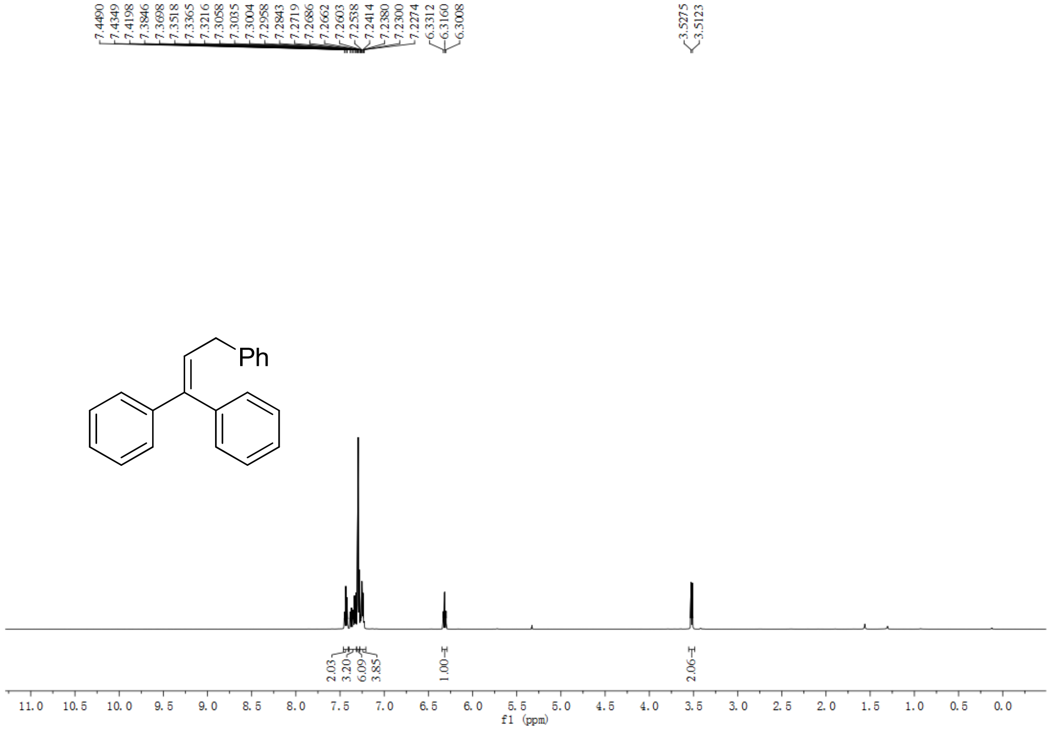
7.4490
7.4349
7.4198
7.3846
7.3698
7.3518
7.3365
7.3216
7.3058
7.3035
7.3004
7.2958
7.2843
7.2719
7.2686
7.2662
7.2603
7.2538
7.2414
7.2380
7.2300
7.2274
6.3312
6.3160
6.3008
3.5275
3.5123
Ph
2.03
3.20
6.09
3.85
1.00
2.06
f1 (ppm)

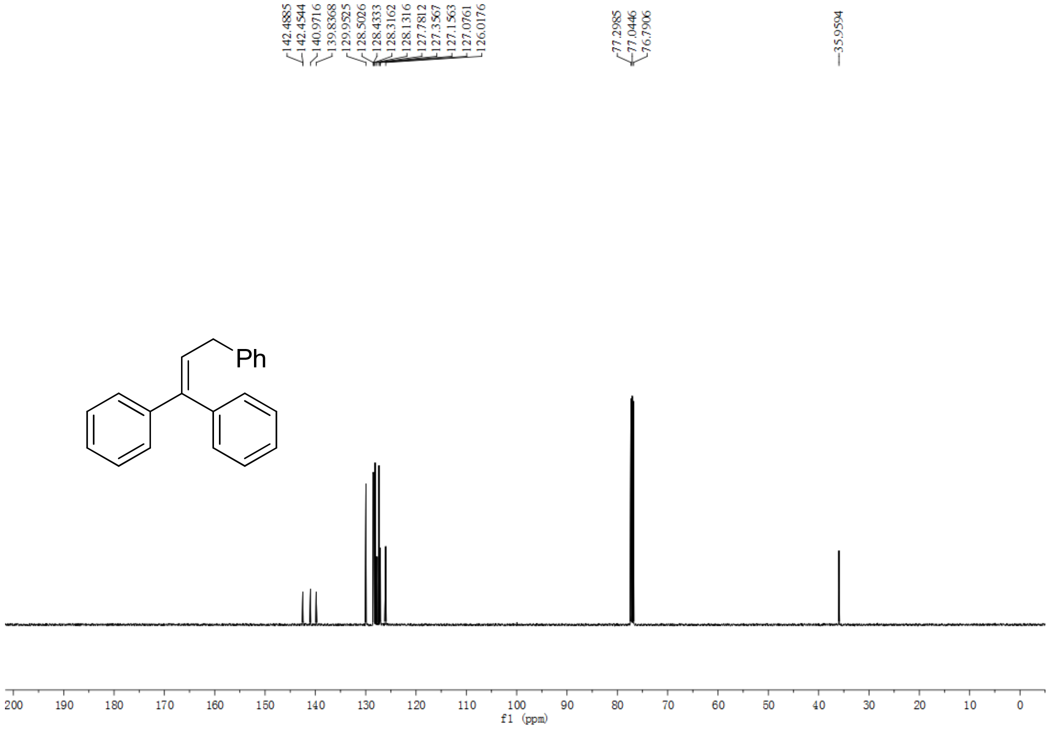
142.4885
142.4544
140.9716
139.8368
129.9525
128.5026
128.4333
128.3162
128.1316
127.7812
127.3567
127.1563
127.0761
126.0176
77.2985
77.0446
76.7906
35.9594
Ph
f1 (ppm)

4f(*E*)

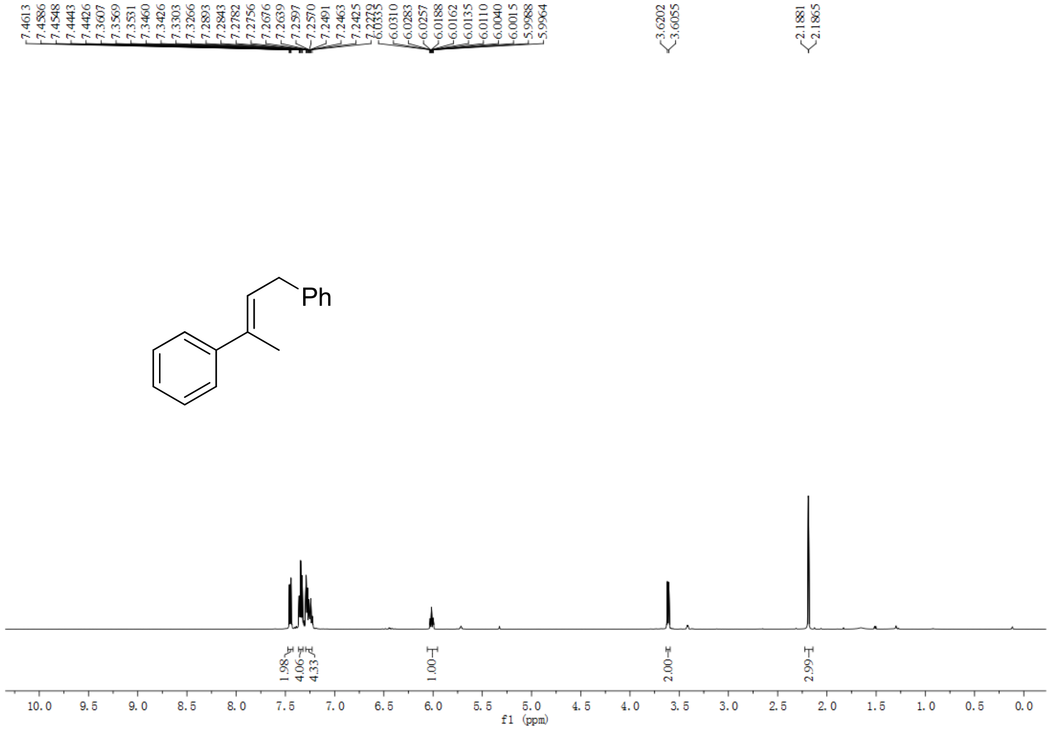
7.4613
7.4586
7.4548
7.4443
7.4426
7.3607
7.3569
7.3531
7.3460
7.3426
7.3303
7.3266
7.2893
7.2843
7.2782
7.2756
7.2676
7.2639
7.2597
7.2570
7.2491
7.2463
7.2425
7.2279
6.0335
6.0310
6.0283
6.0257
6.0188
6.0162
6.0135
6.0110
6.0040
6.0015
5.9988
5.9964
3.6202
3.6055
2.1881
2.1865
Ph
1.98
4.06
4.33
1.00
2.00
2.99
10.0 9.5 9.0 8.5 8.0 7.5 7.0 6.5 6.0 5.5 5.0 4.5 4.0 3.5 3.0 2.5 2.0 1.5 1.0 0.5 0.0
f1 (ppm)

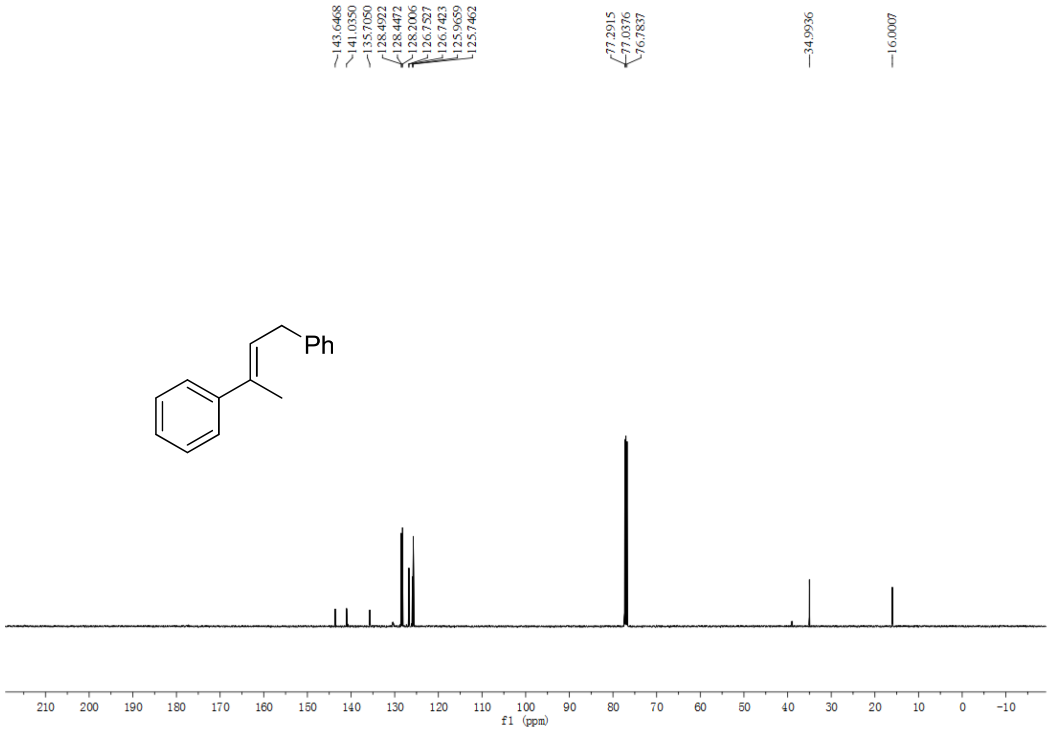
143.6468
141.0350
135.7050
128.4922
128.4472
128.2006
126.7527
126.7423
125.9659
125.7462
77.2915
77.0376
76.7837
34.9936
16.0007
Ph
210 200 190 180 170 160 150 140 130 120 110 100 90 80 70 60 50 40 30 20 10 0 -10
f1 (ppm)

4f(*Z*)

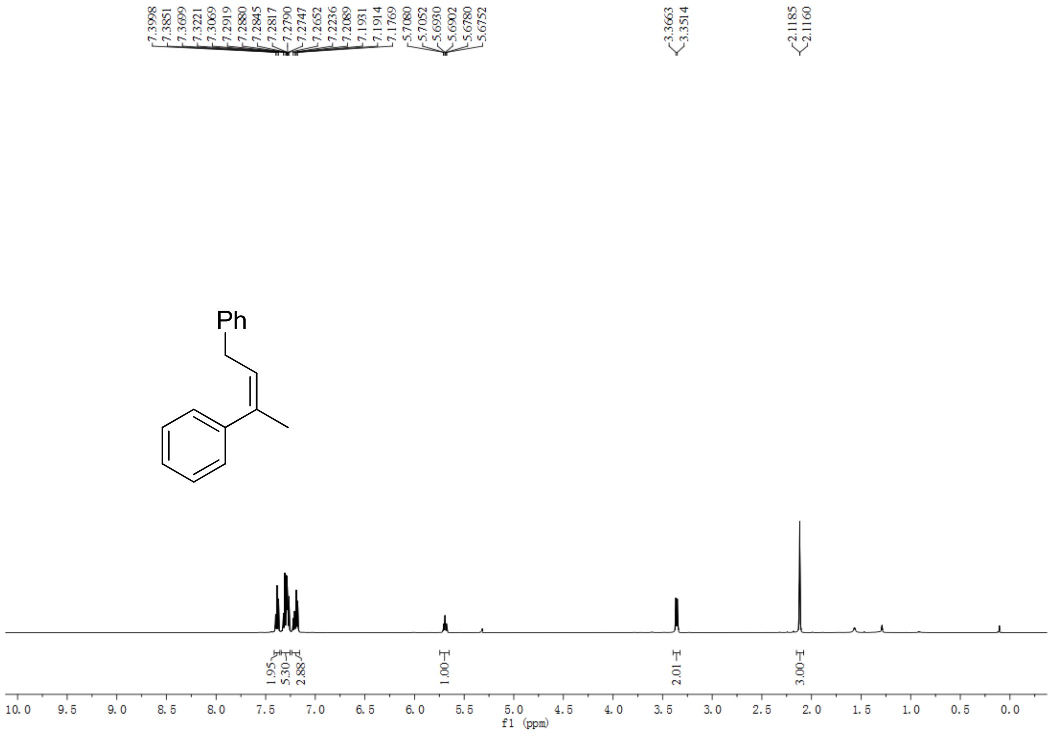

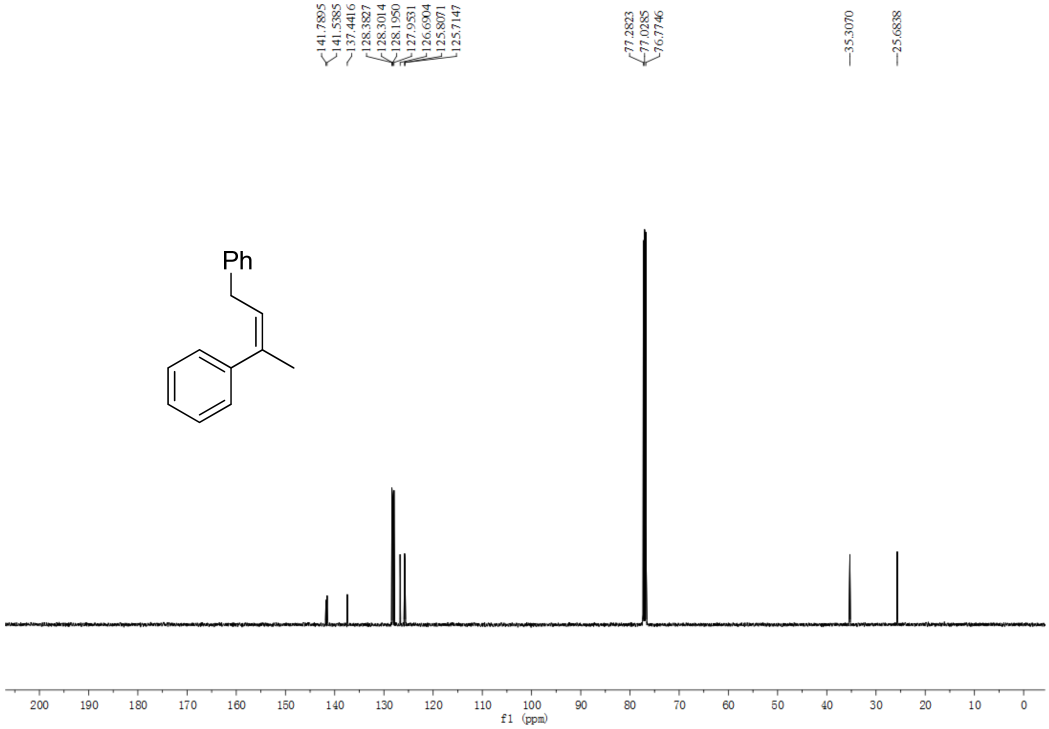

4g

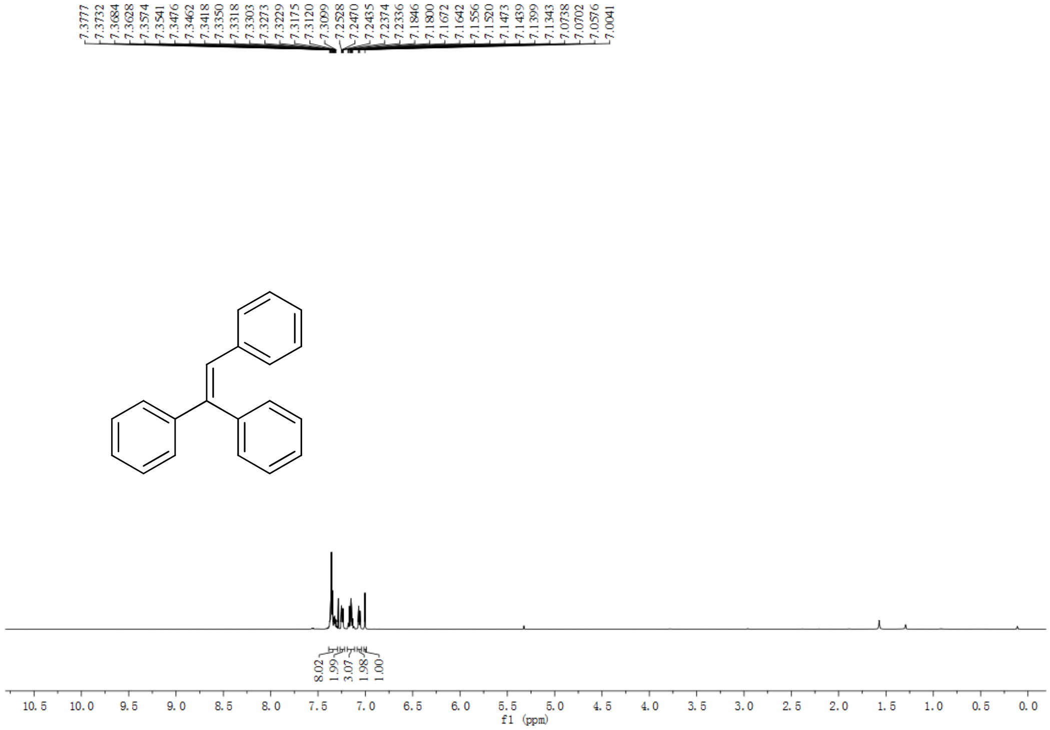
7.3777
7.3732
7.3684
7.3628
7.3574
7.3541
7.3476
7.3462
7.3418
7.3350
7.3318
7.3303
7.3273
7.3229
7.3175
7.3120
7.3099
7.2528
7.2470
7.2435
7.2374
7.2336
7.1846
7.1800
7.1672
7.1642
7.1556
7.1520
7.1473
7.1439
7.1399
7.1343
7.0738
7.0702
7.0576
7.0041
8.02
1.99
3.07
1.98
1.00
f1 (ppm)

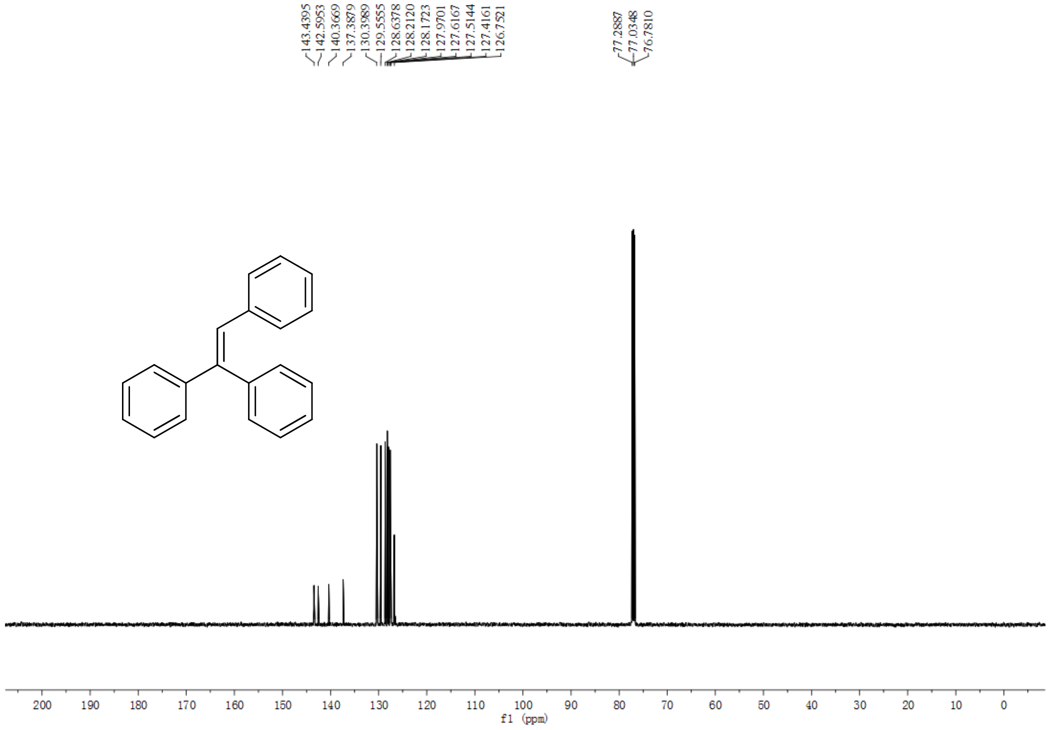
143.4395
142.5953
140.3669
137.3879
130.3989
129.5555
128.6378
128.2120
128.1723
127.9701
127.6167
127.5144
127.4161
126.7521
77.2887
77.0348
76.7810
f1 (ppm)

4h

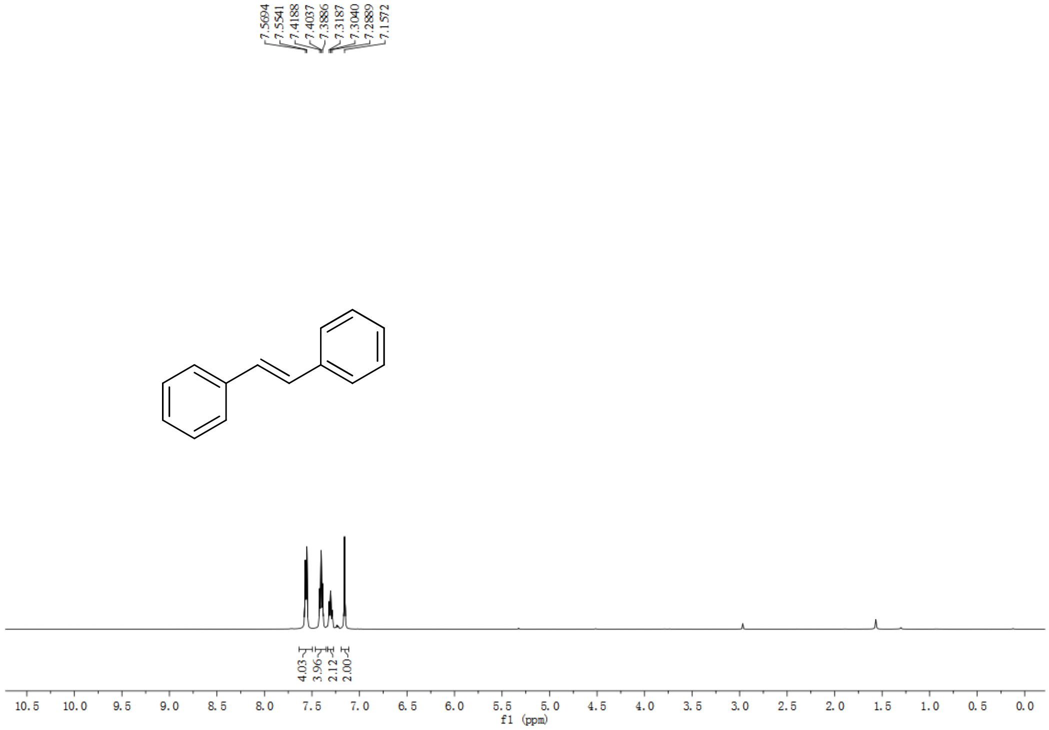

7.5694
7.5541
7.4188
7.4037
7.3886
7.3187
7.3040
7.2889
7.1572
4.03
3.96
2.12
2.00
10.5 10.0 9.5 9.0 8.5 8.0 7.5 7.0 6.5 6.0 5.5 5.0 4.5 4.0 3.5 3.0 2.5 2.0 1.5 1.0 0.5 0.0
f1 (ppm)

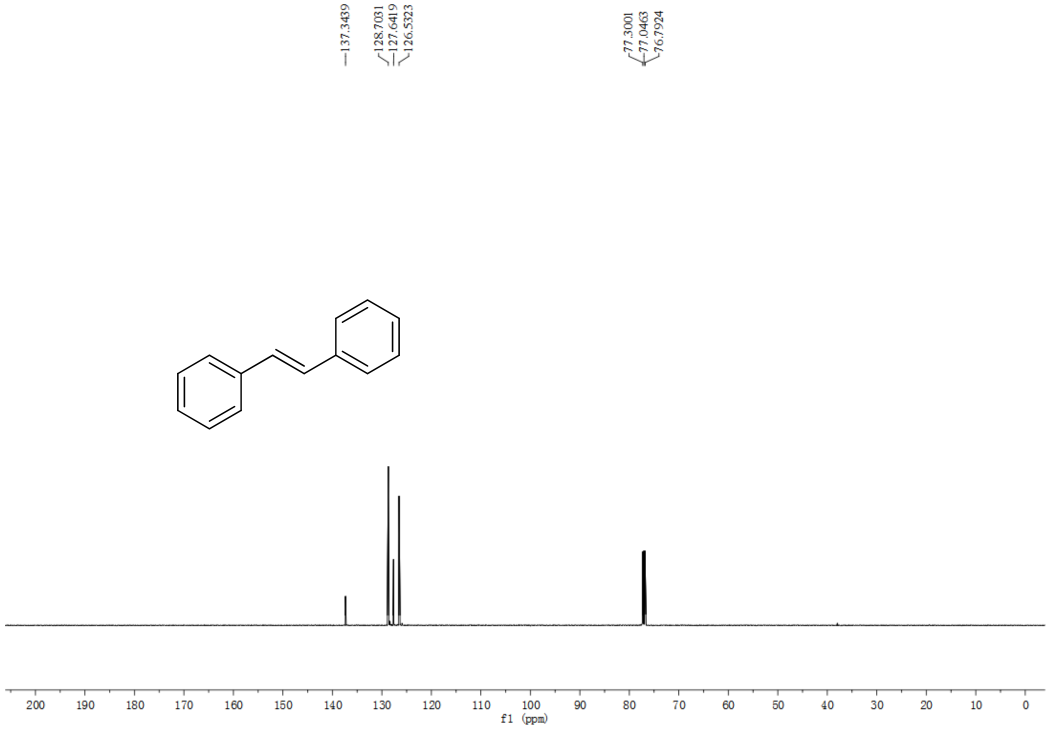

137.3439
128.7031
127.6419
126.5323
77.3001
77.0463
76.7924
200 190 180 170 160 150 140 130 120 110 100 90 80 70 60 50 40 30 20 10 0
f1 (ppm)

4i

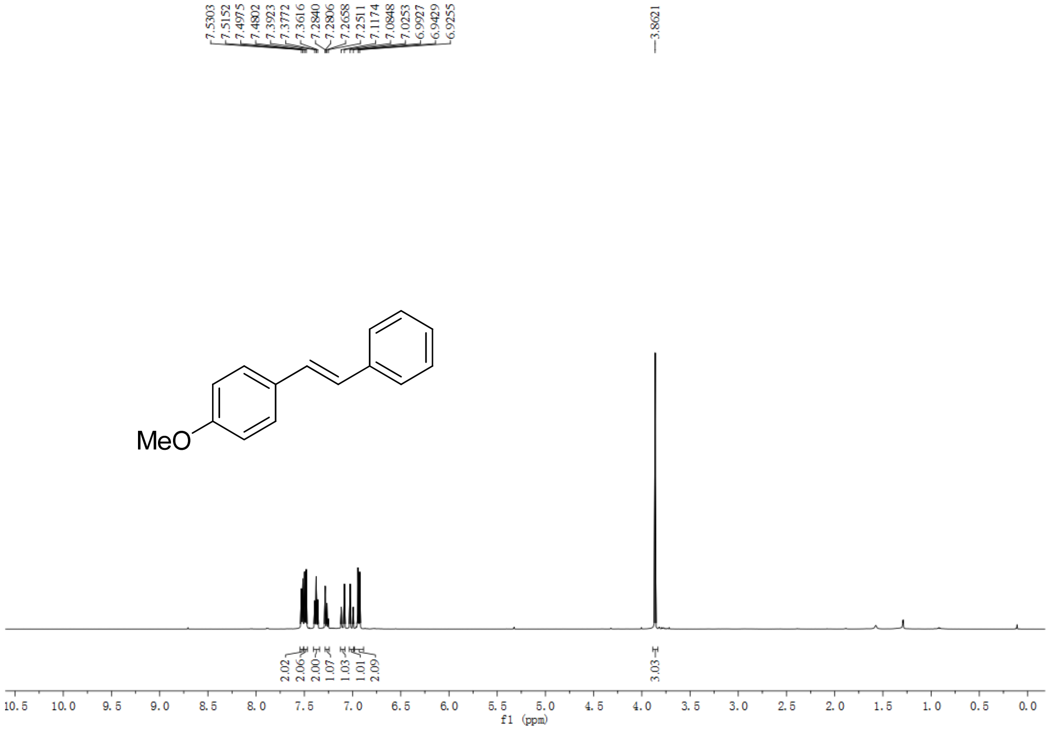
7.5303
7.5152
7.4975
7.4802
7.3923
7.3772
7.3616
7.2840
7.2806
7.2658
7.2511
7.1174
7.0848
7.0253
6.9927
6.9429
6.9255
3.8621
MeO
2.02
2.06
2.00
1.07
1.03
1.01
2.09
3.03
f1 (ppm)

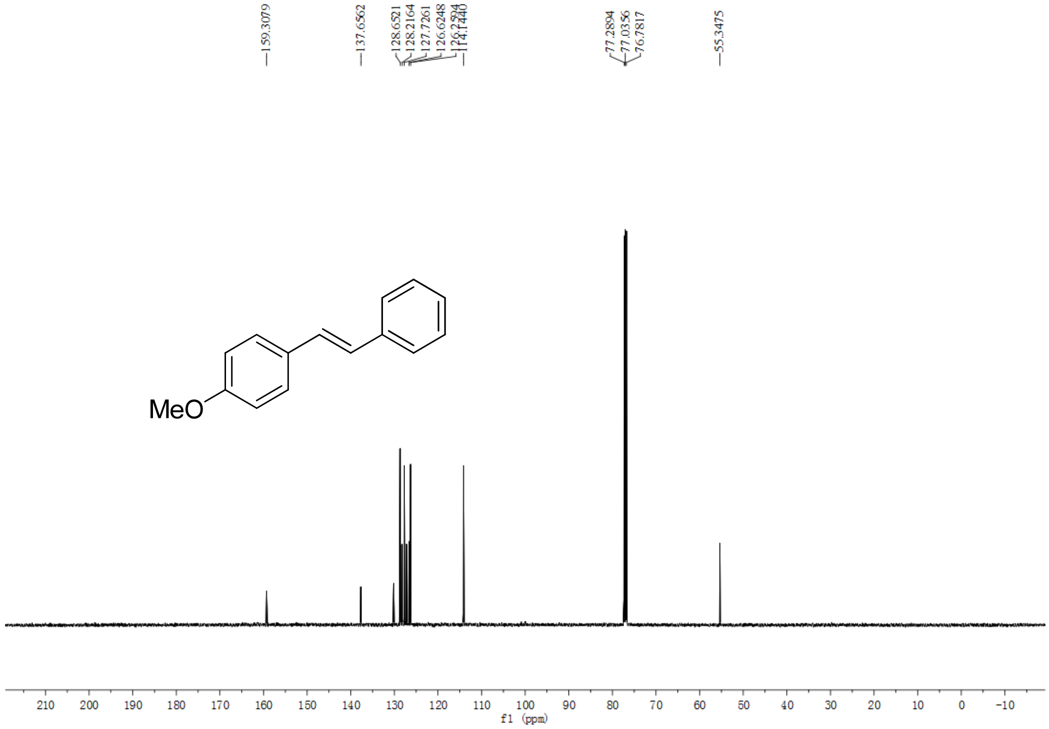
159.3079
137.6562
128.6521
128.2164
127.7261
126.6248
114.1440
77.2894
77.0356
76.7817
55.3475
MeO
f1 (ppm)

4j

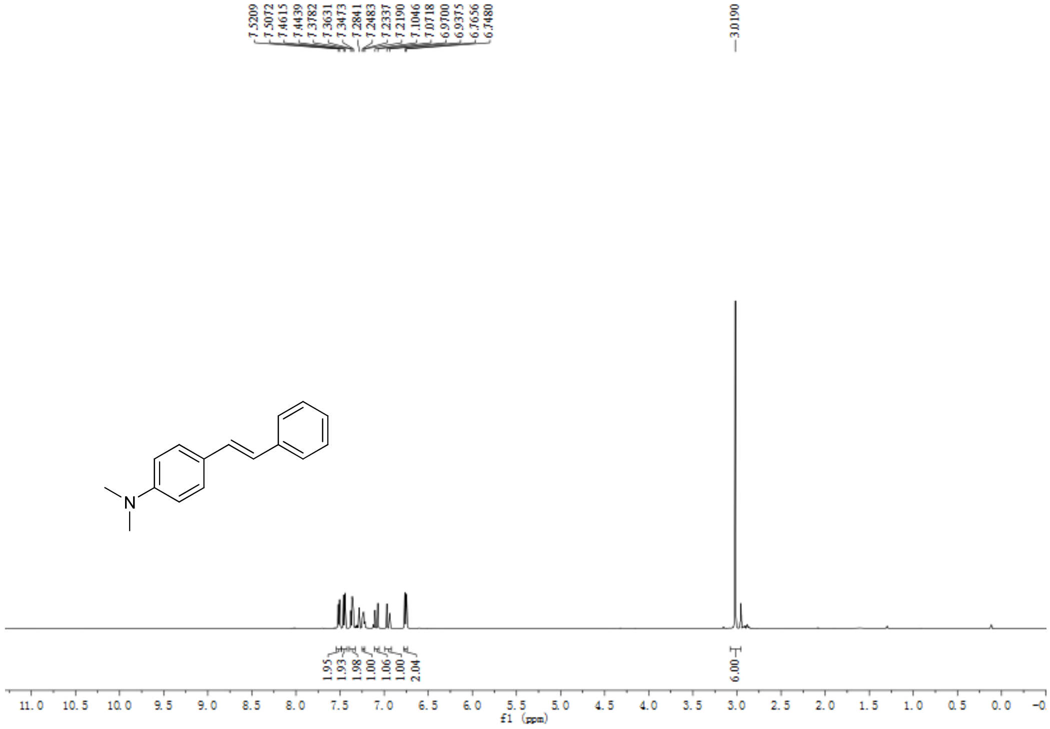
7.5209
7.5072
7.4615
7.4439
7.3782
7.3631
7.3473
7.2841
7.2483
7.2337
7.2190
7.1046
7.0718
6.9700
6.9375
6.7656
6.7480
3.0190
1.95
1.93
1.98
1.00
1.06
1.00
2.04
6.00
f1 (ppm)

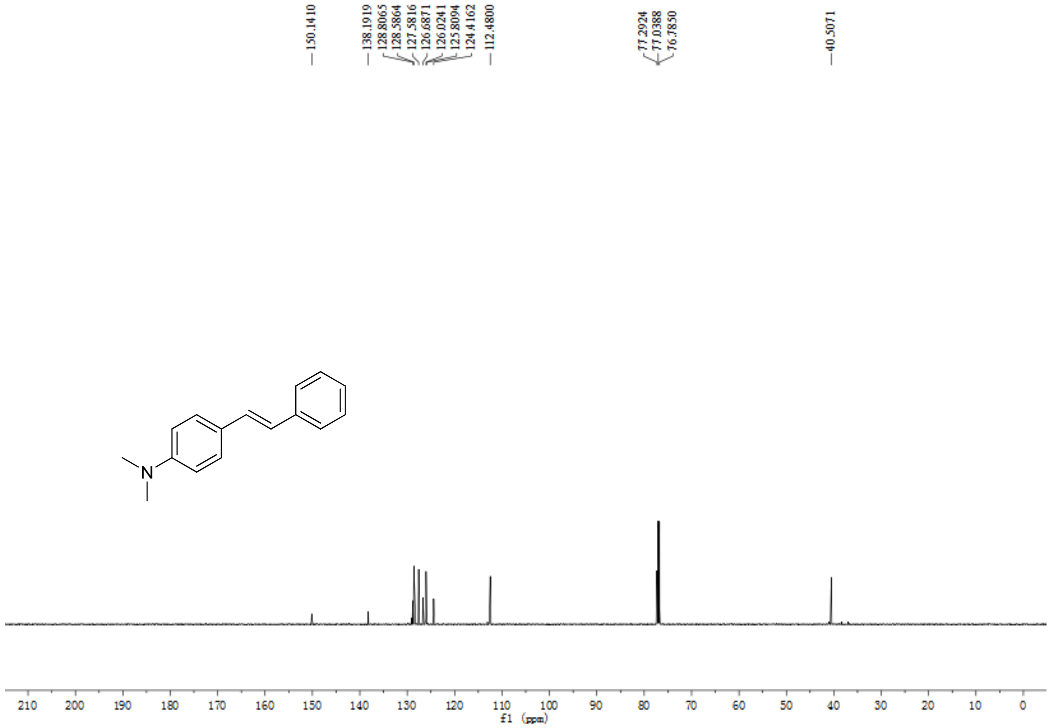
150.1410
138.1919
128.8065
128.5864
127.5816
126.6871
126.0241
125.8094
124.4162
112.4800
77.2924
77.0388
76.7850
40.5071
f1 (ppm)

4k

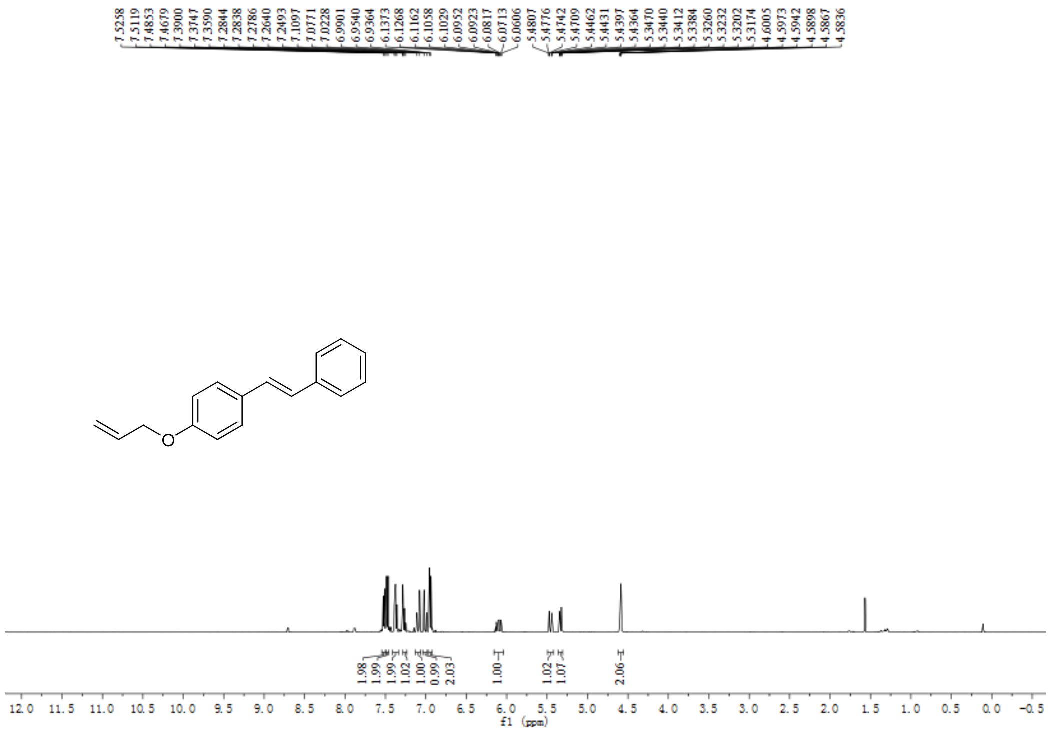
7.5258
7.5119
7.4853
7.4679
7.3900
7.3747
7.3590
7.2844
7.2838
7.2786
7.2640
7.2493
7.1097
7.0771
7.0228
6.9901
6.9540
6.9364
6.1373
6.1268
6.1162
6.1058
6.1029
6.0952
6.0923
6.0817
6.0713
6.0606
5.4807
5.4776
5.4742
5.4709
5.4462
5.4431
5.4397
5.4364
5.3470
5.3440
5.3412
5.3384
5.3260
5.3232
5.3202
5.3174
4.6005
4.5973
4.5942
4.5898
4.5867
4.5836
O
1.98
1.99
1.99
1.02
1.00
0.99
2.03
1.00
1.02
1.07
2.06
12.0 11.5 11.0 10.5 10.0 9.5 9.0 8.5 8.0 7.5 7.0 6.5 6.0 5.5 5.0 4.5 4.0 3.5 3.0 2.5 2.0 1.5 1.0 0.5 0.0 -0.5
f1 (ppm)

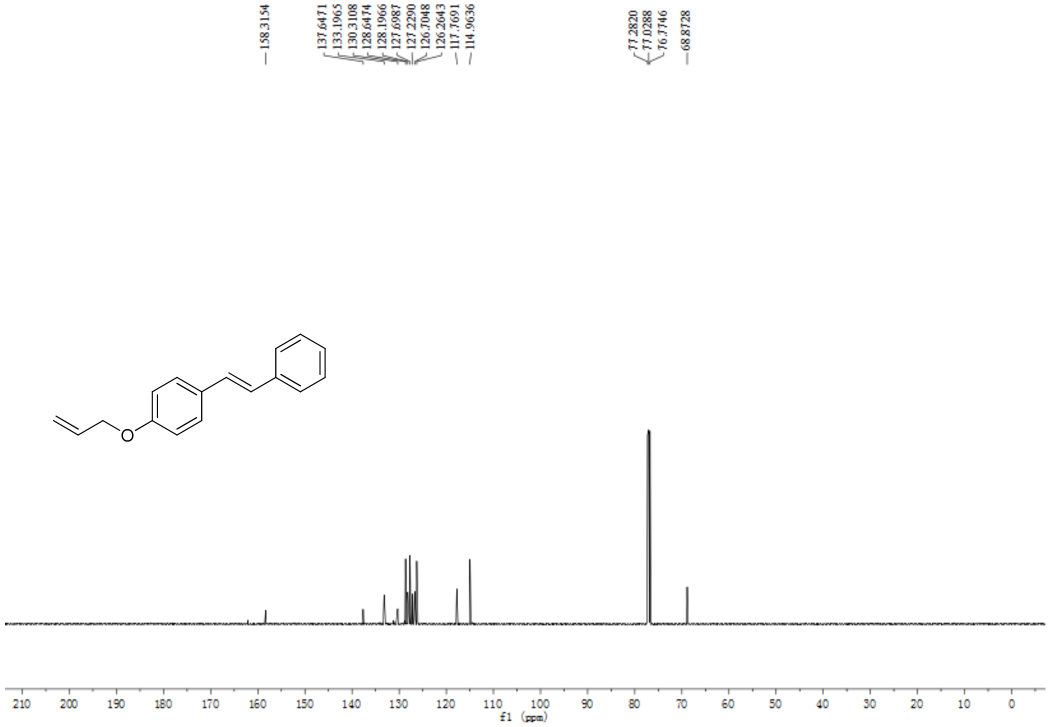
158.3154
137.6471
133.1965
130.3108
128.6474
128.1966
127.6987
127.2290
126.7048
126.2643
117.7691
114.9636
77.2820
77.0288
76.7746
68.8728
O
210 200 190 180 170 160 150 140 130 120 110 100 90 80 70 60 50 40 30 20 10 0
f1 (ppm)

4l

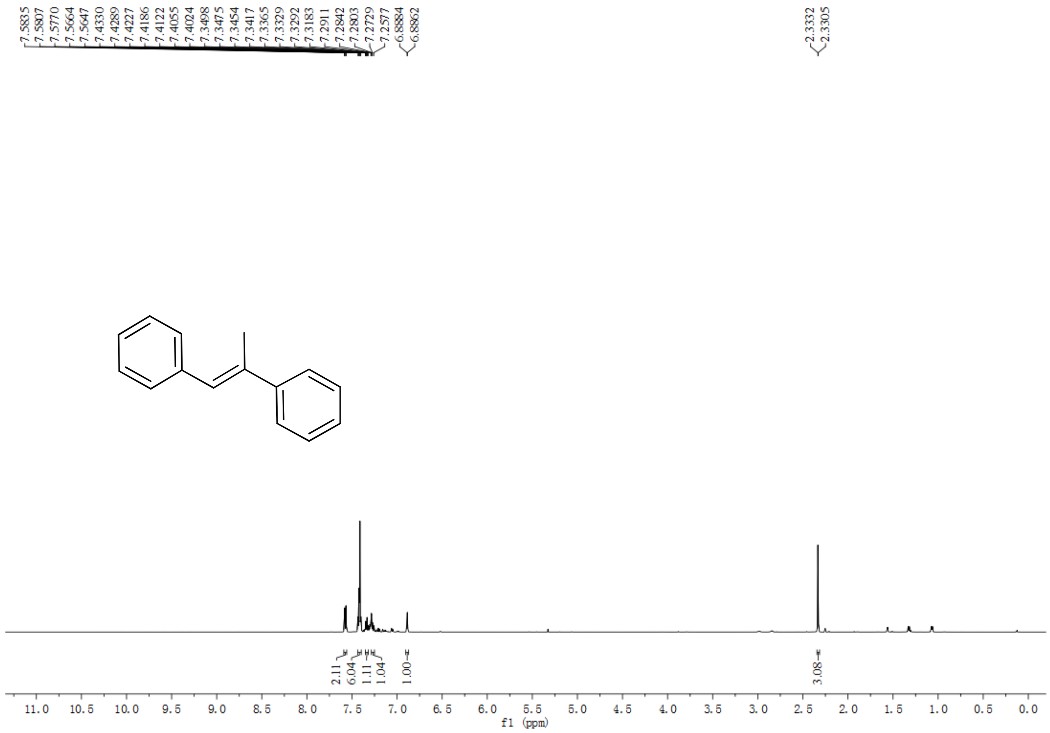
7.5835
7.5807
7.5770
7.5664
7.5647
7.4330
7.4289
7.4227
7.4186
7.4122
7.4055
7.4024
7.3498
7.3475
7.3454
7.3417
7.3365
7.3329
7.3292
7.3183
7.2911
7.2842
7.2803
7.2729
7.2577
6.8884
6.8862
2.3332
2.3305
2.11
6.04
1.11
1.04
1.00
3.08
11.0 10.5 10.0 9.5 9.0 8.5 8.0 7.5 7.0 6.5 6.0 5.5 5.0 4.5 4.0 3.5 3.0 2.5 2.0 1.5 1.0 0.5 0.0
f1 (ppm)

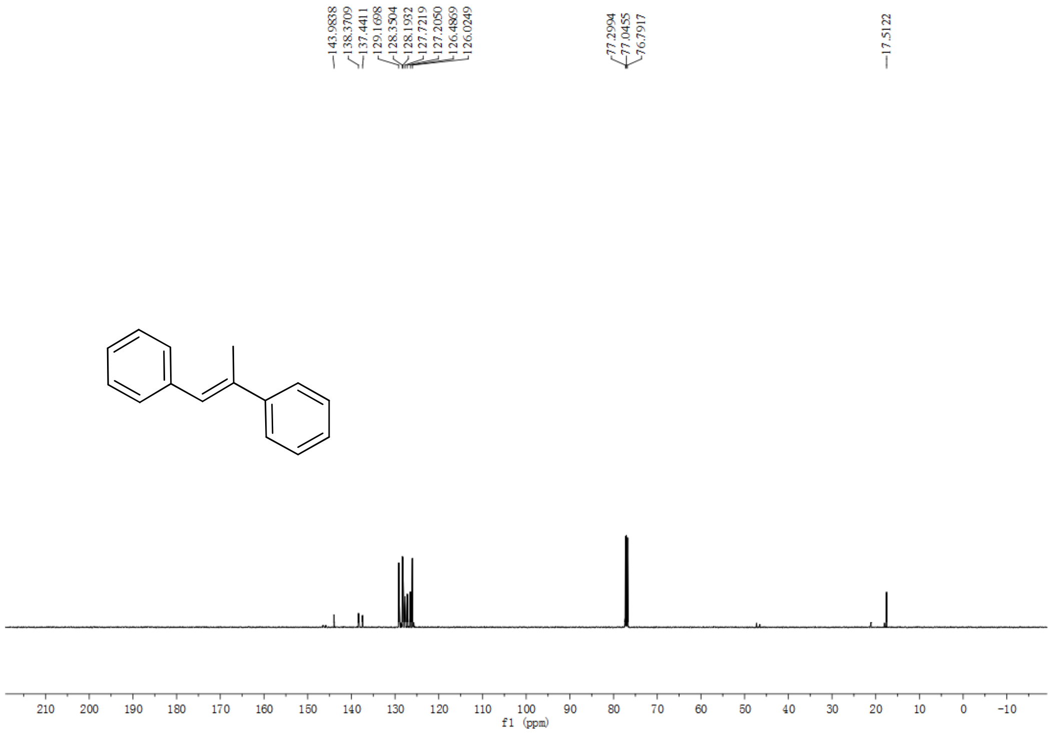
143.9838
138.3709
137.4411
129.1698
128.3504
128.1932
127.7219
127.2050
126.4869
126.0249
77.2994
77.0455
76.7917
17.5122
210 200 190 180 170 160 150 140 130 120 110 100 90 80 70 60 50 40 30 20 10 0 -10
f1 (ppm)

4m

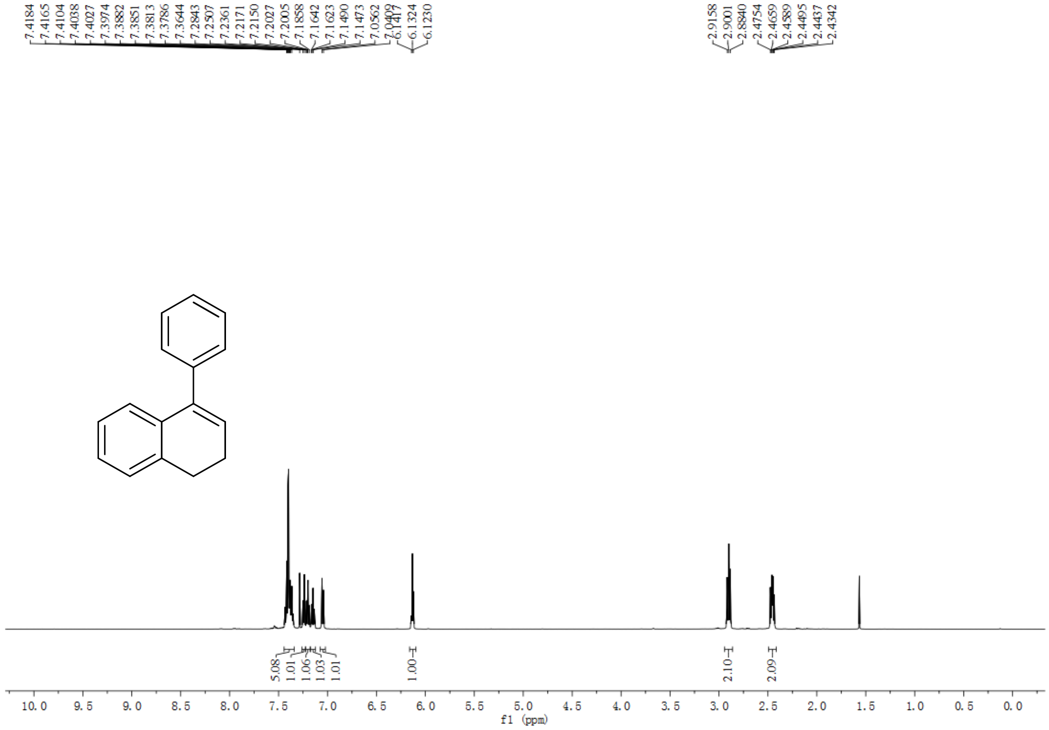

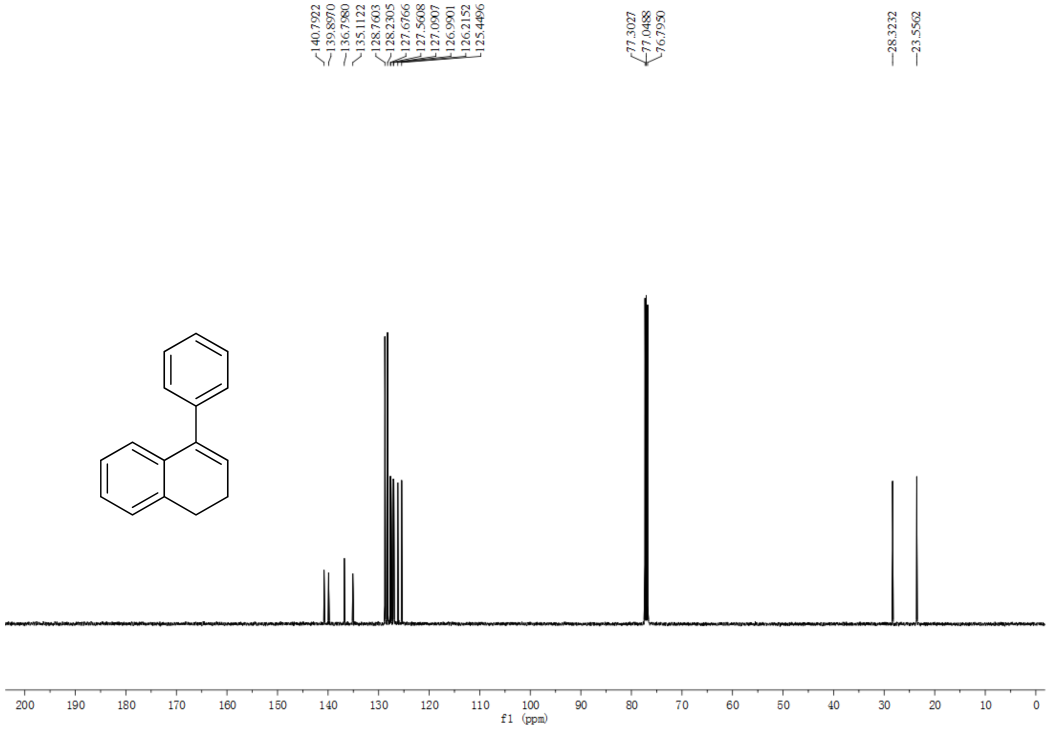

4n

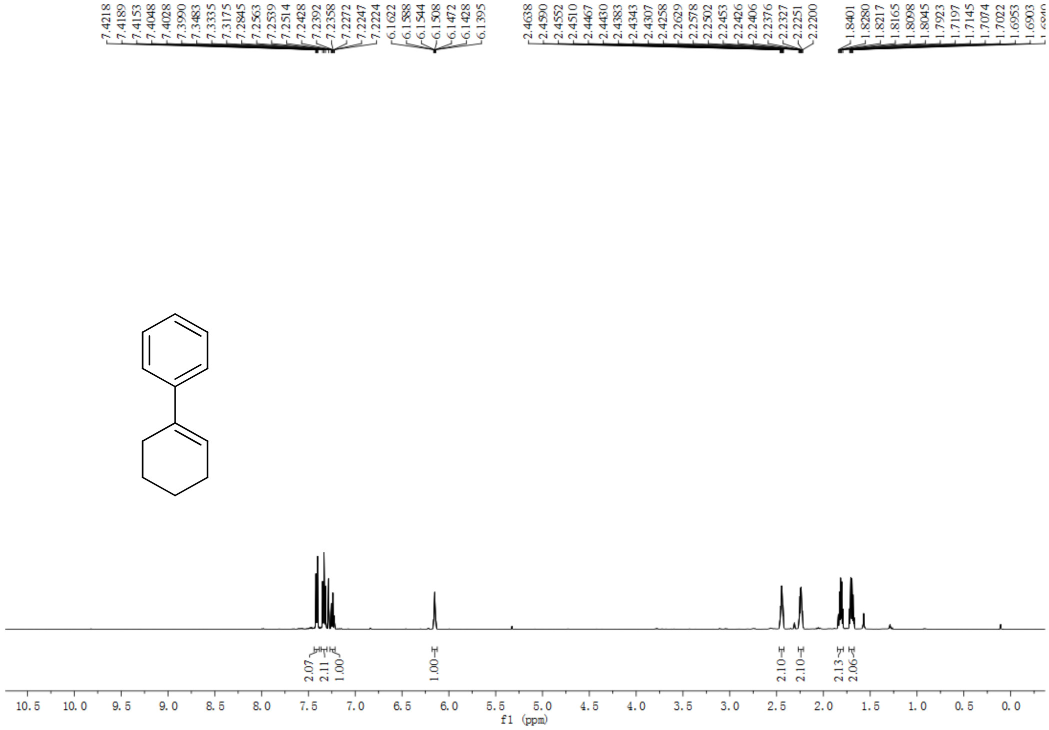

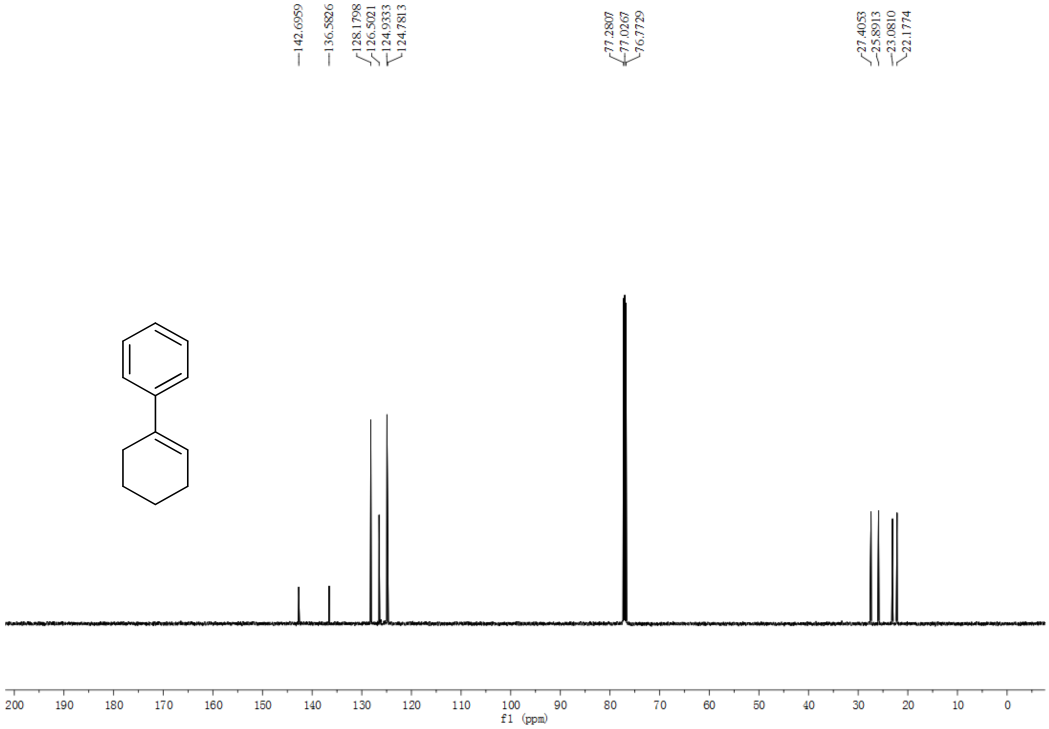

4o

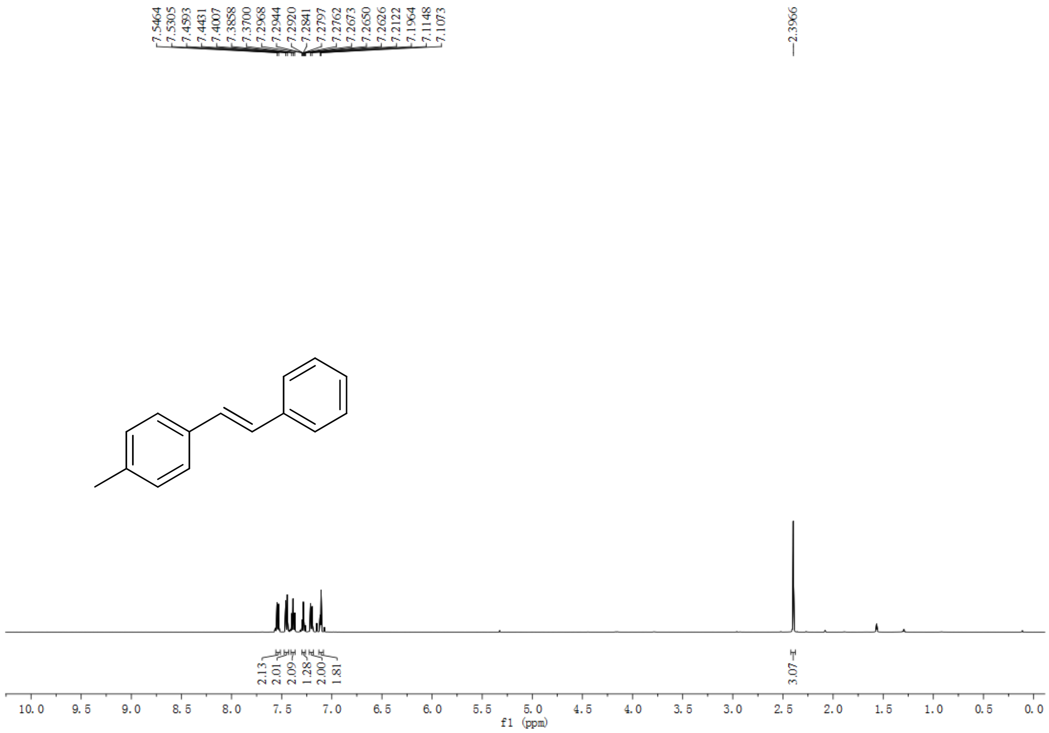

7.5464
7.5305
7.4593
7.4431
7.4007
7.3858
7.3700
7.2968
7.2944
7.2920
7.2841
7.2797
7.2762
7.2673
7.2650
7.2626
7.2122
7.1964
7.1148
7.1073
2.3966
2.13
2.01
2.09
1.28
2.00
1.81
3.07
10.0
9.5
9.0
8.5
8.0
7.5
7.0
6.5
6.0
5.5
5.0
4.5
4.0
3.5
3.0
2.5
2.0
1.5
1.0
0.5
0.0
f1 (ppm)

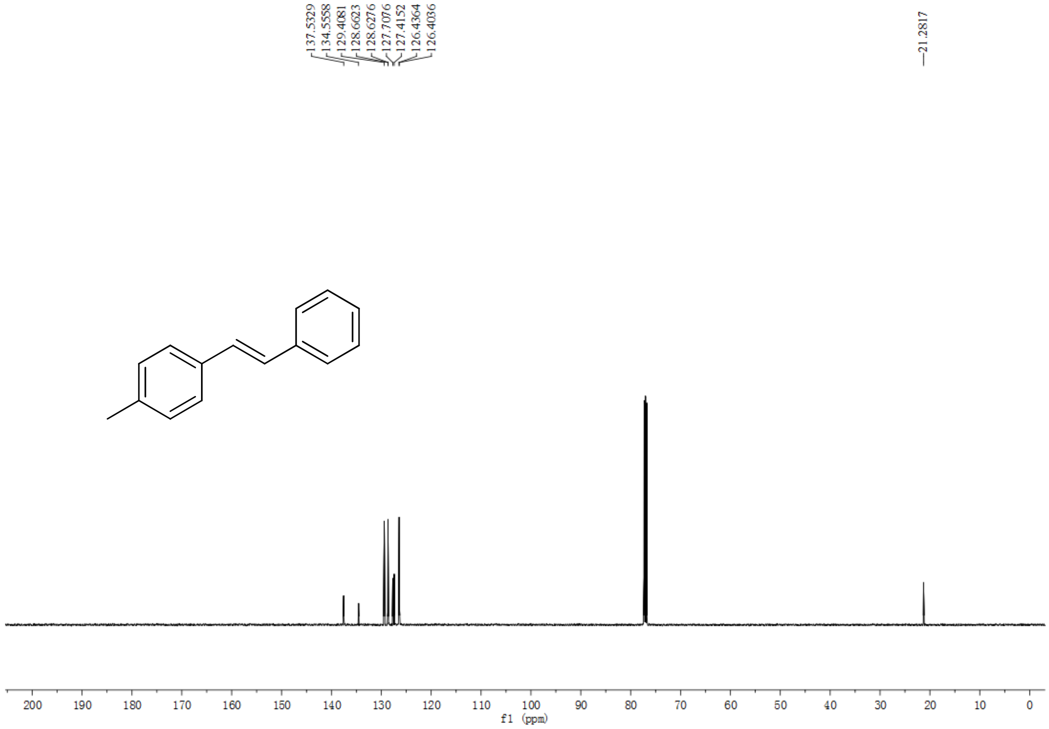

137.5329
134.5558
129.4081
128.6623
128.6276
127.7076
127.4152
126.4364
126.4036
21.2817
200
190
180
170
160
150
140
130
120
110
100
90
80
70
60
50
40
30
20
10
0
f1 (ppm)

4p

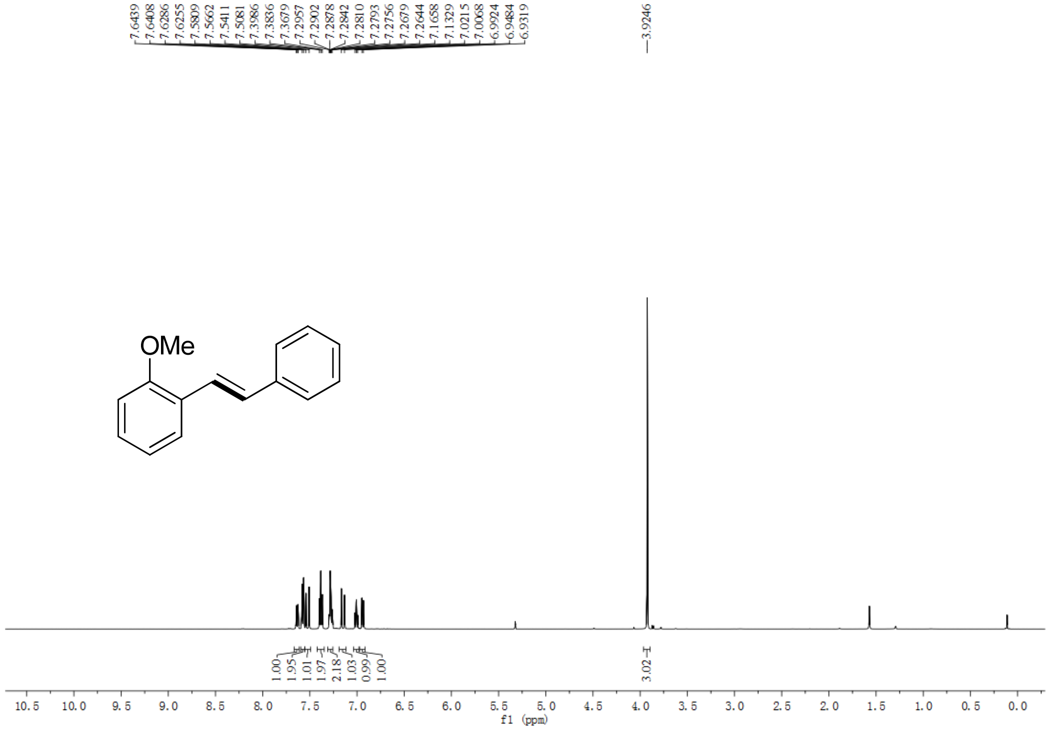
OMe
7.6439
7.6408
7.6286
7.6255
7.5809
7.5662
7.5411
7.5081
7.3986
7.3836
7.3679
7.2957
7.2902
7.2878
7.2842
7.2810
7.2793
7.2756
7.2679
7.2644
7.1658
7.1329
7.0215
7.0068
6.9924
6.9484
6.9319
3.9246
1.00
1.95
1.01
1.97
2.18
1.03
0.99
1.00
3.02
f1 (ppm)

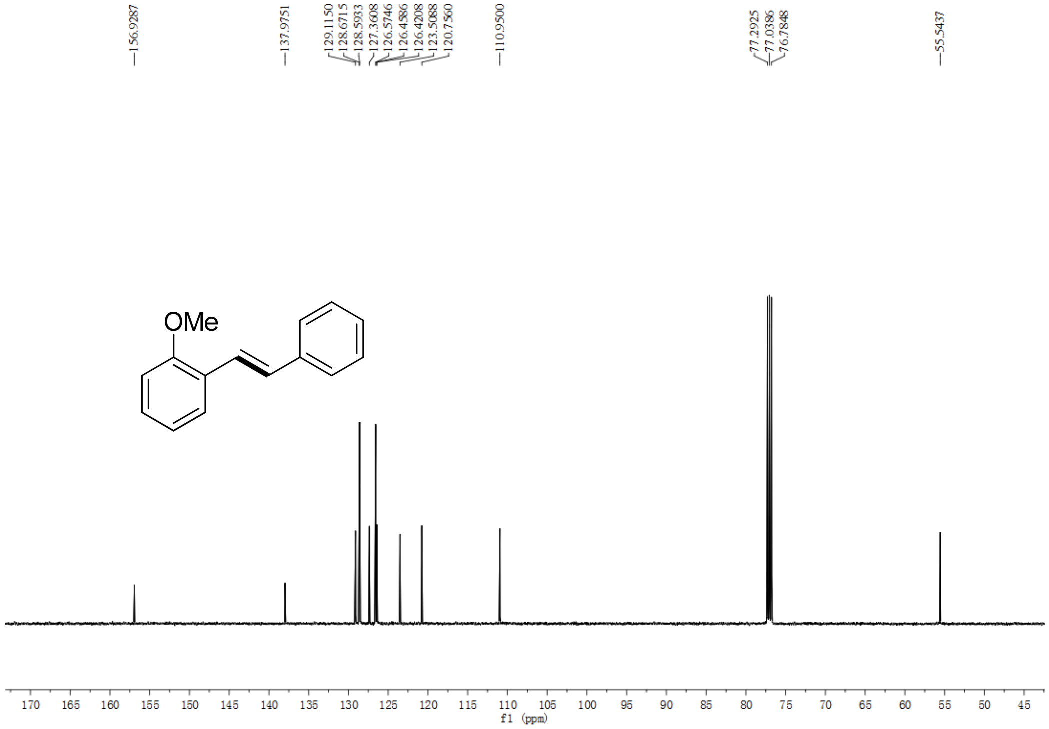
156.9287
137.9751
129.1150
128.6715
128.5933
127.3608
126.5746
126.4586
126.4208
123.5088
120.7560
110.9500
77.2925
77.0386
76.7848
55.5437
OMe
f1 (ppm)

4q

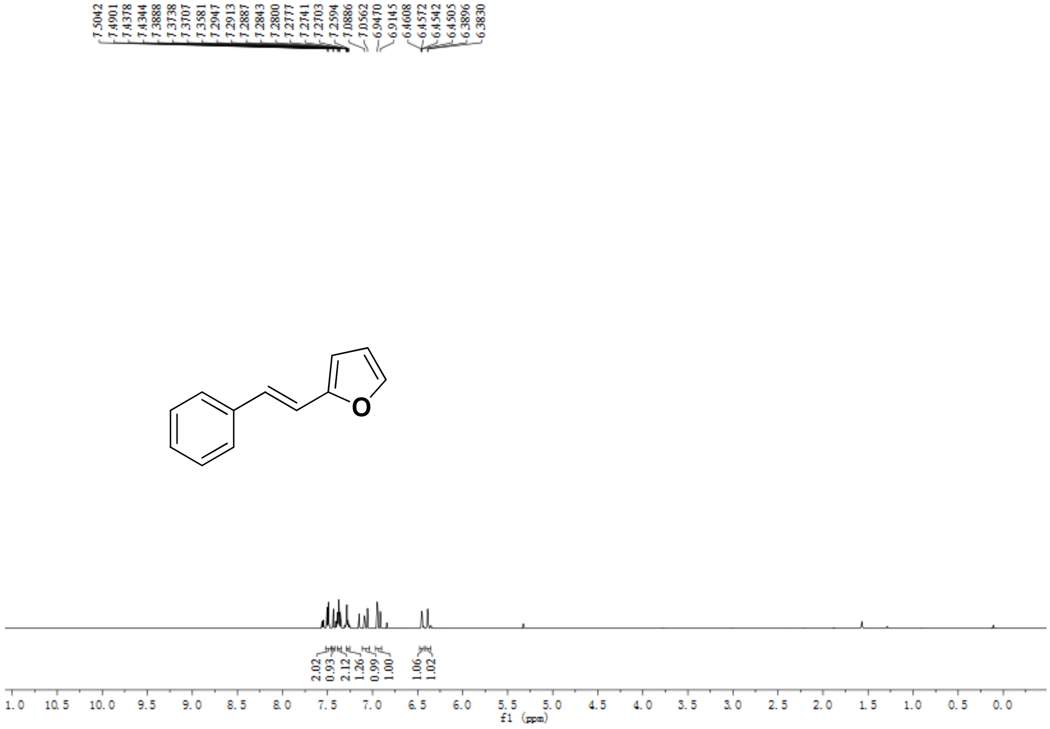
7.5042
7.4901
7.4378
7.4344
7.3888
7.3738
7.3707
7.3581
7.2947
7.2913
7.2887
7.2843
7.2800
7.2777
7.2741
7.2703
7.2594
7.0886
7.0562
6.9470
6.9145
6.4608
6.4572
6.4542
6.4505
6.3896
6.3830
O
2.02
0.93
2.12
1.26
0.99
1.00
1.06
1.02
f1 (ppm)

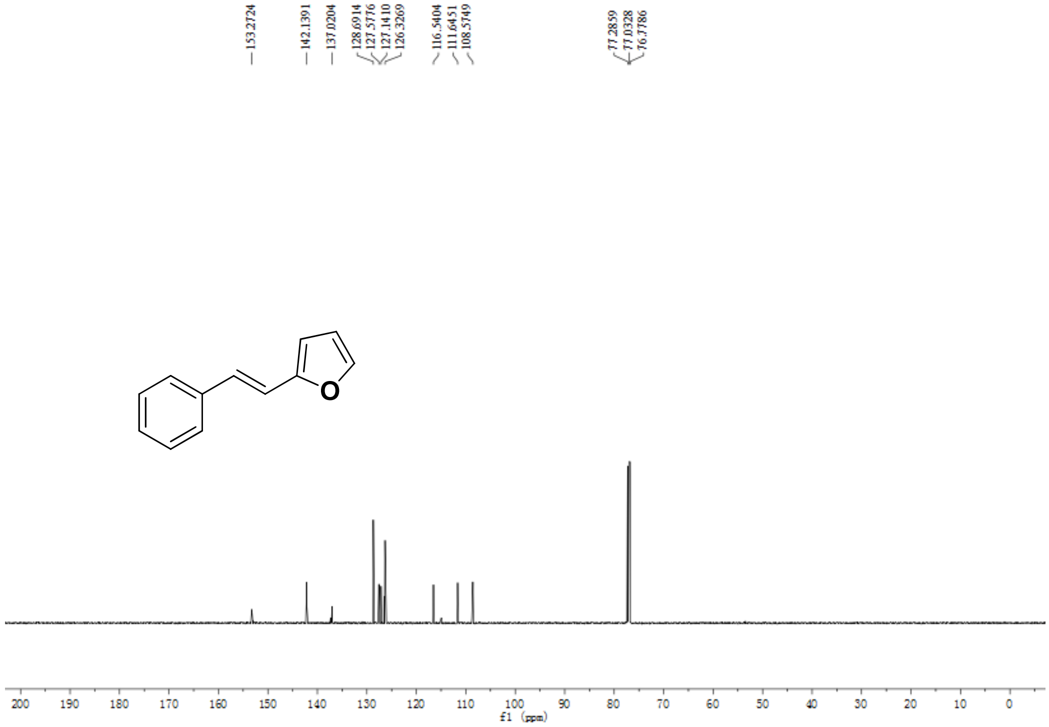
153.2724
142.1391
137.0204
128.6914
127.5776
127.1410
126.3269
116.5404
111.6451
108.5749
77.2859
77.0328
76.7786
O
f1 (ppm)

4r

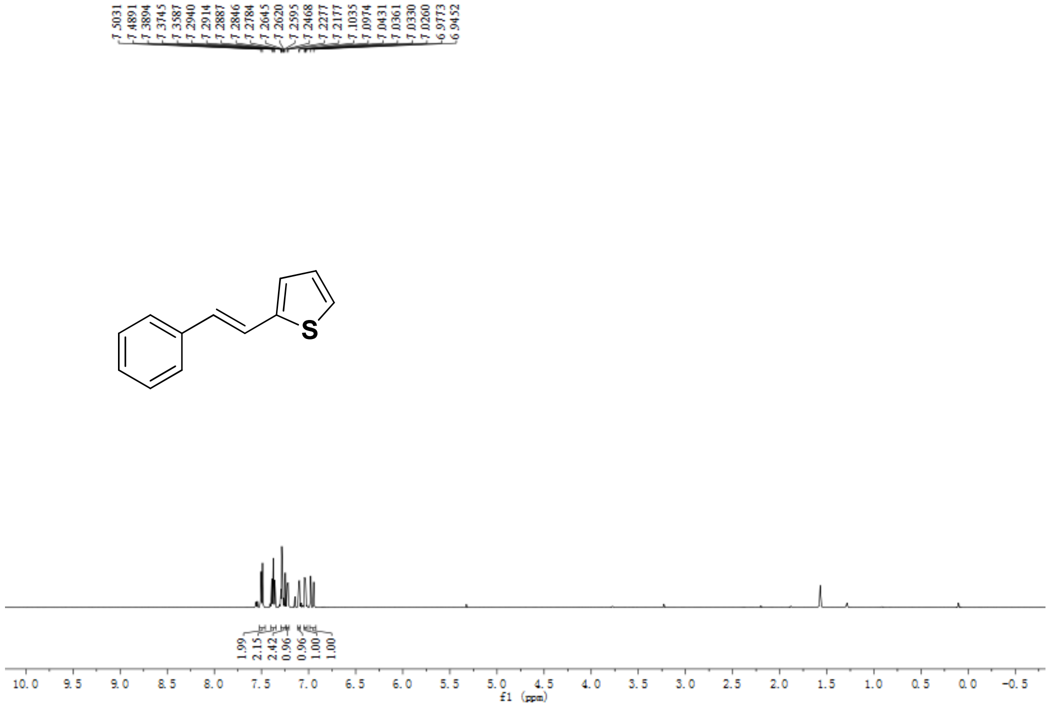
7.5031
7.4891
7.3894
7.3745
7.3587
7.2940
7.2914
7.2887
7.2846
7.2784
7.2645
7.2620
7.2595
7.2468
7.2277
7.2177
7.1035
7.0974
7.0431
7.0361
7.0330
7.0260
6.9773
6.9452
S
1.99
2.15
2.42
0.96
0.96
1.00
1.00
10.0 9.5 9.0 8.5 8.0 7.5 7.0 6.5 6.0 5.5 5.0 4.5 4.0 3.5 3.0 2.5 2.0 1.5 1.0 0.5 0.0 -0.5
f1 (ppm)

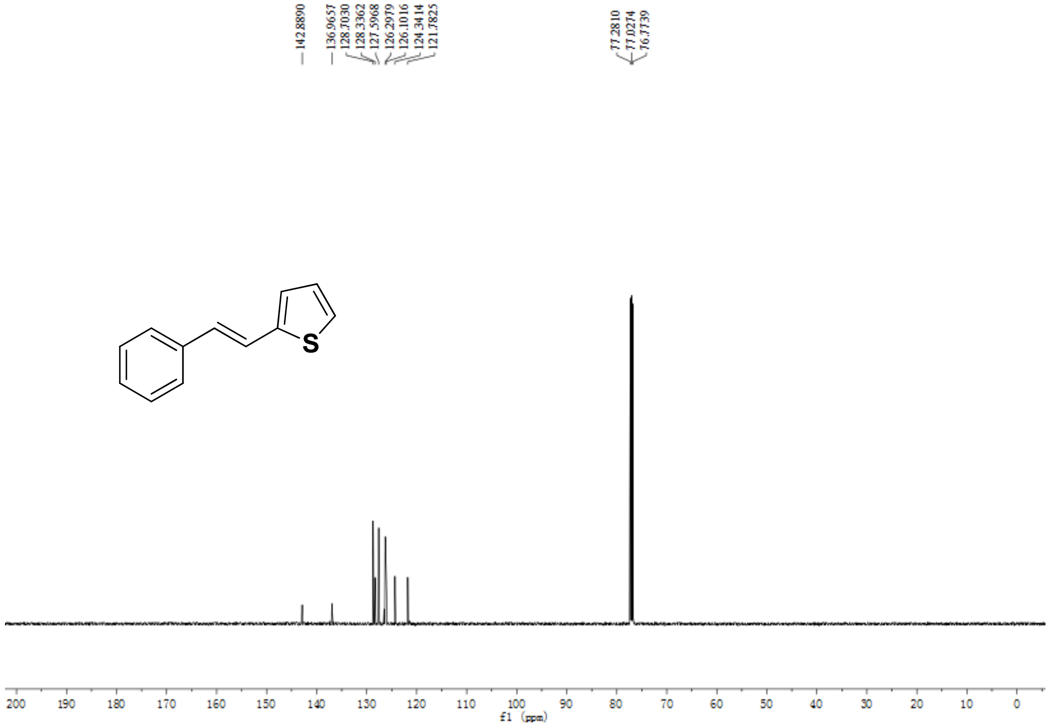
142.8890
136.9657
128.7030
128.3362
127.5968
126.2979
126.1016
124.3414
121.7825
77.2810
77.0274
76.7739
S
200 190 180 170 160 150 140 130 120 110 100 90 80 70 60 50 40 30 20 10 0
f1 (ppm)

4s

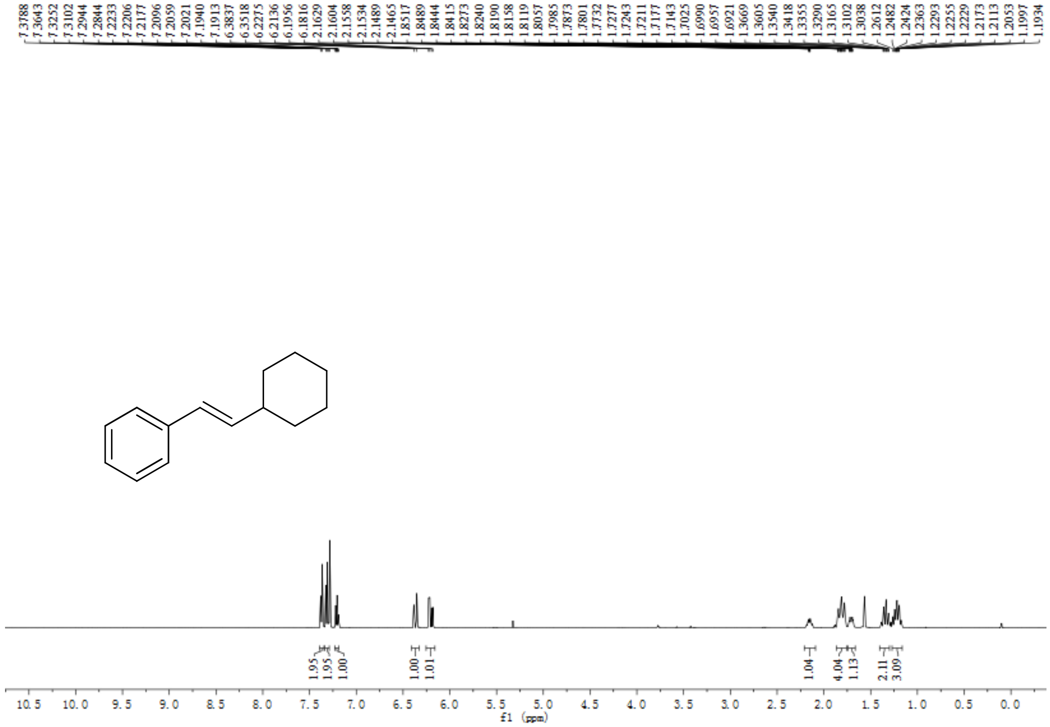
7.3788
7.3643
7.3252
7.3102
7.2944
7.2844
7.2233
7.2206
7.2177
7.2096
7.2059
7.2021
7.1940
7.1913
6.3837
6.3518
6.2275
6.2136
6.1956
6.1816
2.1629
2.1604
2.1558
2.1534
2.1489
2.1465
1.8517
1.8489
1.8444
1.8415
1.8273
1.8240
1.8190
1.8158
1.8119
1.8057
1.7985
1.7873
1.7801
1.7732
1.7277
1.7243
1.7211
1.7177
1.7143
1.7025
1.6990
1.6957
1.6921
1.3669
1.3605
1.3540
1.3418
1.3355
1.3290
1.3165
1.3102
1.3038
1.2612
1.2482
1.2424
1.2363
1.2293
1.2255
1.2229
1.2173
1.2113
1.2053
1.1997
1.1934
1.95
1.95
1.00
1.00
1.01
1.04
4.04
1.13
2.11
3.09
f1 (ppm)

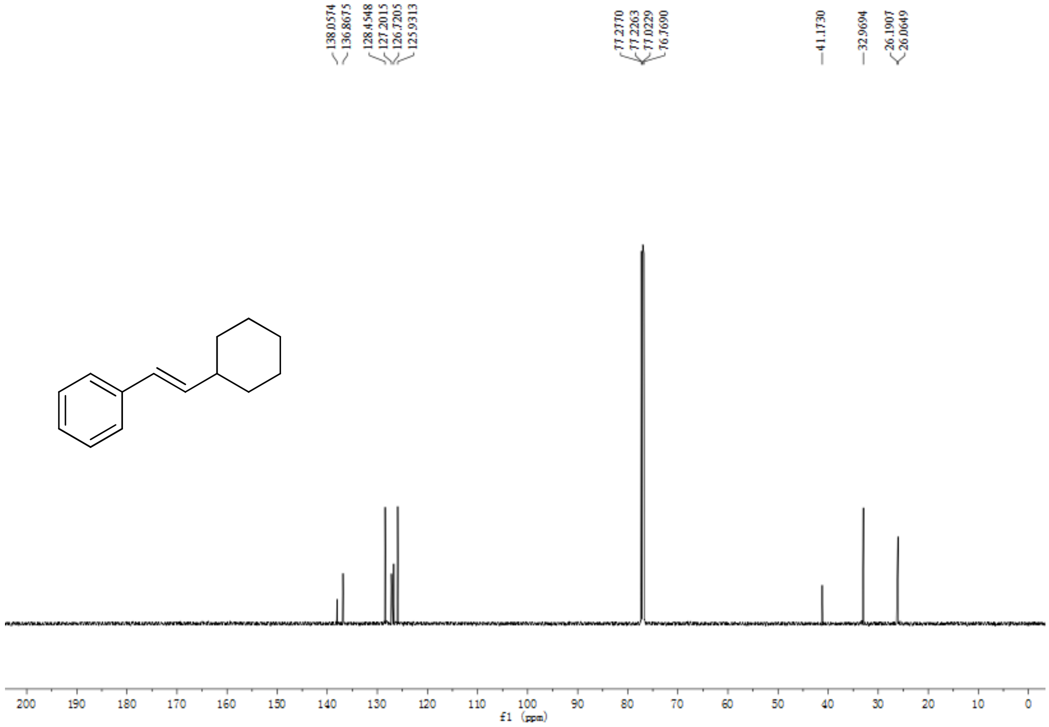
138.0574
136.8675
128.4548
127.2015
126.7205
125.9313
77.2770
77.2263
77.0229
76.7690
41.1730
32.9694
26.1907
26.0649
f1 (ppm)

7. Crude ^{1}H NMR and HRMS copies of cross azines.

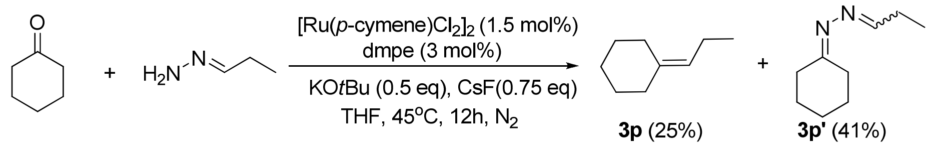

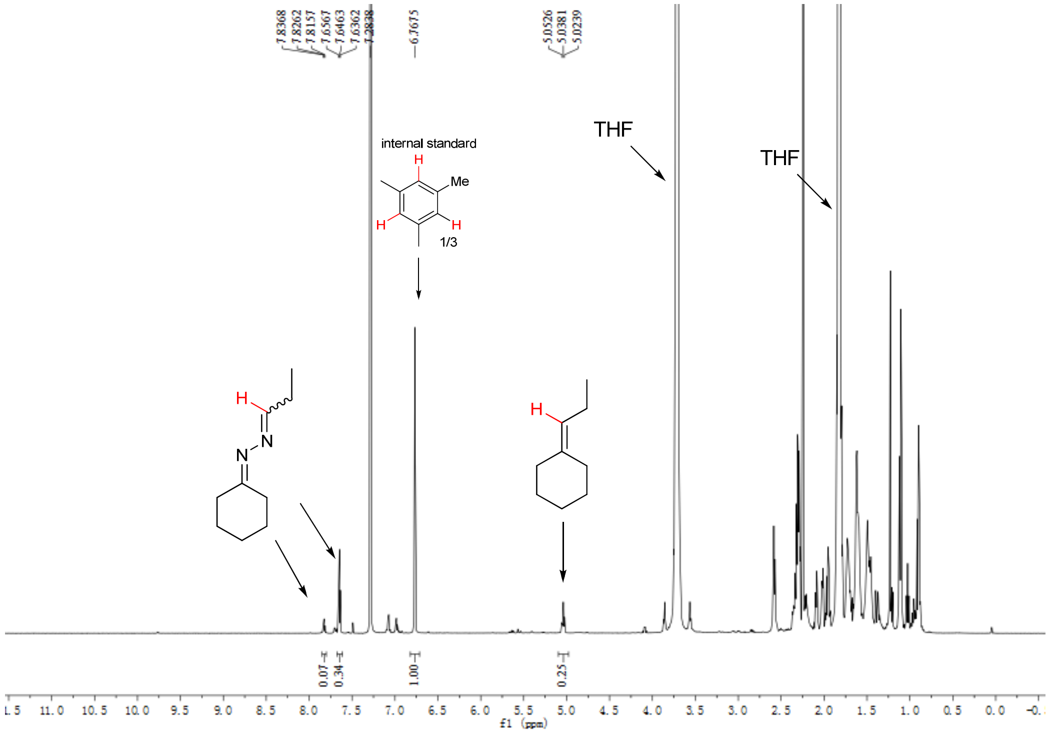

Crude NMR

1-cyclohexylidene-2-propylidenehydrazine 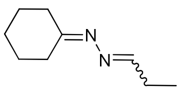 (41%yield), HRMS calc. for $C_9H_{17}N_2$ $(M+H)^+$, 153.13863; found, 153.13834.

170213-02HRAPCI-Li-WeiWei-wx-687 2/13/2017 11:19:41 AM

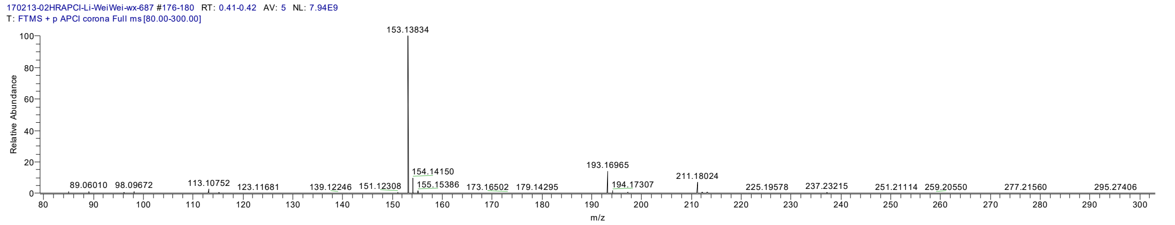

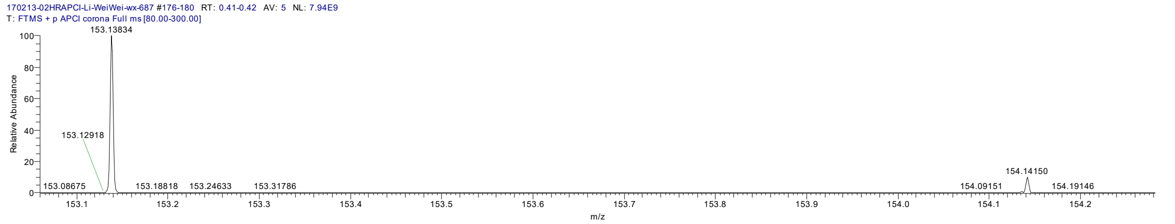

170213-02HRAPCI-Li-WeiWei-wx-687#176-180 RT: 0.41-0.42 AV: 5
T: FTMS + p APCI corona Full ms [80.00-300.00]
m/z= 153.13489-153.14501

m/z	Intensity	Relative	Resolution	Charge	Theo. Mass	Delta (ppm)	RDB equiv.	Composition
153.13834	7991873024.0	100.00	43108.14	1.00	153.13863	-1.85	2.5	$C_9H_{17}N_2$

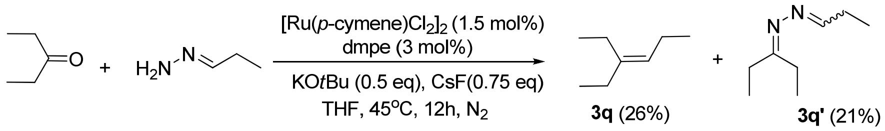

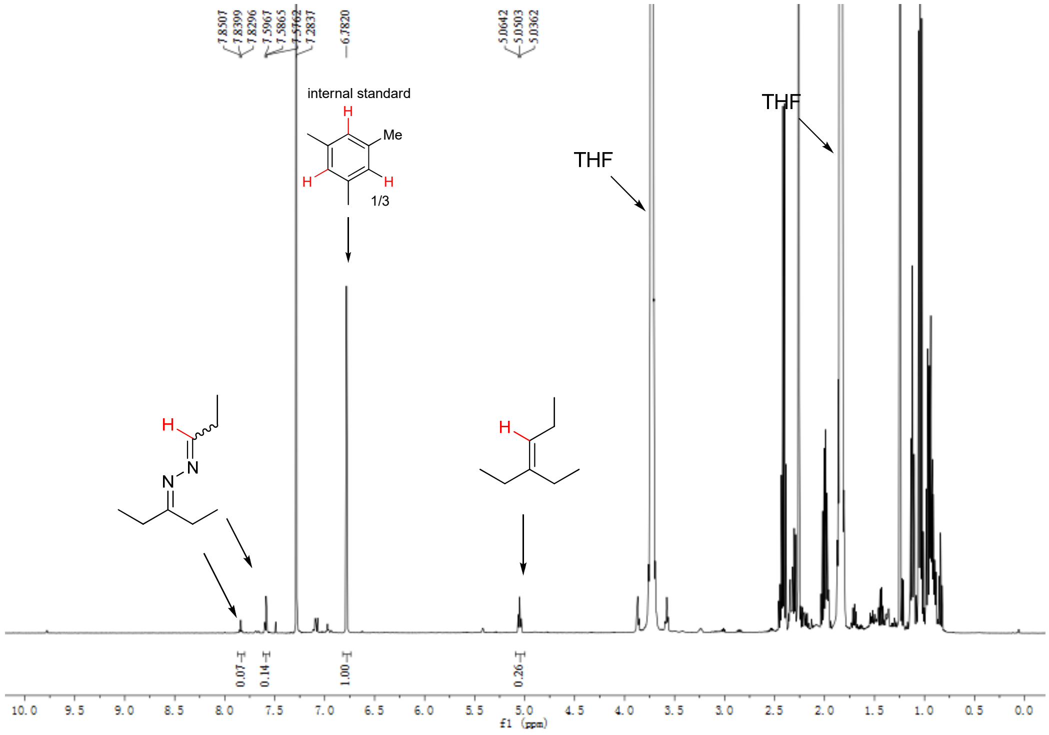

1-(pentan-3-ylidene)-2-propylidenehydrazine 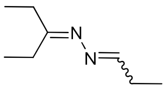 (21%yield), HRMS calc. for $C_8H_{17}N_2$ $(M+H)^+$, 141.13863; found, 141.13869.

170214-01HRAPCI-Li-Wei Wei-wx700 2/14/2017 10:22:57 AM

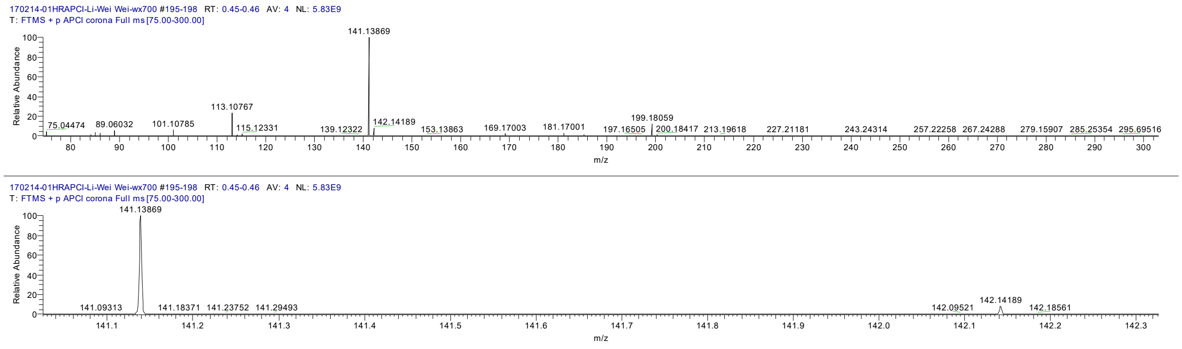

m/z	Intensity	Relative	Resolution	Charge	Theo. Mass	Delta (ppm)	RDB equiv.	Composition
141.13869	5951567360.0	100.00	46054.66	1.00	141.13863	0.45	1.5	$C_8H_{17}N_2$

m/z	Intensity	Relative	Resolution	Charge	Theo. Mass	Delta (ppm)	RDB equiv.	Composition
113.10767	1356701824.0	100.00	50451.31	1.00	113.10732	3.09	1.5	$C_6H_{13}N_2$

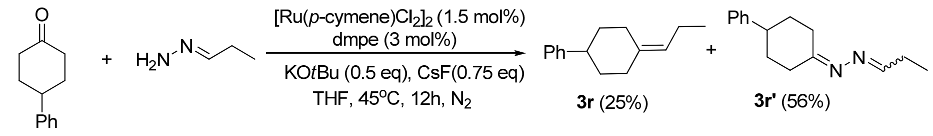

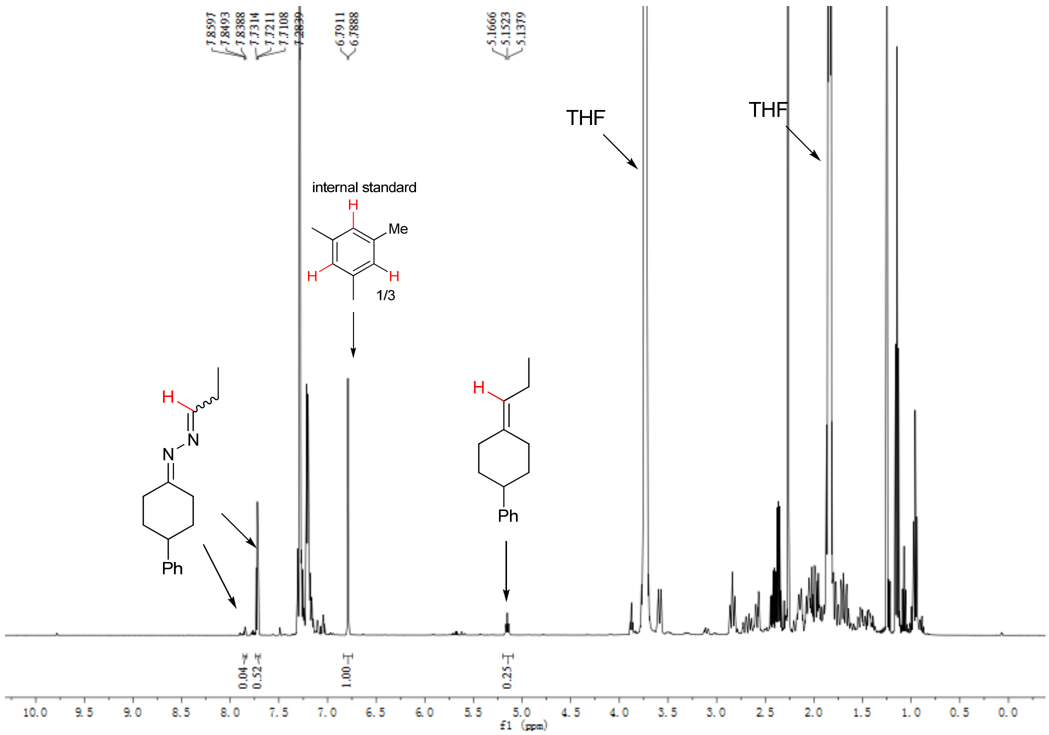

1-(4-phenylcyclohexylidene)-2-propylidenehydrazin 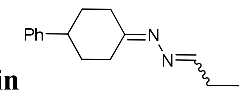 (56%yield),

HRMS calc. for $C_{15}H_{21}N_2$ $(M+H)^+$, 229.16993; found, 229.16964.

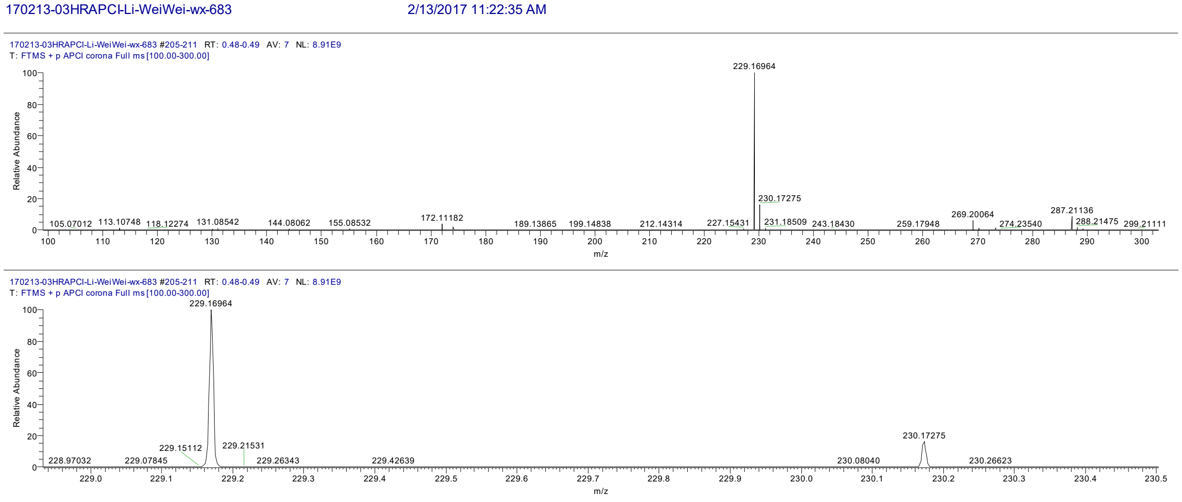

170213-03HRAPCI-Li-WeiWei-wx-683#205-211 RT: 0.48-0.49 AV: 7
T: FTMS + p APCI corona Full ms [100.00-300.00]
m/z= 229.15931-229.18177

m/z	Intensity	Relative	Resolution	Charge	Theo. Mass	Delta (ppm)	RDB equiv.	Composition
229.16964	8991578112.0	100.00	35657.69	1.00	229.16993	-1.23	6.5	$C_{15}H_{21}N_2$

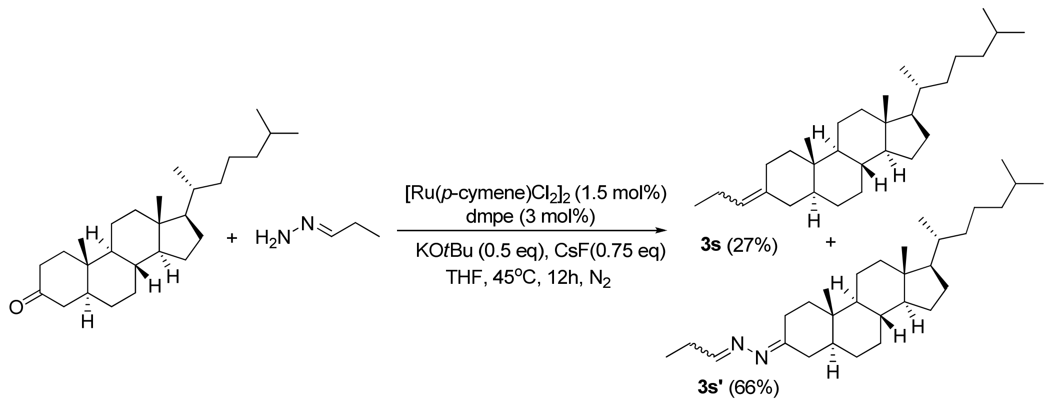

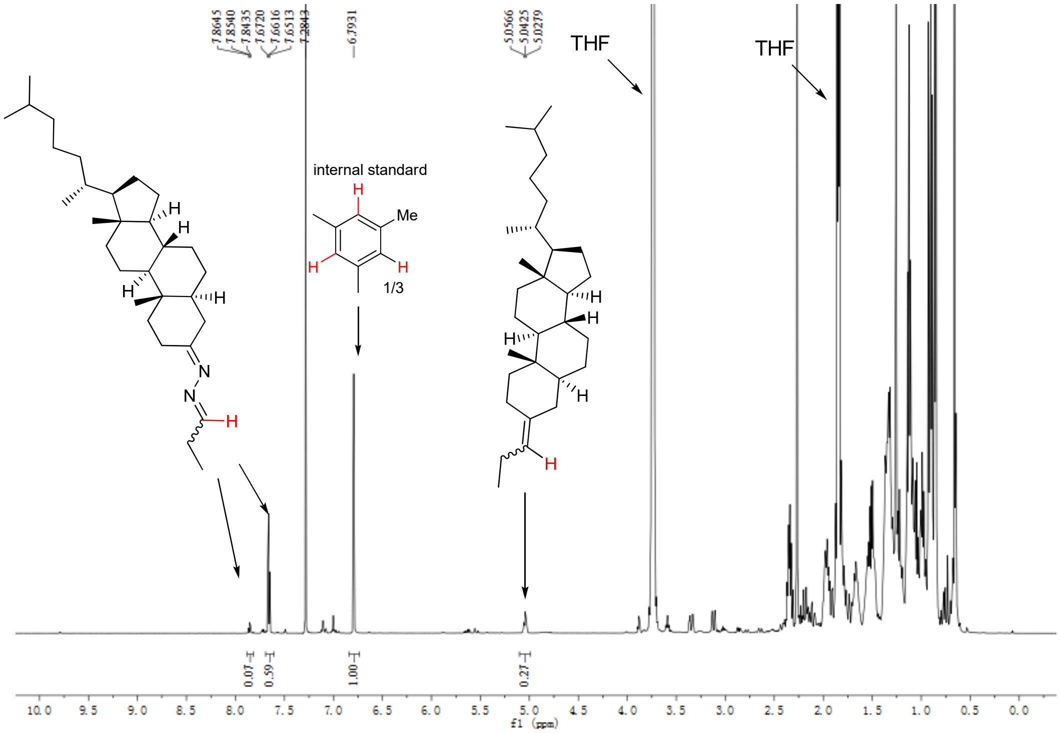

1-((5S,8R,9S,10S,13R,14S,17R)-10,13-dimethyl-17-((R)-6-methylheptan-2-yl)hexahydro-1H-cyclopenta[a]phenanthren-3(2H,4H,10H,12H,13H,14H,15H,16H,17H)-ylidene)-2-propylidenehydrazine

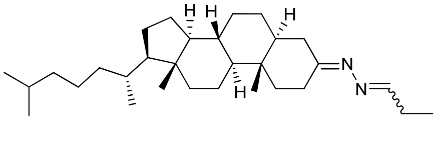 (66%yield), HRMS calc. for $C_{30}H_{53}N_2$ $(M+H)^+$, 441.42033; found, 441.41972.

170213-07HRAPCI-Li-WeiWei-wx-688 2/13/2017 11:37:29 AM

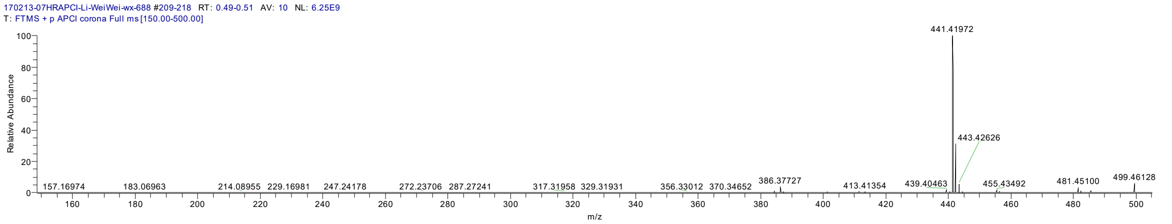

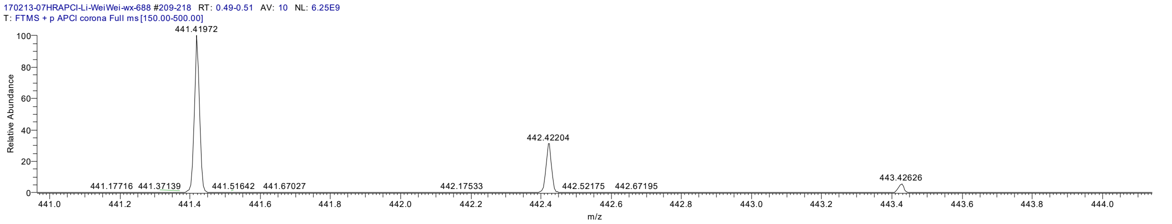

170213-07HRAPCI-Li-WeiWei-wx-688#209-218 RT: 0.49-0.51 AV: 10
T: FTMS + p APCI corona Full ms [150.00-500.00]
m/z= 441.39345-441.45088

m/z	Intensity	Relative	Resolution	Charge	Theo. Mass	Delta (ppm)	RDB equiv.	Composition
441.41972	6253150720.0	100.00	26389.25	1.00	441.42033	-1.36	5.5	$C_{30}H_{53}N_2$
					441.41765	4.71	1.0	$C_{27}H_{55}O_3N$
